# Catalytic asymmetric oxa-Diels–Alder reaction of acroleins with simple alkenes

**DOI:** 10.1038/s41467-023-39184-z

**Published:** 2023-06-14

**Authors:** Lei Zeng, Shihan Liu, Yu Lan, Lizhu Gao

**Affiliations:** 1 Xiamen Key Laboratory of Optoelectronic Materials and Advanced Manufacturing, College of Materials Science and Engineering, Huaqiao University, Xiamen, 361021 P. R. China; 2 School of Chemistry and Chemical Engineering, Chongqing Key Laboratory of Theoretical and Computational Chemistry, Chongqing University, Chongqing, 401331 P. R. China; 3 ZhengZhou JiShu Institute of AI Science, Zhengzhou, 450000 P. R. China

**Keywords:** Stereochemistry, Asymmetric catalysis, Asymmetric synthesis

## Abstract

The catalytic asymmetric inverse-electron-demand oxa-Diels–Alder (IODA) reaction is a highly effective synthetic method for creating enantioenriched six-membered oxygen-containing heterocycles. Despite significant effort in this area, simple α,β-unsaturated aldehydes/ketones and nonpolarized alkenes are seldom utilized as substrates due to their low reactivity and difficulties in achieving enantiocontrol. This report describes an intermolecular asymmetric IODA reaction between α-bromoacroleins and neutral alkenes that is catalyzed by oxazaborolidinium cation **1f**. The resulting dihydropyrans are produced in high yields and excellent enantioselectivities over a broad range of substrates. The use of acrolein in the IODA reaction produces 3,4-dihydropyran with an unoccupied C6 position in the ring structure. This unique feature is utilized in the efficient synthesis of (+)-Centrolobine, demonstrating the practical synthetic utility of this reaction. Additionally, the study found that 2,6-*trans*-tetrahydropyran can undergo efficient epimerization into 2,6-*cis*-tetrahydropyran under Lewis acidic conditions. This structural core is widespread in natural products.

## Abstract (web summary)

The asymmetric inverse-electron-demand oxa-Diels–Alder reaction (IODA) between activated α,β-unsaturated carbonyl compounds with highly polarized alkenes has been reported before but acroleins or simple alkenes were so-far rarely employed as substrates. Here, the authors study the catalytic asymmetric intermolecular IODA reaction between acroleins and simple alkenes.

## Introduction

**Fig. 1:**
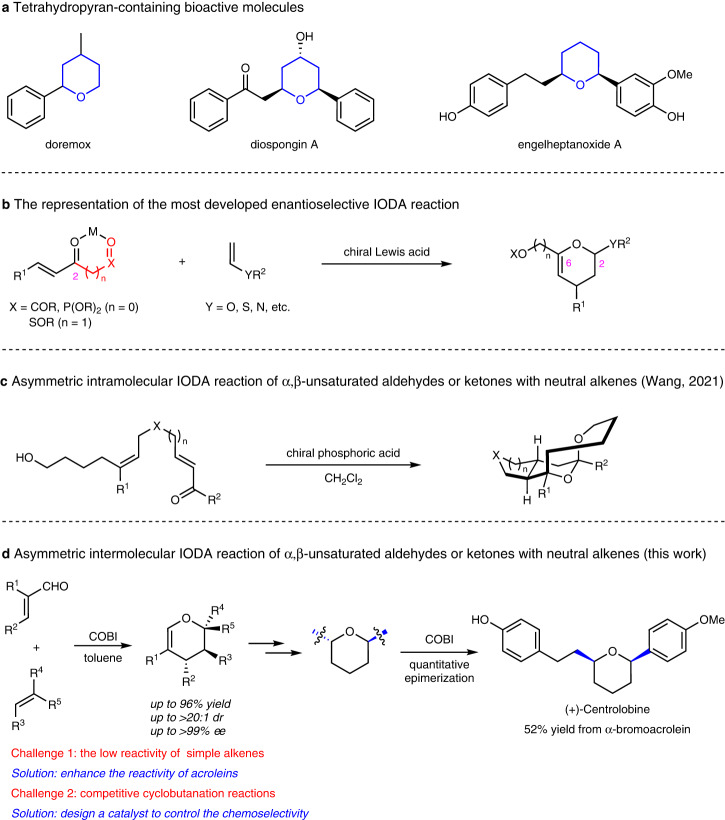
The importance of the tetrahydropyran ring and the oxa-Diels–Alder reaction of α,β-unsaturated carbonyl compounds with alkenes. **a** Importance of the tetrahydropyran ring. **b** The representation of the most developed enantioselective IODA reaction. The red part represents an ancillary group introduced in the oxodiene, and the pink 2 and 6 indicate the carbon positions. **c** Asymmetric intramolecular IODA reaction of acroleins with neutral alkenes. **d** Asymmetric intermolecular IODA reaction of acroleins with neutral alkenes (this work). Blue bonds depict the epimerization process.

The inverse-electron-demand oxa-Diels–Alder (IODA) reaction of α,β-unsaturated carbonyl compounds with alkenes is one of the most powerful methods for the construction of functionalized 3,4-dihydropyrans, a valuable precursor that has been widely applied in the synthesis of bioactive natural and synthetic compounds such as tetrahydropyran derivatives (Fig. 1a)^1–3^. Over the past few decades, considerable attention has been devoted to the development of asymmetric methodologies for IODA reactions^4–9^. Though many advances have been achieved, the distinct limitation of this reaction is its narrow substrate scope. (1) The oxodiene is limited to activated α,β-unsaturated keto compounds, i.e., with an oxygenated withdrawing group such as sulfone^10,11^, phosphonate^12–15^, or ester^16–33^ introduced at the C2 position of the oxodiene (Fig. 1b). The presence of the oxygenated group is essential for achieving high reactivity and stereoselectivity in these reactions, because it can activate the oxodiene electronically and enable the oxodiene to chelate the active center (M) of the catalyst by two-point binding^34,35^. The activated *s*-*cis* confined cyclic oxodienes^36–42^, and the highly reactive *ortho*-quinone methide intermediate^43–49^ were also used as the substrates. In sharp contrast, there were only two enantioselective intermolecular examples utilizing α,β-unsaturated aldehydes (acroleins) as the oxodiene in the chemical literature. Jacobsen’s and Ishihara’s group independently developed the IODA reaction of acrolein with highly polarized vinyl ether or vinyl sulfide^34,35^. (2) Likewise, the dienophile was restricted to highly polarized alkenes, i.e., with O, S, or N atoms attached to the double bond (Fig. 1b). In 2015, Rueping’s group successfully employed styrenes as the substrate in the enantioselective intermolecular IODA reaction with *ortho*-quinone methide intermediates^50^. The application of simple alkenes in the intermolecular IODA reaction has been rather unsuccessful even for the racemic version, which usually requires harsh conditions (mostly under high pressure) and long reaction times^51^. As the only exception, in 2013, Luo’s group developed an enantioselective IODA reaction of simple alkenes with β,γ-unsaturated ketoester^52^. Recently, Wang’s group developed a chiral phosphoric acid-catalyzed intramolecular IODA reaction of α,β-unsaturated aldehydes or ketones with neutral alkenes (Fig. 1c)^53^. To our knowledge, enantioselective intermolecular IODA reaction of α,β-unsaturated aldehydes or ketones with neutral alkenes has never been reported.

As described above, the majority of reported enantioselective IODA reactions produced 3,4-dihydropyrans with an oxygenated withdrawing group at the C6 position and with a heteroatom at the C2 position in the ring, limiting their further derivatizations (Fig. 1b)^4–9^. The development of a new catalytic strategy applying acrolein and neutral alkenes in the reaction would significantly improve the utilization of the IODA reaction, which could give the illustrated compounds shown in Fig. 1a. This will be a great challenge, as the reactivity of acroleins and the neutral alkenes was low, and furthermore the potentially competitive ene reaction and the cyclobutanation reaction should be effectively suppressed^54–56^.

α-Haloacroleins were excellent dienophiles in Diels–Alder reactions, and generally, they were more reactive than simple acroleins because of the inductive effect of the halogen^57^. In addition, the oxazaborolidinium ion (COBI) is a strong Lewis acid that has been frequently used as the catalyst in enantioselective reactions of acroleins. However, since it was first reported by Corey et al. in 2002^57^, the substituent attached to the boron atom in COBI has been an aromatic group^58,59^.

In this work, we show the catalytic enantioselective intermolecular IODA reaction of α,β-unsaturated aldehydes or ketones with neutral alkenes. In the presence of a modified COBI with an alkyl group attached to the boron atom as the catalyst, α-haloacroleins reacted with a series aryl and simple alkenes and produced densely functionalized dihydropyrans in high yields and high diastereoselectivities with excellent enantioselectivities (Fig. 1d). The ability of α-bromoacrolein to act as a masked simple acrolein was demonstrated by the efficient debromination and various cross-coupling reactions of the resulting bromo-substituted dihydropyrans.

## Results and discussion

### Reaction optimization

In 2020, we reported that in the presence of 20 mol % **1a** as the catalyst, the reaction of α-bromoacrolein with α-methylstyrene in toluene at −45 °C produced *trans*-**3** in 83% isolated yield (Table 1, entry 1)^56^. To our surprise, under similar reaction conditions, catalyst **1b** with a methyl group attached to the boron provided dihydropyran **2** as the major product. The resulting optically active 3,4-dihydropyran was provided in 49% yield (NMR yield) and 96% enantioselectivity excess (ee), with a considerable amount of *cis*- and *trans*-cyclobutane **3** (entry 2). The same reaction in dichloromethane produced *cis*-cyclobutane **3** as the major product, while polar butyronitrile afforded a complex mixture (entries 3 and 4). Then, we performed the reaction with **1b** in toluene at 0 and −78 °C and found that the yield of the desired 3,4-dihydropyran was still moderate, as the cyclobutanation reaction always followed (entries 5 and 6). Next, we performed control experiments at −20 °C and examined the effect of reaction time on chemoselectivity. With a 30-min reaction time, the yield increased to 62% with 90% enantioselective excess. After 6 h, the yield reached 83% with only trace amounts of *trans*-**3** remaining, while the ee decreased to 61% (entries 7 and 8). This result indicated that in the presence of **1b**, both *cis*- and *trans*-**3** could be converted into 3,4-dihydropyran, but the enantioselectivity of the total product **2** will diminish. Subsequently, we evaluated the substituent on boron in the catalyst and found that catalyst **1c** was the ideal choice in terms of both yield and enantioselectivity (entries 9–11). To further improve the yield and enantioselectivity, the Ar group in the catalyst was changed into 2-naphthyl, resulting in catalyst **1f** exhibiting satisfactory catalytic activity. The reaction in toluene at −40 °C for 30 min provided the desired 3,4-dihydropyran **2** in 84% isolated yield with 95% ee (entry 12).

**Table 1:** Optimization of reaction conditions^a^


Entry	Catalyst	Solvent	*T* (°C)	*t*	2:*cis*-3:*trans*-3^b^	Yield (%)^c^	ee (%)^d^
1^e^	**1a**	Toluene	−45	30 min	0:1:14	(83)	93
2	**1b**	Toluene	−40	30 min	1:0.4:0.53	49	96
3	**1b**	CH_2_Cl_2_	−40	30 min	1:2.73:0.77	21	73
4	**1b**	Butyronitrile	−40	30 min	trace	–	–
5	**1b**	Toluene	0	5 min	1:0.47:0.6	47	92
6	**1b**	Toluene	−78	1 h	1:2.14:0.83	20	95
7	**1b**	Toluene	−20	30 min	1:0.11:0.43	62	90
8	**1b**	Toluene	−20	6 h	1:0:0.12	83	61
9	**1c**	Toluene	−40	30 min	1:0.13:0.32	64	98
10	**1d**	Toluene	−40	30 min	1:0.47:0.35	47	90
11	**1e**	Toluene	−40	30 min	1:0.02:0.41	68	90
12	**1f**	Toluene	−40	30 min	1:0:0.15	86 (84)	95

^a^The reaction of α-bromoacrolein (0.27 mmol) with α-methylstyrene (0.41 mmol) was performed with 20 mol % catalyst **1**, for 30 min in 1.0 mL of toluene.

^b^Determined by ^1^H NMR analysis of the crude reaction mixture.

^c^NMR yield (isolated yield) of **2**.

^d^The ee of **2** was determined by chiral HPLC.

^e^Yield and ee of *trans*-**3**.

### Substrate scope

**Fig. 2:**
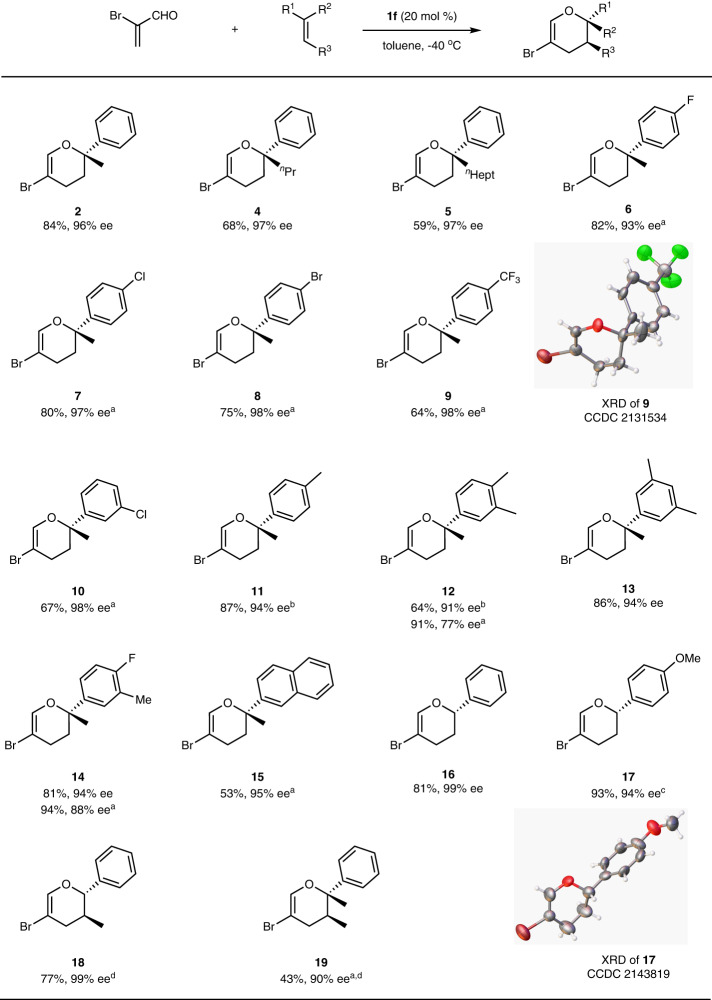
Substrate scope of alkenes. Unless otherwise noted, the reaction of α-bromoacrolein (0.27 mmol) with alkene (0.41 mmol) was performed with 20 mol % catalyst **1f** at −40 °C in 1.0 mL of toluene. Yield refers to the isolated yield, and ee was determined by chiral HPLC. ^a^At −20 °C. ^b^At −60 °C. ^c^Acrolein:alkene = 1:5.7, at −95 °C. ^d^The minor diastereomer was not found. XRD X-ray crystal diffraction analysis.

With the optimal reaction conditions determined, we evaluated this catalytic protocol using a broad range of alkenes. As depicted in Fig. 2, ^*n*^propyl and ^*n*^heptyl substituted styrene could provide the respective product (**4** and **5**) with excellent enantioselectivities, despite the relatively low yields compared with **2**. Various *para*-halogenated styrenes reacted fairly well with α-bromoacrolein and generated optically active 3,4-dihydropyran **6**–**8** in high yields with high ee. Styrene-bearing electron-withdrawing groups such as *p*-CF_3_ or *m*-Cl provided the corresponding products (**9** and **10**) in lower yields than with an electro-donating group such as *p*-Me (**11**), but high enantioselectivities were consistently observed for all products. The absolute configuration of **9** was determined to be *S* based on X-ray crystallographic analysis (the CIF file is provided in Supplementary Data). The reaction of 3,4-dimethyl phenyl styrene produced the product **12** in 64% yield and 91% ee at −60 °C, while at −20 °C the yield increased to 91% but the ee decreased to 77%. 3,5-Dimethyl and 3-methyl-4-fluoro phenyl styrene were quite suitable substrates in this reaction and provided the corresponding products (**13** and **14**) in high yields and high enantioselectivities. Naphthyl alkene in reaction delivered product **15** in a relatively low yield compared to **2**. Styrene and *para*-methoxyl phenyl styrene were also feasible substrates in the reaction (**16** and **17**), despite their tendency to polymerize under Lewis acidic conditions. The optical rotation of **16** and **17** in CHCl_3_ was positive but was negative for other dihydropyrans. The absolute configuration of product **16** was confirmed unambiguously by X-ray crystallographic analysis and the chiral center was *S*, consistent with that of compound **9**. To further investigate the diastereoselectivity of the current catalytic protocol, we employed β-methylstyrene and (*E*)−2-phenyl-2-butene as model substrates and found that they generated the corresponding products (**18** and **19**) *endo*-selectively as single isomers.

**Fig. 3:**
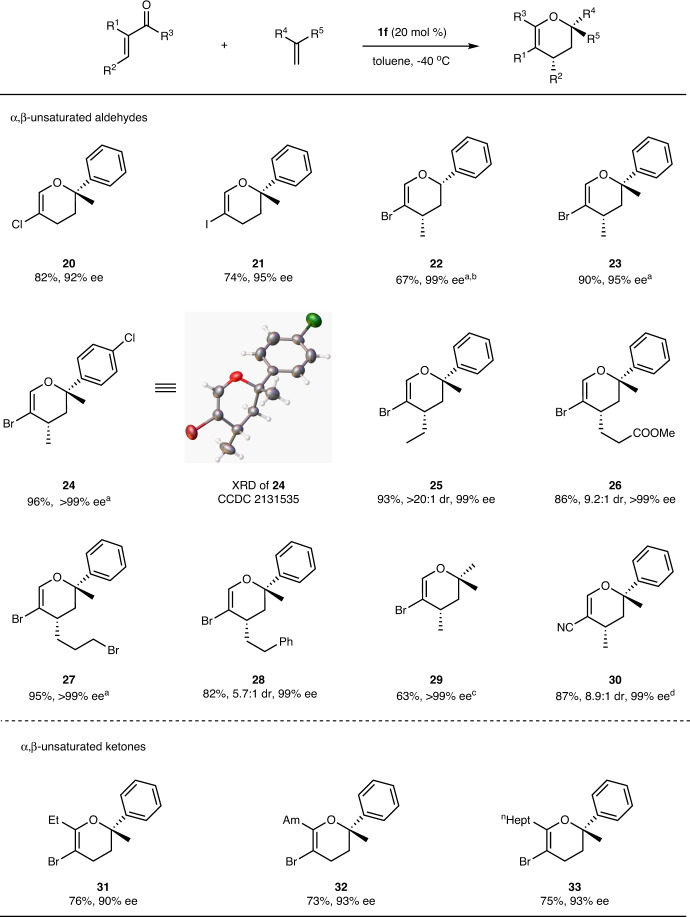
Substrate scope of α,β-unsaturated compounds. Unless otherwise noted, the reaction of α,β-unsaturated compounds (0.27 mmol) with alkene (0.41 mmol) was performed with 20 mol % catalyst **1f** at −40 °C in 1.0 mL of toluene. Yield refers to the isolated yield of major isomer, ee was determined by chiral HPLC, and the dr was determined by ^1^H NMR analysis of the crude reaction mixture. ^a^The minor diastereomer was not found. ^b^With 1.35 mmol of acrolein and 1.8 mmol of alkene. ^c^Acrolein:alkene = 1:3. ^d^Yield of both isomers. XRD X-ray crystal diffraction analysis.

Encouraged by the good results shown in Fig. 2, the substrate scope with respect to various α,β-unsaturated compounds was examined. As described in Fig. 3, α-chloro and α-iodo acroleins worked well with α-methylstyrene to give highly enantiomerically enriched 3,4-dihydropyrans **20** and **21** in high yields and high enantioselectivities. In addition, α,β-disubstituted acroleins were proven to be a more suitable substrate in the current reaction. Compared with α-monosubstituted acroleins, the corresponding products were generated in higher yields and almost complete enantiocontrol (**22**–**29**). Compound **22** was prepared from 1.5 mmol of acrolein with only 4 mol % **1f** as the catalyst. It can act as a synthetic precursor to Doremox after debromination and catalytic hydrogenation^60,61^. The absolute configuration of **24** was assigned as (2*S*, 4*S*) by X-ray diffraction analysis. It was remarkable that 2-methylpropene could react with α-bromo-β-methyl acrolein in the current catalytic system, furnishing product **29** in good yield and excellent enantioselectivity. α-Cyano-β-methyl acrolein in reaction provided compound **30** in high yield with a high diastereomeric ratio. To our delight, α-bromo-unsaturated ketones also were feasible substrates and produced **31**–**33** in good yields and high enantiomeric excess. However, the reaction of α-bromo-β-methyl and α-bromo-β-phenyl unsaturated ketones with α-methylstyrene cannot proceed even at room temperature.

**Fig. 4:**
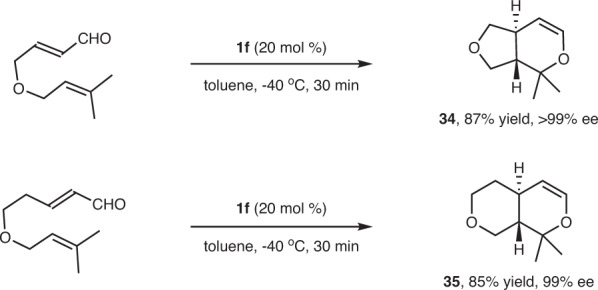
Catalytic asymmetric intramolecular IODA reactions. The reaction was performed with 0.27 mmol of the substrate in the presence of 20 mol % **1f** at −40 °C for 30 min in 1.0 mL of toluene. Yield refers to the isolated yield, and ee was determined by chiral HPLC.

To further investigate the substrate scope of the present catalytic system, we performed the catalytic asymmetric intramolecular IODA reaction. As seen in Fig. 4, the tethered sample acroleins and simple alkenes reacted very well under the standard reaction conditions, and the resulting fused bicyclic dihydropyrans **34** and **35** were generated in high yields with excellent enantioselectivities.

### Mechanistic studies

**Fig. 5:**
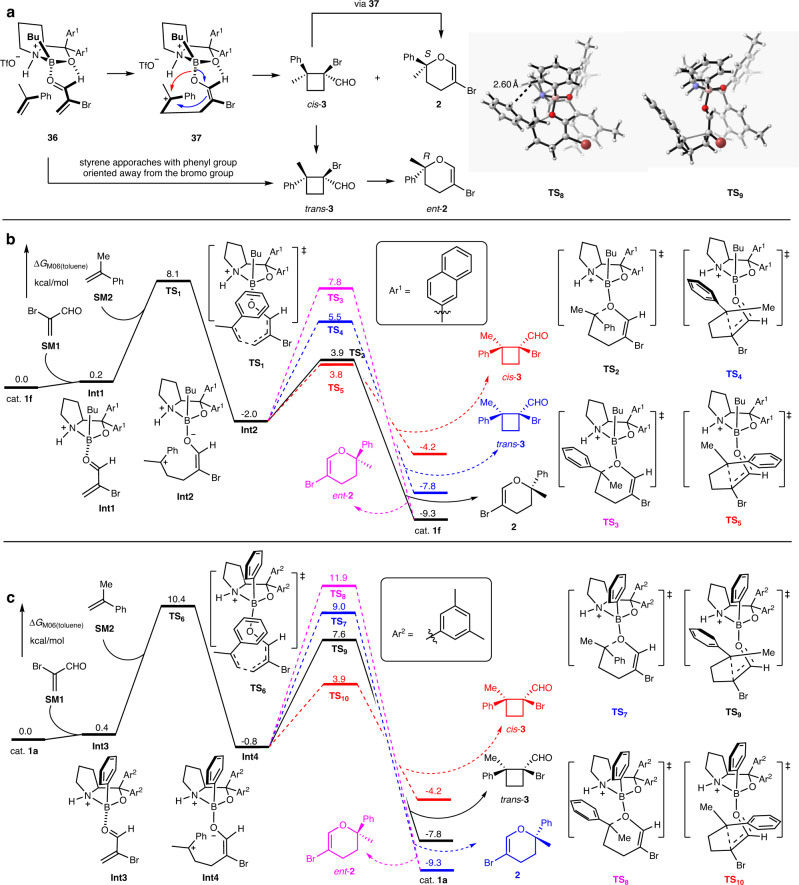
Mechanistic studies. **a** Transition-state model for the asymmetric IODA reactions. Ar^1^ 2-Naphthyl. Red and blue arrows depict the IODA reaction and cyclobutanation, respectively. *ent*-**2** enantiomer of **2**. **b** Free energy profiles of cat. **1f** catalyzed IODA reaction and formal [2+2] cycloaddition of α-bromoacrolein with α-methylstyrene. Energy values are in kcal mol^–1^ and represent the relative free energies calculated at the M06/6-311+G(d,p) SMD(toluene)//B3LYP/6-31G(d)SMD(toluene) level of theory. Source data are provided as a Source Data file. SM starting material, Int intermediate, TS transient state. Routes in pink, blue and red highlight relative free energies of the transition state for the formation of *ent*-**2,** *trans*-**3** and *cis*-**3**, respectively. **c** Free energy profiles of cat. **1a** catalyzed IODA reaction and formal [2+2] cycloaddition of α-bromoacrolein with α-methylstyrene. Source data are provided as a Source Data file. Routes in pink, blue and red highlight relative free energies of the transition state for the formation of *ent*-**2,** **2** and *cis*-**3**, respectively.

The transition-state model for the oxazaborolidinium ion **1f** catalyzed asymmetric IODA reactions is illustrated in Fig. 5a, which explains the observed stereochemical outcome. As proposed previously in cyclobutanation reactions^56^, the coordination of boron to formyl-oxygen cooperates with the binding of formyl hydrogen to the oxygen on boron, organizing the formation of complex (**36** in Fig. 5a). Then, when the styrene approaches acrolein, it must come from the front side, as the backside is effectively shield by the large naphthyl group of the **1f**. In the pre-transition-state assembly, the styrene prefers to place the phenyl ring on the same side as the Br in acrolein, because the opposite orientation would suffer from the steric repulsion between phenyl group and the ^*n*^butyl group in **1f**. Conjugate addition and subsequent cyclization reaction lead to either (2*S*)-dihydropyran **2** (red arrow) or *cis*-(1*S*,2*S*)-cyclobutane **3** (blue arrows). *cis*-**3** is a typical donor-accepter cyclobutane, and under Lewis acidic conditions, the ring is opened and isomerized into dihydropyran **2** with the same chiral center. *Trans*-**3** either arises from the addition of styrene to acrolein with the phenyl ring on the same side as the formyl group in acrolein, or it is generated from the isomerization of *cis*-**3**. Compared with *cis*-**3,** *trans*-**3** was relatively stable and converted into dihydropyran **2** sluggishly with a reversed chiral center. It should be noted that if the boronic substituent in the catalyst is a phenyl group, the steric repulsion between the boron-linked phenyl group and the phenyl group in the styrene would lead to the cyclobutane as the major product^56^.

To validate the mechanism of the cycloaddition step and reveal the catalyst-controlled chemoselectivity, density functional theory (DFT) calculations at the M06 level of theory were then performed. Either IODA reaction or [2+2] cycloaddition could occur in a stepwise manner to achieve different transformations, which would depend on the boron-linked substituent group in the catalyst. In this type of catalyst, we suspect that the steric hindrance of boron-linked substituents will play a key role in the regulation of chemoselectivity. As shown in Fig. 5b, cat. **1f** was chosen as the starting species in our theoretical study. DFT calculation showed that the electrophilic addition of β carbocation of acrolein to styrene via transition-state **TS**_**1**_ could afford zwitterionic intermediate **Int2** with a free energy barrier of 8.1 kcal mol^−1^. Then the C-O bond formation could take place via transition-state **TS**_**2**_ to yield target (*R*)-configured product **2** with an activation-free energy of 5.9 kcal mol^−1^. As a contrast, the generation of (*S*)-configured *ent*-**2** could occur via the corresponding transition-state **TS**_**3**_ with a free energy barrier of 9.8 kcal mol^−1^, which is higher than that of **TS**_**2**_. Therefore, (*S*)-configured **2** could be found as a major product, which is consistence with experimental observations. Moreover, the generation of *cis*-**3** and *trans*-**3** via enantioselective formal [2+2] cycloaddition was also considered in our DFT calculation. Starting from the common intermediate **Int2**, the second C-C bond formation would take place via transition-state **TS**_**4**_ or **TS**_**5**_, respectively, to achieve formal [2+2] cycloaddition. Interestingly, the calculated relative free energy of **TS**_**5**_ is close to **TS**_**2**_. It means the generation of *cis*-**3** is a kinetically favorable process. However, the relative free energy of *cis*-**3** is 5.1 kcal mol^−1^ higher than that of **2**. Therefore, under the Lewis acidic conditions, it would be quickly converted to thermodynamically favorable isomer **2**. Indeed, the formation of *cis*-**3** was also observed in the experiment (Table 1).

To further rationalize the effect of the boron-linked substituent on the chemoselectivity, the cat. **1a** catalyzed possible reaction pathways were also considered by DFT calculation. As shown in Fig. 5c, in the presence of cat. **1a**, the corresponding common intermediate **Int4** also could be generated. We found that the steric hindrance between the boron-linked phenyl group and the phenyl group in the styrene prevents benzylic cation from approaching the oxygen atom in acrolein, which significantly increases the energy barrier of C-O bond formation. In geometry information of **TS**_**8**_, the observed distance between H1 and H2 is only 2.60 angstrom. Meanwhile, the formation of a four-membered ring via transition-state **TS**_**9**_ was not affected by the steric hindrance of boron-linked phenyl group. The calculated barrier of ring-closing that lead to four-membered product *cis*-**3** is only 4.7 kcal mol^−1^. We found that the isomerization of *cis*-**3** to its diastereoisomer *trans*-**3** is exergonic by 3.6 kcal mol^−1^, which could occur via transition-state **TS**_**9**_ with an overall activation-free energy of 11.8 kcal mol^−1^ (from *cis*-**3** to **TS**_**9**_). Therefore, *trans*-**3** was found to be the final product in the presence of cat. **1f**. The control experiment also found that the isomerization of *cis*-**3** to *trans*-**3** could occur under the current condition. However, the reverse one cannot occur^56^. It further proved our conjecture. In this case, the calculated overall activation-free energy for the generation of six-membered product **2** is as high as 13.2 kcal mol^−1^, which is higher than that of the generation of *trans*-**3**. Therefore, the formation of **2** is kinetically unfavorable at a low reaction temperature. Our DFT calculations are in complete agreement with experimental observation.

### Late-stage derivatizations

**Fig. 6:**
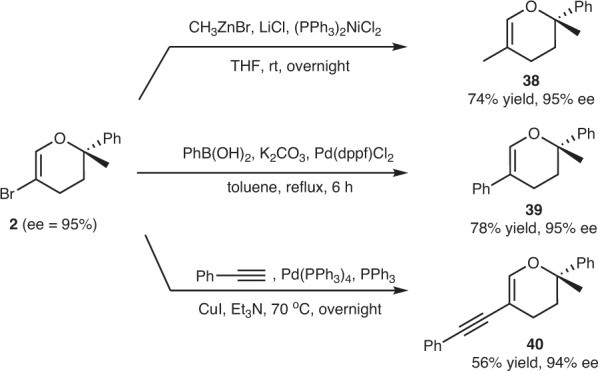
Derivatizations of 5-bromodihydropyran. Negishi reaction, Suzuki reaction and Sonogashira coupling reactions of the C(sp^2^)-Br bond of dihydropyran **2**.

The presence of the C(sp^2^)-Br bond in the ring constituted an additional advantage, and we thus examined a series of coupling reactions using **2** as illustrative examples (Fig. 6). In the presence of (PPh_3_)_2_NiCl_2_, **2** readily underwent the Negishi reaction with methyl zinc bromide at room temperature and afforded **38** in 74% yield. The Suzuki-coupling reaction was accomplished well with phenylboronic acid, as enabled by Pd(dppf)Cl_2_ while retaining the enantiomeric excess (**39**). C(sp^2^)–C(sp) bond formation was also achieved via the Sonogashira coupling reaction and provided **40** in moderate yield.

### Synthetic application

**Fig. 7:**
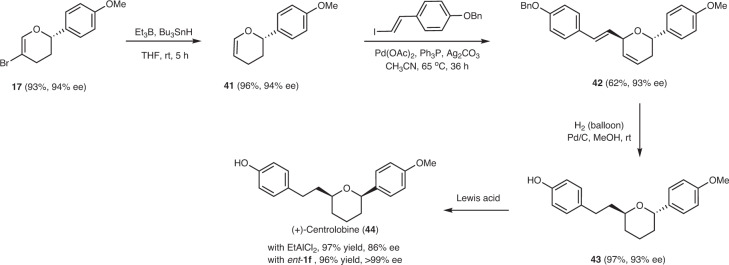
Synthesis of the (+)-centrolobine. After debromination, Heck reaction, catalytic hydrogenation and isomerization reactions, conversion of dihydropyran **17** into (+)-Centrolobine **44** was accomplished. *ent*-**1f** enantiomer of **1f**.

Utilization of acrolein in the IODA reaction provides 3,4-dihydropyran with an unoccupied C6 position in the ring structure. Taking advantage of this, the synthetic practicality of the current reaction method was demonstrated in Fig. 7. Debromination of **17** proceeded effectively using Bu_3_SnH as a reductant at room temperature and furnished **41** in 96% yield (see Supplementary Information for details). Subsequent Heck reaction of **41** with vinyl iodide gave **42** as a single regioisomer in 62% yield. Then catalytic hydrogenation was followed and generated the 6-*epi*-Centrolobine (**43**) directly. Interestingly, in the presence of oxazaborolidinium ion *ent*-**1f** or EtAlCl_2_, *trans*-configured **43** epimerized into *cis*-configured **44** almost quantitatively. With the EtAlCl_2_ as a Lewis acid, epimerization reaction in CH_3_CN provided (+)-Centrolobine **44** in 97% yield with a decreased ee (from 93% to 86%); while with *ent*-**1f** as a Lewis acid, reaction provided (+)-Centrolobine **44** in 96% yield in a single enantiomeric form (>99% ee). This result indicated that the 2,6-*cis*-tetrahydropyran was more thermodynamically stable than the 2,6-*trans* isomer because the conformation of 2,6-*cis*-disubstituted tetrahydropyran has two large substituents equatorially situated in the ring. Thus, the 2,6-disubstituted tetrahydropyran ring in the natural product always adopts the configuration with the two substituents *cis* to each other^62–64^. Compared with previous reports, our newly developed synthetic route to Centrolobine is highly efficient, and the overall yield reached up to 52% when starting from α-bromoacrolein^65–67^.

In summary, a catalytic enantioselective example of the intermolecular IODA reaction of α,β-unsaturated aldehydes or ketones with simple alkenes was developed. The resulting 3,4-dihydropyrans were produced in high yield and with a high diastereomeric ratio as well as excellent enantiomeric excess. The introduction of a bromo group in acrolein not only enhances the inherent reactivity but also facilitates the product undergoing versatile derivatizations. The substituent on the boron in oxazaborolidinium ion plays an important role on the chemoselectivity. Changing the substituent from the phenyl group to an alkyl group, the reaction would prefer IODA reaction rather than cyclobutanation. The practical utility of the developed reaction is illustrated in the quick synthesis of the (+)-Centrolobine. In the reaction process, the decomposition of *trans*-cyclobutane will cause the enantioselectivity of the target 3,4-dihydropyran to decrease. We further found that 2,6-*trans*-disubstituted tetrahydropyran can be efficiently epimerized into the more stable 2,6-*cis*-isomer under Lewis acidic conditions, which is a basic structural core in a wide range of natural products.

## Methods

### General procedure for catalytic asymmetric IODA reaction

To an aliquot of freshly prepared oxazaborolidine precursor (0.065 mmol, theoretical) in 0.73 mL of toluene at −40 °C was added trifluoromethanesulfonic acid (0.20 M solution in toluene, freshly prepared, 0.054 mmol, 0.27 mL) dropwise under Ar. After 10 min at −40 °C, a slightly yellow homogeneous catalyst solution was ready for use. To the catalyst solution were added the corresponding acroleins (0.27 mmol, 1 equiv) at −40 °C, followed by alkene (0.405 mmol, 1.5 equiv). The resulting mixture was stirred at the same temperature until complete consumption of acroleins, and then reaction was quenched with 100 μL of Et_3_N. Solvent was removed under reduced pressure, and the residue was purified by silica gel chromatography, affording the desired corresponding chiral 3,4-dihydro-2*H*-pyrans.

## Supplementary information

Supplementary Information

Description of Additional Supplementary Files

Supplementary Data 1

## Data Availability

All data supporting the findings of this study are available within the article and its Supplementary Information. Details about materials and methods, experimental procedures, characterization data, and NMR spectra are available in the Supplementary Information. Crystallographic data for structures **9**, **17** and **24** reported in this article have been deposited at the Cambridge Crystallographic Data Centre, under deposition numbers CCDC 2131534, 2143819 and 2131535, respectively. Copies of the data can be obtained free of charge via https://www.ccdc.cam.ac.uk/structures/. Source data are provided in this paper for Fig. 5b, c and Supplementary Table 1. Source Data are provided with this paper.

## Source data

Source Data
