# Supplementary material for: Catalytic asymmetric oxa-Diels–Alder reaction of acroleins with simple alkenes
Source: Nat Commun. 2023 Jun 14;14:3511. doi: 10.1038/s41467-023-39184-z (PMC10267187; doi:10.1038/s41467-023-39184-z)
Supplement: Supplementary file 1 — Supplementary Information [file 41467_2023_39184_MOESM1_ESM.pdf]

Supplementary Information for

Catalytic Asymmetric Oxa-Diels–Alder Reaction of Acroleins with Simple Alkenes

Lei Zeng[1], Shihan Liu[2], Yu Lan[2,3,*] & Lizhu Gao[1,*]

[1] Xiamen Key Laboratory of Optoelectronic Materials and Advanced Manufacturing, College of Materials Science and Engineering, Huaqiao University, Xiamen 361021, P. R. China.

[2] School of Chemistry and Chemical Engineering, Chongqing Key Laboratory of Theoretical and Computational Chemistry, Chongqing University, Chongqing 401331, P. R. China.

[3] ZhengZhou JiShu Institute of AI Science, Zhengzhou 450000, P. R. China.

* Corresponding Author. E-mail: lanyu@cqu.edu.cn; lizhugao@hqu.edu.cn.

Table of Contents

1. Supplementary Notes **2**

2. Supplementary Methods **2**

2.1 Preparation of α-bromo acroleins and (*E*)-α-cyano-β-methyl acrolein **2**

2.2 Preparation of oxazaborolidinium ion **5**

2.3 General procedure for catalytic asymmetric oxa-Diels–Alder reaction **7**

2.4 Synthesis and characterization of chiral 3,4-dihydro-2*H*-pyrans **8**

2.5 Derivatization of 5-bromodihydropyran **33**

2.6 Synthesis of the (+)-*centrolobine* **36**

3. Supplementary Discussion **39**

4. Supplementary References **130**

1. Supplementary Notes

Unless stated otherwise, reactions were carried out under a dry argon atmosphere in vacuum-flame dried glassware. Thin-layer chromatography (TLC) was performed on Merck silica gel 60 F254. Flash chromatography was performed using E. Merck silica gel (40-60 μm particle size). ^{1}H and ^{13}C NMR spectra were recorded on a Bruker AVIII-500M spectrometers at 500 and 126 MHz. Chemical shift values are reported in ppm from tetramethylsilane as the internal standard (TMS: δ 7.26 for ^{1}H and δ 77.16 for ^{13}C). Data are reported as follows: chemical shifts, integration, multiplicity (s = singlet, d = doublet, t = triplet, q = quartet, dd = doublet of doublets, dq = doublet of quartets, m = multiplet), and coupling constants (Hz). Infrared spectra were recorded on Thermo Scientific Nicolet iS50. HRMS were recorded on Agilent 1290 UHPLC/6545 Q-TOF mass spectrometer. Analytical high performance liquid chromatography (HPLC) was performed on Shimadzu Prominence LC-20 A using the indicated chiral column (4.6 mm × 25 cm). Optical rotations were measured with an Anton Paar MCP 500 polarimeter at 589 nm. The crystallographic data were collected at 100 K on a Rigaku Oxford Diffraction Supernova Dual Source, Cu at Zero equipped with an AtlasS$_2$ CCD using Cu Kα radiation. Commercial grade reagents and solvents were used without further purification except as indicated below. α-Halo acroleins were distilled from $CaSO_4$ under reduced pressure or purified by column chromatography on silica gel and stored over activated molecular sieve 4 Å at -40 °C. Commercial available arylalkenes such as styrene, α-methylstyrene and 4-methoxystyrene were dried over activated molecular sieve 4A. Butyronitrile, and dichloromethane was distilled from calcium hydride. Toluene and THF was distilled from sodium.

2. Supplementary Methods

2.1 Preparation of α-bromo acroleins and (*E*)-α-cyano-β-methyl acrolein

2.1.1 Preparation of α-bromo acroleins[1]:

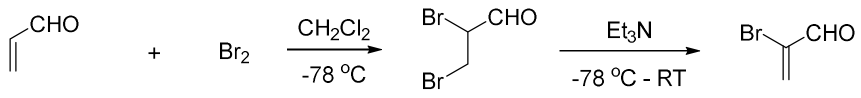

To a stirred solution of acroleins (23.1 mmol) in CH_2Cl_2 (35 mL) at -78°C under argon was added

Br_2 (1.18 mL, 23.1 mmol) over 30 min. After an additional stirring for 30 min at the same temperature, Et_3N (3.2 mL, 23.1 mmol) was added dropwise and the mixture was warmed to room temperature slowly within 2 h. The reaction was then quenched by the addition of H_2O (40 mL). The layers were separated, and the aqueous layer was extracted with CH_2Cl_2 (1 × 40 mL). The combined organic layers were washed with a 9:1 mixture of brine and 1 M HCl (40 mL), dried (Na_2SO_4), filtered and concentrated at room temperature. The residue was purified by distillation under reduced pressure from anhydrous $CaSO_4$ to give the corresponding α-bromo acroleins as a slight yellow oil.

2.1.2 Preparation of (*E*)-α-cyano-β-methyl acrolein:

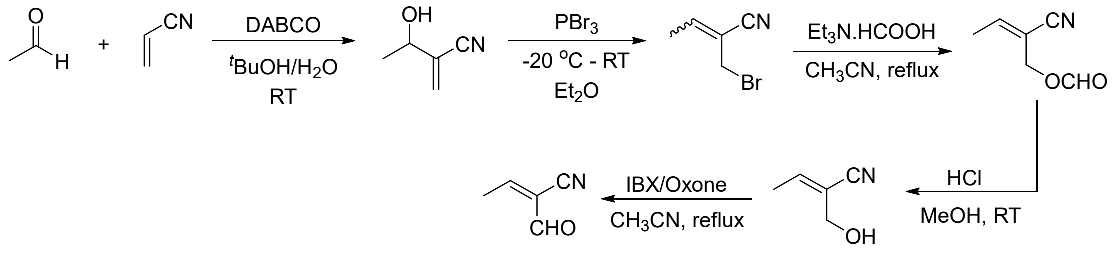

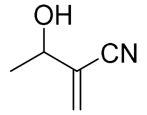

To a stirred solution of acetaldehyde (9.26 mL, 0.165 mol, 1 equiv) and acrylonitrile (10.86 mL, 0.165 mol, 1 equiv) in ^{t}butanol/water (60/40, 200 mL) was added DABCO (18.15 mL, 0.165 mol, 1 equiv) at 0 °C. Then reaction was allowed to warm to room temperature and stirred overnight. After reaction was complete, the mixture was extracted with ethyl acetate (3 × 80 mL). The combined organic phase was dried over anhydrous Na_2SO_4 and concentrated under reduced pressure. The residue was purified by distillation under reduced pressure to give the aldehyde as a colorless oil.

TLC: R_f 0.50 (hexane/ethyl acetate = 1:1);

^{1}H NMR (500 MHz, Chloroform-*d*) δ 6.02 (d, J = 1.4 Hz, 1H), 5.96 (d, J = 1.1 Hz, 1H), 4.42 (q, J = 6.5 Hz, 1H), 1.43 (d, J = 6.5 Hz, 3H).

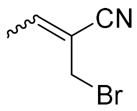

To a solution of alcohol (9.7g, 0.1 mol, 1 equiv) prepared above in anhydrous ethyl ether (200 mL) was added PBr_3 (28.2 mL, 0.3 mol, 3 equiv) at -30 °C under argon[2]. The reaction was allowed to warm to room temperature and stirred for 1 h. Then the mixture was slowly poured into precooled water (150 mL, 0 °C) with vigorous stirring and was extracted with hexane (3 × 70 mL). The combined organic phase was dried with anhydrous Na_2SO_4 and concentrated under reduced pressure. The residue was used in next step without further purification. The crude ^{1}H NMR showed it was a *E*/*Z* mixture in a ratio of 3:2.

TLC: R_f 0.50 (hexane/ethyl acetate = 5:1).

Minor: crude ^{1}H NMR (500 MHz, Chloroform-*d*) δ 6.63 (q, *J* = 7.3 Hz, 1H), 4.04 (s, 2H), 1.94 (d, *J* = 7.3 Hz, 3H);

Major: crude ^{1}H NMR (500 MHz, Chloroform-*d*) δ 6.61 (q, *J* = 7.0 Hz, 1H), 4.03 (s, 2H), 2.06 (d, *J* = 7.0 Hz, 3H).

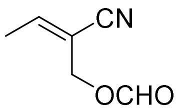

To a solution of Et_3N (6.95 mL, 50 mmol, 2 equiv) in CH_3CN (100 mL, 0 °C) at 0 °C was added HCOOH (4.75 mL, 125 mmol, 5 equiv) dropwise. The reaction was allowed to warm to room temperature and stirred for 10 min. Then the bromide (4.0 g, 25 mmol, 1 equiv) prepared above was added and the mixture was reflux overnight. After reaction was complete, the mixture was extracted with ethyl ether (3 × 40 mL). The combined organic phase was dried over anhydrous Na_2SO_4 and concentrated under reduced pressure. The residue was purified by column chromatography on silica gel to give the product as a colorless oil.

TLC: R_f 0.15 (hexane/ethyl acetate = 5:1).

^{1}H NMR (500 MHz, Chloroform-*d*) δ 8.00 (s, 1H), 6.58 (dq, *J* = 7.2, 1.0 Hz, 1H), 4.28 (s, 2H), 1.90 (d, *J* = 7.3 Hz, 3H).

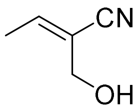

To a solution of formate prepared above in MeOH (50 mL) was added one drop of concentrated HCl and the mixture was stirred at room temperature for 3 h. Then the solvent was removed under reduced pressure. The residue was subjected to oxidation without further purification.

TLC: R_f 0.60 (hexane/ethyl acetate = 3:2).

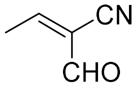

To a solution of alcohol (3.5 g, 36 mmol, 1 equiv) prepared above in anhydrous CH_3CN (100 mL) was added IBX (2.02 g, 7.2 mmol, 0.2 equiv) and Oxone ($KHSO_5$) (22.13 g, 36 mmol, 1 equiv). The mixture was reflux under argon until reaction was complete (ca. 5 h). Reaction mixture was filtered, and the filtrate was concentrated under reduced pressure. The residue was purified by column chromatography on silica gel to give the aldehyde as a slight yellow oil[3].

TLC: R_f 0.60 (hexane/ethyl acetate = 3:2);

^{1}H NMR (500 MHz, $CDCl_3$) δ 9.45 (d, J = 2.0 Hz, 1H), 7.59 (q, J = 7.5 Hz, 1H), 2.37 (dd, J = 7.5, 2.0 Hz, 3H);

^{13}C NMR (126 MHz, $CDCl_3$) δ 186.25, 164.49, 121.40, 112.14, 18.51;

IR (neat) 3063, 2959, 2871, 1712, 1457, 763, 702 cm^{-1};

HRMS (ESI) m/z: $[M + Na]^+$ Calcd. for C_5H_5NNaO: 118.0263; Found 118.0263.

2.2 Preparation of oxazaborolidinium Ion

2.2.1 Preparation of α,α-diaryl-2-pyrrolidinemethanol[4]

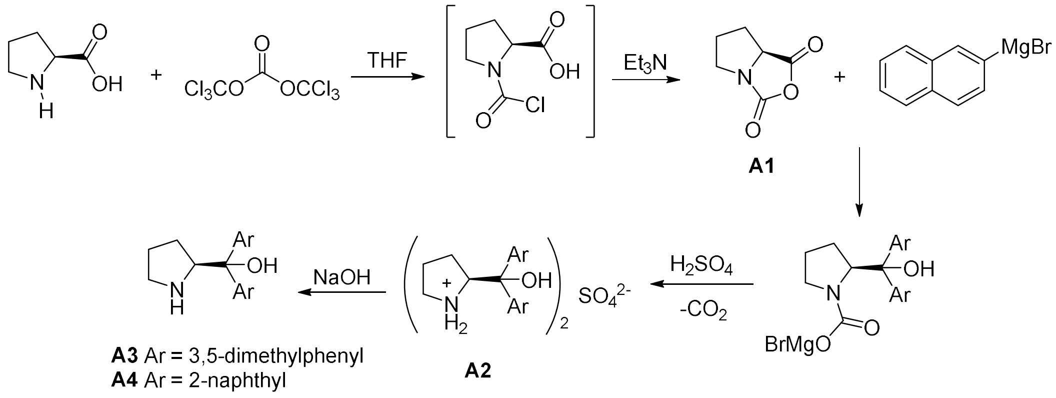

Preparation of **A1**: A 500 mL single-necked flask containing dry THF (120 mL) was charged with (*S*)-proline (11.5g, 0.1 mol). Triphosgene (10.8 g, 0.11 mol) was dissolved in 80 mL of dry toluene (slightly exothermic) in a 100 mL single-necked flask. To the suspension of proline was added the solution of triphosgene, and then the reaction mixture was warmed to 30-40 °C and stirred until be homogeneous (ca. 3 h). The reaction was stirred for another 0.5 h, and then was cooled to room temperature. The reaction mixture was concentrated to a volume of ca. 15 mL to remove the hydrogen chloride and phosgene that generated in reaction. The residue was dissolved in dry THF (180 mL), and the solution was cooled to 0-5 °C. With vigorous stirring, dry Et_3N (14.56 mL, 0.105 mol) was added slowly. After the addition was complete, the mixture was stirred for another 0.5 h at the same temperature. The mixture was filtered under reduced pressure into a 500 mL single-necked flask, and the cake of $Et_3N{\cdot}HCl$ was washed with THF (3 × 20 mL). The combined filtrate and THF washers were used immediately as **A1** was not very stable.

Preparation of **A2**: To the solution of 2-naphthylmagnesium bromide (1.0 M in THF, 300 mL, 300 mmol) at -15 °C in a 1 L single-necked flask was added the solution of **A1** over course of 1 h. The reaction was stirred for 3 h at this temperature and another 1 h at 0 °C. Then reaction mixture was poured into a precooled (0 °C) solution of 2 M aqueous H_2SO_4 (200 mL, 0.4 mol) over course of 20 min and stirred for 1 h at 0 °C. The solution was filtered under reduced pressure, and $MgSO_4$/**A2** cake was washed with hot H_2O (100 mL), EtOAc (2 × 15 mL), then dried in vacuo to afforded **A2** as a white solid.

Preparation of **A4**: To a stirred solution of THF (70 mL) and 2 M aqueous NaOH (70 mL) at room temperature was added **A2**. The mixture was stirred until all solids dissolved and was then diluted with toluene (250 mL). The two-phase mixture was filtered under reduced pressure and then partitioned, and the organic phase was washed with H_2O (30 mL). The solvent was removed under reduced pressure to afford **A4** as a white solid. Further purification of **A4** was performed by recrystallization from hexane/^{i}PrOAc.

2.2.2 Preparation of oxazaborolidinium Ion[5]

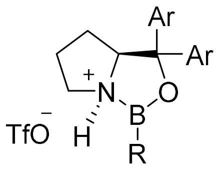

1a: Ar = 3,5-dimethylphenyl, R = phenyl
1b: Ar = 3,5-dimethylphenyl, R = methyl
1c: Ar = 3,5-dimethylphenyl, R = ^{n}butyl
1d: Ar = 3,5-dimethylphenyl, R = ^{n}octyl
1e: Ar = 3,5-dimethylphenyl, R = cyclopropyl
1f: Ar = 2-naphthyl, R = ^{n}butyl

Supplementary Fig. 1. Structures of the catalyst.

A 25-mL round-bottomed flask equipped with a magnetic stirring bar, and a Dean-Stark trap (fully charged with activated 4A molecular sieves) fitted on top with a reflux condenser and a argon balloon was charged with (*S*)-di(naphthalen-2-yl)(pyrrolidin-2-yl)methanol (0.130 mmol, 46.0 mg), alkylboronic acid (0.156 mmol, 15.9 mg) and 20 mL of toluene. The resulting mixture was maintained at 100°C for 2 hours and then was heated to reflux for 3 hours. The reaction mixture was cooled to *ca.* 60 °C and the Dean-Stark trap and condenser were quickly replaced with a short-path distillation head. The mixture was concentrated by distillation (air-cooling) to a volume *ca.* 5 mL. This distillation protocol was repeated three times by re-charging with 3 × 5 mL of toluene. The solution was then allowed to cool to room temperature and the distillation head was quickly replaced with a vacuum adaptor. Concentration in vacuo (ca. 0.1 mmHg, 1 h) afforded the corresponding oxazaborolidine as clear oil.

To an aliquot of oxazaborolidine precursor (0.065 mmol, theoretical) in 0.73 mL of toluene at -45 °C was added trifluoromethanesulfonic acid (0.20 M solution in toluene, freshly prepared, 0.054 mmol, 0.27 mL) dropwise under Ar. After 10 min at -45 °C, a slight yellow homogeneous catalyst solution was ready for use in catalytic asymmetric oxa-Diels–Alder reaction of acroleins with simple alkenes.

2.3 General procedure for catalytic asymmetric oxa-Diels–Alder reaction

To a catalyst **1f** solution prepared as described above in 1 mL of toluene was added the corresponding α-halo acroleins (0.27 mmol, 1 equiv) at -40 °C, followed by arylalkene (0.405 mmol, 1.5 equiv). The resulting mixture was stirred at the same temperature until complete consumption of α-halo acroleins, and then was quenched with 100 μL of Et_3N. Solvent was removed under

reduced pressure, and the residue was purified by silica gel chromatography, affording the desired corresponding chiral 3,4-dihydro-2*H*-pyrans.

2.4 Synthesis and characterization of chiral 3,4-dihydro-2*H*-pyrans

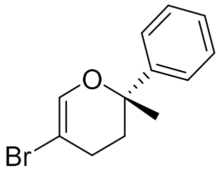

(*S*)-5-bromo-2-methyl-2-phenyl-3,4-dihydro-2*H*-pyran (2)

The compound was prepared according to general procedure with α-bromo acrolein (0.27 mmol, 36.4 mg) and α-methyl styrene (0.41 mmol, 48.5 mg) catalyzed by **1f** (20 mol %) over a course of 30 min at -40 °C. The crude material was purified by column chromatography on silica gel (eluting with hexane) to give the desired product [84% yield (57.4 mg)] as a colorless oil.

The enantiomeric purity was determined by HPLC analysis (Daicel Chiralcel AS-H, hexane/2-propanol = 99.5:0.5, 0.3 mL/min, λ = 236 nm, t_R = 13.7 min (minor) and t_R = 14.6 min (major).

TLC: R_f 0.20 (hexane);

^{1}H NMR (500 MHz, $CDCl_3$) δ 7.36 – 7.31 (m, 4H), 7.29 – 7.23 (m, 1H), 6.71 (s, 1H), 2.35 – 2.24 (m, 2H), 2.16 – 2.01 (m, 2H), 1.51 (s, 3H);

^{13}C NMR (126 MHz, $CDCl_3$) δ 144.77, 141.70, 128.53, 127.15, 124.58, 98.34, 77.72, 34.51, 28.84, 27.90;

IR (neat) 3063, 2979, 2930, 1655, 1446, 1267, 1146, 1028, 761, 700 cm^{-1};

HRMS (ESI) m/z: $[M + H]^+$ Calcd. for $C_{12}H_{14}{}^{79}BrO$: 253.0223; Found 253.0225. $[M + Na]^+$ Calcd. for $C_{12}H_{13}{}^{81}BrNaO$: 277.0021; Found 277.0022;

$[\alpha]_D^{25}$ = -32.57 (c = 1.0, $CHCl_3$; 96% ee).

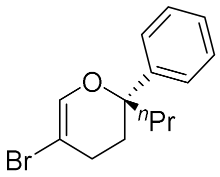

(*S*)-5-bromo-2-phenyl-2-propyl-3,4-dihydro-2*H*-pyran (4)

The compound was prepared according to general procedure with α-bromo acrolein (0.27 mmol, 36.4 mg) and pent-1-en-2-ylbenzene (0.41 mmol, 60.0 mg) catalyzed by **1f** (20 mol %) over a course of 25 min at -40 °C. The crude material was purified by column chromatography on silica gel (eluting with hexane) to give the desired product [68% yield (51.6 mg)] as a colorless oil.

The enantiomeric purity was determined by HPLC analysis (Daicel Chiralcel AS-H, hexane/2-propanol = 99.5:0.5, 0.5 mL/min, λ = 236 nm, t_R = 9.6 min (major) and t_R = 10.7 min (minor).

TLC: R_f 0.20 (hexane);

^{1}H NMR (500 MHz, $CDCl_3$) δ 7.35 – 7.32 (m, 2H), 7.28 – 7.23 (m, 3H), 6.71 (s, 1H), 2.31 – 2.22 (m, 2H), 2.13 – 2.02 (m, 2H), 1.82 – 1.76 (m, 1H), 1.72 – 1.66 (m, 1H), 1.37 – 1.26 (m, 1H), 1.09 – 0.98 (m, 1H), 0.80 (t, J = 7.0 Hz, 3H);

^{13}C NMR (126 MHz, $CDCl_3$) δ 143.18, 141.70, 128.40, 126.99, 125.23, 98.61, 80.24, 44.35, 33.02, 27.71, 16.63, 14.40;

IR (neat) 3063, 2969, 2923, 1652, 1470, 1444, 1353, 1169, 1109, 763, 700 cm^{-1};

HRMS (ESI) m/z: $[M + H]^+$ Calcd. for $C_{14}H_{18}{}^{81}BrO$: 283.0515; Found 283.0511. $[M + Na]^+$ Calcd. for $C_{14}H_{17}{}^{79}BrNaO$: 303.0355; Found 303.0354;

$[\alpha]_D^{25}$ = -58.61 (c = 0.9, $CHCl_3$; 97% ee).

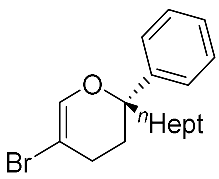

(*S*)-5-bromo-2-heptyl-2-phenyl-3,4-dihydro-2*H*-pyran (5)

The compound was prepared according to general procedure with α-bromo acrolein (0.27 mmol, 36.4 mg) and non-1-en-2-ylbenzene (0.41 mmol, 82.3 mg) catalyzed by **1f** (20 mol %) over a course of 30 min at -40 °C. The crude material was purified by column chromatography on silica gel (eluting with hexane) to give the desired product [59% yield (53.7 mg)] as a colorless oil.

The enantiomeric purity was determined by HPLC analysis (Daicel Chiralcel IG, hexane/2-propanol = 100:0, 0.5 mL/min, λ = 236 nm, t_R = 11.0 min (minor) and t_R = 11.9 min (major).

TLC: R_f 0.20 (hexane);

^{1}H NMR (500 MHz, $CDCl_3$) δ 7.35 – 7.32 (m, 2H), 7.28 – 7.24 (m, 3H), 6.71 (s, 1H), 2.31 – 2.22

(m, 2H), 2.13 – 2.02 (m, 2H), 1.83 – 1.68 (m, 2H), 1.33 – 1.17 (m, 10H), 0.84 (t, J = 7.0 Hz, 3H);

^{13}C NMR (126 MHz, $CDCl_3$) δ 143.23, 141.71, 128.41, 126.98, 125.22, 98.60, 80.25, 42.12, 33.02, 31.92, 29.92, 29.27, 27.72, 23.27, 22.75, 14.20;

IR (neat) 3063, 2929, 2850, 1654, 1467, 1444, 1158, 1121, 769, 700 cm^{-1};

HRMS (ESI) m/z: $[M + H]^+$ Calcd. for $C_{18}H_{26}{}^{81}BrO$: 339.1141; Found 339.1144. $[M + Na]^+$ Calcd. for $C_{18}H_{25}{}^{79}BrNaO$: 359.0981; Found 359.0983;

$[\alpha]_D^{25}$ = -48.49 (c = 1.0, $CHCl_3$; 97% ee).

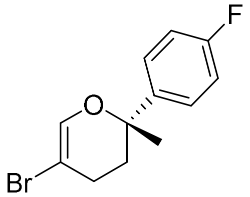

(*S*)-5-bromo-2-(4-fluorophenyl)-2-methyl-3,4-dihydro-2*H*-pyran (6)

The compound was prepared according to general procedure with α-bromo acrolein (0.27 mmol, 36.4 mg) and 1-fluoro-4-(prop-1-en-2-yl)benzene (0.41 mmol, 55.8 mg) catalyzed by **1f** (20 mol %) over a course of 25 min at -20 °C. The crude material was purified by column chromatography on silica gel (eluting with hexane) to give the desired product [82% yield (60.0 mg)] as a colorless oil. The enantiomeric purity was determined by HPLC analysis (Daicel Chiralcel OJ-H, hexane/2-propanol = 99.5:0.5, 0.5 mL/min, λ = 254 nm, t_R = 14.0 min (minor) and t_R = 14.8 min (major).

TLC: R_f 0.7 (ethyl acetate/hexane = 1:20);

^{1}H NMR (500 MHz, $CDCl_3$) δ 7.33 – 7.28 (m, 2H), 7.05 – 7.00 (m, 2H), 6.69 (t, J = 1.5 Hz, 1H), 2.34 – 2.24 (m, 2H), 2.14 – 2.03 (m, 2H), 1.50 (s, 3H);

^{13}C NMR (126 MHz, $CDCl_3$) δ 161.95 (d, J_{CF} = 245.8 Hz), 141.58, 140.58 (d, J_{CF} = 3.2 Hz), 126.40 (d, J_{CF} = 8.1 Hz), 115.37 (d, J_{CF} = 21.3 Hz), 98.39, 77.40, 34.56, 28.86, 27.87;

^{19}F NMR (471 MHz, $CDCl_3$) δ -115.93 (s);

IR (neat) 3077, 2983, 2926, 1654, 1510, 1456, 1274, 1237, 1152, 1035, 840 cm^{-1};

HRMS (ESI) m/z: $[M + H]^+$ Calcd. for $C_{12}H_{13}{}^{81}BrFO$: 273.0108; Found 273.0107. $[M + Na]^+$ Calcd. for $C_{12}H_{12}{}^{79}BrFNaO$: 292.9948; Found 292.9945;

$[\alpha]_D^{25}$ = -32.69 (c = 1.0, $CHCl_3$; 93% ee).

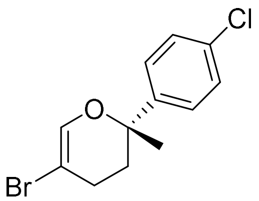

(*S*)-5-bromo-2-(4-chlorophenyl)-2-methyl-3,4-dihydro-2*H*-pyran (7)

The compound was prepared according to general procedure with α-bromo acrolein (0.27 mmol, 36.4 mg) and 1-chloro-4-(prop-1-en-2-yl)benzene (0.41 mmol, 62.6 mg) catalyzed by **1f** (20 mol %) over a course of 25 min at -20 °C. The crude material was purified by column chromatography on silica gel (eluting with hexane) to give the desired product [80% yield (62.1 mg)] as a colorless oil. The enantiomeric purity was determined by HPLC analysis (Daicel Chiralcel OJ-H, hexane/2-propanol = 99.5:0.5, 0.5 mL/min, λ = 254 nm, t_R = 13.2 min (minor) and t_R = 14.5 min (major).

TLC: R_f 0.20 (hexane);

^{1}H NMR (500 MHz, $CDCl_3$) δ 7.33 – 7.30 (m, 2H), 7.28 – 7.25 (m, 2H), 6.69 (s, 1H), 2.33 – 2.23 (m, 2H), 2.13 – 2.03 (m, 2H), 1.49 (s, 3H);

^{13}C NMR (126 MHz, $CDCl_3$) δ 143.38, 141.54, 133.02, 128.74, 126.20, 98.44, 77.39, 34.39, 28.83, 27.82;

IR (neat) 3085, 2986, 2929, 1652, 1498, 1268, 1155, 1092, 1010, 831 cm^{-1};

HRMS (ESI) m/z: $[M + H]^+$ Calcd. for $C_{12}H_{13}{}^{79}BrClO$: 286.9833; Found 286.9830. $[M + Na]^+$ Calcd. for $C_{12}H_{12}{}^{81}BrClNaO$: 310.9632; Found 310.9628;

$[\alpha]_D^{25}$ = -12.52 (c = 1.0, $CHCl_3$; 97% ee).

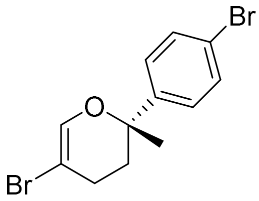

(*S*)-5-bromo-2-(4-bromophenyl)-2-methyl-3,4-dihydro-2*H*-pyran (8)

The compound was prepared according to general procedure with α-bromo acrolein (0.27 mmol, 36.4 mg) and 1-bromo-4-(prop-1-en-2-yl)benzene (0.41 mmol, 80.8 mg) catalyzed by **1f** (20 mol %) over a course of 25 min at -20 °C. The crude material was purified by column chromatography on silica gel (eluting with hexane) to give the desired product [75% yield (67.2 mg)] as a colorless oil. The enantiomeric purity was determined by HPLC analysis (Daicel Chiralcel OJ-H, hexane/2-

propanol = 99.5:0.5, 0.5 mL/min, λ = 254 nm, t_R = 14.3 min (minor) and t_R = 15.5 min (major).

TLC: R_f 0.20 (hexane);

^{1}H NMR (500 MHz, $CDCl_3$) δ 7.48 – 7.45 (m, 2H), 7.23 – 7.20 (m, 2H), 6.69 (s, 1H), 2.33 – 2.23 (m, 2H), 2.13 – 2.02 (m, 2H), 1.49 (s, 3H);

^{13}C NMR (126 MHz, $CDCl_3$) δ 143.91, 141.52, 131.69, 126.56, 121.13, 98.44, 77.42, 34.32, 28.80, 27.80;

IR (neat) 3080, 2980, 2932, 1654, 1490, 1396, 1265, 1155, 1081, 1013, 976, 825 cm^{-1};

HRMS (ESI) m/z: $[M + H]^+$ Calcd. for $C_{12}H_{13}{}^{79}Br_2O$: 330.9328; Found 330.9324;

$[\alpha]_D^{25}$ = -4.65 (c = 1.0, $CHCl_3$; 98% ee).

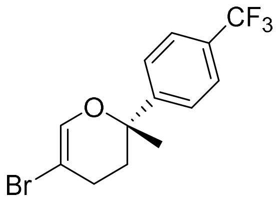

(*S*)-5-bromo-2-methyl-2-(4-(trifluoromethyl)phenyl)-3,4-dihydro-2*H*-pyran (9)

The compound was prepared according to general procedure with α-bromo acrolein (0.27 mmol, 36.4 mg) and 1-(prop-1-en-2-yl)-4-(trifluoromethyl)benzene (0.41 mmol, 76.3 mg) catalyzed by **1f** (20 mol %) over a course of 35 min at -20 °C. The crude material was purified by column chromatography on silica gel (eluting with hexane) to give the desired product [64% yield (55.5 mg)] as a white solid (mp: 50-52 °C).

The enantiomeric purity was determined by HPLC analysis (Daicel Chiralcel AS-H, hexane/2-propanol = 99.5:0.5, 0.5 mL/min, λ = 236 nm, t_R = 8.3 min (minor) and t_R = 9.0 min (major).

TLC: R_f 0.6 (ethyl acetate/hexane = 1:50);

^{1}H NMR (500 MHz, $CDCl_3$) δ 7.61 (d, J = 8.5 Hz, 2H), 7.46 (d, J = 8.5 Hz, 2H), 6.72 (s, 1H), 2.37 – 2.27 (m, 2H), 2.14 – 2.05 (m, 2H), 1.53 (s, 3H);

^{13}C NMR (126 MHz, $CDCl_3$) δ 148.87, 141.49, 129.57 (q, $^2J_{CF}$ = 32.5 Hz), 125.15, 125.63 (q, $^3J_{CF}$ = 3.5 Hz), 124.26 (q, $^1J_{CF}$ = 272.5 Hz), 98.49, 77.51, 34.34, 28.75, 27.75;

^{19}F NMR (471 MHz, $CDCl_3$) δ -62.48 (s);

IR (neat) 3085, 2986, 2941, 1652, 1623, 1410, 1328, 1163, 1126, 1081, 837 cm^{-1};

HRMS (ESI) m/z: $[M + H]^+$ Calcd. for $C_{13}H_{13}{}^{81}BrF_3O$: 323.0076; Found 323.0076. $[M + Na]^+$ Calcd.

for $C_{13}H_{12}{}^{79}BrF_3NaO$: 342.9916; Found 342.9922;

$[\alpha]_D^{25}$ = -30.07 (c = 1.0, $CHCl_3$; 98% ee).

Recrystallization from a mixture solvent (dichloromethane/pentane = 1:3) provided colorless crystals suitable for X-ray structure determination.

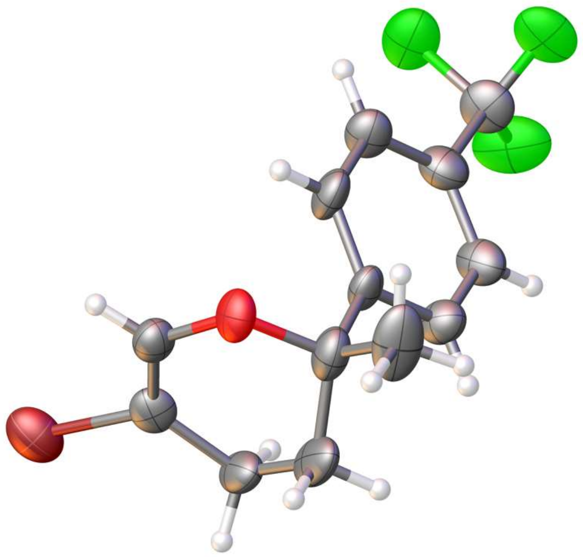

Supplementary Fig. 2. The crystal structure of **9**.

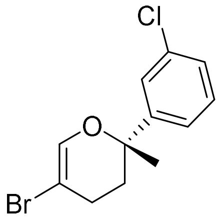

(*S*)-5-bromo-2-(4-bromophenyl)-2-methyl-3,4-dihydro-2*H*-pyran (10)

The compound was prepared according to general procedure with α-bromo acrolein (0.27 mmol, 36.4 mg) and 1-chloro-3-(prop-1-en-2-yl)benzene (0.41 mmol, 62.6 mg) catalyzed by **1f** (20 mol %) over a course of 35 min at -20 °C. The crude material was purified by column chromatography on silica gel (eluting with hexane) to give the desired product [67% yield (52.0 mg)] as a colorless oil. The enantiomeric purity was determined by HPLC analysis (Daicel Chiralcel AS-H, hexane/2-propanol = 99.5:0.5, 0.5 mL/min, λ = 236 nm, t_R = 8.8 min (minor) and t_R = 9.8 min (major).

TLC: R_f 0.4 (ethyl acetate/hexane = 1:50);

^1H NMR (500 MHz, $CDCl_3$) δ 7.33 (t, *J* = 1.5 Hz, 1H), 7.30 – 7.20 (m, 3H), 6.70 (t, *J* = 1.5 Hz, 1H), 2.35 – 2.24 (m, 2H), 2.16 – 2.04 (m, 2H), 1.50 (s, 3H);

^{13}C NMR (126 MHz, $CDCl_3$) δ 147.06, 141.49, 134.53, 129.92, 127.43, 125.10, 122.87, 98.44, 77.31, 34.31, 28.75, 27.79;

IR (neat) 3074, 2983, 2923, 1652, 1478, 1413, 1257, 1152, 1027, 692 cm^{-1};

HRMS (ESI) m/z: $[M + H]^+$ Calcd. for $C_{12}H_{13}{}^{81}BrClO$: 288.9813; Found 288.9820. $[M + Na]^+$ Calcd. for $C_{12}H_{12}{}^{79}BrClNaO$: 308.9652; Found 308.9650;

$[\alpha]_D^{25}$ = -23.28 (c = 1.0, $CHCl_3$; 98% ee).

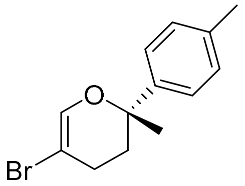

(*S*)-5-bromo-2-methyl-2-(p-tolyl)-3,4-dihydro-2*H*-pyran (11)

The compound was prepared according to general procedure with α-bromo acrolein (0.27 mmol, 36.4 mg) and 1-methyl-4-(prop-1-en-2-yl)benzene (0.41 mmol, 54.2 mg) catalyzed by **1f** (20 mol %) over a course of 30 min at -60 °C. The crude material was purified by column chromatography on silica gel (eluting with hexane) to give the desired product [87% yield (62.8 mg)] as a colorless oil. The enantiomeric purity was determined by HPLC analysis (Daicel Chiralcel AS-H, hexane/2-propanol = 99.5:0.5, 0.5 mL/min, λ = 236 nm, t_R = 7.9 min (minor) and t_R = 8.2 min (major).

TLC: R_f 0.20 (hexane);

^1H NMR (500 MHz, $CDCl_3$) δ 7.23 – 7.21 (m, 2H), 7.16 – 7.14 (m, 2H), 6.70 (s, 1H), 2.34 (s, 3H), 2.32 – 2.26 (m, 2H), 2.16 – 2.02 (m, 2H), 1.50 (s, 3H);

^{13}C NMR (126 MHz, $CDCl_3$) δ 141.86, 141.77, 136.76, 129.24, 124.55, 98.35, 77.71, 34.53, 28.97, 27.97, 21.12;

IR (neat) 3031, 2972, 2923, 1654, 1512, 1456, 1265, 1155, 1035, 814 cm^{-1};

HRMS (ESI) m/z: $[M + H]^+$ Calcd. for $C_{13}H_{16}{}^{79}BrO$: 267.0379; Found 267.0378. $[M + Na]^+$ Calcd. for $C_{13}H_{15}{}^{81}BrNaO$: 291.0178; Found 291.0180;

$[\alpha]_D^{25}$ = -19.16 (c = 1.0, $CHCl_3$; 94% ee).

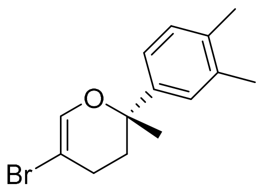

(*S*)-5-bromo-2-(3,4-dimethylphenyl)-2-methyl-3,4-dihydro-2*H*-pyran (12)

The compound was prepared according to general procedure with α-bromo acrolein (0.27 mmol, 36.4 mg) and 1,2-dimethyl-4-(prop-1-en-2-yl)benzene (0.41 mmol, 60.0 mg) catalyzed by **1f** (20 mol %) over a course of 30 min at -60 °C. The crude material was purified by column chromatography on silica gel (eluting with hexane) to give the desired product [64% yield (48.6 mg)] as a white solid (mp: 38-40 °C).

The enantiomeric purity was determined by HPLC analysis (Daicel Chiralcel OJ-H, hexane/2-propanol = 99.5:0.5, 0.5 mL/min, λ = 254 nm, t_R = 14.2 min (minor) and t_R = 19.0 min (major).

TLC: R_f 0.20 (hexane);

^{1}H NMR (500 MHz, $CDCl_3$) δ 7.11 – 7.09 (m, 2H), 7.07 – 7.05 (m, 1H), 6.70 (s, 1H), 2.32 – 2.28 (m, 2H), 2.27 (s, 3H), 2.25 (s, 3H), 2.18 – 2.11 (m, 1H), 2.07 – 2.01 (m, 1H), 1.50 (s, 3H);

^{13}C NMR (126 MHz, $CDCl_3$) δ 142.38, 141.80, 136.65, 135.43, 129.78, 125.87, 122.01, 98.31, 77.64, 34.51, 28.91, 28.00, 20.19, 19.48;

IR (neat) 3082, 2977, 2926, 1652, 1510, 1450, 1265, 1155, 1030, 820 cm^{-1};

HRMS (ESI) m/z: $[M + H]^+$ Calcd. for $C_{14}H_{18}{}^{79}BrO$: 281.0536; Found 281.0535. $[M + Na]^+$ Calcd. for $C_{14}H_{17}{}^{81}BrNaO$: 305.0334; Found 305.0329;

$[\alpha]_D^{25}$ = -12.50 (c = 0.7, $CHCl_3$; 91% ee).

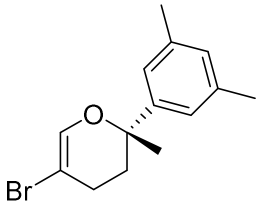

(*S*)-5-bromo-2-(3,5-dimethylphenyl)-2-methyl-3,4-dihydro-2*H*-pyran (13)

The compound was prepared according to general procedure with α-bromo acrolein (0.27 mmol, 36.4 mg) and 1,3-dimethyl-5-(prop-1-en-2-yl)benzene (0.41 mmol, 60.0 mg) catalyzed by **1f** (20 mol %) over a course of 25 min at -40 °C. The crude material was purified by column chromatography on silica gel (eluting with hexane) to give the desired product [86% yield (67.2

mg)] as a colorless oil.

The enantiomeric purity was determined by HPLC analysis (Daicel Chiralcel OJ-H, hexane/2-propanol = 99.5:0.5, 0.5 mL/min, λ = 236 nm, t_R = 8.5 min (minor) and t_R = 8.9 min (major).

TLC: R_f 0.20 (hexane);

^{1}H NMR (500 MHz, $CDCl_3$) δ 6.94 (s, 2H), 6.90 (s, 1H), 6.70 (t, *J* = 1.5 Hz, 1H), 2.31 (s, 3H), 2.30 – 2.25 (m, 2H), 2.19 – 2.12 (m, 1H), 2.07 – 2.01 (m, 1H), 1.49 (s, 3H);

^{13}C NMR (126 MHz, $CDCl_3$) δ 144.91, 141.74, 137.97, 128.83, 122.37, 98.26, 77.68, 34.54, 28.69, 27.98, 21.66;

IR (neat) 3074, 2986, 2926, 1652, 1461, 1376, 1234, 1155, 854, 706 cm^{-1};

HRMS (ESI) m/z: $[M + H]^+$ Calcd. for $C_{14}H_{18}{}^{81}BrO$: 283.0515; Found 283.0517. $[M + Na]^+$ Calcd. for $C_{14}H_{17}{}^{79}BrNaO$: 303.0355; Found 303.0356;

$[\alpha]_D^{25}$ = -20.68 (c = 1.3, $CHCl_3$; 94% ee).

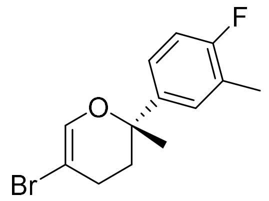

(*S*)-5-bromo-2-(4-fluoro-3-methylphenyl)-2-methyl-3,4-dihydro-2*H*-pyran (14)

The compound was prepared according to general procedure with α-bromo acrolein (0.27 mmol, 36.4 mg) and 1-fluoro-2-methyl-4-(prop-1-en-2-yl)benzene (0.41 mmol, 61.6 mg) catalyzed by **1f** (20 mol %) over a course of 25 min at -40 °C. The crude material was purified by column chromatography on silica gel (eluting with hexane) to give the desired product [81% yield (62.4 mg)] as a colorless oil.

The enantiomeric purity was determined by HPLC analysis (Daicel Chiralcel OJ-H, hexane/2-propanol = 99.5:0.5, 0.5 mL/min, λ = 236 nm, t_R = 11.2 min (minor) and t_R = 12.2 min (major).

TLC: R_f 0.6 (ethyl acetate/hexane = 1:20);

^{1}H NMR (500 MHz, $CDCl_3$) δ 7.15 – 7.09 (m, 2H), 6.95 (t, *J* = 9.0 Hz, 1H), 6.68 (t, *J* = 1.5 Hz, 1H), 2.34 – 2.23 (m, 2H), 2.27 (d, *J* = 2.0 Hz, 3H), 2.16 – 2.10 (m, 1H), 2.07 – 2.01 (m, 1H), 1.49 (s, 3H);

^{13}C NMR (126 MHz, $CDCl_3$) δ 160.47 (d, J_{CF} = 244.6 Hz), 141.61, 140.30 (d, J_{CF} = 3.5 Hz), 127.82 (d, J_{CF} = 5.0 Hz), 124.77 (d, J_{CF} = 18.0 Hz), 123.59 (d, J_{CF} = 8.0 Hz), 114.94 (d, J_{CF} = 22.5 Hz), 98.34, 77.34, 34.52, 28.82, 27.90, 14.92 (d, J_{CF} = 3.5 Hz);

^{19}F NMR (471 MHz, $CDCl_3$) δ -120.19 (s);

IR (neat) 3063, 2969, 2923, 1652, 1470, 1444, 1353, 1169, 1109, 763, 700 cm^{-1};

HRMS (ESI) m/z: $[M + H]^+$ Calcd. for $C_{13}H_{15}{}^{79}BrFO$: 285.0285; Found 285.0283. $[M + Na]^+$ Calcd. for $C_{13}H_{14}{}^{81}BrFNaO$: 309.0084; Found 309.0085;

$[\alpha]_D^{25}$ = -22.78 (c = 1.35, $CHCl_3$; 94% ee).

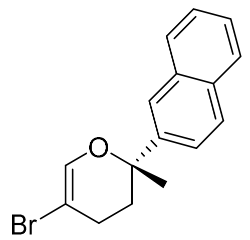

(*S*)-5-bromo-2-methyl-2-(naphthalen-2-yl)-3,4-dihydro-2*H*-pyran (15)

The compound was prepared according to general procedure with α-bromo acrolein (0.27 mmol, 36.4 mg) and 2-(prop-1-en-2-yl)naphthalene (0.41 mmol, 69.0 mg) catalyzed by **1f** (20 mol %) over a course of 25 min at -20 °C. The crude material was purified by column chromatography on silica gel (eluting with ethyl acetate/hexane = 1:100) to give the desired product [53% yield (43.4 mg)] as a colorless oil.

The enantiomeric purity was determined by HPLC analysis (Daicel Chiralcel OJ-H, hexane/2-propanol = 99.5:0.5, 1 mL/min, λ = 254 nm, t_R = 13.7 min (minor) and t_R = 17.9 min (major).

TLC: R_f 0.5 (ethyl acetate/hexane = 1:50);

^1H NMR (500 MHz, $CDCl_3$) δ 7.84 – 7.79 (m, 4H), 7.50 – 7.43 (m, 3H), 6.78 (s, 1H), 2.45 – 2.28 (m, 2H), 2.17 – 2.10 (m, 2H), 1.59 (s, 3H);

^{13}C NMR (126 MHz, $CDCl_3$) δ 142.09, 141.73, 133.35, 132.60, 128.42, 128.28, 127.64, 126.28, 126.06, 123.50, 122.98, 98.49, 77.90, 34.43, 28.91, 27.98;

IR 3057, 2977, 2926, 1654,1456, 1376, 1282, 1155, 1030, 752 (neat) cm^{-1};

HRMS (ESI) m/z: $[M + H]^+$ Calcd. for $C_{16}H_{16}{}^{79}BrO$: 303.0379; Found 303.0375. $[M + H]^+$ Calcd. for $C_{16}H_{16}{}^{81}BrO$: 305.0359; Found 305.0359;

$[\alpha]_D^{25}$ = 27.18 (c = 1.0, $CHCl_3$; 95% ee).

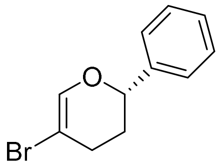

(*S*)-5-bromo-2-phenyl-3,4-dihydro-2*H*-pyran (16)

The compound was prepared according to general procedure with α-bromo acrolein (0.27 mmol, 36.4 mg) and styrene (0.41 mmol, 42.7 mg) catalyzed by **1f** (20 mol %) over a course of 30 min at -40 °C. The crude material was purified by column chromatography on silica gel (eluting with hexane) to give the desired product [81% yield (52.3 mg)] as a white solid (mp: 49-51 °C).

The enantiomeric purity was determined by HPLC analysis (Daicel Chiralcel OJ-H, hexane/2-propanol = 99.5:0.5, 1 mL/min, λ = 236 nm, t_R = 13.1 min (minor) and t_R = 17.0 min (major).

TLC: R_f 0.6 (ethyl acetate/hexane = 1:50);

^{1}H NMR (500 MHz, $CDCl_3$) δ 7.38 – 7.29 (m, 5H), 6.78 (s, 1H), 4.84 (dd, *J* = 9.5, 3.5 Hz, 1H), 2.68 – 2.60 (m, 1H), 2.41 – 2.36 (m, 1H), 2.18 – 2.07 (m, 2H);

^{13}C NMR (126 MHz, $CDCl_3$) δ 143.45, 140.60, 128.66, 128.14, 125.98, 99.19, 76.74, 31.48, 30.11;

IR (neat) 3037, 2923, 2853, 1649, 1450, 1444, 1183, 1146, 1035, 984, 757, 698cm^{-1};

HRMS (ESI) m/z: $[M + H]^+$ Calcd. for $C_{11}H_{12}{}^{79}BrO$: 239.0066; Found 239.0068. $[M + Na]^+$ Calcd. for $C_{11}H_{11}{}^{81}BrNaO$: 241.0046; Found 241.0045;

$[\alpha]_D^{25}$ = 47.73 (c = 1.0, $CHCl_3$; 99% ee).

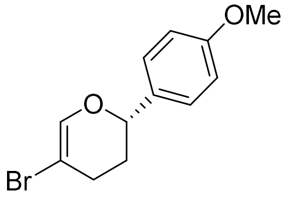

(*S*)-5-bromo-2-(4-methoxyphenyl)-3,4-dihydro-2*H*-pyran (17)

To a catalyst **1f** solution (0.108 mmol, 5 mol %) in toluene (5 mL) was added α-bromo acrolein (2.16 mmol) at -95 °C (methanol/dry ice/liquid nitrogen), followed by *p*-methoxyl styrene solution (1 mL, 3.24 M in toluene). After stirring at the same low temperature for 1 hour, the *p*-methoxyl styrene solution was added more every 30 min (3 times, 0.3 mL/time) until the complete

consumption of α-bromo acrolein. The reaction was quenched with NEt_3 and concentrated in vacuo, then the residue as purified by column chromatography on silica gel (ethyl acetate/hexane = 1:30) to give the desired product [93% yield (540.6 mg)] as a white solid (mp: 62-64 °C).

The enantiomeric purity was determined by HPLC analysis (Daicel Chiralcel AD-H, hexane/2-propanol = 95:5, 0.5 mL/min, λ = 254 nm, t_R = 14.1 min (minor) and t_R = 16.0 min (major).

TLC: R_f 0.4 (ethyl acetate/hexane = 1:20);

^{1}H NMR (500 MHz, $CDCl_3$) δ 7.27 – 7.25 (m, 2H), 6.91 – 6.88 (m, 2H), 6.76 (s, 1H), 4.80 – 4.76 (m, 1H), 3.80 (d, J = 1.0, 3H), 2.67 – 2.60 (m, 1H), 2.40 – 2.36 (m, 1H), 2.14 – 2.10 (m, 2H);

^{13}C NMR (126 MHz, $CDCl_3$) δ 159.53, 143.58, 132.69, 127.39, 114.03, 99.15, 76.51, 55.42, 31.33, 30.24;

IR (neat) 3002, 2930, 2836, 1648, 1514, 1448, 1251, 1146, 1032, 985, 832, 748 cm^{-1};

HRMS (ESI) m/z: $[M + H]^+$ Calcd. for $C_{12}H_{14}{}^{81}BrO_2$: 271.0151; Found 271.0152. $[M + Na]^+$ Calcd. for $C_{12}H_{13}{}^{79}BrNaO_2$: 290.9991; Found 290.9993;

$[\alpha]_D^{25}$ = 56.31 (c = 1.2, $CHCl_3$; 94% ee).

Recrystallization from a mixture solvent (dichloromethane/pentane = 1:3) provided colorless crystals suitable for X-ray structure determination.

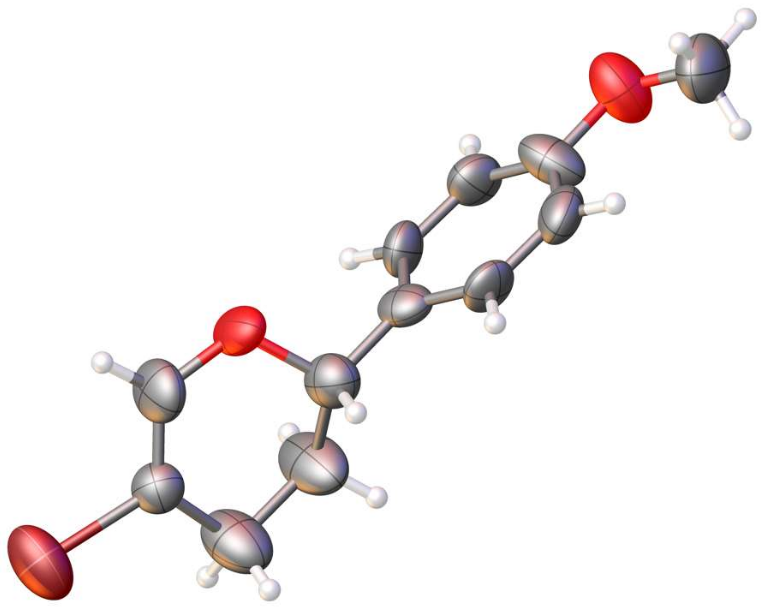

Supplementary Fig. 3. The crystal structure of **17**.

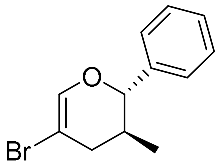

(2*S*,3*S*)-5-bromo-3-methyl-2-phenyl-3,4-dihydro-2*H*-pyran (18)

The compound was prepared according to general procedure with α-bromo acrolein (0.27 mmol, 36.4 mg) and (*E*)-prop-1-en-1-ylbenzene (0.41 mmol, 48.5 mg) catalyzed by **1f** (20 mol %) over a course of 30 min at -40 °C. The crude material was purified by column chromatography on silica gel (eluting with ethyl acetate/hexane = 1:50) to give the desired product [77% yield (52.6 mg)] as a white solid (mp: 44-46 °C).

The enantiomeric purity was determined by HPLC analysis (Daicel Chiralcel OJ-H, hexane/2-propanol = 99.5:0.5, 0.5 mL/min, λ = 236 nm, t_R = 18.5 min (minor) and t_R = 25.9 min (major).

TLC: R_f 0.3 (ethyl acetate/hexane = 1:50);

^{1}H NMR (500 MHz, $CDCl_3$) δ 7.38 – 7.28 (m, 5H), 6.76 (s, 1H), 4.37 (d, *J* = 9.5 Hz, 1H), 2.50 – 2.46 (m, 1H), 2.31 – 2.18 (m, 2H), 0.76 (d, *J* = 6.5 Hz, 3H);

^{13}C NMR (126 MHz, $CDCl_3$) δ 143.33, 139.12, 128.61, 128.50, 127.27, 98.50, 83.16, 38.43, 34.92, 17.76;

IR (neat) 3071, 2969, 2904, 1660, 1458, 1379, 1163, 1146, 1013, 700 cm^{-1};

HRMS (ESI) m/z: $[M + H]^+$ Calcd. for $C_{12}H_{14}{}^{81}BrO$: 255.0202; Found 255.0203. $[M + Na]^+$ Calcd. for $C_{12}H_{13}{}^{79}BrNaO$: 275.0042; Found 275.0043;

$[\alpha]_D^{25}$ = 56.47 (c = 1.0, $CHCl_3$; 99% ee).

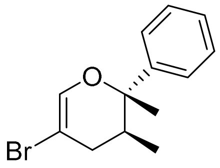

(2*S*,3*S*)-5-bromo-2,3-dimethyl-2-phenyl-3,4-dihydro-2*H*-pyran (19)

The compound was prepared according to general procedure with α-bromo acrolein (0.27 mmol, 36.4 mg) and (*E*)-but-2-en-2-ylbenzene (0.41 mmol, 54.2 mg) catalyzed by **1f** (20 mol %) over a course of 50 min at -20 °C. The crude material was purified by column chromatography on silica

gel (eluting with hexane) to give the desired product [43% yield (31.1 mg)] as a white solid (mp: 53-55 °C).

The enantiomeric purity was determined by HPLC analysis (Daicel Chiralcel OJ-H, hexane/2-propanol = 99.0:1.0, 1 mL/min, λ = 220 nm, t_R = 7.7 min (minor) and t_R = 10.6 min (major).

TLC: R_f 0.7 (ethyl acetate/hexane = 1:20);

^1H NMR (500 MHz, $CDCl_3$) δ 7.37 – 7.33 (m, 4H), 7.29 – 7.27 (m, 1H), 6.69 (s, 1H), 2.34 – 2.28 (m, 2H), 2.11 – 2.06 (m, 1H), 1.48 (s, 3H), 0.92 (d, J = 6.5 Hz, 3H);

^{13}C NMR (126 MHz, $CDCl_3$) δ 144.94, 141.39, 128.37, 127.37, 125.30, 97.38, 80.92, 37.47, 36.44, 20.89, 15.89;

IR (neat) 3068, 2980, 2915, 1654, 1450, 1385, 1268, 1158, 1016, 1004, 766, 709 cm^{-1};

HRMS (ESI) m/z: $[M + H]^+$ Calcd. for $C_{13}H_{16}{}^{81}BrO$: 269.0359; Found 269.0359. $[M + Na]^+$ Calcd. for $C_{13}H_{15}{}^{79}BrNaO$: 289.0198; Found 289.0199;

$[\alpha]_D^{25}$ = -32.56 (c = 0.9, $CHCl_3$; 90% ee).

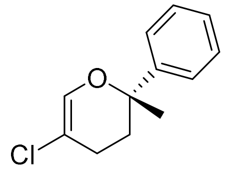

(*S*)-5-chloro-2-methyl-2-phenyl-3,4-dihydro-2*H*-pyran (20)

The compound was prepared according to general procedure with α-chloro acrolein (0.27 mmol, 37.1 mg) and α-methyl styrene (0.41 mmol, 48.5 mg) catalyzed by **1f** (20 mol %) over a course of 25 min at -40 °C. The crude material was purified by column chromatography on silica gel (eluting with hexane) to give the desired product [82% yield (46.2 mg)] as a colorless oil.

The enantiomeric purity was determined by HPLC analysis (Daicel Chiralcel AS-H, hexane/2-propanol = 99.5:0.5, 0.5 mL/min, λ = 236 nm, t_R = 8.2 min (minor) and t_R = 8.8 min (major).

TLC: R_f 0.2 (hexane);

^1H NMR (500 MHz, $CDCl_3$) δ 7.34 (d, J = 4.5 Hz, 4H), 7.28 – 7.24 (m, 1H), 6.65 (s, 1H), 2.32 – 2.28 (m, 1H), 2.24 – 2.18 (m, 1H), 2.07 – 2.00 (m, 2H), 1.52 (s, 3H);

^{13}C NMR (126 MHz, $CDCl_3$) δ 144.75, 139.92, 128.55, 127.16, 124.63, 110.22, 77.88, 33.62, 28.87, 26.25;

IR (neat) 3065, 2992, 2926, 1663, 1450, 1189, 1155, 1038, 769, 698 cm^{-1};

HRMS (ESI) m/z: $[M + H]^+$ Calcd. for $C_{12}H_{14}ClO$: 209.0728; Found 209.0728;

$[\alpha]_D^{25}$ = -25.20 (c = 1.0, $CHCl_3$; 92% ee).

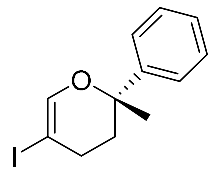

(*S*)-5-iodo-2-methyl-2-phenyl-3,4-dihydro-2*H*-pyran (21)

The compound was prepared according to general procedure with α- iodo acrolein (0.27 mmol, 74.6 mg) and α-methyl styrene (0.41 mmol, 48.5 mg) catalyzed by **1f** (20 mol %) over a course of 25 min at -40 °C. The crude material was purified by column chromatography on silica gel (eluting with hexane) to give the desired product [74% yield (60.0 mg)] as a slight brown solid (mp: 51-53 °C).

The enantiomeric purity was determined by HPLC analysis (Daicel Chiralcel AD-H, hexane/2-propanol = 99.5:0.5, 0.5 mL/min, λ = 236 nm, t_R = 9.7 min (minor) and t_R = 10.6 min (major).

TLC: R_f 0.2 (hexane);

^{1}H NMR (500 MHz, $CDCl_3$) δ 7.36 – 7.32 (m, 4H), 7.28 – 7.25 (m, 1H), 6.77 (s, 1H), 2.34 – 2.25 (m, 2H), 2.14 – 2.05 (m, 2H), 1.51 (s, 3H);

^{13}C NMR (126 MHz, $CDCl_3$) δ 146.11, 144.96, 128.54, 127.14, 124.57, 77.44, 67.41, 35.58, 30.87, 28.98;

IR (neat) 3063, 2986, 2926, 1637, 1453, 1263, 1149, 1027, 760, 703 cm^{-1};

HRMS (ESI) m/z: $[M + H]^+$ Calcd. for $C_{12}H_{14}IO$: 301.0084; Found 301.0088;

$[\alpha]_D^{25}$ = -40.67 (c = 1.0, $CHCl_3$; 95% ee).

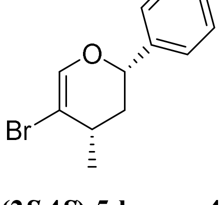

(2*S*,4*S*)-5-bromo-4-methyl-2-phenyl-3,4-dihydro-2*H*-pyran (22)

The compound was prepared according to general procedure with (*Z*)-2-bromobut-2-enal (1.35

mmol, 201.1 mg) and styrene (1.8 mmol, 187.5 mg) catalyzed by **1f** (4 mol %) over a course of 3 days at -40 °C. The crude material was purified by column chromatography on silica gel (eluting with hexane) to give the desired product [67% yield (229.0 mg)] as a colorless oil.

The enantiomeric purity was determined by HPLC analysis (Daicel Chiralcel OJ-H, hexane/2-propanol = 99.5:0.5, 1 mL/min, λ = 236 nm, t_R = 10.1 min (minor) and t_R = 13.3 min (major).

TLC: R_f 0.8 (ethyl acetate/hexane = 1:20);

^{1}H NMR (500 MHz, $CDCl_3$) δ 7.38 – 7.29 (m, 5H), 6.76 (s, 1H), 4.91 (d, *J* = 11.5 Hz, 1H), 2.77 – 2.70 (m, 1H), 2.29 (dd, *J* = 13.5, 5.5 Hz, 1H), 1.87 – 1.80 (m, 1H), 1.17 (d, *J* = 7.0 Hz, 3H);

^{13}C NMR (126 MHz, $CDCl_3$) δ 143.34, 140.50, 128.65, 128.21, 126.04, 107.73, 78.14, 41.39, 33.62, 20.43;

IR (neat) 3065, 2975, 2918, 1643, 1456, 1231, 1163, 996, 763, 700 cm^{-1};

HRMS (ESI) m/z: $[M + H]^+$ Calcd. for $C_{12}H_{14}{}^{81}BrO$: 255.0202; Found 255.0203. $[M + Na]^+$ Calcd. for $C_{12}H_{13}{}^{79}BrNaO$: 275.0042; Found 275.0043;

$[\alpha]_D^{25}$ = 12.48 (c = 1.0, $CHCl_3$; 99% ee).

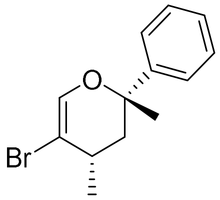

(2*S*,4*S*)-5-bromo-2,4-dimethyl-2-phenyl-3,4-dihydro-2*H*-pyran (23)

The compound was prepared according to general procedure with (*Z*)-2-bromobut-2-enal (0.27 mmol, 40.2 mg) and prop-1-en-2-ylbenzene (0.41 mmol, 48.5 mg) catalyzed by **1f** (20 mol %) over a course of 18 hours at -40 °C. The crude material was purified by column chromatography on silica gel (eluting with hexane) to give the desired product [90% yield (64.9 mg)] as a colorless oil.

The enantiomeric purity was determined by HPLC analysis (Daicel Chiralcel AD-H, hexane/2-propanol = 99.5:0.5, 0.5 mL/min, λ = 254 nm, t_R = 8.0 min (minor) and t_R = 8.6 min (major).

TLC: R_f 0.8 (ethyl acetate/hexane = 1:20);

^{1}H NMR (500 MHz, $CDCl_3$) δ 7.41 – 7.39 (m, 2H), 7.36 – 7.33 (m, 2H), 7.28 – 7.24 (m, 1H), 6.70 (d, *J* = 2.0 Hz, 1H), 2.61 – 2.54 (m, 1H), 2.21 (dd, *J* = 13.5, 6.5 Hz, 1H), 1.81 (dd, *J* = 13.5, 10.0 Hz, 1H), 1.58 (s, 3H), 1.07 (d, *J* = 6.5 Hz, 3H);

^{13}C NMR (126 MHz, $CDCl_3$) δ 145.99, 141.21, 128.41, 127.21, 124.46, 105.70, 78.24, 44.77, 30.91,

24.74, 19.98;

IR (neat) 3060 2986, 2932, 1640, 1493, 1376, 1254, 1166, 1121, 979, 763, 700 cm^{-1};

HRMS (ESI) m/z: $[M + H]^+$ Calcd. for $C_{13}H_{16}{}^{79}BrO$: 267.0379; Found 267.0377. $[M + H]^+$ Calcd. for $C_{13}H_{16}{}^{81}BrO$: 269.0359; Found 269.0359;

$[\alpha]_D^{25}$ = -7.67 (c = 2.0, $CHCl_3$; 95% ee).

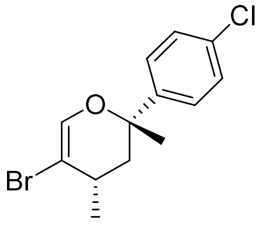

(2*S*,4*S*)-5-bromo-4-methyl-2-phenyl-3,4-dihydro-2*H*-pyran (24)

The compound was prepared according to general procedure with (*Z*)-2-bromobut-2-enal (0.27 mmol, 40.2 mg) and 1-chloro-4-(prop-1-en-2-yl)benzene (0.41 mmol, 62.6 mg) catalyzed by **1f** (20 mol %) over a course of 3 days at -40 °C. The crude material was purified by column chromatography on silica gel (eluting with hexane) to give the desired product [96% yield (78.2 mg)] as a colorless oil.

The enantiomeric purity was determined by HPLC analysis (Daicel Chiralcel OD-H, hexane/2-propanol = 100:0, 0.5 mL/min, λ = 236 nm, t_R = 15.1 min (minor) and t_R = 17.2 min (major).

TLC: R_f 0.2 (hexane);

^1H NMR (500 MHz, $CDCl_3$) δ 7.34 – 7.30 (m, 4H), 6.68 (d, *J* = 2.0 Hz, 1H), 2.61 – 2.53 (m, 1H), 2.19 (dd, *J* = 13.5, 6.0 Hz, 1H), 1.77 (dd, *J* = 13.5, 10.0 Hz, 1H), 1.56 (s, 3H), 1.08 (d, *J* = 7.0 Hz, 3H);

^{13}C NMR (126 MHz, $CDCl_3$) δ 144.59, 140.99, 133.04, 128.54, 126.04, 105.79, 77.89, 44.63, 30.85, 24.76, 19.97;

IR (neat) 3087, 2989, 2931, 1659, 1372, 1259, 1183, 1107, 1043, 837 cm^{-1};

HRMS (ESI) m/z: $[M + H]^+$ Calcd. for $C_{13}H_{15}{}^{79}BrClO$: 300.9989; Found 300.9985. $[M + H]^+$ Calcd. for $C_{13}H_{15}{}^{81}BrClO$: 302.9969; Found 302.9970;

$[\alpha]_D^{25}$ = -19.87 (c = 0.9, $CHCl_3$; 99.5% ee).

Recrystallization from a mixture solvent (dichloromethane/pentane = 1:3) provided colorless

crystals suitable for X-ray structure determination.

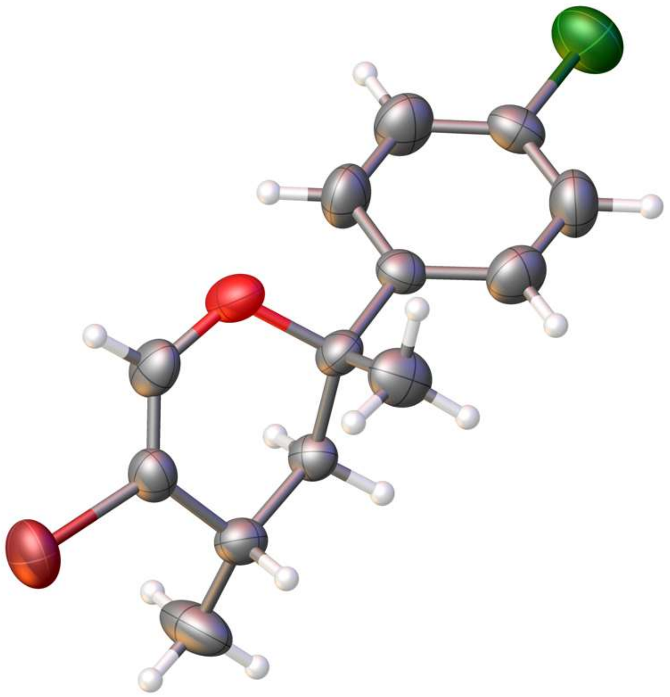

Supplementary Fig. 4. The crystal structure of **24**.

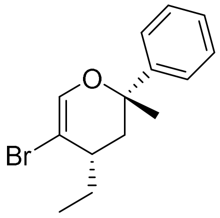

(2*S*,4*S*)-5-bromo-4-ethyl-2-methyl-2-phenyl-3,4-dihydro-2*H*-pyran (25)

The compound was prepared according to general procedure with (*Z*)-2-bromopent-2-enal (0.27 mmol, 44.0 mg) and prop-1-en-2-ylbenzene (0.41 mmol, 48.5 mg) catalyzed by **1f** (20 mol %) over a course of 18 hours at -40 ^{o}C. The crude material was purified by column chromatography on silica gel (eluting with hexane) to give the desired product [93% yield (70.6 mg)] as a colorless oil.

The enantiomeric purity was determined by HPLC analysis (Daicel Chiralcel AD-H, hexane/2-propanol = 100:0, 0.5 mL/min, λ = 254 nm, t_R = 12.1 min (minor) and t_R = 12.8 min (major).

TLC: R_f 0.8 (ethyl acetate/hexane = 1:20);

^{1}H NMR (500 MHz, $CDCl_3$) δ 7.42 – 7.41 (m, 2H), 7.37 – 7.34 (m, 2H), 7.28 – 7.25 (m, 1H), 6.73 (d, J = 1.5 Hz, 1H), 2.46 – 2.40 (m, 1H), 2.14 (dd, J = 14.0, 6.5 Hz, 1H), 1.88 – 1.80 (m, 2H), 1.59 (s, 3H), 1.29 – 1.20 (m, 1H), 0.86 (t, J = 7.5 Hz, 3H);

^{13}C NMR (126 MHz, $CDCl_3$) δ 146.23, 141.92, 128.42, 127.23, 124.43, 105.21, 78.26, 41.26, 36.54, 25.90, 24.52, 10.19;

IR (neat) 3065, 2975, 2929, 1637, 1447, 1189, 1163, 1123, 1021, 766, 698 cm^{-1};

HRMS (ESI) m/z: $[M + H]^+$ Calcd. for $C_{14}H_{18}{}^{81}BrO$: 283.0515; Found 283.0518. $[M + Na]^+$ Calcd. for $C_{14}H_{17}{}^{79}BrNaO$: 303.0355; Found 303.0357;

$[\alpha]_D^{25}$ = -6.24 (c = 1.9, $CHCl_3$; 99% ee).

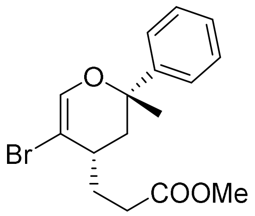

methyl 3-((2*S*,4*S*)-5-bromo-2-methyl-2-phenyl-3,4-dihydro-2*H*-pyran-4-yl)propanoate (26)

The compound was prepared according to general procedure with methyl (*Z*)-5-bromo-6-oxohex-4-enoate (0.27 mmol, 59.7 mg) and prop-1-en-2-ylbenzene (0.41 mmol, 48.5 mg) catalyzed by **1f** (20 mol %) over a course of 18 hours at -40 °C. The crude material was purified by column chromatography on silica gel (eluting with ethyl acetate/hexane = 1:20) to give the desired product [86% yield (78.8 mg)] as a colorless oil.

The enantiomeric purity was determined by HPLC analysis (Daicel Chiralcel IC, hexane/2-propanol = 99.5:0.5, 1 mL/min, λ = 236 nm, t_R = 14.3 min (major).

TLC: R_f 0.2 (ethyl acetate/hexane = 1:20);

^1H NMR (500 MHz, $CDCl_3$) δ 7.40 – 7.38 (m, 2H), 7.36 – 7.33 (m, 2H), 7.28 – 7.25 (m, 1H), 6.75 (d, J = 2.0 Hz, 1H), 3.65 (s, 3H), 2.59 – 2.52 (m, 1H), 2.39 – 2.33 (m, 1H), 2.28 – 2.21 (m, 1H), 2.17 – 2.08 (m, 2H), 1.83 (dd, J = 13.5, 10.0 Hz, 1H), 1.58 (s, 3H), 1.60 – 1.53 (m, 1H);

^{13}C NMR (126 MHz, $CDCl_3$) δ 173.69, 145.75, 142.41, 128.45, 127.33, 124.34, 103.63, 78.13, 51.73, 41.28, 34.72, 30.47, 28.14, 24.57;

IR (neat) 3055, 2973, 2935, 1745, 1452, 1195, 1130, 1050, 760, 700 cm^{-1};

HRMS (ESI) m/z: $[M + H]^+$ Calcd. for $C_{16}H_{20}{}^{79}BrO_3$: 339.0590; Found 339.0595. $[M + H]^+$ Calcd. for $C_{16}H_{20}{}^{81}BrO_3$: 341.0570; Found 341.0573;

$[\alpha]_D^{25}$ = -8.34 (c = 2.0, $CHCl_3$; 100% ee).

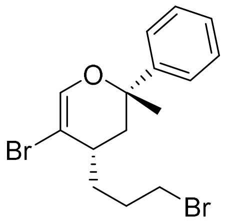

(2*S*,4*S*)-5-bromo-4-(3-bromopropyl)-2-methyl-2-phenyl-3,4-dihydro-2*H*-pyran (27)

The compound was prepared according to general procedure with (*Z*)-2,6-dibromohex-2-enal (0.27 mmol, 69.1 mg) and prop-1-en-2-ylbenzene (0.41 mmol, 48.5 mg) catalyzed by **1f** (20 mol %) over a course of 18 hours at -40 °C. The crude material was purified by column chromatography on silica gel (eluting with hexane) to give the desired product [95% yield (96.0 mg)] as a colorless oil.

The enantiomeric purity was determined by HPLC analysis (Daicel Chiralcel OD-H, hexane/2-propanol = 99:1, 1.0 mL/min, λ = 254 nm, t_R = 9.0 min (major) and t_R = 11.4 min (minor);

TLC: R_f 0.7 (ethyl acetate/hexane = 1:20);

^{1}H NMR (500 MHz, $CDCl_3$) δ 7.40 – 7.38 (m, 2H), 7.36 – 7.33 (m, 2H), 7.28 – 7.25 (m, 1H), 6.74 (d, *J* = 2.0 Hz, 1H), 3.33 (t, *J* = 6.5 Hz, 2H), 2.54 – 2.50 (m, 1H), 2.30 (d, *J* = 4.5 Hz, 1H), 2.14 (dd, *J* = 13.5, 6.0 Hz, 1H), 1.90 – 1.86 (m, 2H), 1.80 – 1.71 (m, 1H), 1.58 (s, 3H), 1.35 – 1.28 (m, 1H);

^{13}C NMR (126 MHz, $CDCl_3$) δ 145.79, 142.24, 128.46, 127.32, 124.36, 104.13, 78.14, 41.49, 34.99, 33.50, 31.75, 29.28, 24.71;

IR 3063, 2960, 2923, 1643, 1495, 1450, 1194, 1166, 766, 700 (neat) cm^{-1};

HRMS (ESI) m/z: $[M + Na]^+$ Calcd. for $C_{15}H_{19}{}^{81}Br_2O$: 396.9596; Found 396.9596;

$[\alpha]_D^{25}$ = -8.17 (c = 2.0, $CHCl_3$; 100% ee).

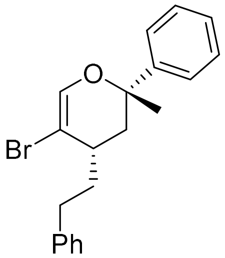

(2*S*,4*S*)-5-bromo-2-methyl-4-phenethyl-2-phenyl-3,4-dihydro-2*H*-pyran (28)

The compound was prepared according to general procedure with (*Z*)-2-bromo-5-phenylpent-2-enal (0.27 mmol, 64.6 mg) and prop-1-en-2-ylbenzene (0.41 mmol, 48.5 mg) catalyzed by **1f** (20 mol %)

over a course of 18 hours at -40 °C. The crude material was purified by column chromatography on silica gel (eluting with hexane) to give the desired product [82% yield (79.1 mg)] as a colorless oil. The enantiomeric purity was determined by HPLC analysis (Daicel Chiralcel AD-H, hexane/2-propanol = 99.5:0.5, 1.0 mL/min, λ = 254 nm, t_R = 12.9 min (major) and t_R = 19.2 min (minor);

TLC: R_f 0.7 (ethyl acetate/hexane = 1:20);

^{1}H NMR (500 MHz, $CDCl_3$) δ 7.43 (d, J = 7.5 Hz, 2H), 7.37 (t, J = 7.5 Hz, 2H), 7.30 – 7.25 (m, 3H), 7.19 – 7.14 (m, 3H), 6.76 (d, J = 1.0 Hz, 1H), 2.70 – 2.64 (m, 1H), 2.56 – 2.45 (m, 2H), 2.26 – 2.21 (m, 1H), 2.12 – 2.05 (m, 1H), 1.98 (dd, J = 14.0, 10.0 Hz, 1H), 1.59 (s, 3H), 1.53 – 1.46 (m, 1H);

^{13}C NMR (126 MHz, $CDCl_3$) δ 146.03, 142.11, 141.99, 128.52, 128.51, 128.47, 127.32, 126.01, 124.46, 104.68, 78.22, 41.57, 35.27, 34.85, 32.19, 24.79;

IR 3065, 2960, 2926, 1646, 1498, 1450, 1194, 1160, 763, 695 (neat) cm^{-1};

HRMS (ESI) m/z: $[M + Na]^+$ Calcd. for $C_{20}H_{21}{}^{79}BrNaO$: 379.0668; Found 379.0668. $[M + Na]^+$ Calcd. for $C_{20}H_{21}{}^{81}BrNaO$: 381.0647; Found 381.0643;

$[\alpha]_D^{25}$ = -0.56 (c = 0.7, $CHCl_3$; 99.2% ee).

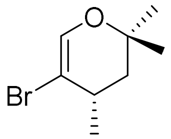

(*S*)-5-bromo-2,2,4-trimethyl-3,4-dihydro-2*H*-pyran (29)

The compound was prepared according to general procedure with (*Z*)-2-bromobut-2-enal (0.27 mmol, 61.1 mg) and 2-methylprop-1-ene (0.81 mmol, 2 M in DCM) catalyzed by **1f** (20 mol %) over a course of 3 days at -40 °C. The crude material was purified by column chromatography on silica gel (eluting with hexane) to give the desired product [63% yield (34.9 mg)] as a colorless oil. The enantiomeric purity was determined by HPLC analysis (Daicel Chiralcel AD-H, hexane/2-propanol = 100:0, 0.5 mL/min, λ = 236 nm, t_R = 9.2 min (minor) and t_R = 10.2 min (major).

TLC: R_f 0.4 (hexane);

^{1}H NMR (500 MHz, $CDCl_3$) δ 6.49 (s, 1H), 2.49 – 2.42 (m, 1H), 1.88 (dd, J = 13.5, 6.0 Hz, 1H), 1.57 – 1.55 (m, 1H), 1.26 (d, J = 19.0 Hz, 6H), 1.15 (d, J = 6.5 Hz, 3H);

^{13}C NMR (126 MHz, $CDCl_3$) δ 141.51, 105.49, 75.32, 44.02, 30.47, 29.15, 24.07, 20.27;

HRMS (ESI) m/z: $[M + H]^+$ Calcd. for $C_8H_{14}{}^{79}BrO$: 205.0223; Found 205.0222. $[M + H]^+$ Calcd. for $C_8H_{14}{}^{81}BrO$: 207.0202; Found 207.0205;

$[\alpha]_D^{25}$ = -0.65 (c = 0.5, $CHCl_3$; 99.6% ee).

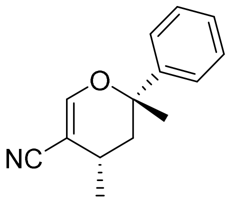

(2*S*,4*S*)-2,4-dimethyl-2-phenyl-3,4-dihydro-2*H*-pyran-5-carbonitrile (30)

The compound was prepared according to general procedure with (*E*)-2-formylbut-2-enenitrile (0.27 mmol, 25.7 mg) and prop-1-en-2-ylbenzene (0.41 mmol, 48.5 mg) catalyzed by **1f** (20 mol %) over a course of 24 hours at -40 °C. The crude material was purified by column chromatography on silica gel (eluting with ethyl acetate/hexane = 1:20) to give the both isomers [87% yield (50.1 mg), 8.9:1 dr] as a colorless oil.

The enantiomeric purity was determined by HPLC analysis (Daicel Chiralcel OD-H, hexane/2-propanol = 95:5, 1 mL/min, λ = 236 nm, t_R = 11.2 min (major) and t_R = 14.3 min (minor).

TLC: R_f 0.5 (ethyl acetate/hexane = 1:8);

^{1}H NMR (500 MHz, $CDCl_3$) δ 7.39 – 7.37 (m, 4H), 7.31 – 7.28 (m, 1H), 7.27 – 7.22 (m, 1H), 2.60 – 2.53 (m, 1H), 2.15 (dd, *J* = 14.0, 6.0 Hz, 1H), 1.60 – 1.57 (m, 1H), 1.58 (s, 3H), 1.19 (d, *J* = 7.0 Hz, 3H);

^{13}C NMR (126 MHz, $CDCl_3$) δ 155.19, 144.87, 128.58, 127.67, 127.17, 118.67, 92.56, 80.78, 42.39, 24.76, 24.60, 18.92;

HRMS (ESI) m/z: $[M + H]^+$ Calcd. for $C_{13}H_{14}NO$: 200.1071; Found 200.1070;

$[\alpha]_D^{25}$ = -50.96 (c = 0.8, $CHCl_3$; 99.0% ee).

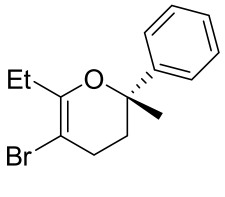

(*S*)-5-bromo-6-ethyl-2-methyl-2-phenyl-3,4-dihydro-2*H*-pyran (31)

The compound was prepared according to general procedure with 2-bromopent-1-en-3-one (0.27

mmol, 44.0 mg) and prop-1-en-2-ylbenzene (0.41 mmol, 48.5 mg) catalyzed by **1f** (20 mol %) over a course of 4 days at -40 °C. The crude material was purified by column chromatography on silica gel (eluting with ethyl acetate/hexane = 1:20) to give the desired product [76% yield (57.7 mg)] as a colorless oil.

The enantiomeric purity was determined by HPLC analysis (Daicel Chiralcel OJ-H, hexane/2-propanol = 99:1, 0.5 mL/min, λ = 236 nm, t_R = 13.1 min (major) and t_R = 18.8 min (minor).

TLC: R_f 0.4 (ethyl acetate/hexane = 1:20);

^{1}H NMR (500 MHz, $CDCl_3$) δ 7.37 – 7.35 (m, 4H), 7.24 – 7.22 (m, 1H), 3.08 – 3.02 (m, 1H), 2.91 (q, J = 10.0 Hz, 1H), 2.84 – 2.75 (m, 1H), 2.71 – 2.63 (m, 1H), 2.40 – 2.36 (m, 1H), 1.91 – 1.86 (m, 1H), 1.51 (s, 3H), 1.18 (t, J = 4.5 Hz, 3H);

^{13}C NMR (126 MHz, $CDCl_3$) δ 205.41, 147.18, 128.11, 126.76, 126.54, 77.36, 72.41, 31.27, 31.12, 27.38, 26.89, 8.36;

HRMS (ESI) m/z: $[M + H]^+$ Calcd. for $C_{14}H_{18}{}^{79}BrO$: 281.0536; Found 281.0531. $[M + H]^+$ Calcd. for $C_{14}H_{18}{}^{81}BrO$: 283.0515; Found 283.0516;

$[\alpha]_D^{25}$ = -2.66 (c = 1.0, $CHCl_3$; 90% ee).

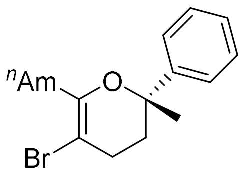

(*S*)-5-bromo-2-methyl-6-pentyl-2-phenyl-3,4-dihydro-*2H*-pyran (32)

The compound was prepared according to general procedure with 2-bromooct-1-en-3-one (0.27 mmol, 55.1 mg) and prop-1-en-2-ylbenzene (0.41 mmol, 48.5 mg) catalyzed by **1f** (20 mol %) over a course of 4 days at -40 °C. The crude material was purified by column chromatography on silica gel (eluting with diethyl ether /hexane = 1:50) to give the desired product [73% yield (63.7 mg)] as a colorless oil.

The enantiomeric purity was determined by HPLC analysis (Daicel Chiralcel OJ-H, hexane/2-propanol = 99:1, 0.5 mL/min, λ = 210 nm, t_R = 9.9 min (major) and t_R = 15.8 min (minor).

TLC: R_f 0.25 (diethyl ether /hexane = 1:50);

^{1}H NMR (500 MHz, $CDCl_3$) δ 7.36 – 7.35 (m, 4H), 7.26 – 7.22 (m, 1H), 3.06 – 3.00 (m, 1H), 2.91

(q, J = 10.0 Hz, 1H), 2.76 – 2.70 (m, 1H), 2.67 – 2.60 (m, 1H), 2.40 – 2.36 (m, 1H), 1.91 – 1.87 (m, 1H), 1.72 – 1.66 (m, 2H), 1.52 (s, 3H), 1.39 – 1.30 (m, 4H), 0.92 (t, J = 4.5 Hz, 3H);

^{13}C NMR (126 MHz, $CDCl_3$) δ 204.81, 147.28, 128.11, 126.77, 126.50, 72.51, 53.13, 37.70, 31.40, 31.30, 27.27, 26.95, 23.71, 22.65, 14.08;

HRMS (ESI) m/z: $[M + H]^+$ Calcd. for $C_{17}H_{24}{}^{79}BrO$: 323.1005; Found 323.1000. $[M + H]^+$ Calcd. for $C_{17}H_{24}{}^{81}BrO$: 325.0985; Found 325.0990;

$[\alpha]_D^{25}$ = -72.23 (c = 0.35, $CHCl_3$; 93% ee).

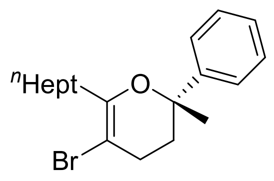

(*S*)-5-bromo-6-heptyl-2-methyl-2-phenyl-3,4-dihydro-2*H*-pyran (33)

The compound was prepared according to general procedure with 2-bromodec-1-en-3-one (0.27 mmol, 63.0 mg) and prop-1-en-2-ylbenzene (0.41 mmol, 48.5 mg) catalyzed by **1f** (20 mol %) over a course of 5 days at -40 °C. The crude material was purified by column chromatography on silica gel (eluting with diethyl ether /hexane = 1:50) to give the desired product [75% yield (71.2 mg)] as a colorless oil.

The enantiomeric purity was determined by HPLC analysis (Daicel Chiralcel OJ-H, hexane/2-propanol = 99:1, 0.5 mL/min, λ = 220 nm, t_R = 9.2 min (major) and t_R = 11.0 min (minor).

TLC: R_f 0.25 (diethyl ether /hexane = 1:50);

^{1}H NMR (500 MHz, $CDCl_3$) δ 7.37 – 7.33 (m, 4H), 7.27 – 7.22 (m, 1H), 3.06 – 3.00 (m, 1H), 2.91 (q, J = 10.0 Hz, 1H), 2.76 – 2.70 (m, 1H), 2.66 – 2.60 (m, 1H), 2.40 – 2.36 (m, 1H), 1.91 – 1.86 (m, 1H), 1.71 – 1.66 (m, 2H), 1.52 (s, 3H), 1.39 – 1.25 (m, 8H), 0.89 (t, J = 4.5 Hz, 3H);

^{13}C NMR (126 MHz, $CDCl_3$) δ 204.81, 147.28, 128.10, 126.78, 126.50, 72.52, 53.13, 37.74, 31.84, 31.29, 29.27, 29.22, 27.27, 26.95, 24.04, 22.78, 14.24;

HRMS (ESI) m/z: $[M + H]^+$ Calcd. for $C_{19}H_{28}{}^{79}BrO$: 351.1318; Found 351.1320. $[M + H]^+$ Calcd. for $C_{19}H_{28}{}^{81}BrO$: 353.1298; Found 353.1301;

$[\alpha]_D^{25}$ = -70.60 (c = 0.47, $CHCl_3$; 93% ee).

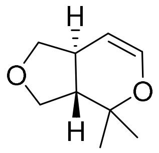

(3a*S*,7a*S*)-4,4-dimethyl-1,3a,4,7a-tetrahydro-3*H*-furo[3,4-*c*]pyran (34)

The compound was prepared according to general procedure with (*E*)-4-((3-methylbut-2-en-1-yl)oxy)but-2-enal[6] (0.27 mmol, 41.6 mg) catalyzed by **1f** (20 mol %) in toluene over a course of 30 min at -40 °C. The crude material was purified by column chromatography on silica gel (eluting with diethyl ether/pentane = 1:10) to give the desired product [87% yield (36.2 mg)] as a colorless oil.

The enantiomeric purity was determined by HPLC analysis (Daicel Chiralcel OD-H, hexane, 0.5 mL/min, λ = 210 nm, t_R = 16.7 min (minor) and t_R = 18.6 min (major).

TLC: R_f 0.2 (diethyl ether/pentane = 1:10);

^{1}H NMR (500 MHz, $CDCl_3$) δ 6.29 (dd, *J* = 6.2, 2.6 Hz, 1H), 4.78 (d, *J* = 6.2 Hz, 1H), 4.10 (t, *J* = 7.1 Hz, 1H), 3.93 (t, *J* = 7.4 Hz, 1H), 3.42 (dd, *J* = 11.6, 7.2 Hz, 1H), 3.35 (dd, *J* = 11.1, 7.0 Hz, 1H), 2.53 – 2.47 (m, 1H), 2.13 – 2.06 (m, 1H), 1.33 (s, 3H), 1.21 (s, 3H).;

^{13}C NMR (126 MHz, $CDCl_3$) δ 143.71, 97.18, 77.09, 71.52, 67.58, 52.12, 35.74, 29.24, 20.57;

HRMS (ESI) m/z: $[M + Na]^+$ Calcd. for $C_9H_{14}NaO_2$: 177.0886; Found 177.0891.

$[\alpha]_D^{25}$ = -61.92 (c = 0.30, $CHCl_3$; >99% ee).

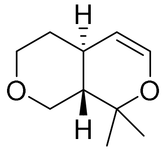

(4a*S*,8a*S*)-8,8-dimethyl-4,4a,8,8a-tetrahydro-1*H*,3*H*-pyrano[3,4-*c*]pyran (35)

The compound was prepared according to general procedure with (*E*)-5-((3-methylbut-2-en-1-yl)oxy)pent-2-enal[6] (0.27 mmol, 45.4 mg) catalyzed by **1f** (20 mol %) in toluene over a course of 30 min at -40 °C. The crude material was purified by column chromatography on silica gel (eluting with diethyl ether/pentane = 1:10) to give the desired product [85% yield (38.6 mg)] as a colorless oil.

The enantiomeric purity was determined by HPLC analysis (Daicel Chiralcel OD-H, hexane, 0.5 mL/min, λ = 210 nm, t_R = 19.5 min (major) and t_R = 21.9 min (minor).

TLC: R_f 0.2 (diethyl ether/pentane = 1:10);

^{1}H NMR (500 MHz, $CDCl_3$) δ δ 6.31 (dd, J = 6.3, 2.7 Hz, 1H), 4.54 (dt, J = 6.2, 1.7 Hz, 1H), 4.00 (dd, J = 11.4, 4.7 Hz, 1H), 3.92 (d, J = 11.4 Hz, 1H), 3.54 (dd, J = 11.5, 3.0 Hz, 1H), 3.35 – 3.30 (m, 1H), 2.37 – 2.34 (m, 1H), 1.65 – 1.53 (m, 2H), 1.48 – 1.43 (m, 1H), 1.24 (s, 3H), 1.23 (s, 3H);

^{13}C NMR (126 MHz, $CDCl_3$) δ 143.21, 100.74, 75.84, 71.69, 67.83, 37.99, 30.09, 25.54, 24.39, 23.08;

HRMS (ESI) m/z: $[M + H]^+$ Calcd. for $C_{10}H_{17}O_2$: 169.1223; Found 169.1226.

$[\alpha]_D^{25}$ = -54.79 (c = 0.30, $CHCl_3$; >99% ee).

2.5 Derivatization of 5-bromodihydropyran

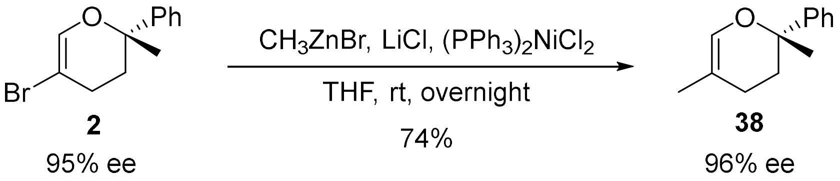

To a solution of CH_3MgBr (9 mmol) in THF (3 mL) was added $ZnBr_2$ solution (9 mmol in 5 mL of THF) dropwise at 0 °C under argon. The reaction mixture was stirred at room temperature for 1 hour. Then reaction mixture was re-cooled to 0 °C without stir for precipitation.

To a solution of **2** (66.7 mg, 0.2635 mmol), LiCl (16.79 mg, 0.396 mmol) and $(PPh_3)_2NiCl_2$ (8.5 mg, 0.013 mmol) in THF (1 mL) was added the clear solution of CH_3ZnBr (0.79 mmol) under argon[7]. The resulting mixture was stirred at room temperature overnight. Solvent was removed under reduced pressure, and the residue was purified by silica gel chromatography, affording **38** as a colorless oil (36.7 mg, 74%).

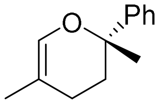

(*S*)-2,5-dimethyl-2-phenyl-3,4-dihydro-2*H*-pyran (38)

The enantiomeric purity was determined by HPLC analysis (Daicel Chiralcel IH, hexane/2-propanol = 100:0, 0.2 mL/min, λ = 254 nm, t_R = 9.5 min (minor) and t_R = 10.0 min (major).

TLC: R_f 0.5 (DCM/hexane = 1:10);

^{1}H NMR (500 MHz, $CDCl_3$) δ 7.38 – 7.35 (m, 2H), 7.34 – 7.30 (m, 2H), 7.25 – 7.21 (m, 1H), 6.28

(s, 1H), 2.22 – 2.17 (m, 1H), 1.95 – 1.89 (m, 1H), 1.84 – 1.79 (m, 1H), 1.68 – 1.61 (m, 1H), 1.48 (s, 6H);

^{13}C NMR (126 MHz, $CDCl_3$) δ 146.23, 137.06, 128.27, 126.65, 124.90, 108.12, 76.64, 33.16, 29.38, 23.68, 18.44;

IR (neat) 3068, 2977, 2921, 1674, 1447, 1274, 1146, 1030, 766, 703 cm^{-1};

HRMS (ESI) m/z: $[M + H]^+$ Calcd. for $C_{13}H_{17}O$: 211.1093; Found 211.1095;

$[\alpha]_D^{25}$ = -19.68 (c = 0.5, $CHCl_3$; 96% ee).

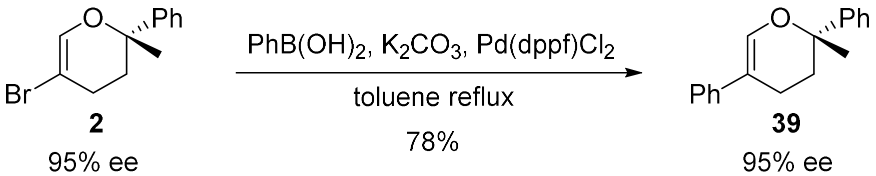

To a solution of **2** (38.5 mg, 0.152 mmol), $PhB(OH)_2$ (22.25 mg, 0.183 mmol), and K_2CO_3 (42 mg, 0.304 mmol) in toluene (2 mL) was added $Pd(dppf)Cl_2$ (0.5 mol %) under argon, then the solution was refluxed for 6 h. Then H_2O (1 mL) and EtOH (1 mL) were added, and the resulting mixture was refluxed for another 6 h[8]. Solvent was removed under reduced pressure, and the residue was purified by silica gel chromatography, affording **39** (29.7 mg, 78%) as a colorless oil.

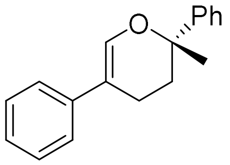

(*S*)-2-methyl-2,5-diphenyl-3,4-dihydro-2*H*-pyran (39)

The enantiomeric purity was determined by HPLC analysis (Daicel Chiralcel OJ-H, hexane/2-propanol = 95:5, 1 mL/min, λ = 254 nm, t_R = 9.0 min (minor) and t_R = 14.8 min (major).

TLC: R_f 0.2 (DCM/hexane = 1:2);

^{1}H NMR (500 MHz, $CDCl_3$) δ 7.41 – 7.39 (m, 2H), 7.34 – 7.31 (m, 2H), 7.27 – 7.22 (m, 5H), 7.15 – 7.11 (m, 1H), 7.06 (s, 1H), 2.37 – 2.31 (m, 2H), 2.12 – 2.04 (m, 2H), 1.58 (s, 3H);

^{13}C NMR (126 MHz, $CDCl_3$) δ 145.66, 140.78, 139.44, 128.50, 128.43, 126.91, 125.82, 124.74, 124.06, 112.50, 77.85, 33.32, 29.16, 20.80;

IR (neat) 3057, 2977, 2921, 1637, 1598, 1493, 1453, 1197, 1158, 754, 698 cm^{-1};

HRMS (ESI) m/z: $[M + Na]^+$ Calcd. for $C_{18}H_{18}NaO$: 273.1250; Found 273.1252;

$[\alpha]_D^{25}$ = -92.1 (c = 0.3, $CHCl_3$; 96% ee).

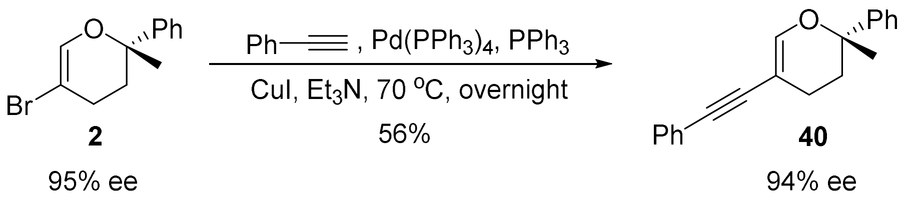

To a solution of **2** (52.6 mg, 0.208 mmol), $Pd(PPh_3)_4$ (6 mg, 2.5 mmol %), PPh_3 (1.3 mg, 2.5 mmol %), CuI (0.5 mg, 1.25 mmol %) in triethyl amine (2 mL) was added phenylacetylene (31.9 mg, 0.312 mmol)[9]. The resulting mixture was stirred at 70 °C overnight. Solvent was removed under reduced pressure, and the residue was purified by silica gel chromatography, affording **40** as a colorless oil (32 mg, 56%).

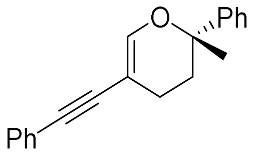

(*S*)-2-methyl-2-phenyl-5-(phenylethynyl)-3,4-dihydro-2*H*-pyran (40)

The enantiomeric purity was determined by HPLC analysis (Daicel Chiralcel OD-H, hexane/2-propanol = 99.5:0.5, 1 mL/min, λ = 236 nm, t_R = 9.7 min (major) and t_R = 13.1 min (minor).

TLC: R_f 0.2 (DCM/hexane = 1:10);

^{1}H NMR (500 MHz, $CDCl_3$) δ 7.39 – 7.32 (m, 6H), 7.27 – 7.21 (m, 4H), 7.03 (s, 1H), 2.25 – 2.15 (m, 2H), 2.00 – 1.93 (m, 2H), 1.56 (s, 3H);

^{13}C NMR (126 MHz, $CDCl_3$) δ 149.12, 145.05, 131.20, 128.54, 128.32, 127.50, 127.12, 124.60, 124.11, 96.58, 88.86, 87.92, 78.65, 32.79, 29.06, 22.61;

IR (neat) 3065, 2986, 2926, 2208, 1635, 1493, 1265, 1194, 1169, 1067, 757, 698 cm^{-1};

HRMS (ESI) m/z: $[M + H]^+$ Calcd. for $C_{20}H_{19}O$: 275.1431; Found 275.1434;

$[\alpha]_D^{25}$ = -59.99 (c = 0.2, $CHCl_3$; 94% ee).

2.6 Synthesis of the (+)-*centrolobine*

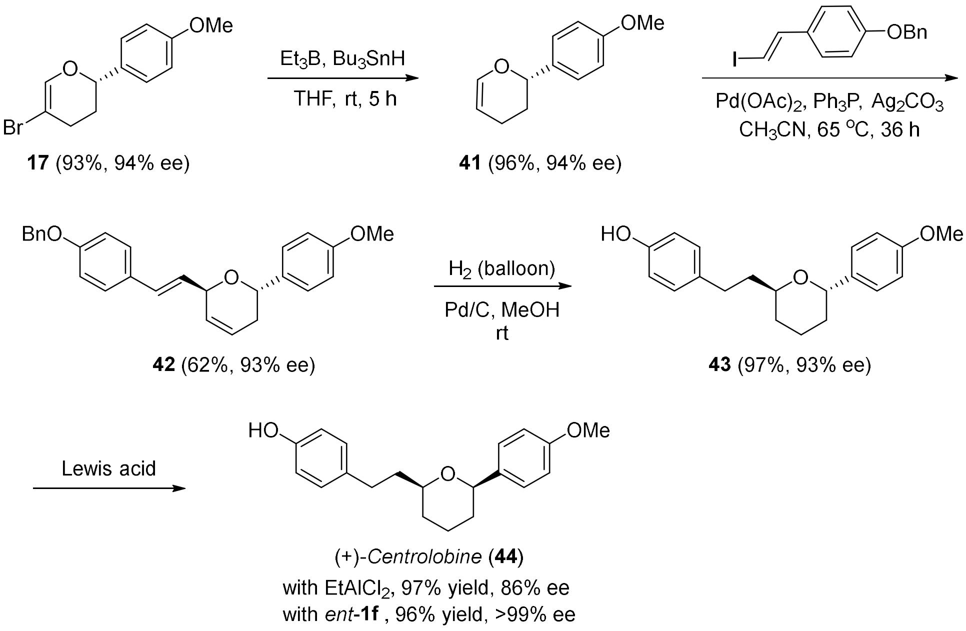

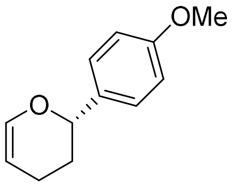

(*S*)-2-(4-methoxyphenyl)-3,4-dihydro-2*H*-pyran (41)

To a solution of compound **17** (99.6 mg, 0.37 mmol) in 2 mL of dry toluene at room temperature was added triethylborane (74 μL, 1.0 M solution in THF, 0.074 mmol) and tributyltin hydride (118.9 μL, 0.45 mmol)[10]. The reaction mixture was stirred at room temperature for 5 h and then was quenched with 10 mL of saturated NH_4Cl solution. The mixture was extracted with dichloromethane (3 × 20 mL). The combined organic phase was dried over anhydrous Na_2SO_4, and concentrated in vacuo. The residue was purified by column chromatography on the mixture of silica gel and K_2CO_3 (10/1 W/W) to give the desired product compound **41** [96% yield (67.6 mg)] as a colorless oil.

The enantiomeric purity was determined by HPLC analysis (Daicel Chiralcel OD-H, hexane/2-propanol = 95: 5, 0.5 mL/min, λ = 254 nm, t_R = 9.8 min (minor) and t_R = 10.7 min (major).

TLC: R_f 0.3 (DCM/hexane = 1:3);

^1H NMR (500 MHz, $CDCl_3$) δ 7.28 (d, J = 8.5 Hz, 2H), 6.88 (d, J = 8.5 Hz, 2H), 6.51 (d, J = 6.5 Hz, 1H), 4.77 – 4.74 (m, 2H), 3.78 (s, 3H), 2.26 – 2.18 (m, 1H), 2.05 – 1.89 (m, 3H);

^{13}C NMR (126 MHz, $CDCl_3$) δ 159.19, 144.38, 134.25, 127.32, 113.85, 100.62, 76.86, 55.33, 30.26, 20.54;

$[\alpha]_D^{25}$ = 24.69 (c = 2.0, $CHCl_3$; 94% ee).

The debromination reaction can also be achieved with tris(trimethylsilyl)silane: Under dry air conditions, to a solution of compound **17** (60 mg, 0.22 mmol) in 2 mL of dry toluene at 0 °C was added triethylborane (330 µL, 1.0 M solution in THF, 0.33 mmol) and tris(trimethylsilyl)silane (80 µL, 0.26 mmol). Then the reaction mixture was stirred at room temperature for 4 h and the solvent was removed under reduced pressure quickly. The residue was purified by column chromatography on the mixture of silica gel and K_2CO_3 (10/1 W/W) to give compound **37** in 94% yield (39.3 mg)], but the enantiomeric excess decreased to 81%.

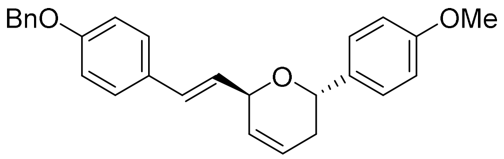

(2*S*,6*S*)-6-((*E*)-4-(benzyloxy)styryl)-2-(4-methoxyphenyl)-3,6-dihydro-2*H*-pyran (42)

The mixture of compound **41** (95.1 mg, 0.5 mmol), freshly prepared (*E*)-1-(benzyloxy)-4-(2-iodovinyl)benzene[11] (218.5 mg, 0.65 mmol), $Pd(OAc)_2$ (3.9 mg, 3.5 mol %), Ph_3P (11.8 mg, 9 mol %), and Ag_2CO_3 (275.8 mg, 2 equiv.) in anhydrous CH_3CN (5 mL) under argon was stirred at 70°C for 36 h[12]. Then the mixture was filtered and the filtrate was concentrated under reduced pressure. The crude material was purified by column chromatography on silica gel (eluting with ethyl acetate/hexane = 1:15) to give the compound **42** [62% yield (123.5 mg)] as a white solid (mp: 103-105 °C).

The enantiomeric purity was determined by HPLC analysis (Daicel Chiralcel AD-H, hexane/2-propanol = 9:1, 1.0 mL/min, λ = 254 nm, t_R = 20.7 min (major) and t_R = 24.7 min (minor).

TLC: R_f 0.20 (ethyl acetate/hexane = 1:15);

^1H NMR (500 MHz, $CDCl_3$) δ 7.43 – 7.30 (m, 9H), 6.90 (dd, J = 20.8, 8.6 Hz, 4H), 6.52 (d, J = 16.0 Hz, 1H), 6.24 (dd, J = 16.0, 5.6 Hz, 1H), 6.06 – 6.03 (m, 1H), 5.90 (d, J = 10.3 Hz, 1H), 5.05 (s, 2H), 4.96 (s, 1H), 4.69 (dd, J = 10.0, 3.4 Hz, 1H), 3.79 (s, 3H), 2.41 – 2.35 (m, 1H), 2.25 – 2.20

(m, 1H);

^{13}C NMR (126 MHz, $CDCl_3$) δ 159.03, 158.54, 136.92, 134.72, 131.82, 129.80, 128.64, 128.03, 127.83, 127.81, 127.62, 127.50, 126.61, 125.72, 114.96, 113.81, 77.09, 73.72, 70.04, 69.60, 55.33, 32.47;

HRMS (ESI) m/z: $[M + Na]^+$ Calcd. for $C_{27}H_{26}NaO_3$: 421.1774; Found 421.1775.

$[\alpha]_D^{25}$ = -61.92 (c = 0.25, $CHCl_3$; 93% ee).

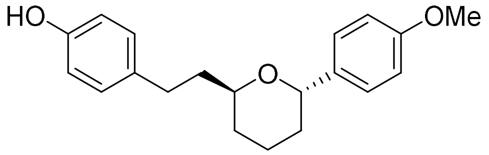

4-(2-((2*S*,6*S*)-6-(4-methoxyphenyl)tetrahydro-2*H*-pyran-2-yl)ethyl)phenol (43)

To the solution of **42** (40 mg, 0.1 mmol) in methanol (15 mL) was added and Pd/C (10%) (8 mg) and the suspension was stirred under H_2 atmosphere at room temperature overnight. The reaction mixture was filtered and concentrated under reduced pressure. The crude material was purified by short column chromatography on silica gel (eluting with ethyl acetate/hexane = 1:5) to give the compound **42** [97% yield (30.3 mg)] as a white solid.

The enantiomeric purity was determined by HPLC analysis (Daicel Chiralcel IA, hexane/2-propanol = 10:1, 1.0 mL/min, λ = 236 nm, t_R = 14.0 min (minor) and t_R = 17.3 min (major).

TLC: R_f 0.20 (ethyl acetate/hexane = 1:5);

^{1}H NMR (500 MHz, $CDCl_3$) δ 7.32 (d, *J* = 8.7 Hz, 2H), 7.03 (d, *J* = 8.4 Hz, 2H), 6.89 (d, *J* = 8.7 Hz, 2H), 6.71 (d, *J* = 8.5 Hz, 2H), 5.56 (s, 1H), 4.83 – 4.81 (m, 1H), 3.80 (s, 3H), 3.79 – 3.74 (m, 1H), 2.78 – 2.68 (m, 1H), 2.57 – 2.51 (m, 1H), 2.11 – 2.04 (m, 1H), 1.97 – 1.84 (m, 2H), 1.81 – 1.61 (m, 4H), 1.51 – 1.39 (m, 1H);

^{13}C NMR (126 MHz, $CDCl_3$) δ 158.70, 153.87, 134.37, 134.30, 129.55, 127.96, 115.29, 113.87, 72.06, 71.49, 55.41, 35.35, 31.37, 30.12, 30.06, 19.15;

The physical and spectral data were identical to the previously reported for this compound[13].

$[\alpha]_D^{25}$ = -7.49 (c = 0.7, $CHCl_3$; 93% ee).

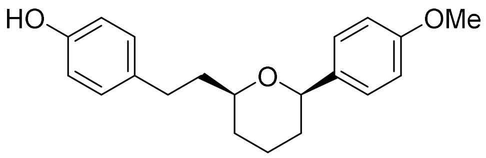

4-(2-((2*S*,6*R*)-6-(4-methoxyphenyl)tetrahydro-2*H*-pyran-2-yl)ethyl)phenol (44)

To a solution of **43** (30 mg) in CH_3CN (2 mL) at 0 °C was added $EtAlCl_2$ (15μL, 25 wt% in ^{n}hexane). The mixture was stirred at the same temperature for 10 min, and it was fully converted into **44**. Then the reaction was quenched MeOH (30 μL) and the mixture was concentrated under reduced pressure. The crude material was purified by short column chromatography on silica gel (eluting with ethyl acetate/hexane = 1:5) to give the compound **44** [97% yield (29.1 mg), 86% ee] as a white solid.

To a solution of *ent*-**1f** (0.054 mmol, theoretical) in CH_2Cl_2 (1 mL) at -20 °C was added compound **43** (20 mg in 1 mL of CH_2Cl_2). The mixture was stirred at the same temperature for 48 h, and it was fully converted into **44**. Then the reaction was quenched Et_3N (30 μL) and the mixture was concentrated under reduced pressure. The crude material was purified by short column chromatography on silica gel (eluting with ethyl acetate/hexane = 1:5) to give the compound **44** [96% yield (19.2 mg), 99.8% ee] as a white solid.

The enantiomeric purity was determined by HPLC analysis (Daicel Chiralcel IA, hexane/2-propanol = 10:1, 1.0 mL/min, λ = 225 nm, t_R = 14.0 min (minor) and t_R = 18.7 min (major).

TLC: R_f 0.23 (ethyl acetate/hexane = 1:5);

^{1}H NMR (500 MHz, $CDCl_3$) δ 7.31 (d, *J* = 8.7 Hz, 2H), 7.02 (d, *J* = 8.3 Hz, 2H), 6.88 (d, *J* = 8.7 Hz, 2H), 6.70 (d, *J* = 8.2 Hz, 2H), 5.17 (d, *J* = 35.7 Hz, 1H), 4.30 (dd, *J* = 11.1, 2.2 Hz, 1H), 3.79 (s, 3H), 3.46 – 3.42 (m, 1H), 2.74 – 2.61 (m, 2H), 1.94 – 1.80 (m, 3H), 1.75 – 1.69 (m, 1H), 1.66 - 1.58 (m, 2H), 1.56 – 1.47 (m, 1H), 1.37 – 1.28 (m, 1H).

^{13}C NMR (126 MHz, $CDCl_3$) δ 158.81, 153.66, 135.89, 134.61, 129.65, 127.28, 115.22, 113.74, 79.27, 77.38, 77.16, 55.42, 38.39, 33.32, 31.34, 30.86, 24.15.

The physical and spectral data were identical to the previously reported for this compound[14].

$[\alpha]_D^{25}$ = +42.36 (c = 0.3, $CHCl_3$; 99.8% ee).

3. Supplementary Discussion

Computational Methods

All DFT calculations were carried out using Gaussian 09 program[15]. All the geometry optimizations and frequency calculations in this paper were performed with B3LYP[16,17] functional in implicit toluene, at 6-31G(d) basis set by using the Solvation Model based on Density[18] (SMD) with keyword in the Gaussian code route section "SCRF = (SMD, Solvent = toluene)". The vibrational frequencies were computed at the same level of theory as for the geometry optimizations to confirm whether each optimized structure is an energy minimum or a transition state, and to evaluate the zero-point vibrational energy (ZPVE) and thermal corrections. Single-point energy calculations were also performed on an optimized geometry using a higher level basis set 6-311+G(d,p). The Gibbs free energies presented in this paper are the M06[19] calculated single-point energy in toluene solvent with B3LYP calculated thermodynamic corrections in toluene solvent.

Supplementary Table 1. B3LYP and M06 calculated absolute energies, enthalpies, and free energies of all structures.

Geometry	$E_{(elec-B3LYP)}$[1]	$H_{(corr-B3LYP)}$[2]	$G_{(corr-B3LYP)}$[3]	$E_{(solv-M06)}$[4]	IF[5]
cat. **1f**	-1278.528510	0.568713	0.482518	-1277.878102	
**SM1**	-2762.715121	0.058333	0.021751	-2765.260299	
**Int1**	-4041.254721	0.629382	0.527441	-4043.161317	
**Int2**	-4390.239505	0.804466	0.682245	-4391.962413	
**SM2**	-348.966359	0.171251	0.128869	-348.771716	
**TS_1**	-4390.223500	0.801905	0.678729	-4391.942786	215.35*i*
**TS_2**	-4390.226595	0.803978	0.687992	-4391.958695	124.22*i*
**TS_3**	-4390.221380	0.804939	0.689580	-4391.953987	243.89*i*
**TS_4**	-4390.227940	0.804153	0.687167	-4391.955408	212.87*i*
**TS_5**	-4390.230648	0.803535	0.685783	-4391.956628	236.48*i*
**2**	-3111.719757	0.235705	0.182289	-3114.078601	

***ent*-2**	-3111.719757	0.235705	0.182289	-3114.078601	
***trans*-3**	-3111.705293	0.233938	0.178252	-3114.072076	
***cis*-3**	-3111.700094	0.234190	0.179301	-3114.067404	
cat. **1a**	-1202.314842	0.551144	0.468728	-1201.703714	
**Int3**	-3965.039081	0.614810	0.511612	-3966.984455	
**Int4**	-4314.024993	0.788907	0.666883	-4315.784673	
**TS_7**	-4314.007151	0.789914	0.672462	-4315.774357	425.80*i*
**TS_8**	-4313.998936	0.790671	0.671779	-4315.769160	228.66*i*
**TS_9**	-4314.012065	0.789389	0.670832	-4315.762007	216.25*i*
**TS_{10}**	-4314.014502	0.789465	0.669083	-4315.779111	229.94*i*

[1]The electronic energy calculated by B3LYP in toluene solvent. [2]The thermal correction to enthalpy calculated by B3LYP in toluene solvent. [3]The thermal correction to Gibbs free energy calculated by B3LYP in toluene solvent. [4]The electronic energy calculated by M06 in toluene solvent. [5]The B3LYP calculated imaginary frequencies for the transition states. Source data are provided as a Source Data file.

^{1}H NMR, ^{13}C NMR, ^{19}F NMR and NOE Spectra

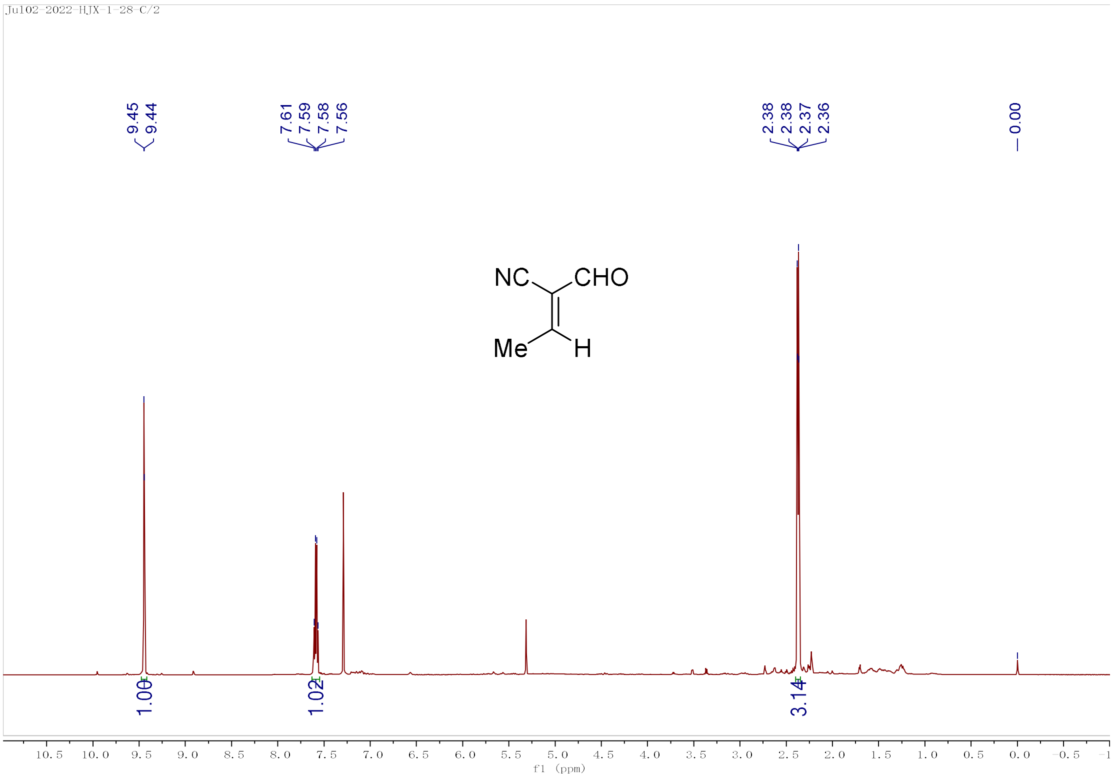

Supplementary Fig. 5. ^{1}H NMR spectrum of (*E*)-2-formylbut-2-enenitrile ($CDCl_3$, 500 MHz)

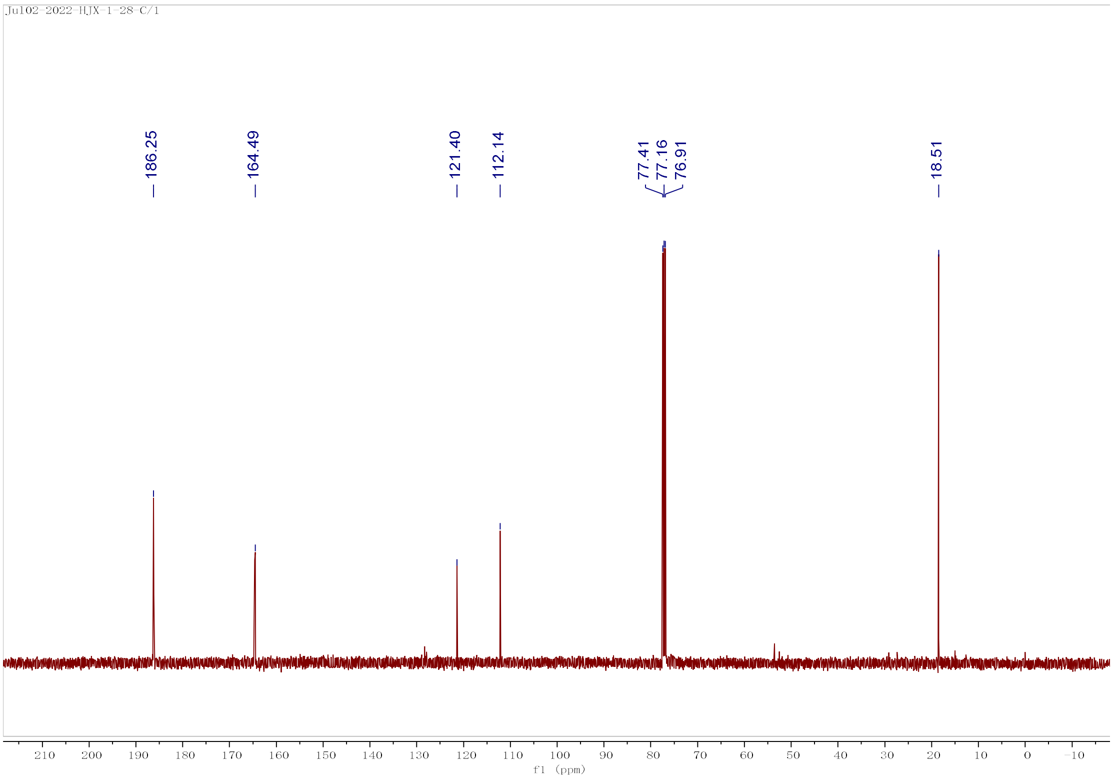

Supplementary Fig. 6. ^{13}C NMR spectrum of (*E*)-2-formylbut-2-enenitrile ($CDCl_3$, 126 MHz)

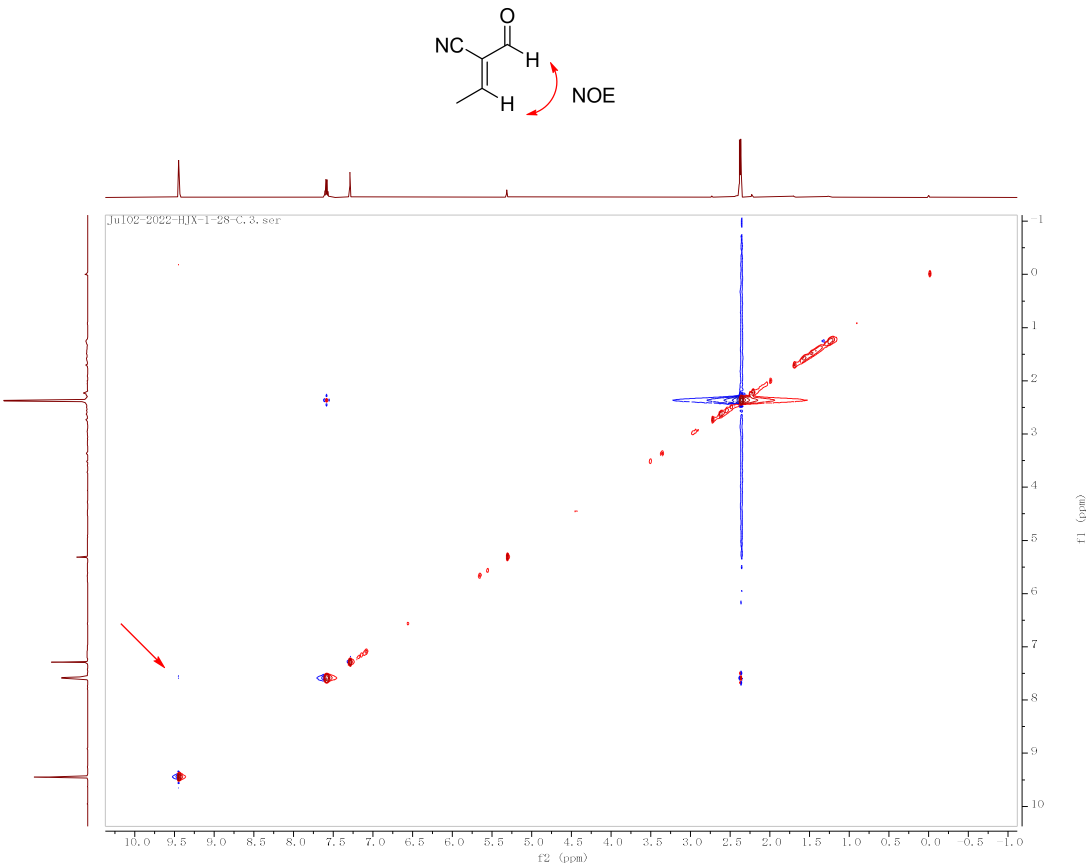

Supplementary Fig. 7. NOE spectrum of (*E*)-2-formylbut-2-enenitrile ($CDCl_3$, 500 MHz)

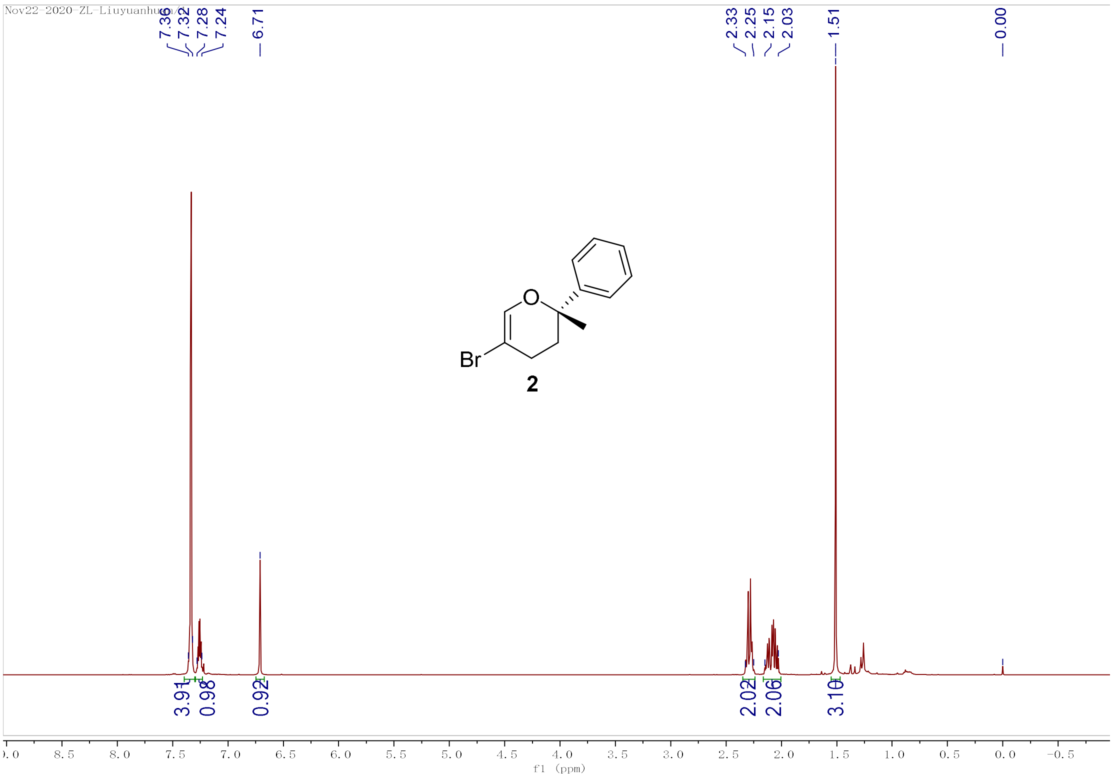

Supplementary Fig. 8. ^{1}H NMR spectrum of **2** ($CDCl_3$, 500 MHz)

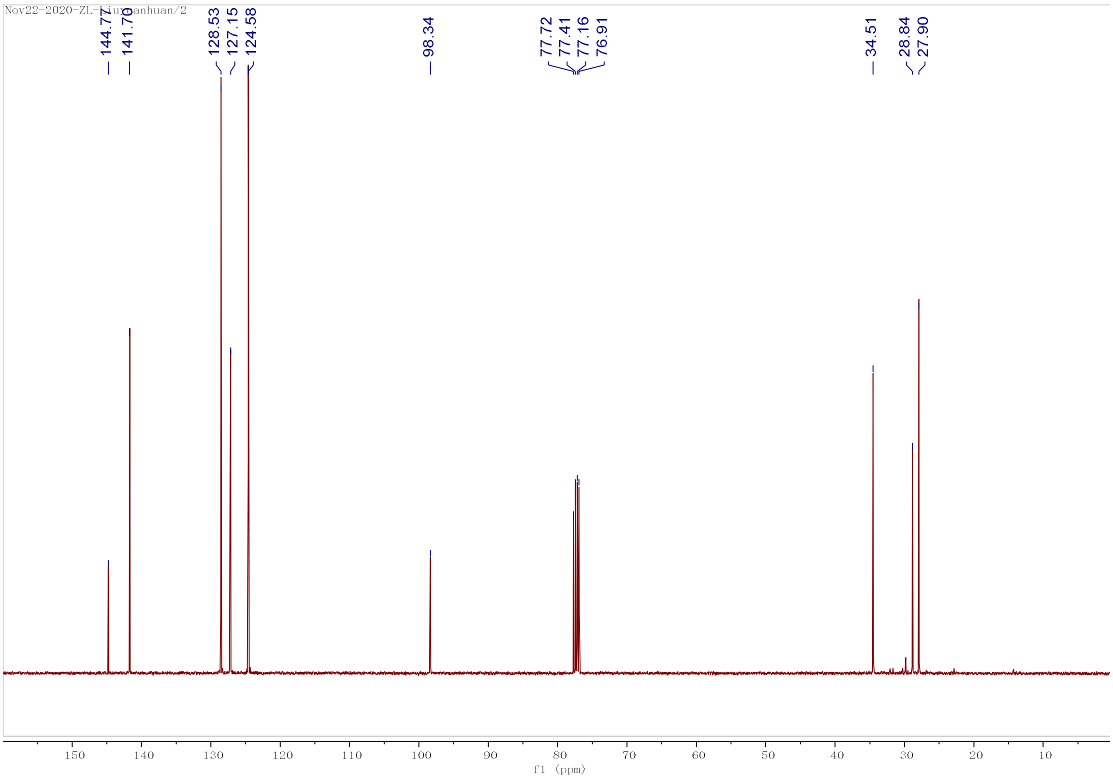

Supplementary Fig. 9. ^{13}C NMR spectrum of **2** ($CDCl_3$, 126 MHz)

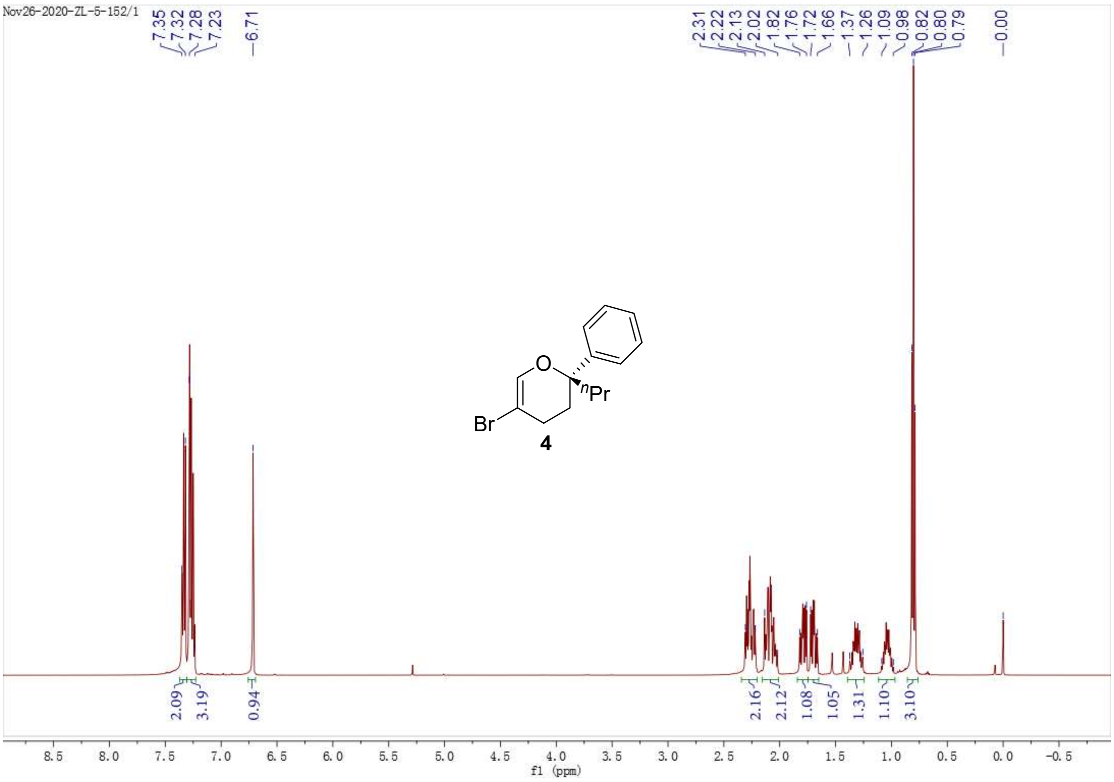

Supplementary Fig. 10. ^{1}H NMR spectrum of **4** ($CDCl_3$, 500 MHz)

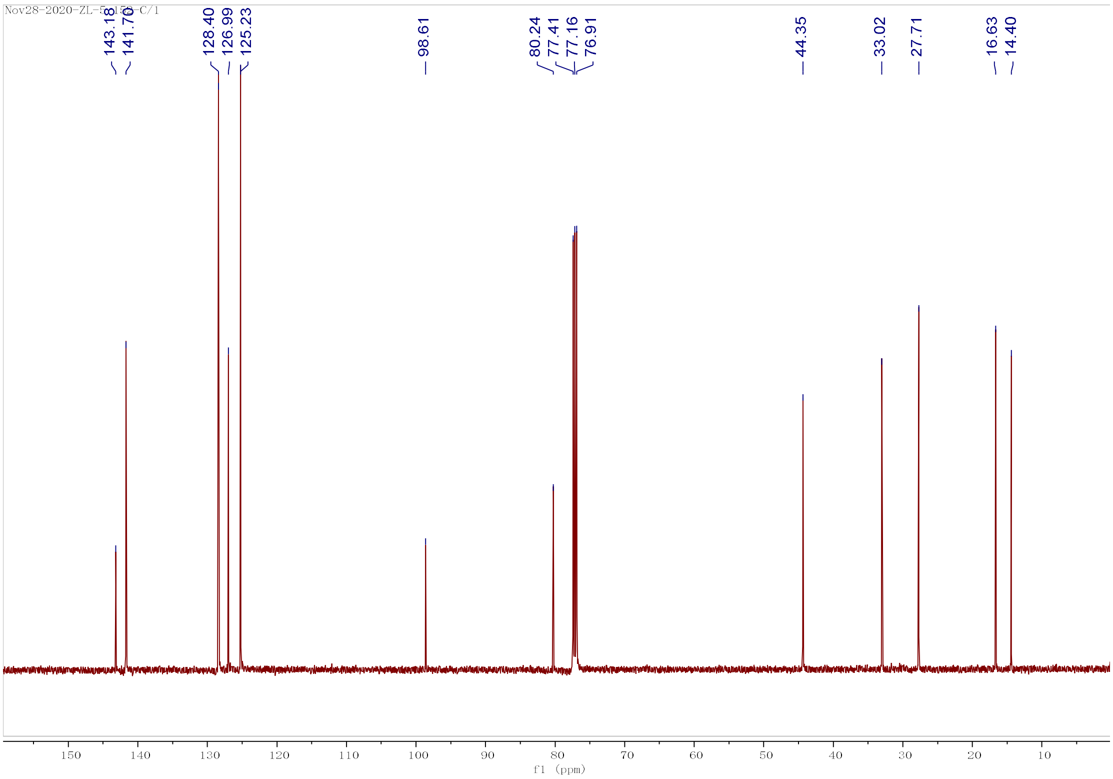

Supplementary Fig. 11. ^{13}C NMR spectrum of **4** ($CDCl_3$, 126 MHz)

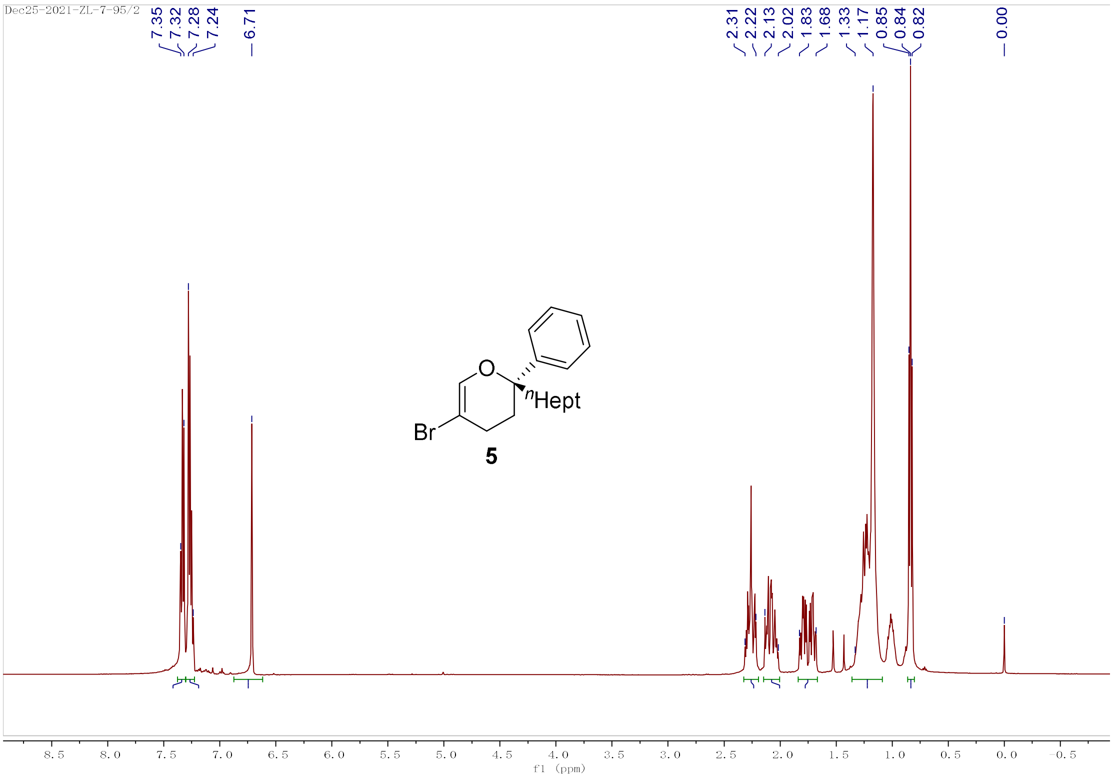

Supplementary Fig. 12. ^{1}H NMR spectrum of **5** ($CDCl_3$, 500 MHz)

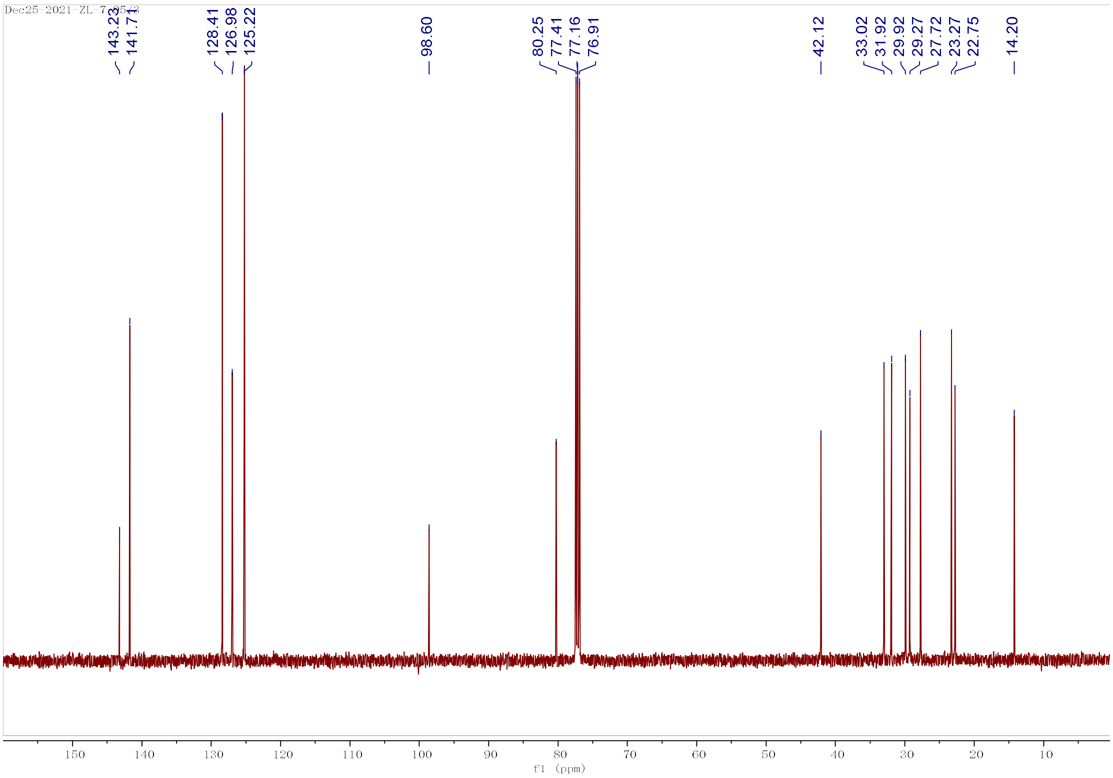

Supplementary Fig. 13. ^{13}C NMR spectrum of **5** ($CDCl_3$, 126 MHz)

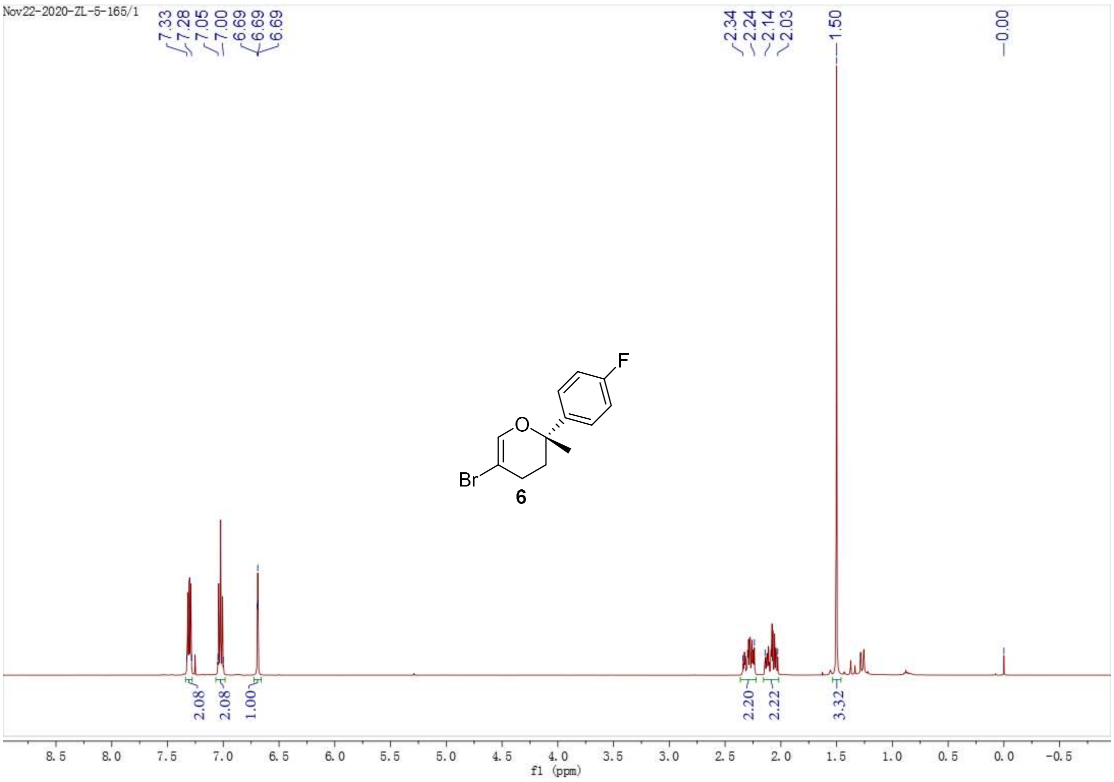

Supplementary Fig. 14. ^{1}H NMR spectrum of **6** ($CDCl_3$, 500 MHz)

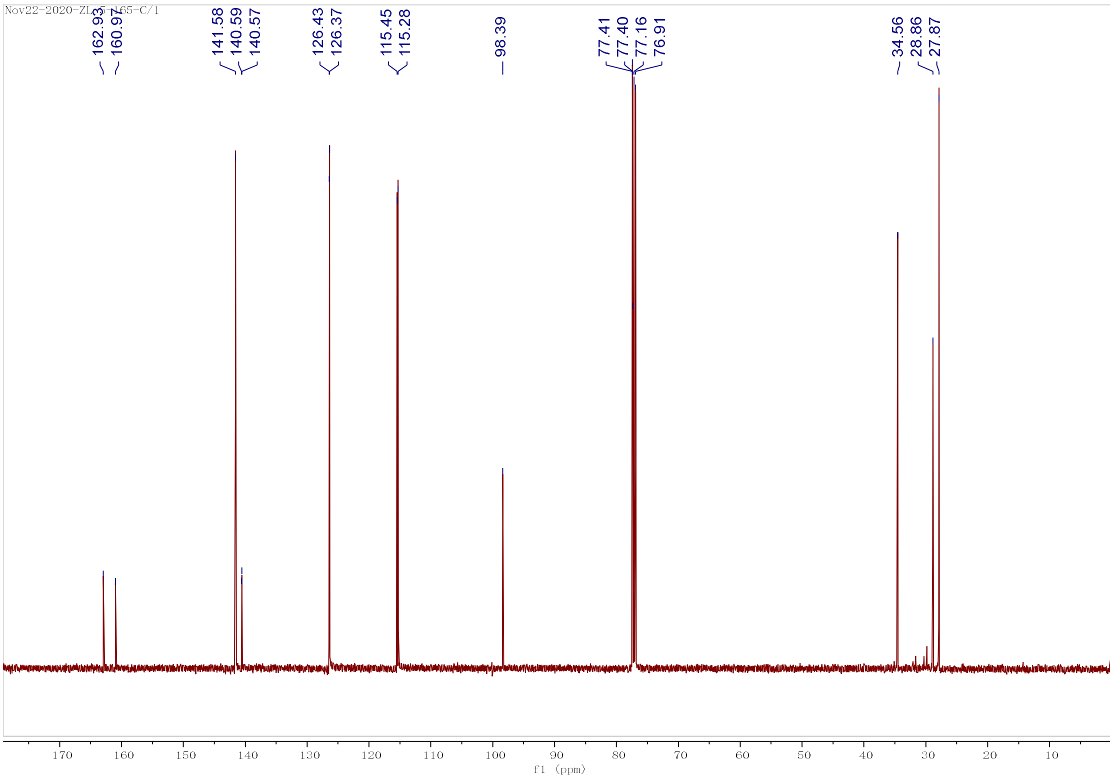

Supplementary Fig. 15. ^{13}C NMR spectrum of **6** ($CDCl_3$, 126 MHz)

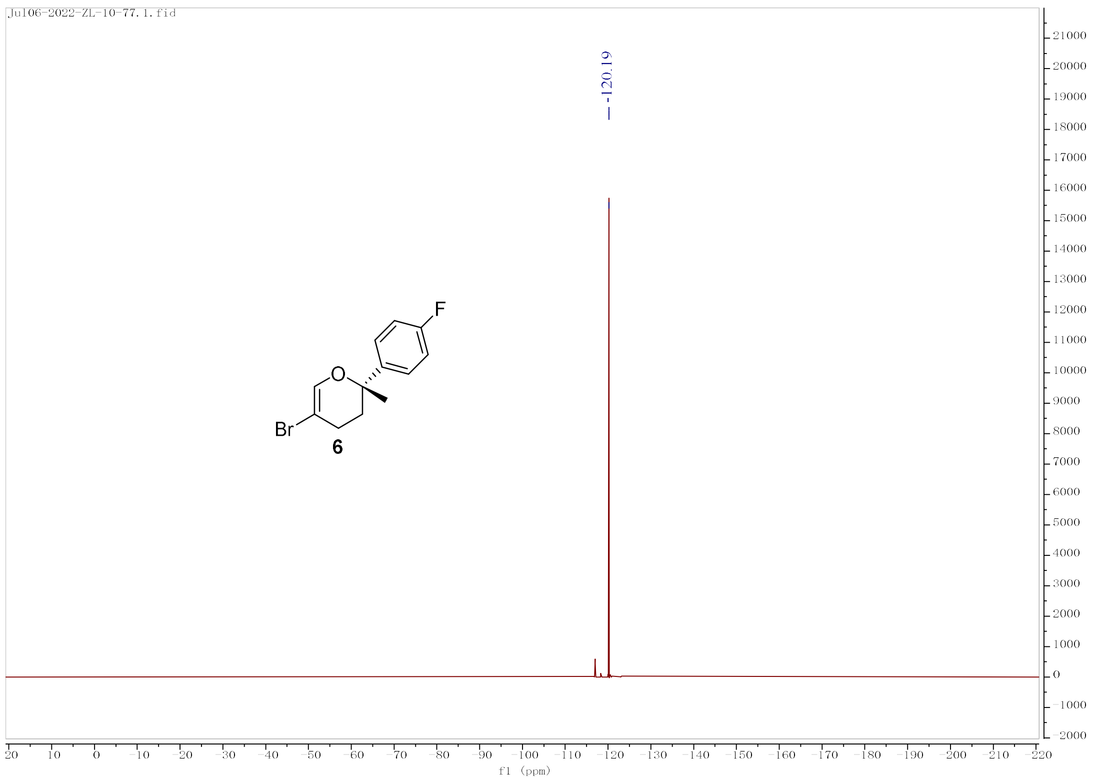

Supplementary Fig. 16. ^{19}F NMR spectrum of **6** in $CDCl_3$

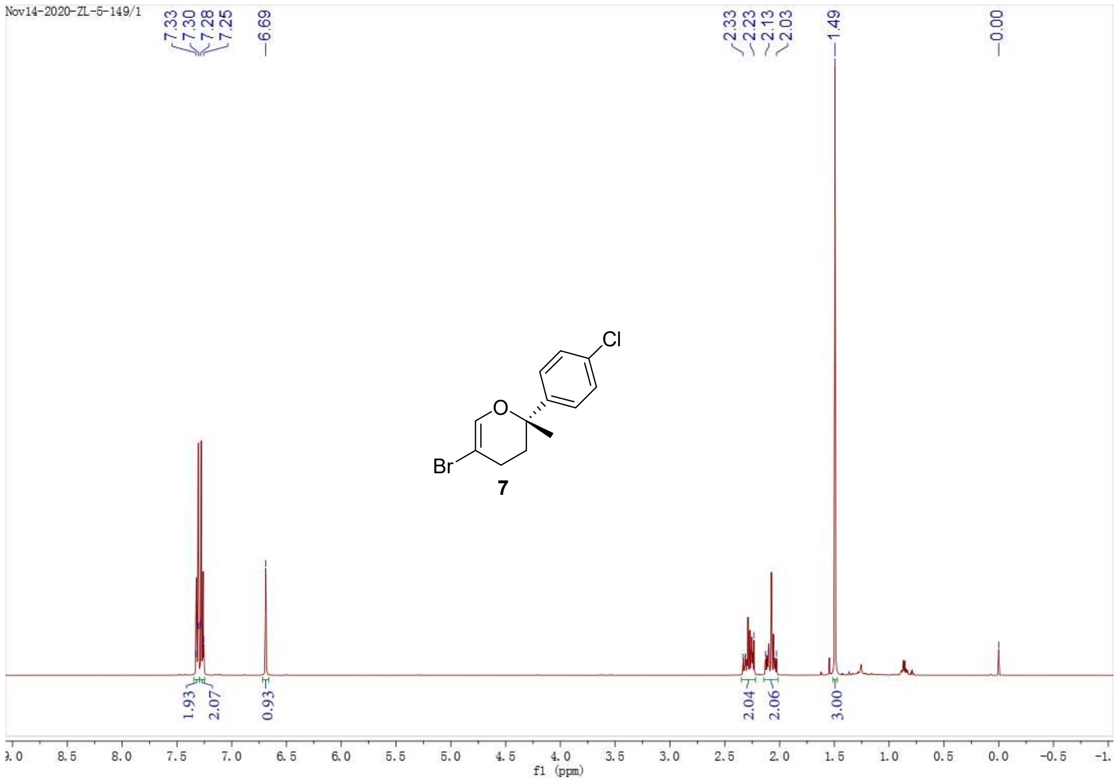

Supplementary Fig. 17. ^{1}H NMR spectrum of **7** ($CDCl_3$, 500 MHz)

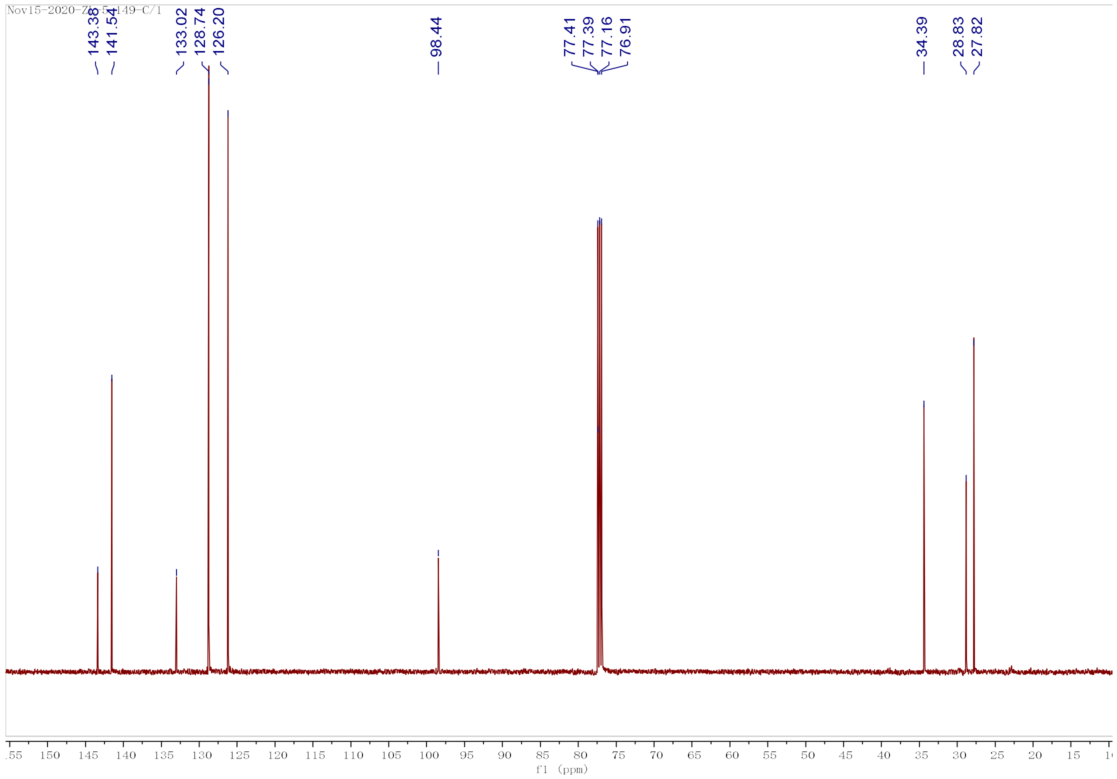

Supplementary Fig. 18. ^{13}C NMR spectrum of **7** ($CDCl_3$, 126 MHz)

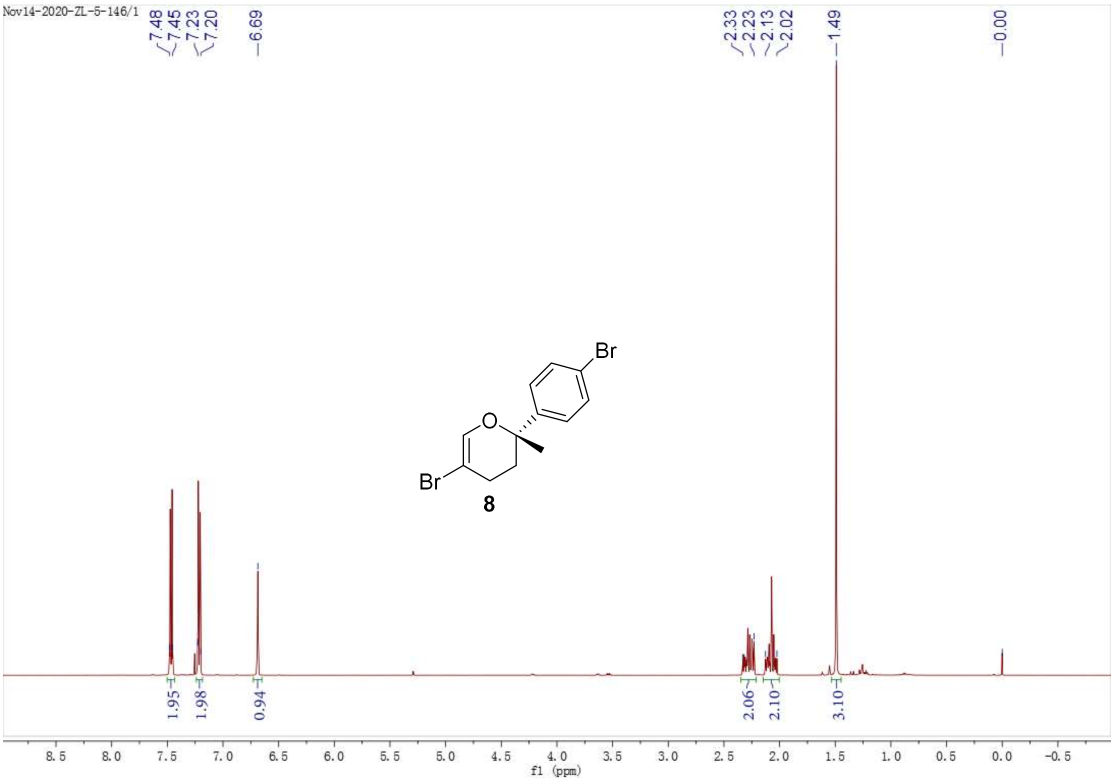

Supplementary Fig. 19. ^{1}H NMR spectrum of **8** ($CDCl_3$, 500 MHz)

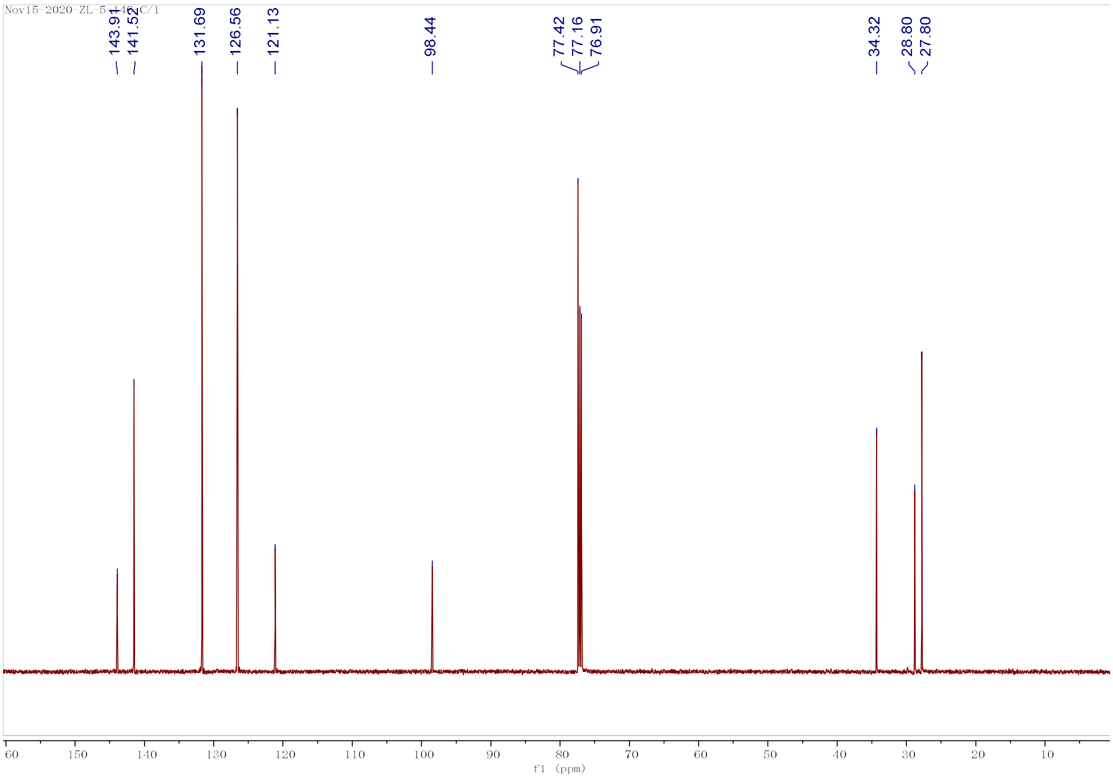

Supplementary Fig. 20. ^{13}C NMR spectrum of **8** ($CDCl_3$, 126 MHz)

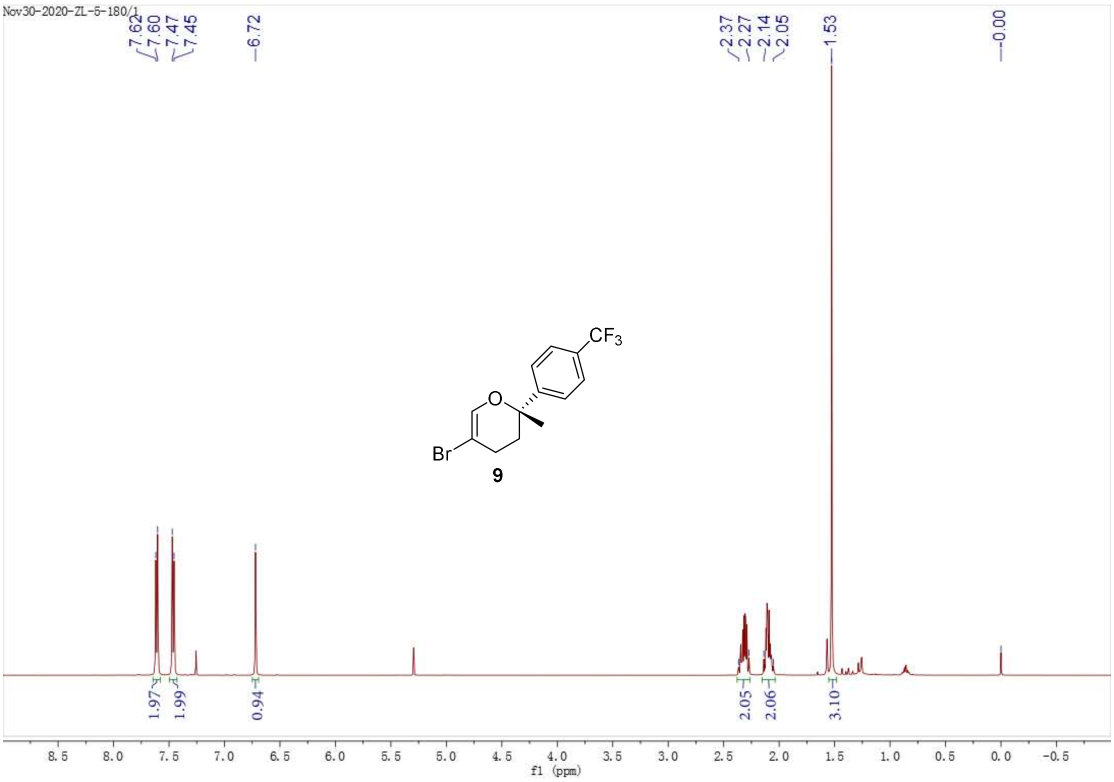

Supplementary Fig. 21. ^{1}H NMR spectrum of **9** ($CDCl_3$, 500 MHz)

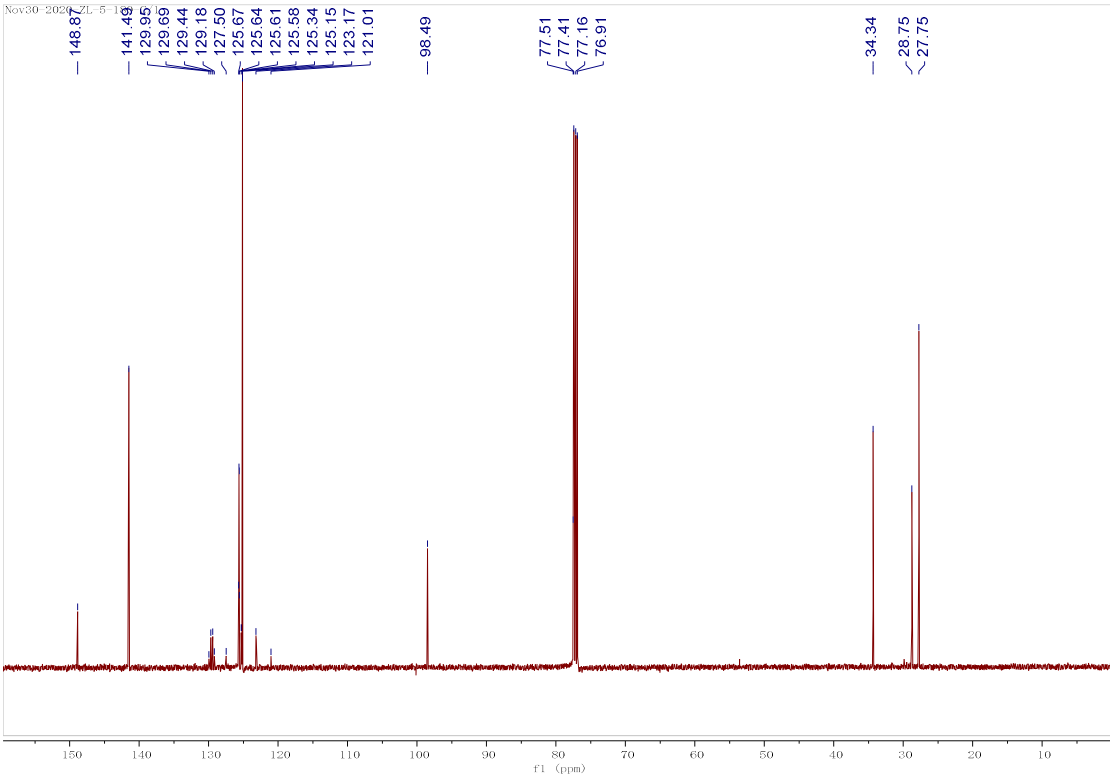

Supplementary Fig. 22. ^{13}C NMR spectrum of **9** ($CDCl_3$, 126 MHz)

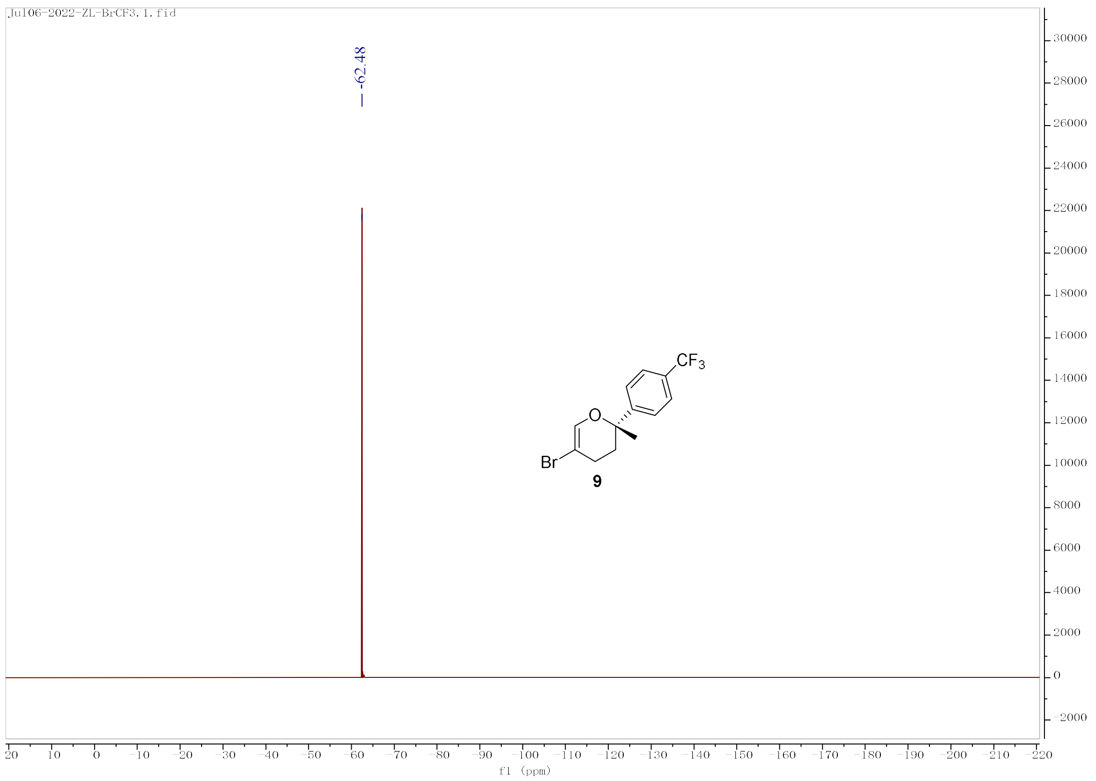

Supplementary Fig. 23. ^{19}F NMR spectrum of **9** in $CDCl_3$

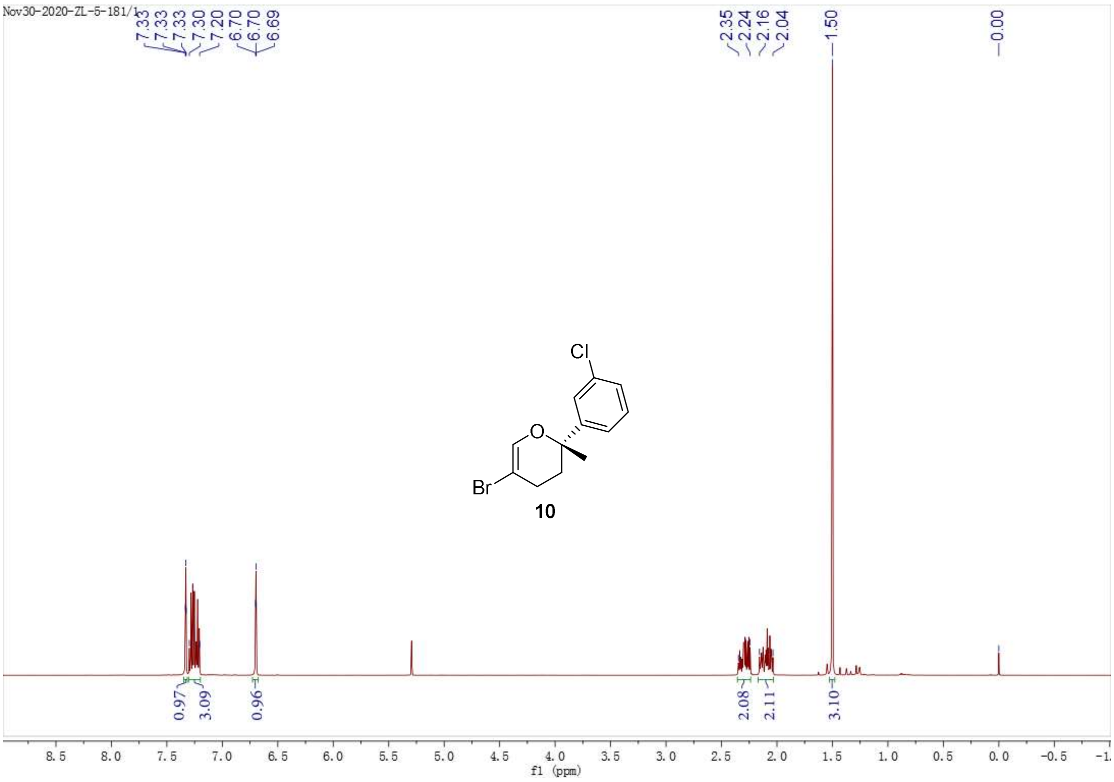

Supplementary Fig. 24. ^{1}H NMR spectrum of **10** ($CDCl_3$, 500 MHz)

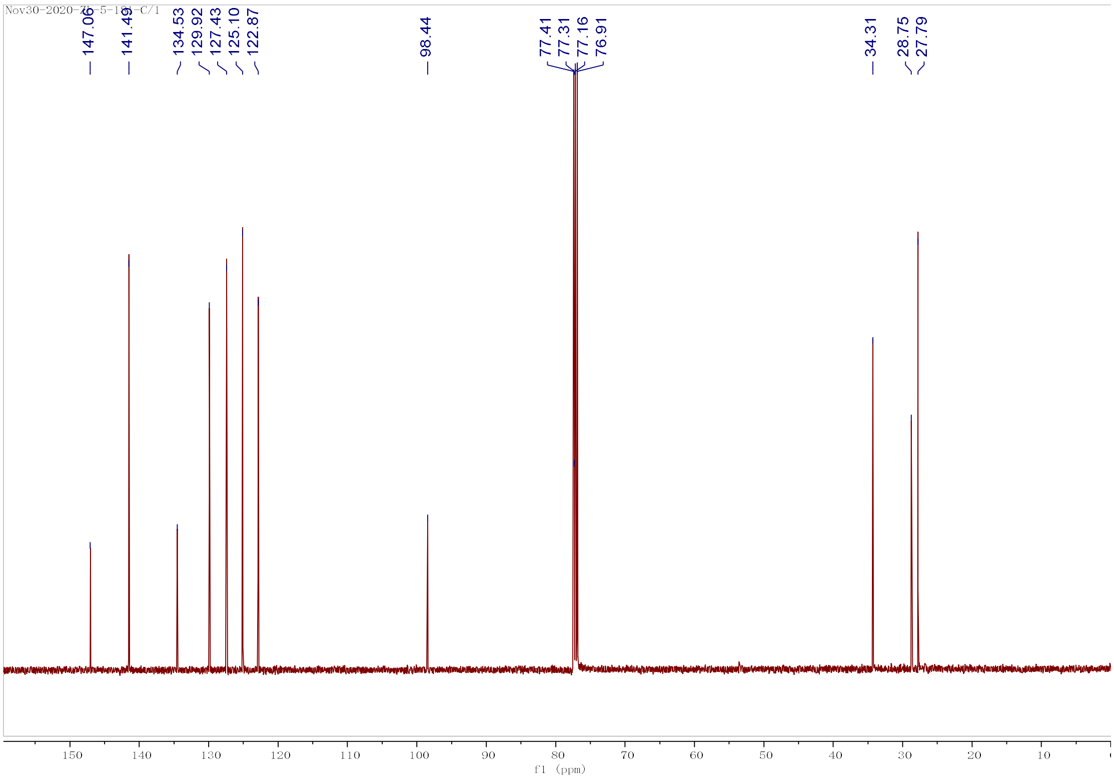

Supplementary Fig. 25. ^{13}C NMR spectrum of **10** ($CDCl_3$, 126 MHz)

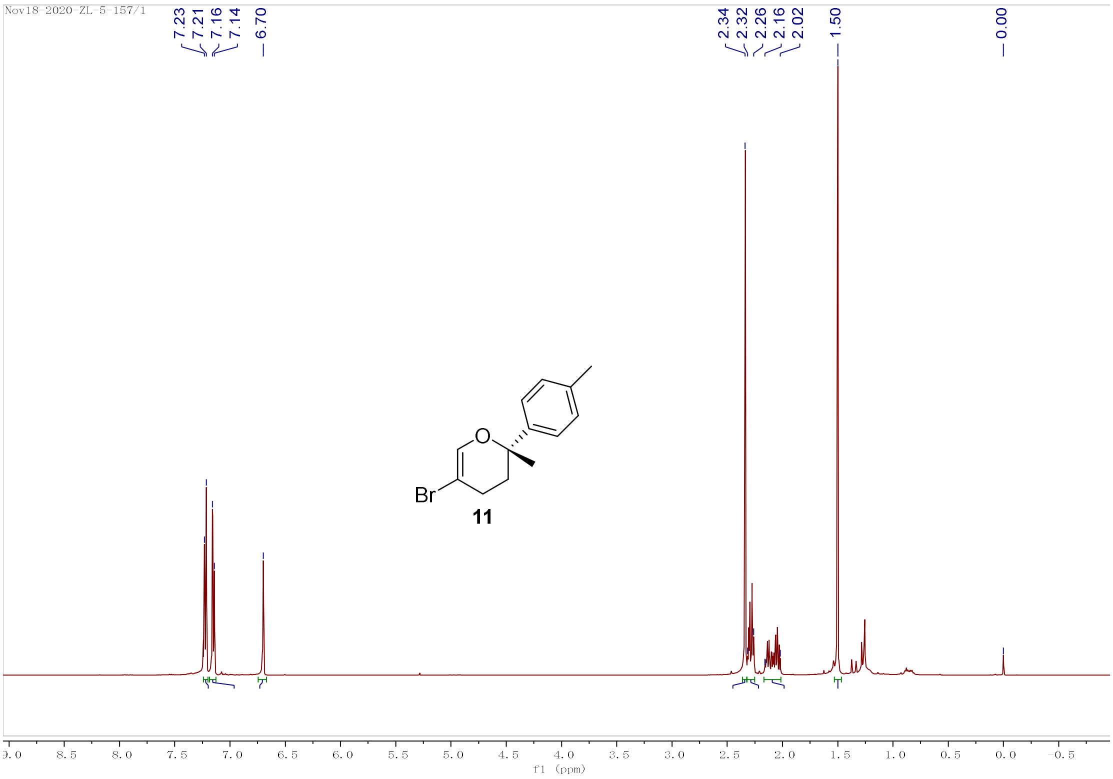

Supplementary Fig. 26. ^{1}H NMR spectrum of **11** ($CDCl_3$, 500 MHz)

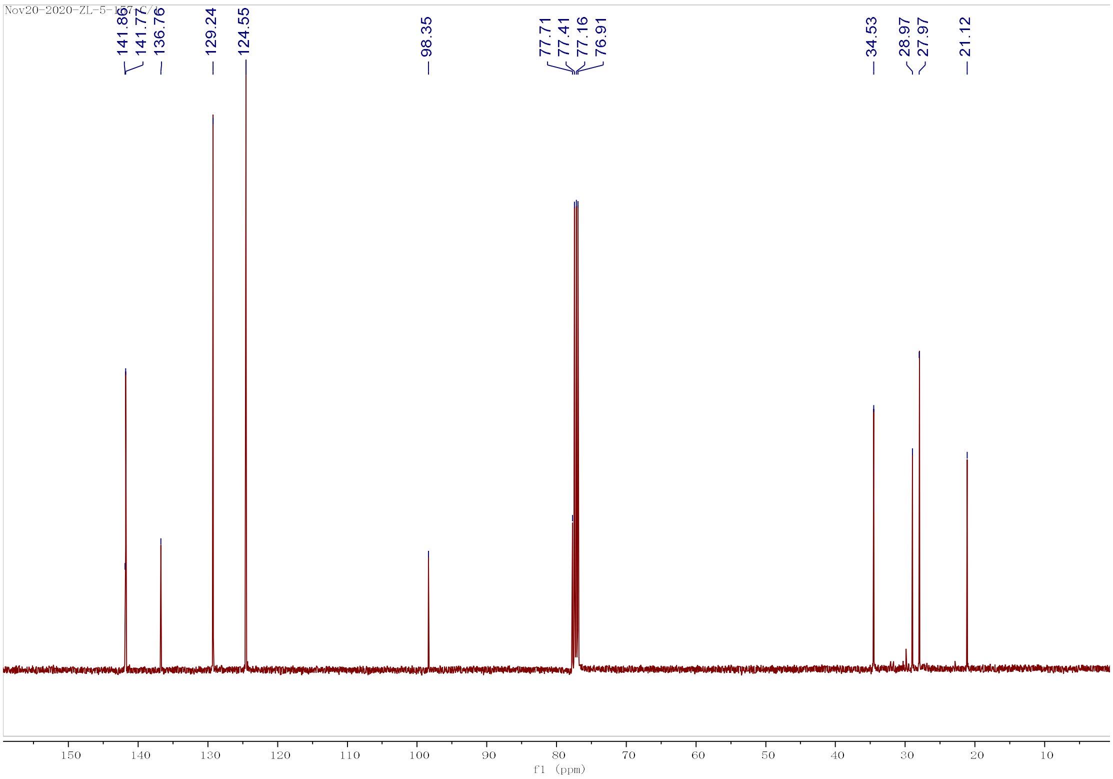

Supplementary Fig. 27. ^{13}C NMR spectrum of **11** ($CDCl_3$, 126 MHz)

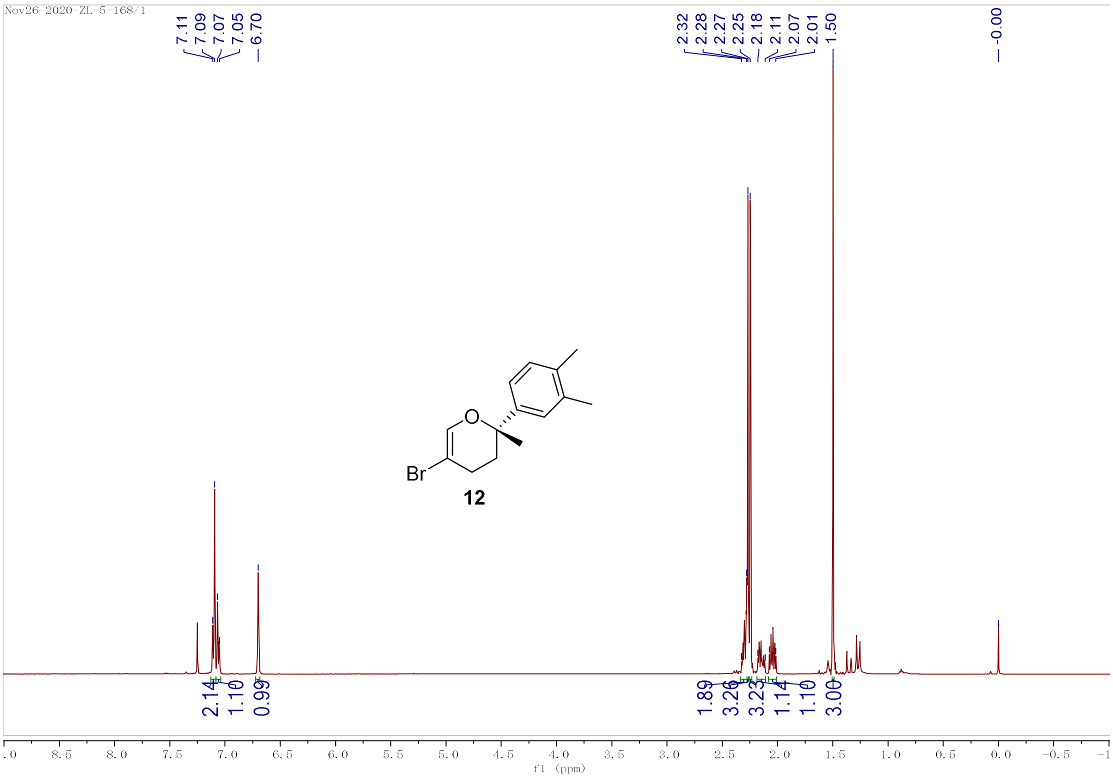

Supplementary Fig. 28. ^{1}H NMR spectrum of **12** ($CDCl_3$, 500 MHz)

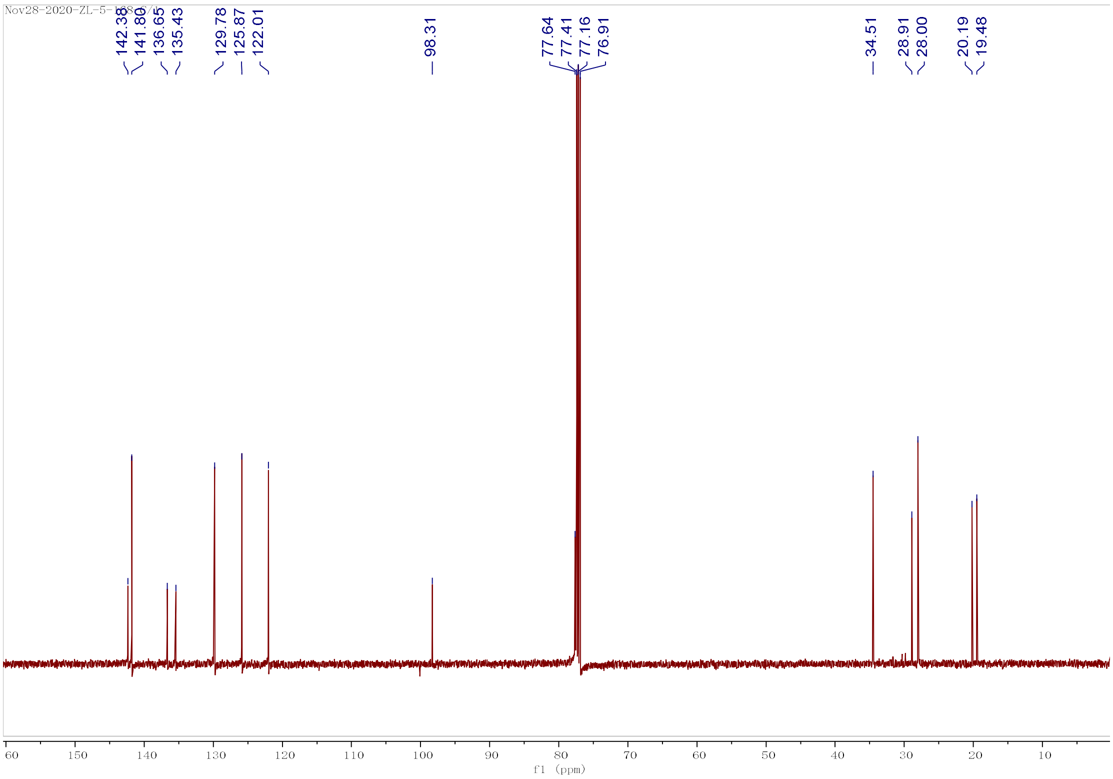

Supplementary Fig. 29. ^{13}C NMR spectrum of **12** ($CDCl_3$, 126 MHz)

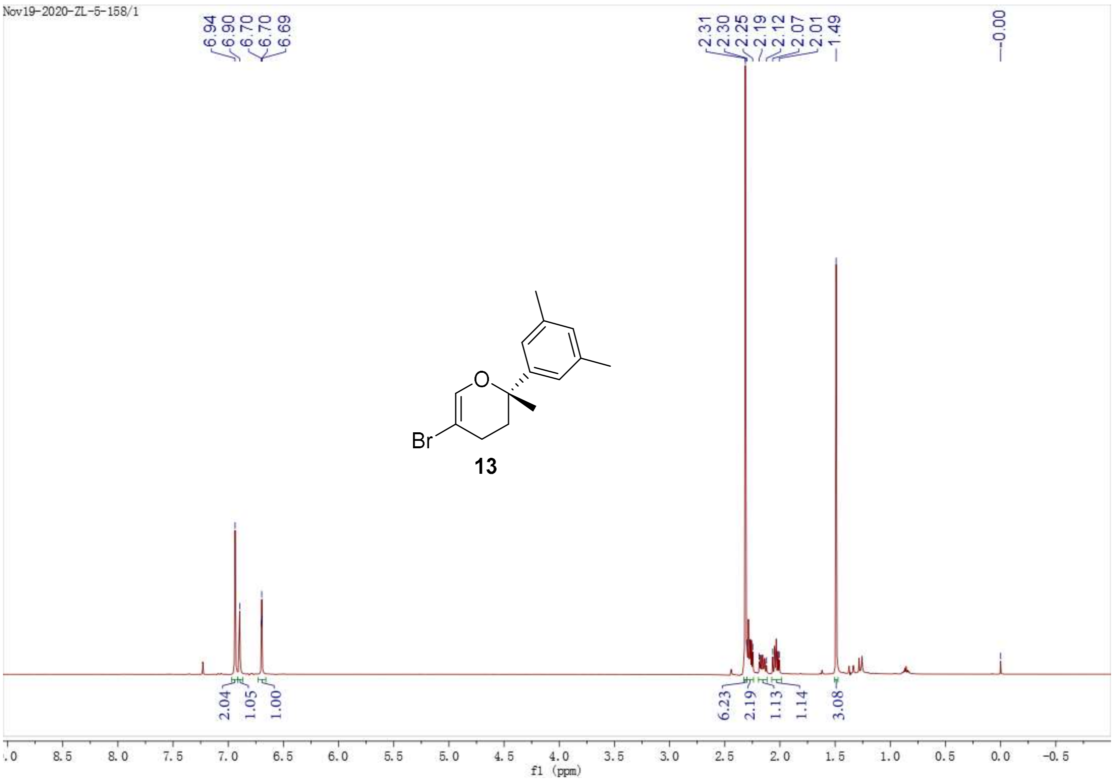

Supplementary Fig. 30. ^{1}H NMR spectrum of **13** ($CDCl_3$, 500 MHz)

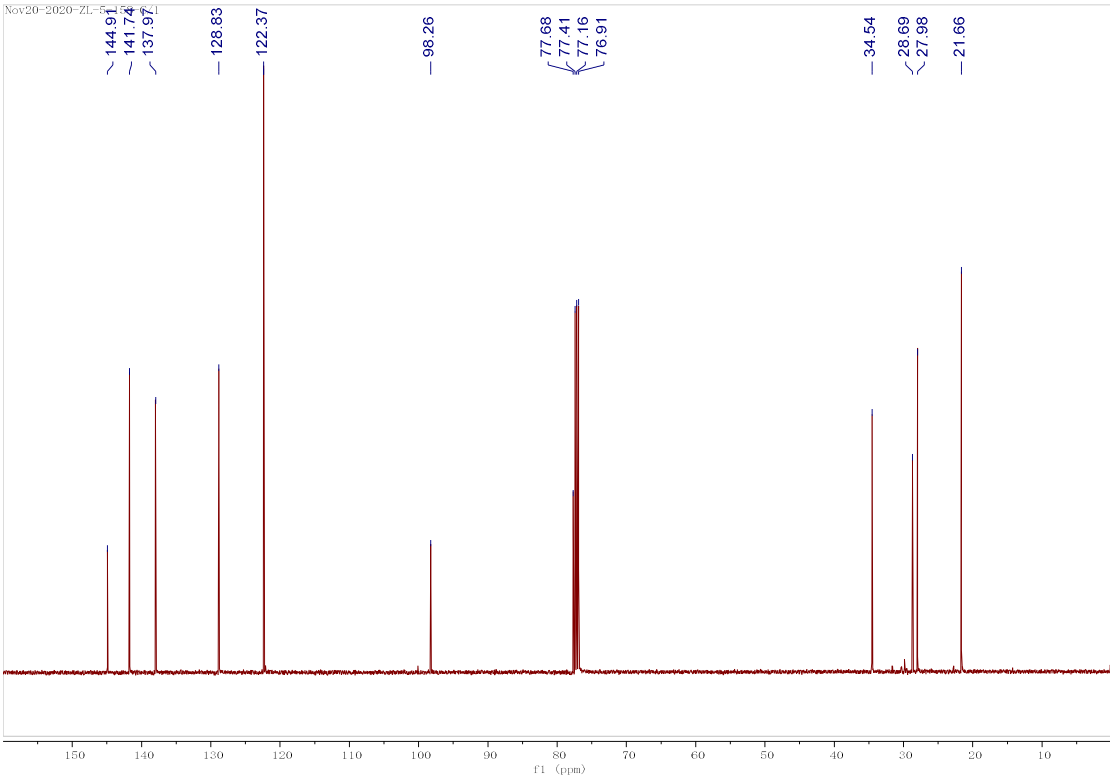

Supplementary Fig. 31. ^{13}C NMR spectrum of **13** ($CDCl_3$, 126 MHz)

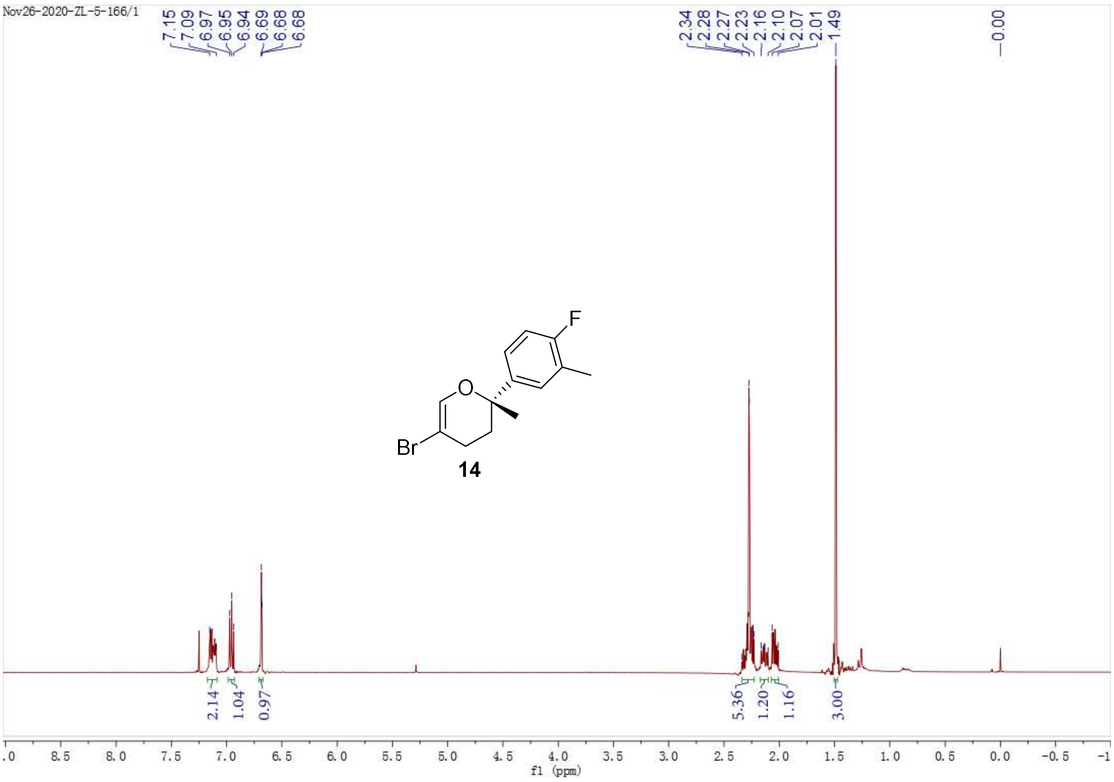

Supplementary Fig. 32. ^{1}H NMR spectrum of **14** ($CDCl_3$, 500 MHz)

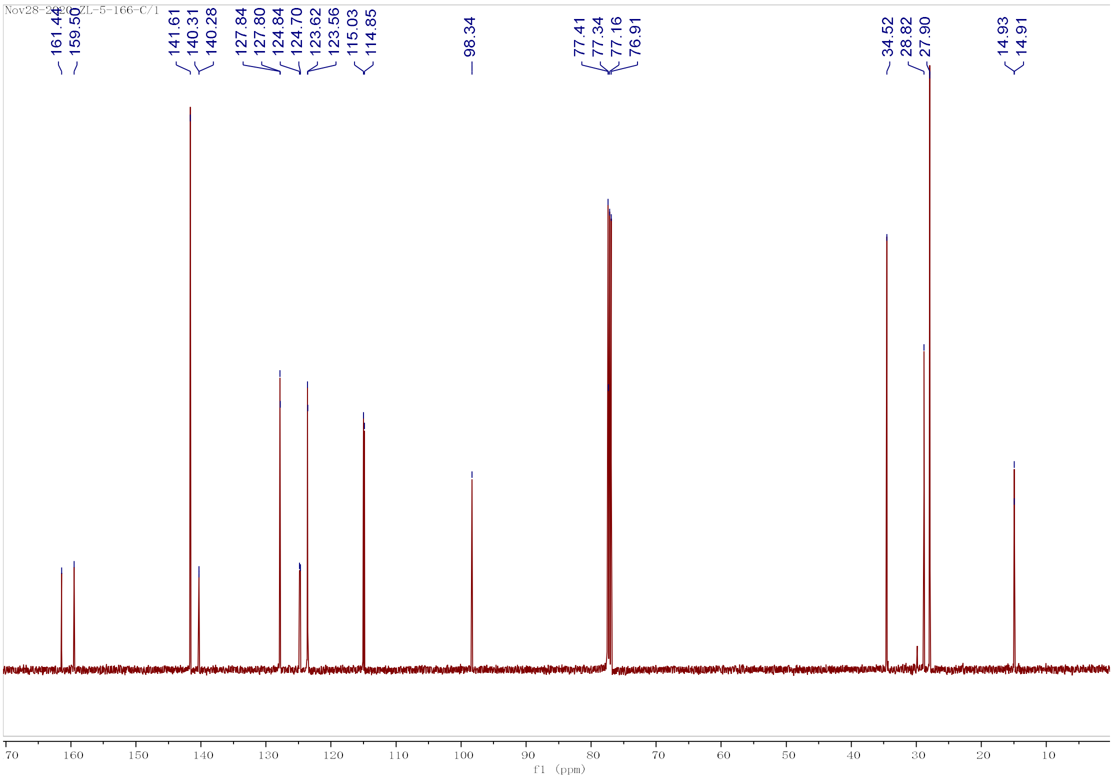

Supplementary Fig. 33. ^{13}C NMR spectrum of **14** ($CDCl_3$, 126 MHz)

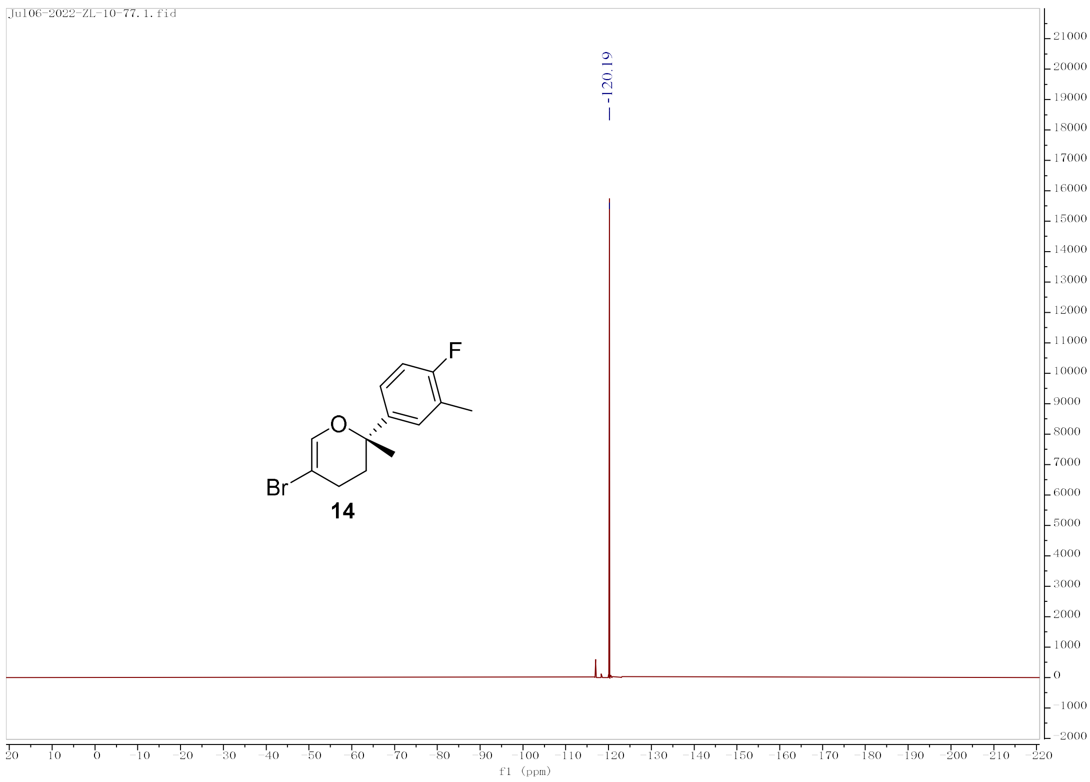

Supplementary Fig. 34. ^{19}F NMR spectrum of **14** in $CDCl_3$

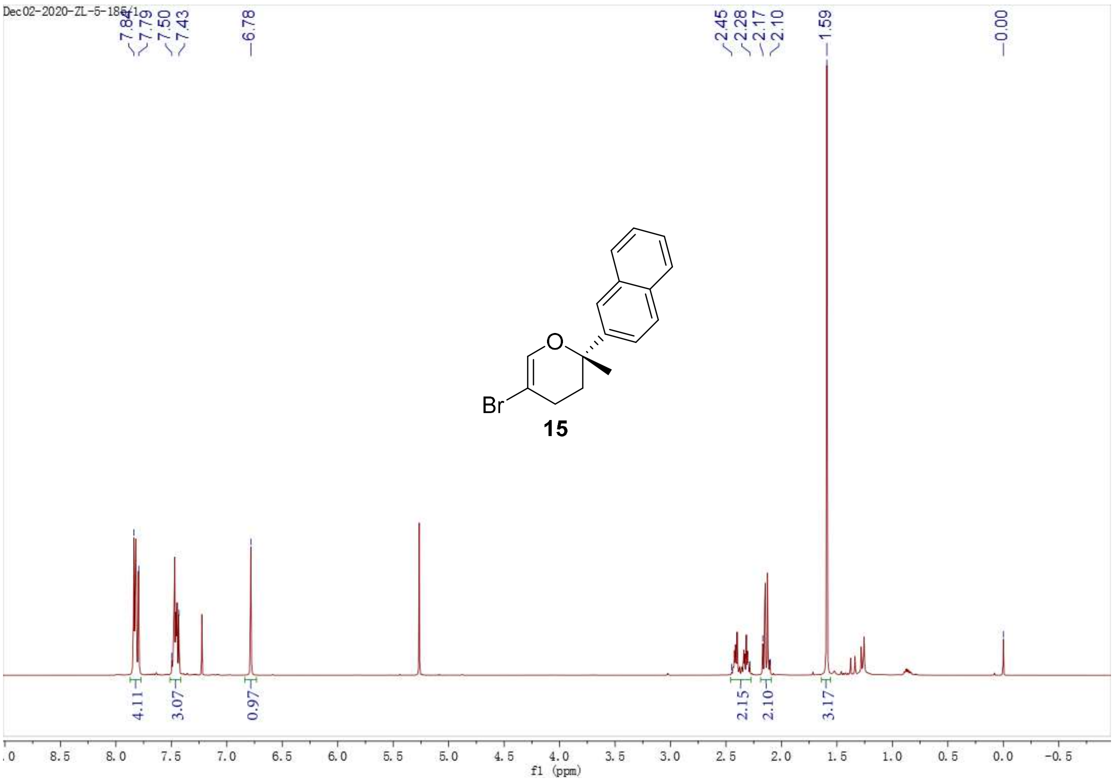

Supplementary Fig. 35. ^{1}H NMR spectrum of **15** ($CDCl_3$, 500 MHz)

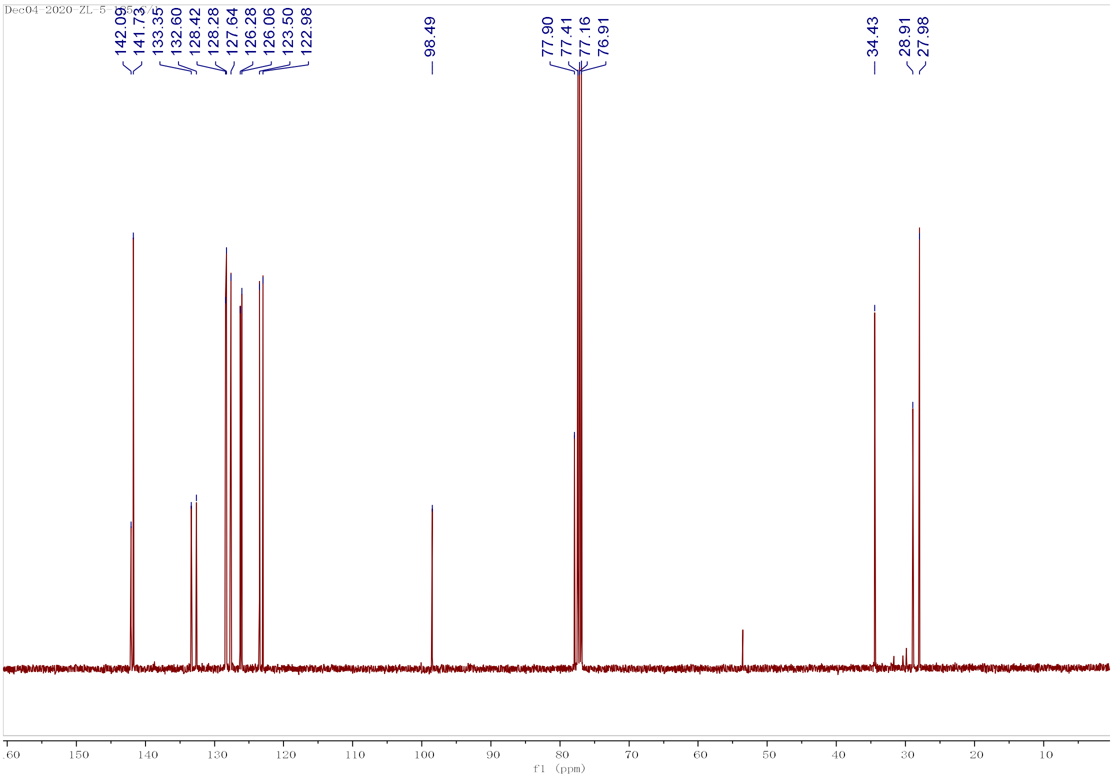

Supplementary Fig. 36. ^{13}C NMR spectrum of **15** ($CDCl_3$, 126 MHz)

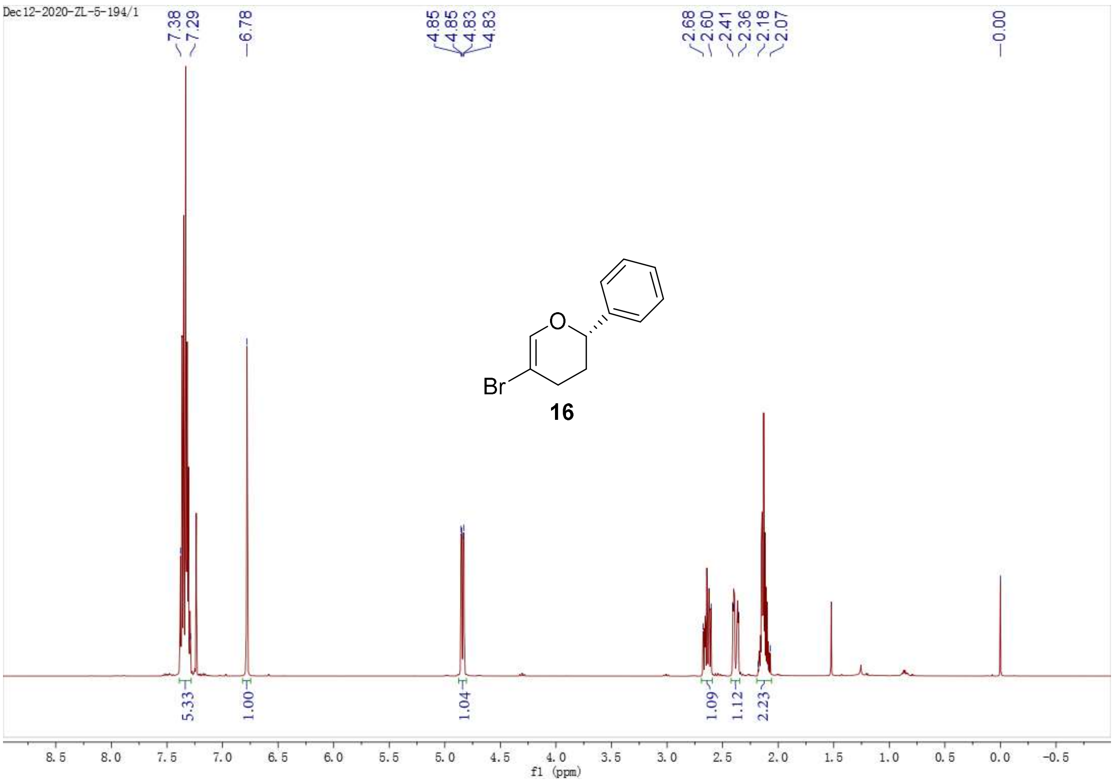

Supplementary Fig. 37. ^{1}H NMR spectrum of **16** ($CDCl_3$, 500 MHz)

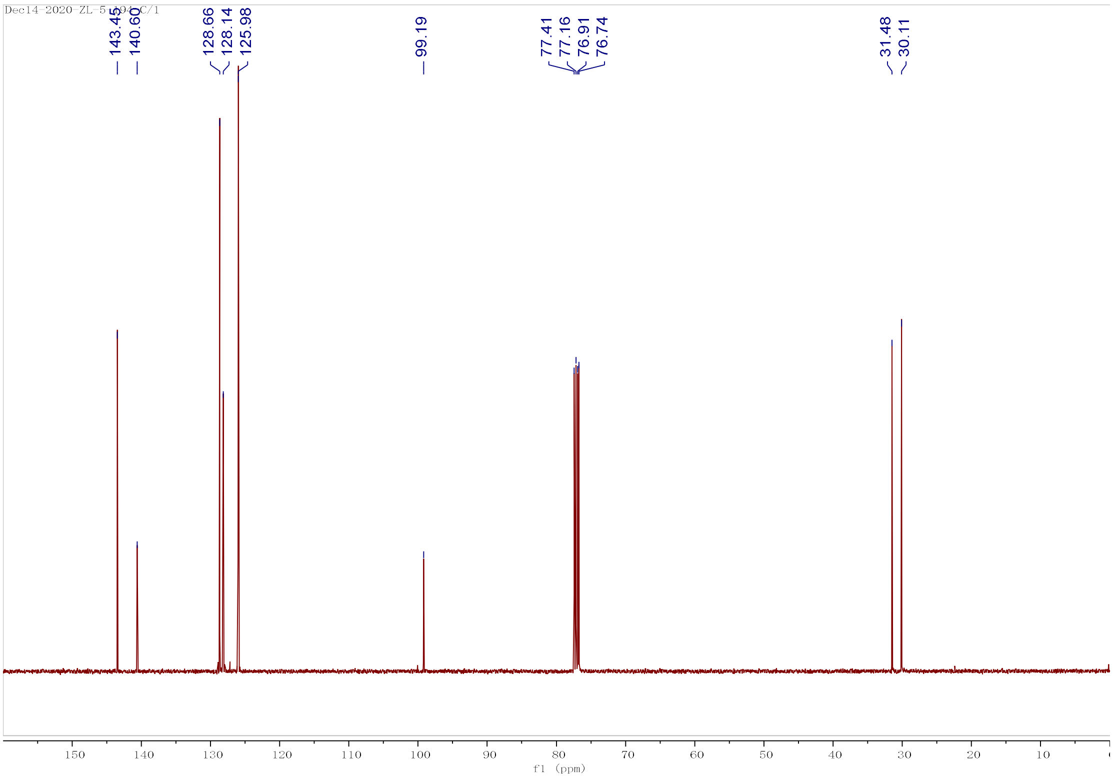

Supplementary Fig. 38. ^{13}C NMR spectrum of **16** ($CDCl_3$, 126 MHz)

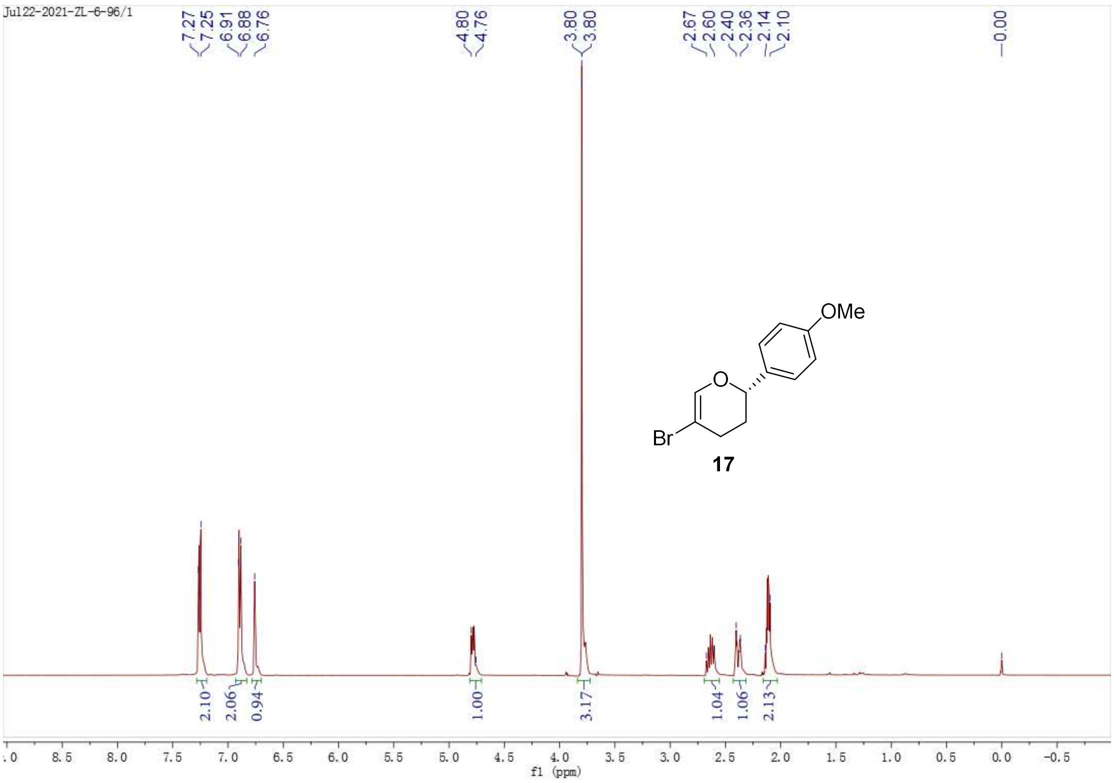

Supplementary Fig. 39. ^{1}H NMR spectrum of **17** ($CDCl_3$, 500 MHz)

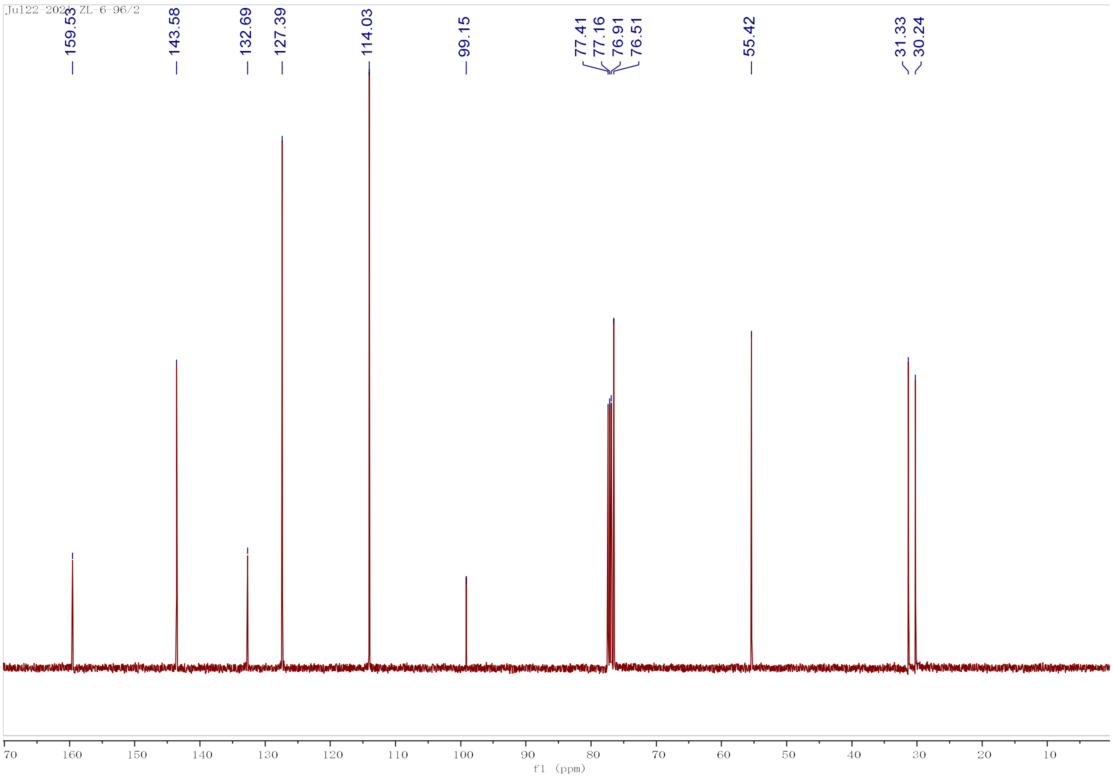

Supplementary Fig. 40. ^{13}C NMR spectrum of **17** ($CDCl_3$, 126 MHz)

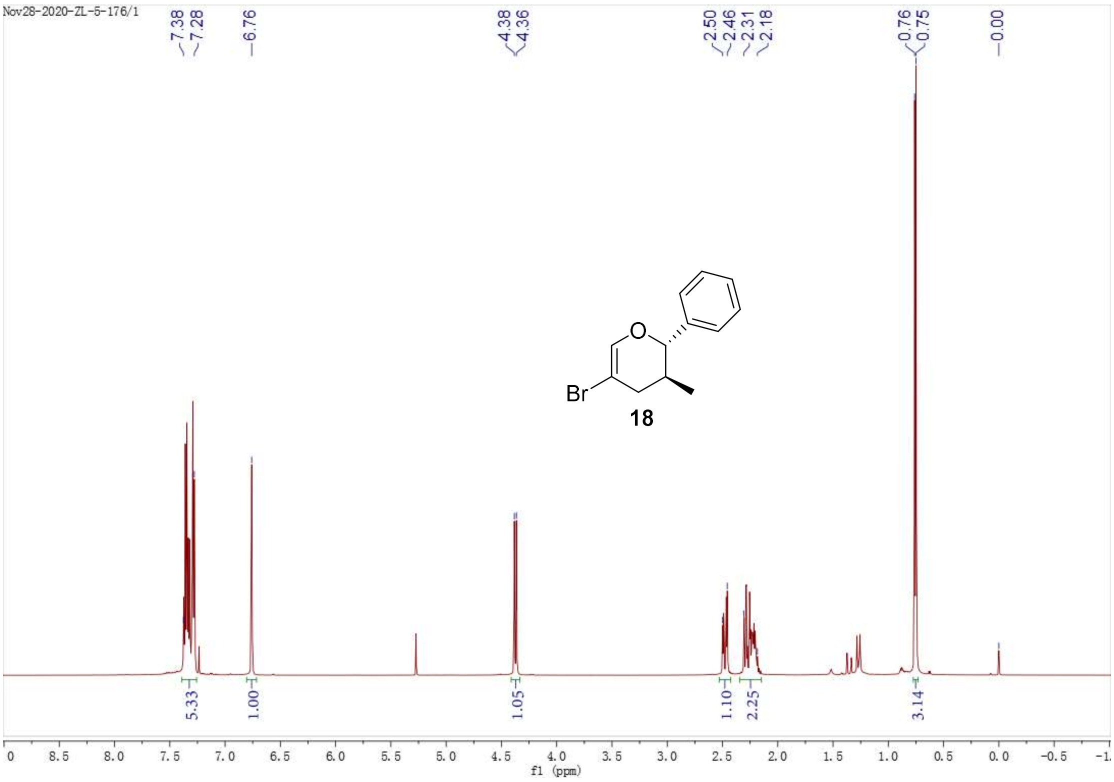

Supplementary Fig. 41. ^{1}H NMR spectrum of **18** ($CDCl_3$, 500 MHz)

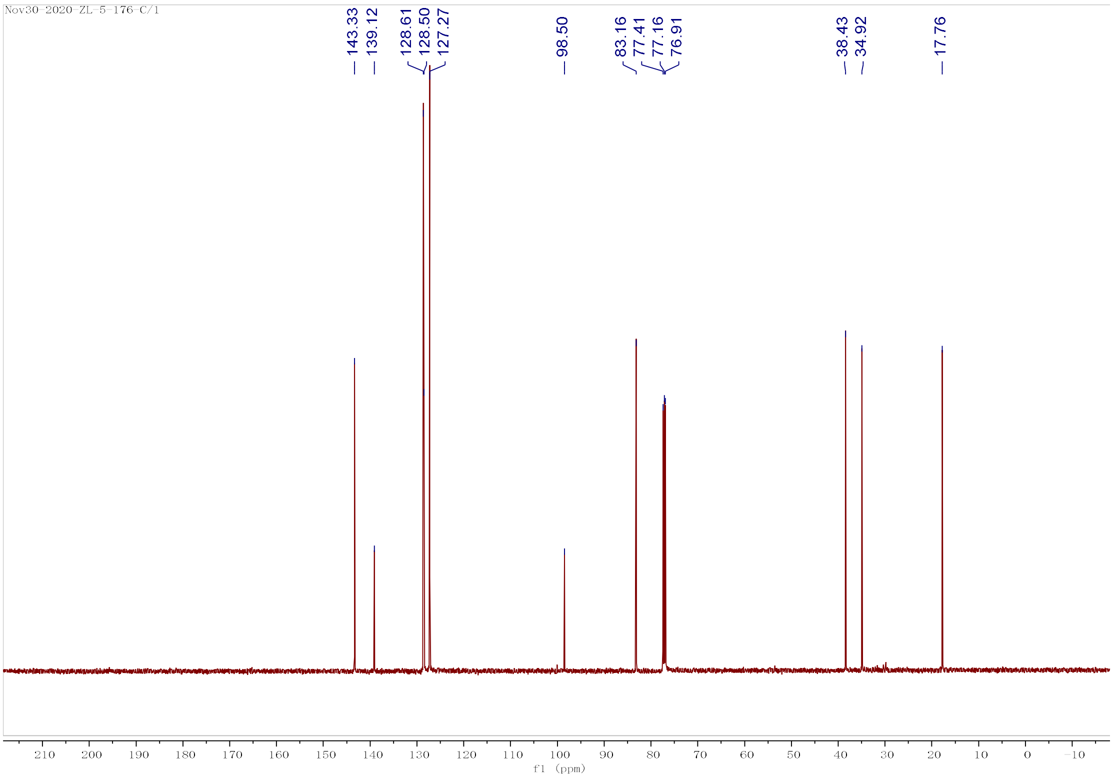

Supplementary Fig. 42. ^{13}C NMR spectrum of **18** ($CDCl_3$, 126 MHz)

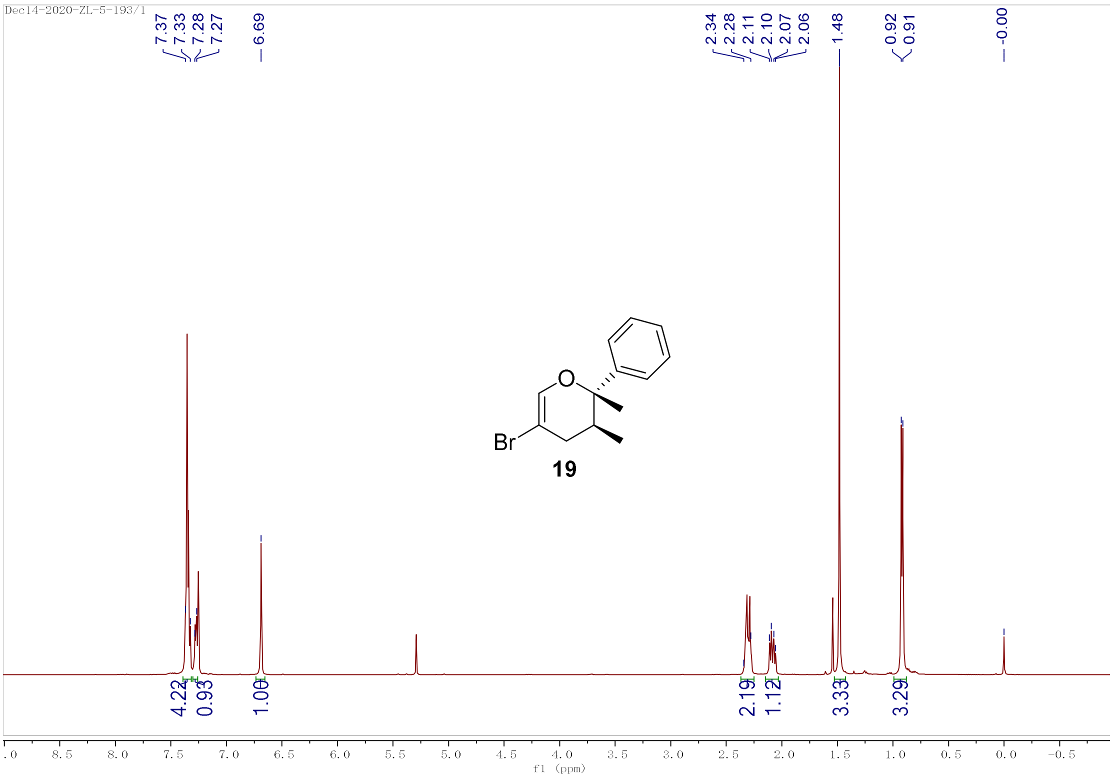

Supplementary Fig. 43. ^{1}H NMR spectrum of **19** ($CDCl_3$, 500 MHz)

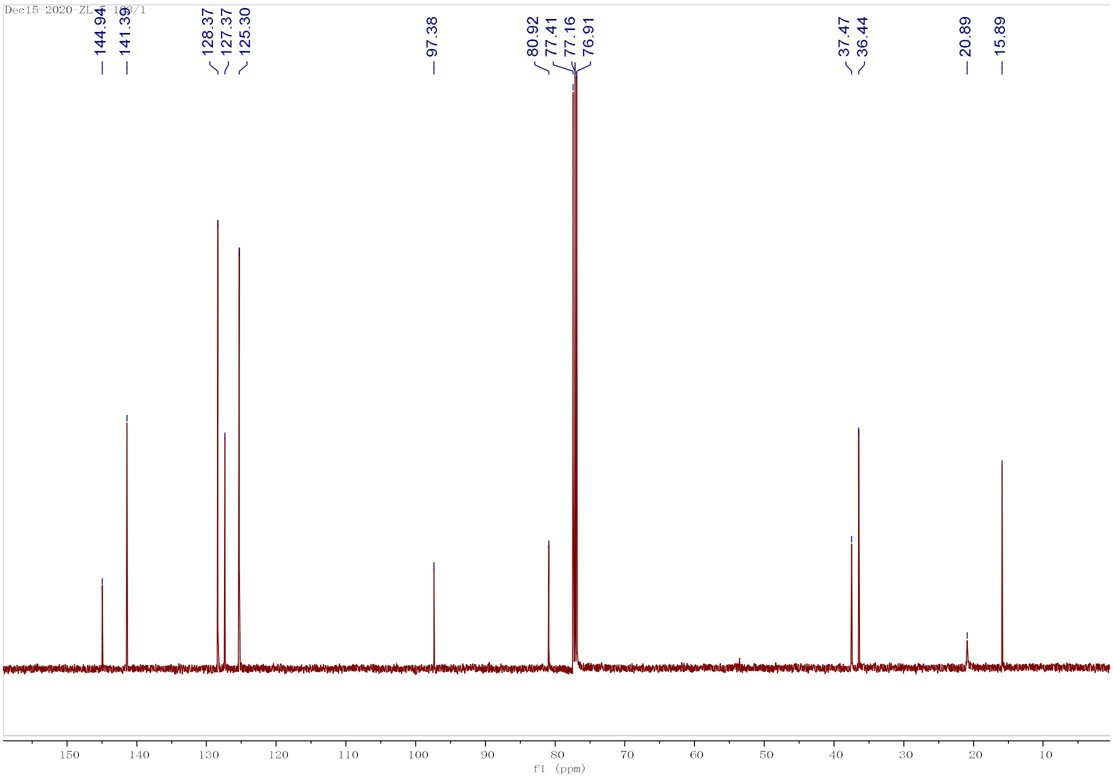

Supplementary Fig. 44. ^{13}C NMR spectrum of **19** ($CDCl_3$, 126 MHz)

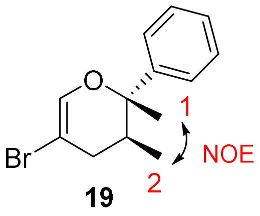

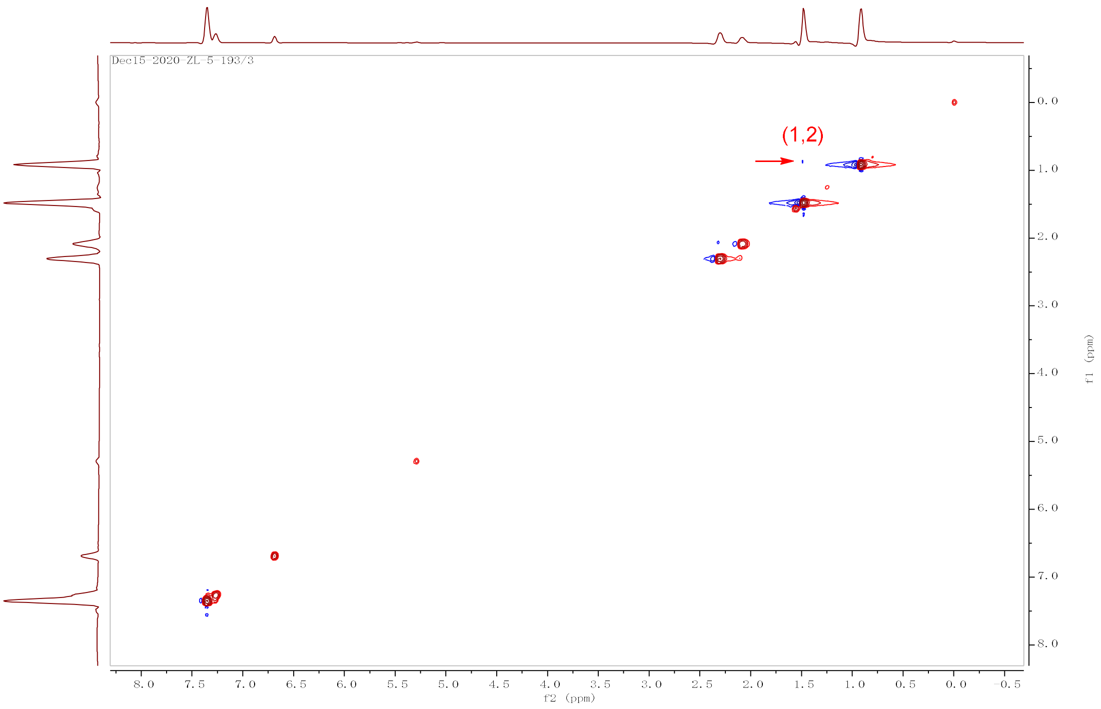

Supplementary Fig. 45. NOE spectrum of **19** ($CDCl_3$, 500 MHz)

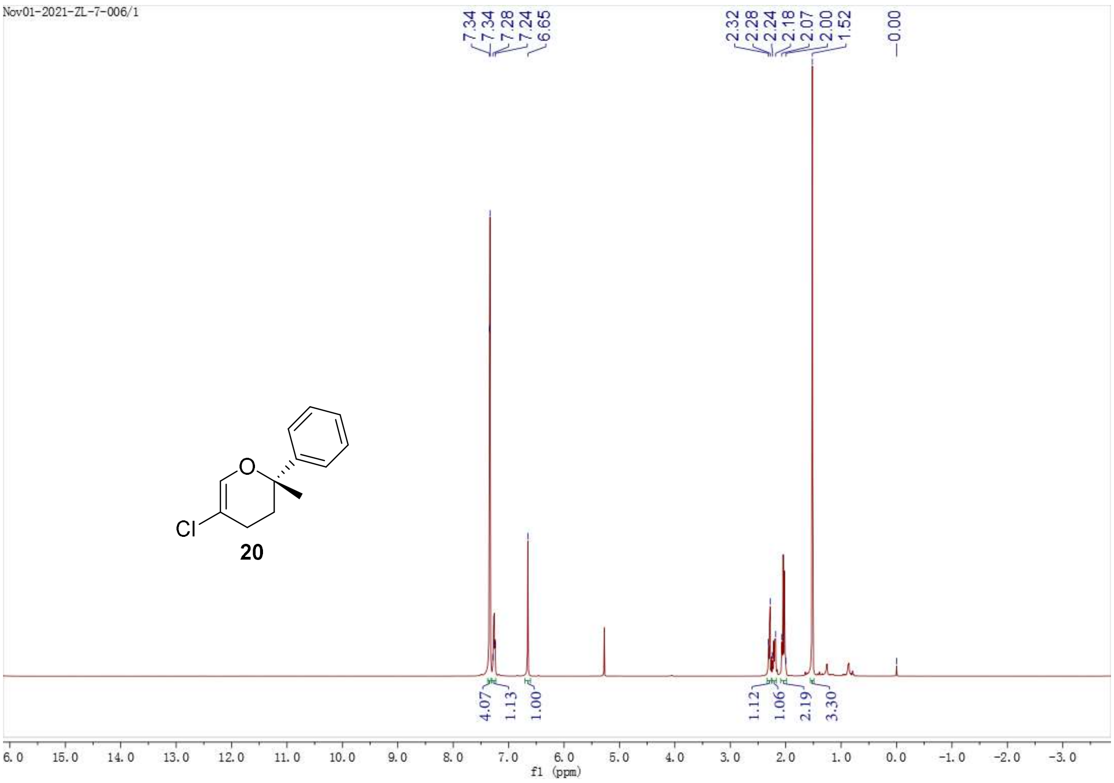

Supplementary Fig. 46. ^{1}H NMR spectrum of **20** ($CDCl_3$, 500 MHz)

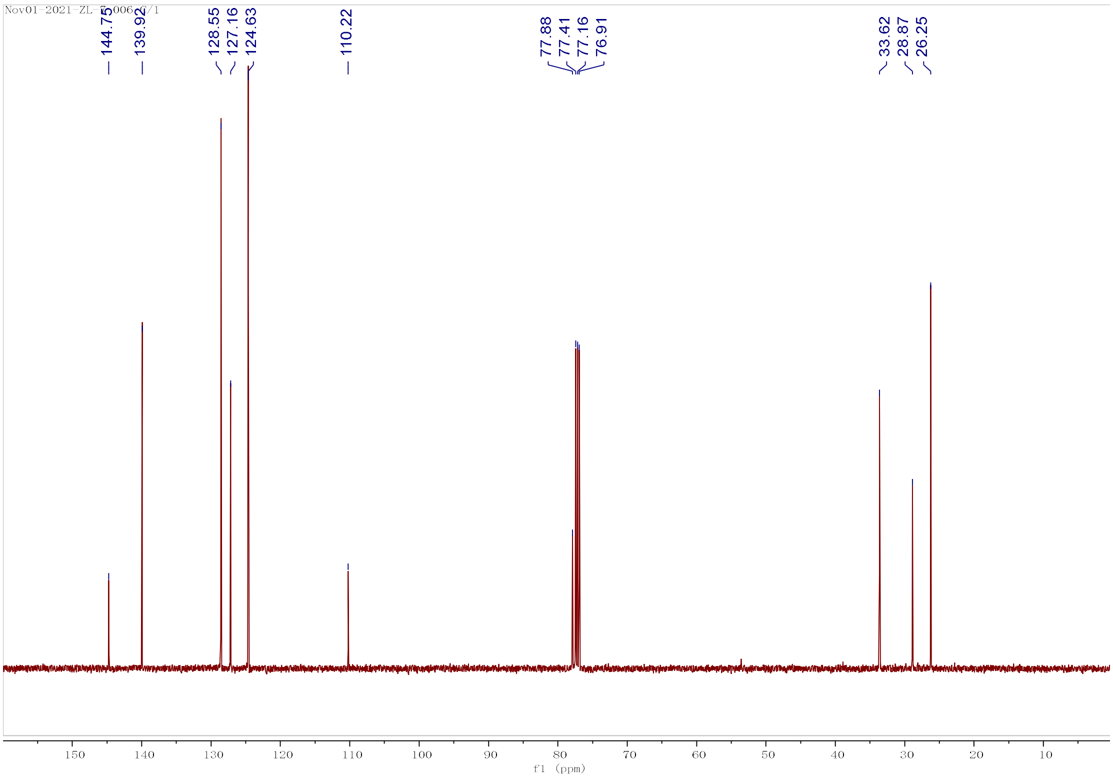

Supplementary Fig. 47. ^{13}C NMR spectrum of **20** ($CDCl_3$, 126 MHz)

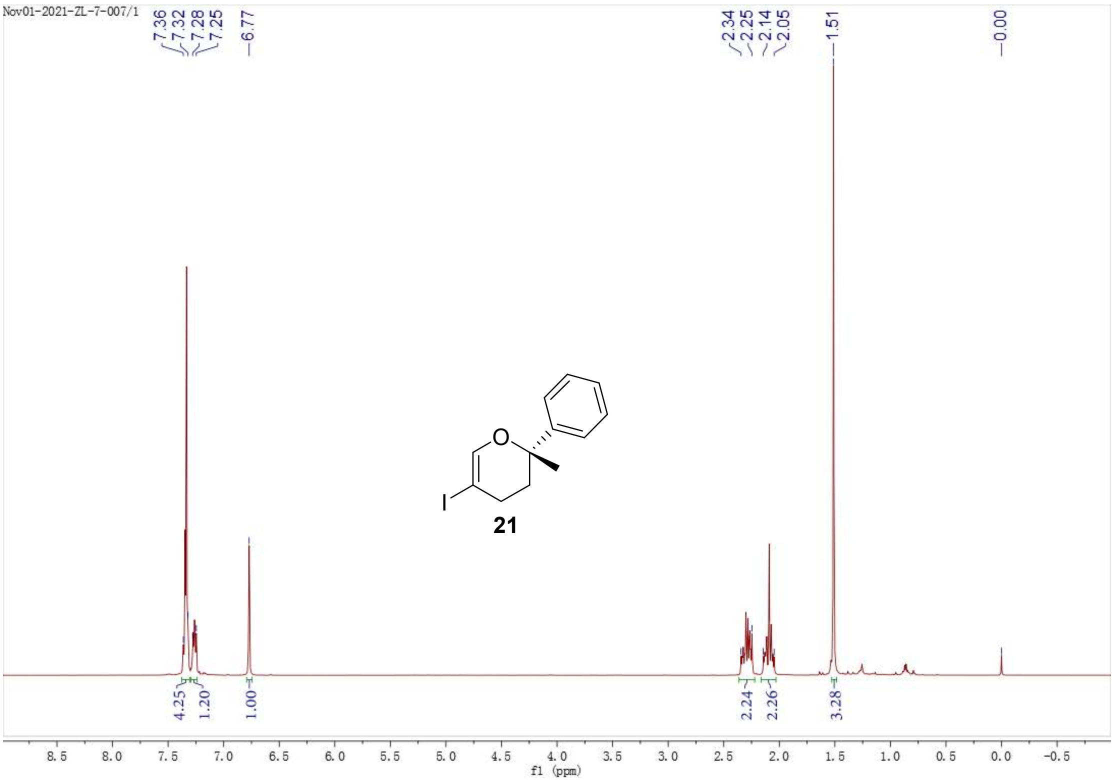

Supplementary Fig. 48. ^{1}H NMR spectrum of **21** ($CDCl_3$, 500 MHz)

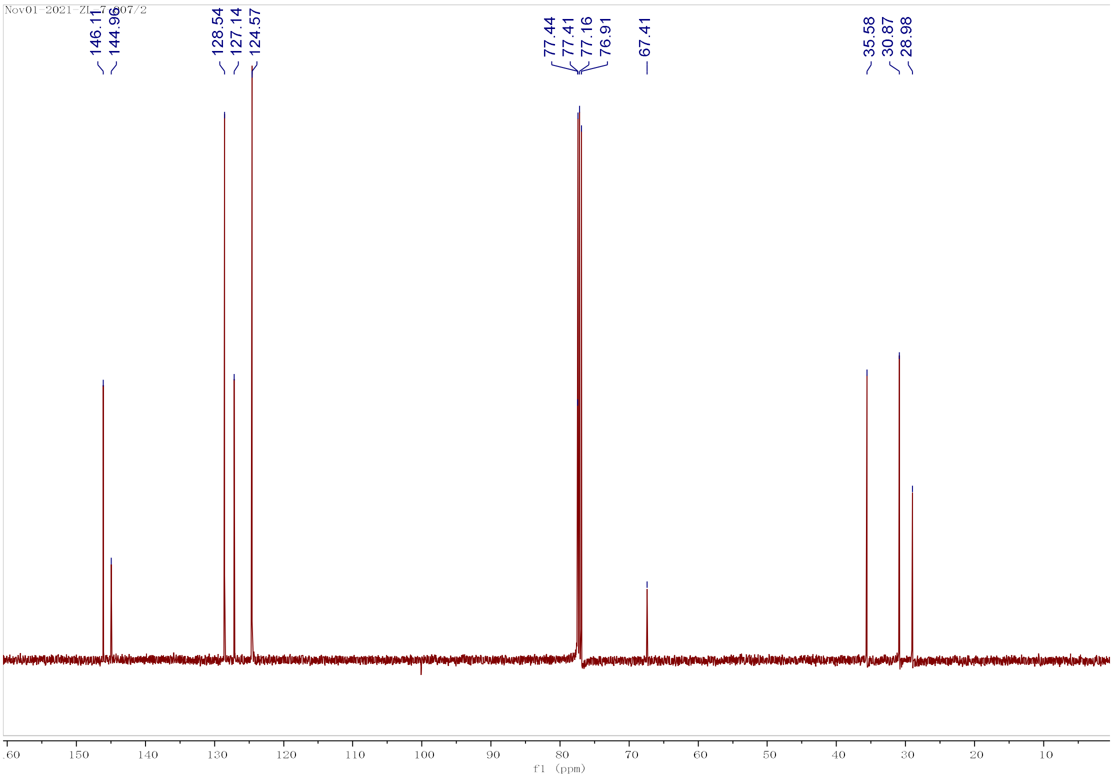

Supplementary Fig. 49. ^{13}C NMR spectrum of **21** ($CDCl_3$, 126 MHz)

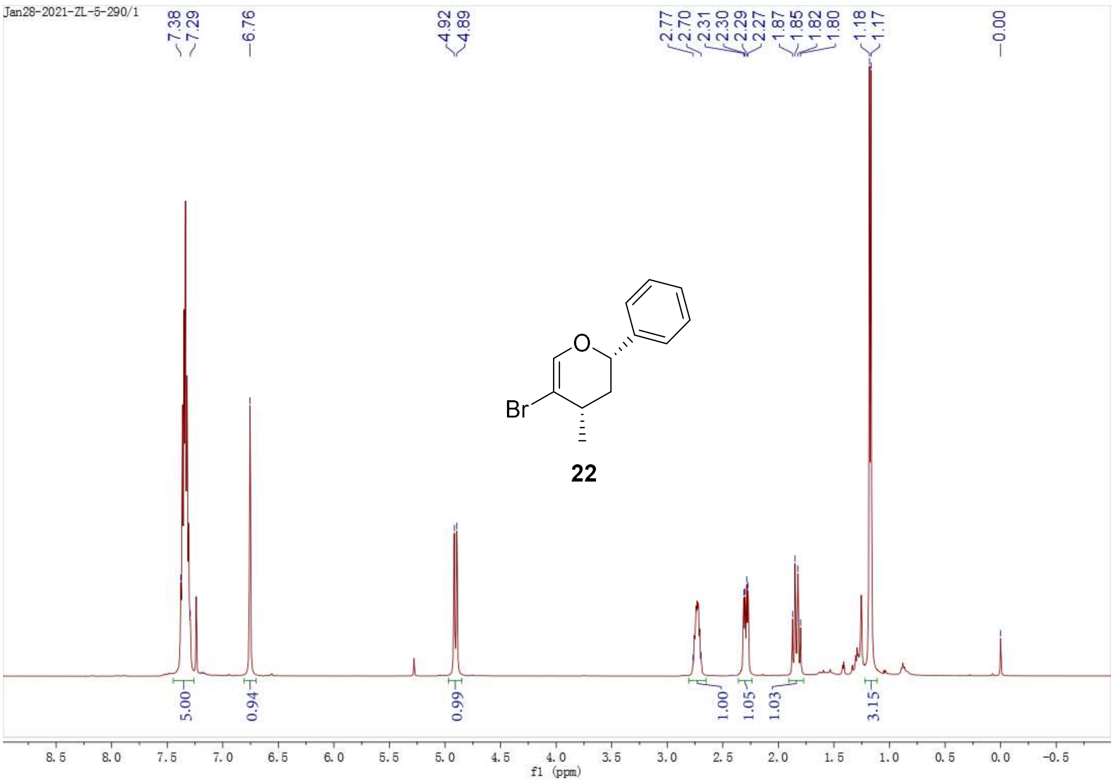

Supplementary Fig. 50. ^{1}H NMR spectrum of **22** ($CDCl_3$, 500 MHz)

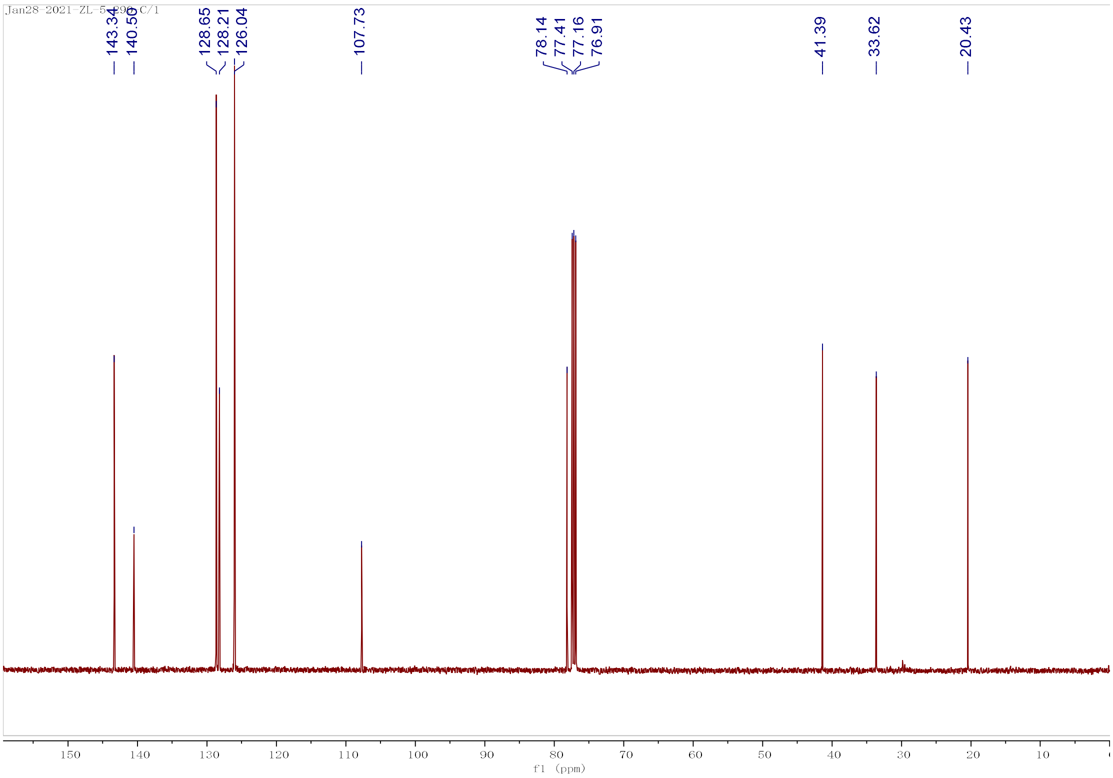

Supplementary Fig. 51. ^{13}C NMR spectrum of **22** ($CDCl_3$, 126 MHz)

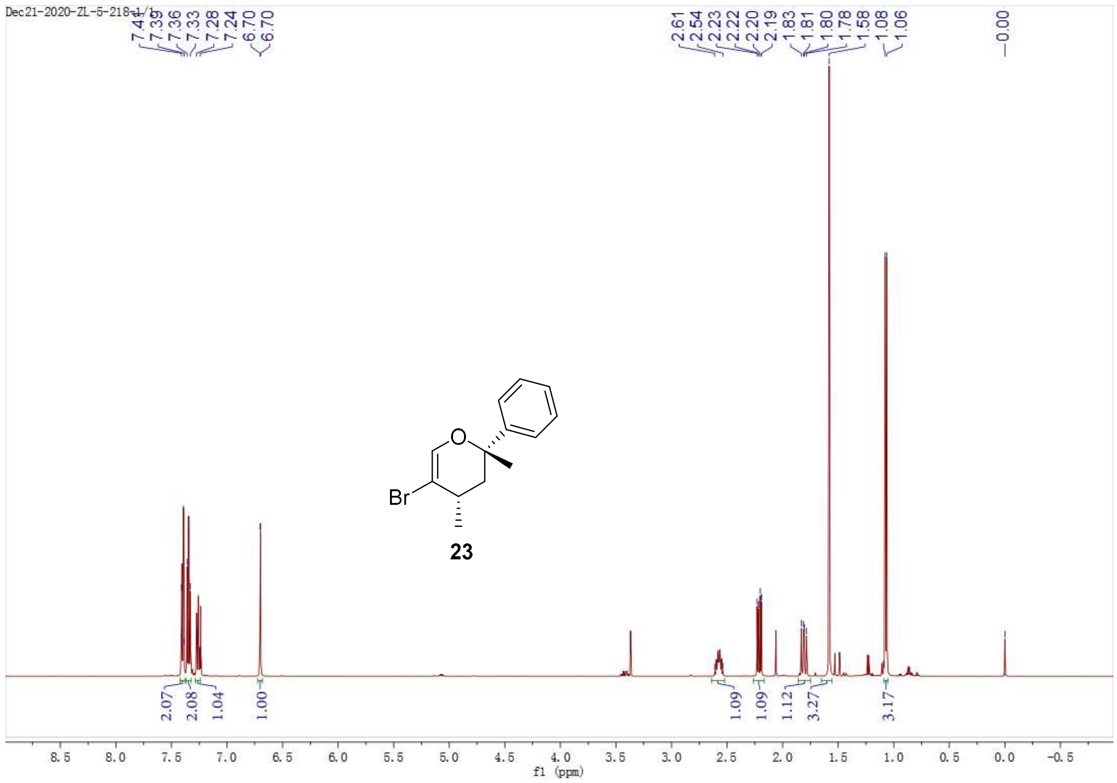

Supplementary Fig. 52. ^{1}H NMR spectrum of **23** ($CDCl_3$, 500 MHz)

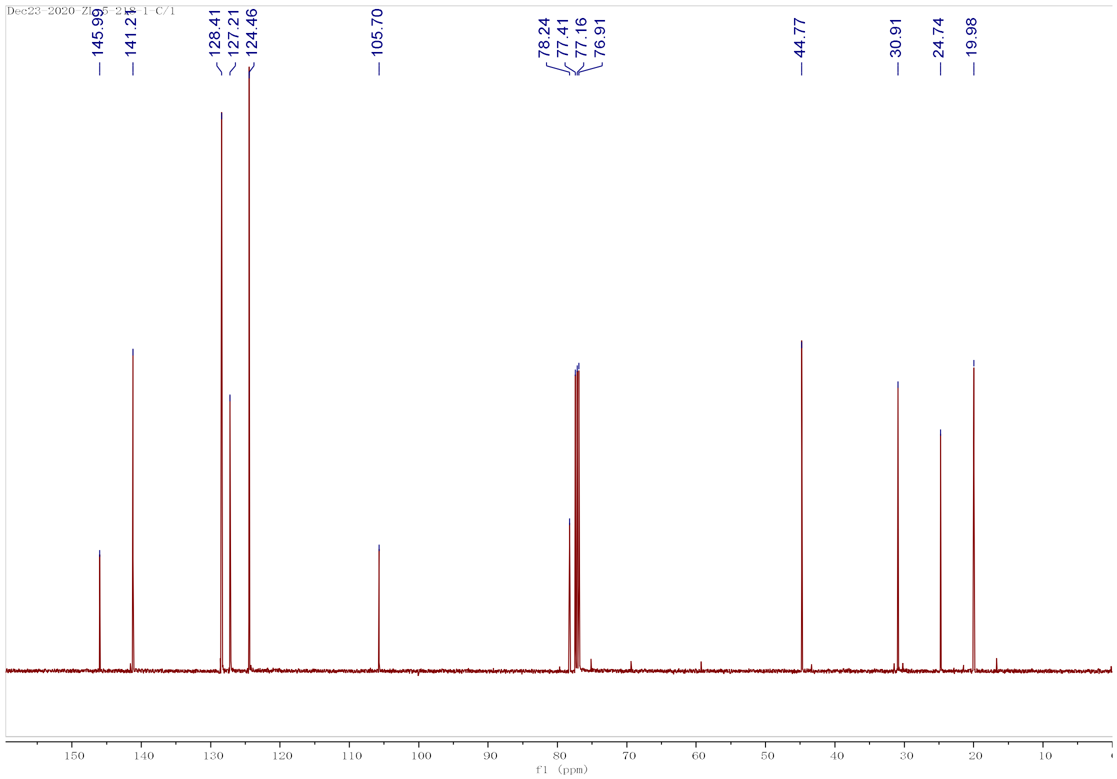

Supplementary Fig. 53. ^{13}C NMR spectrum of **23** ($CDCl_3$, 126 MHz)

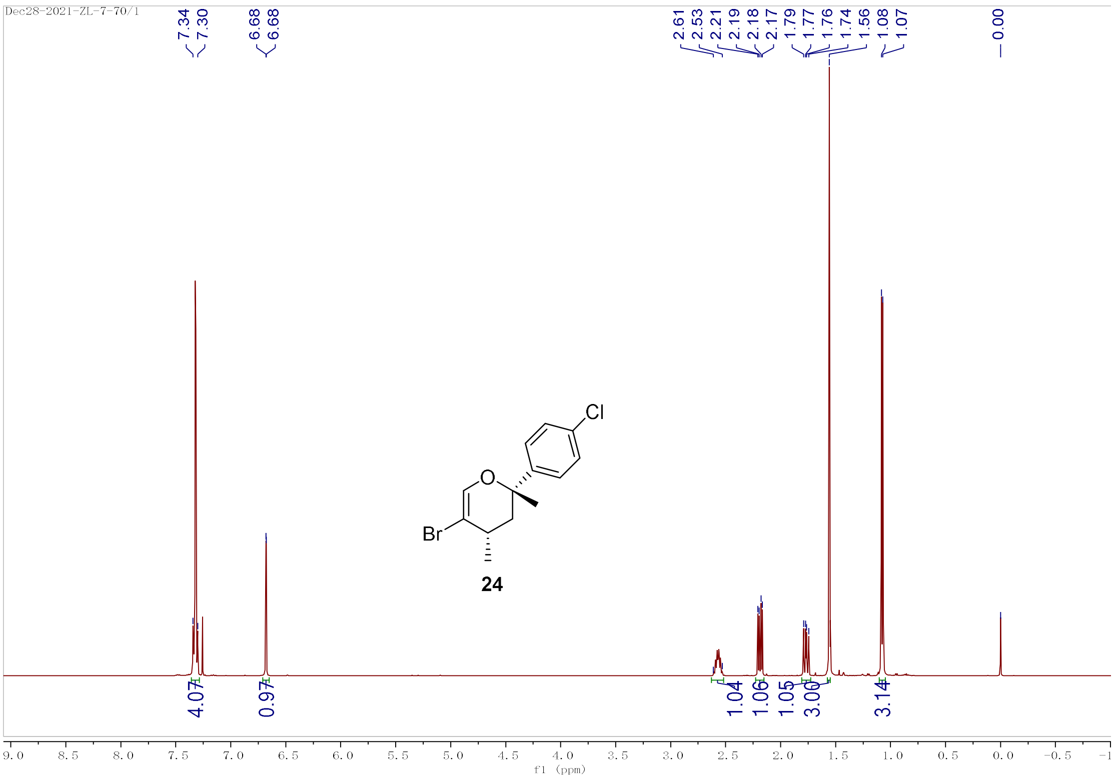

Supplementary Fig. 54. ^{1}H NMR spectrum of **24** ($CDCl_3$, 500 MHz)

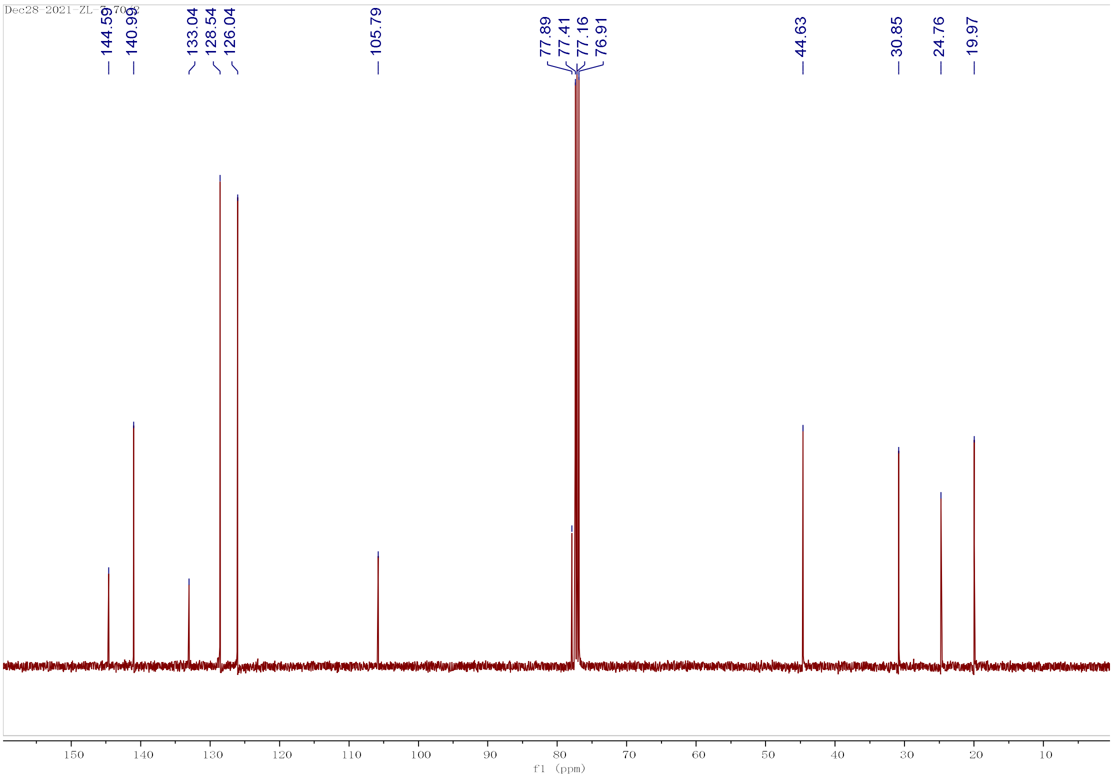

Supplementary Fig. 55. ^{13}C NMR spectrum of **24** ($CDCl_3$, 126 MHz)

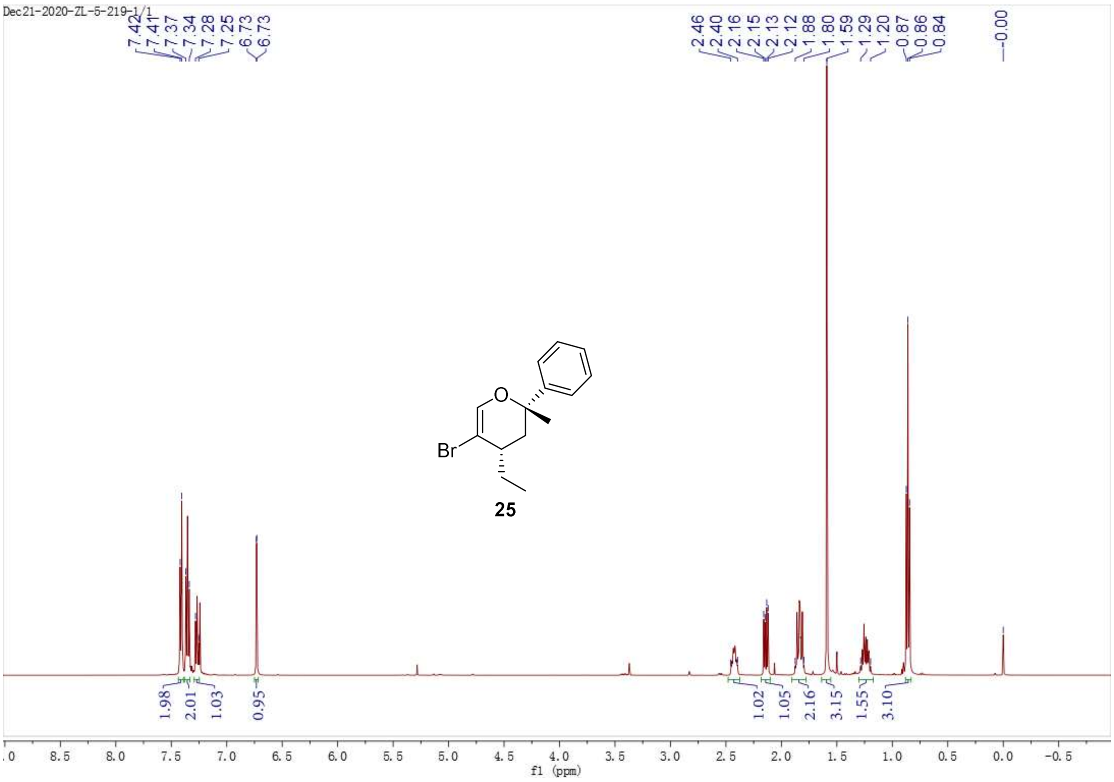

Supplementary Fig. 56. ^{1}H NMR spectrum of **25** ($CDCl_3$, 500 MHz)

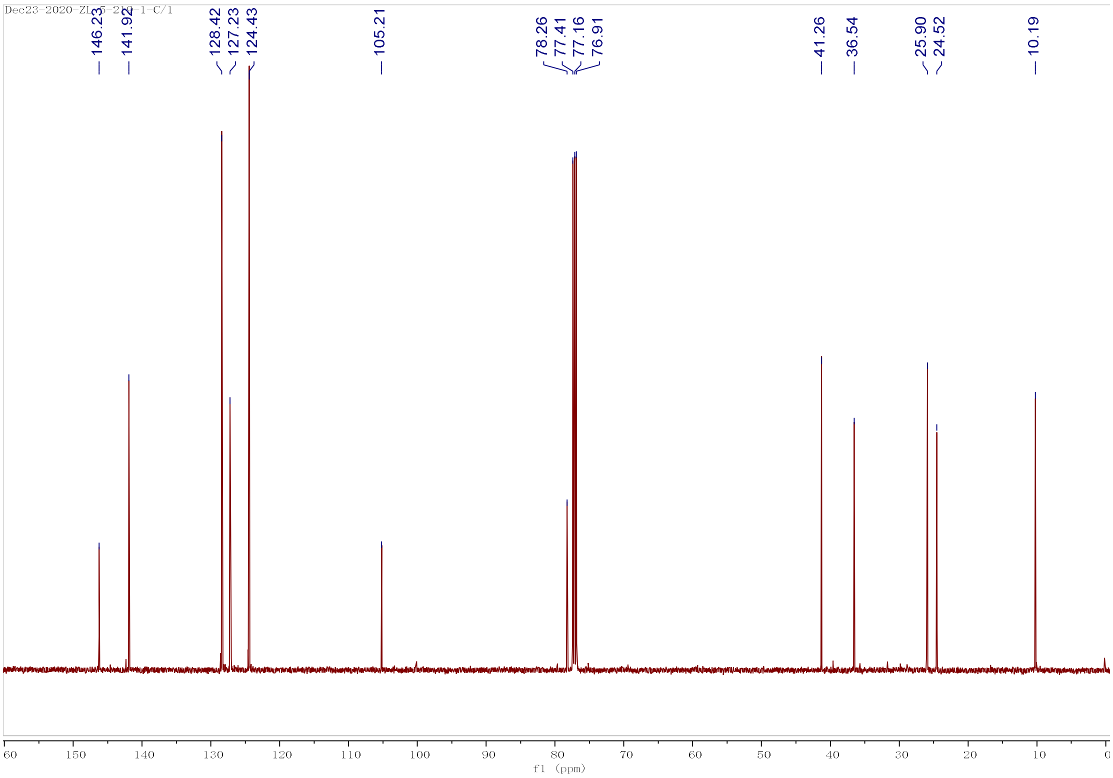

Supplementary Fig. 57. ^{13}C NMR spectrum of **25** ($CDCl_3$, 126 MHz)

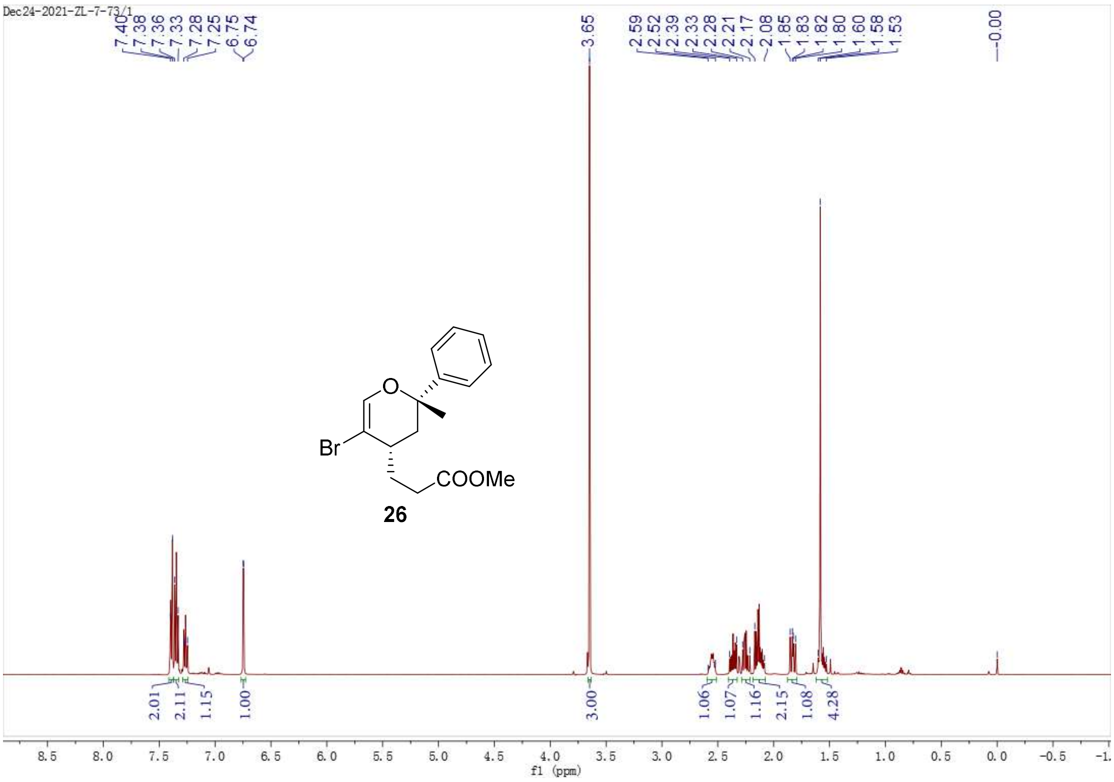

Supplementary Fig. 58. ^{1}H NMR spectrum of **26** ($CDCl_3$, 500 MHz)

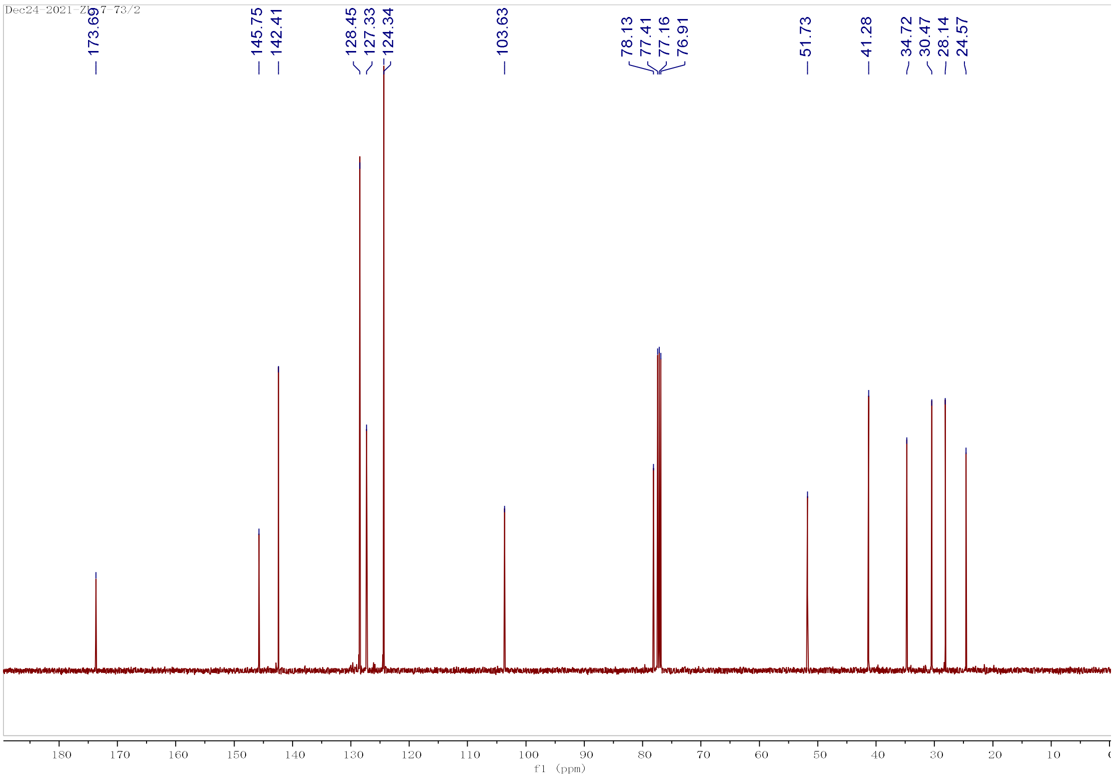

Supplementary Fig. 59. ^{13}C NMR spectrum of **26** ($CDCl_3$, 126 MHz)

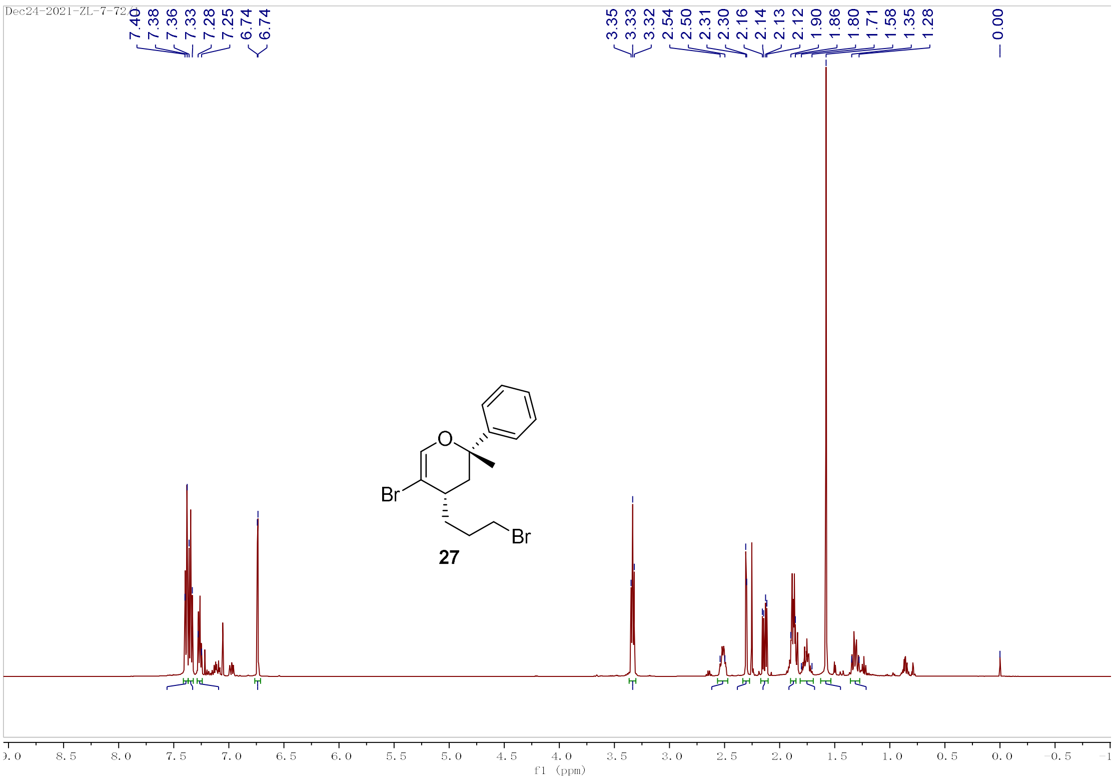

Supplementary Fig. 60. ^{1}H NMR spectrum of **27** ($CDCl_3$, 500 MHz)

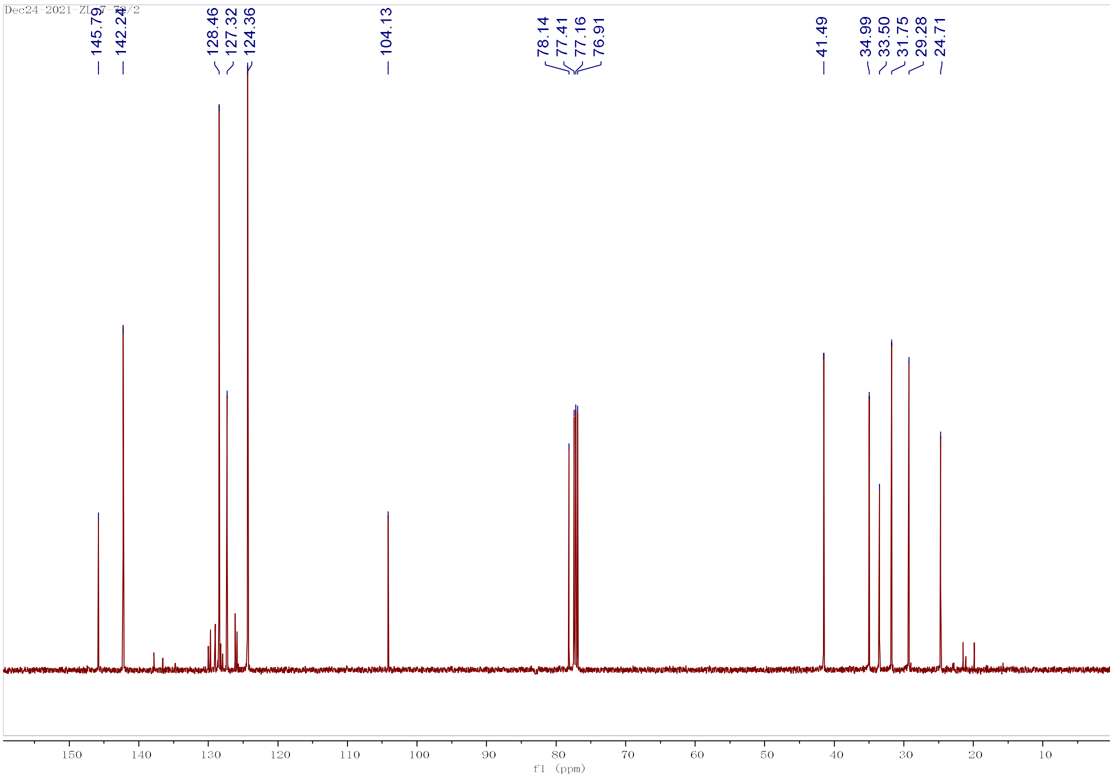

Supplementary Fig. 61. ^{13}C NMR spectrum of **27** ($CDCl_3$, 126 MHz)

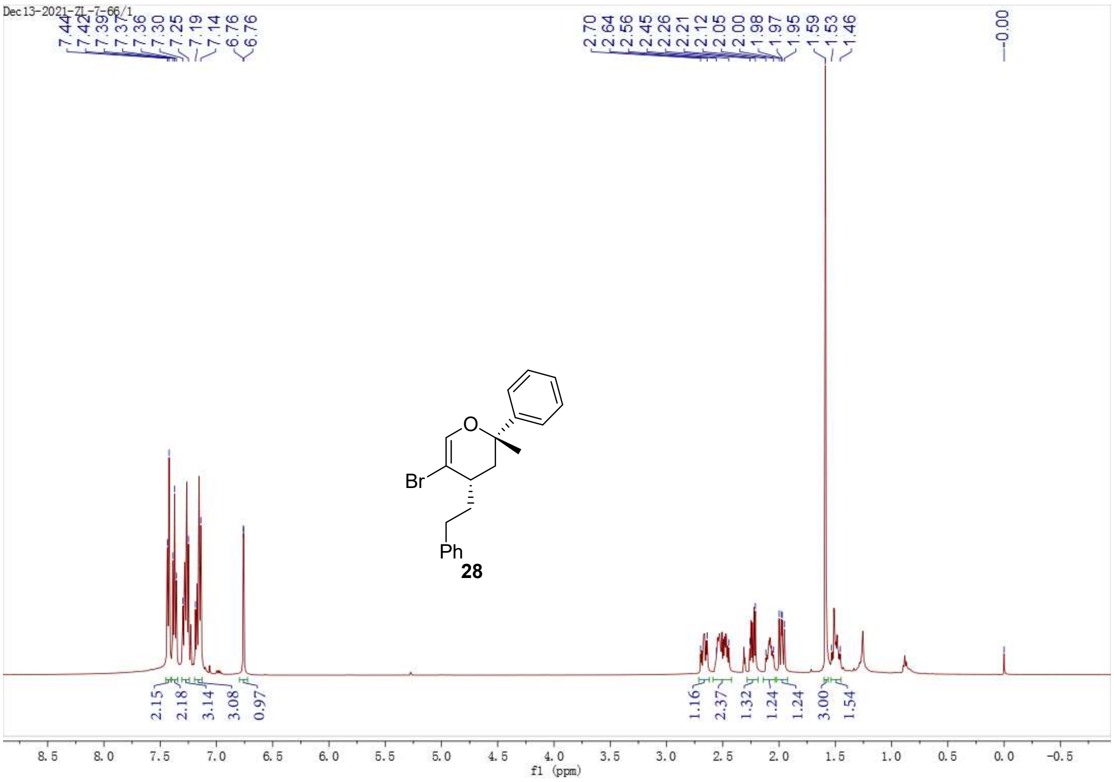

Supplementary Fig. 62. ^{1}H NMR spectrum of **28** ($CDCl_3$, 500 MHz)

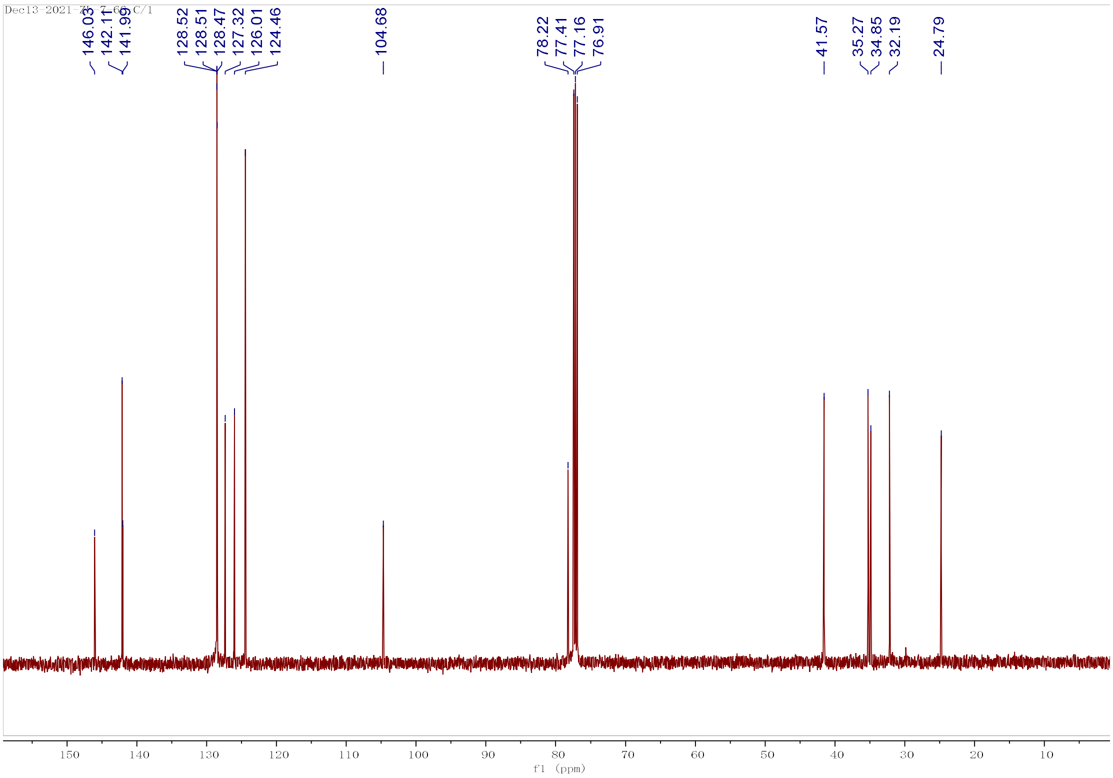

Supplementary Fig. 63. ^{13}C NMR spectrum of **28** ($CDCl_3$, 126 MHz)

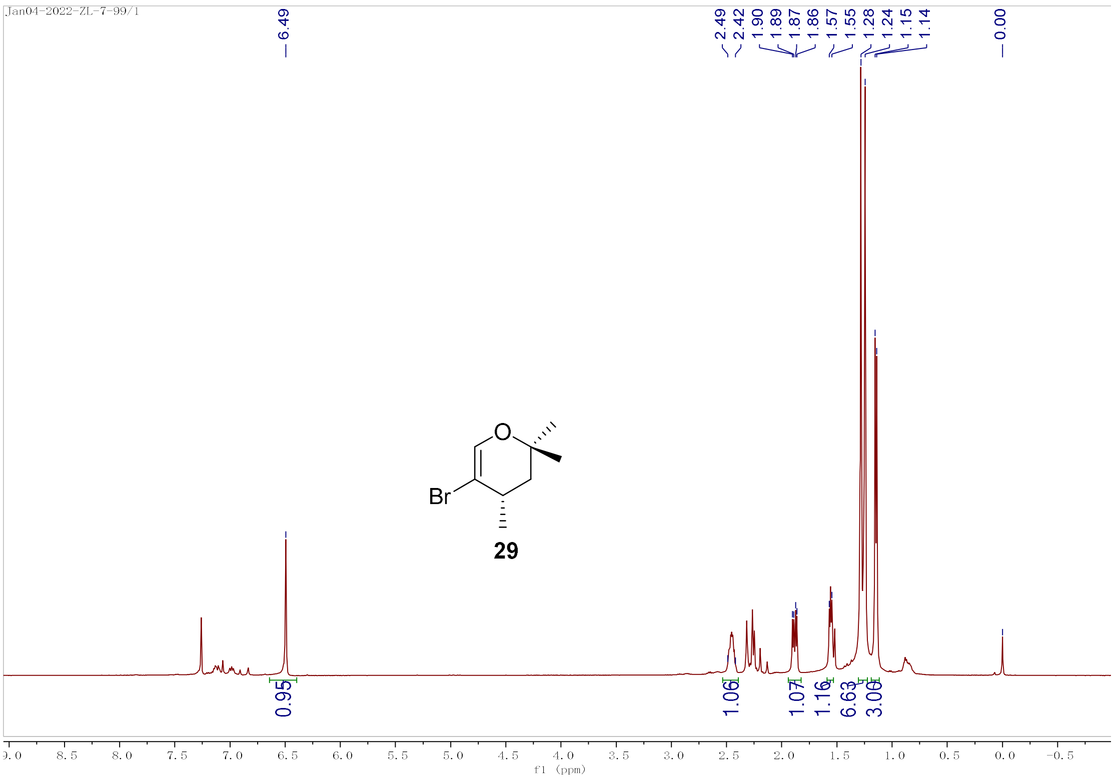

Supplementary Fig. 64. ^{1}H NMR spectrum of **29** ($CDCl_3$, 500 MHz)

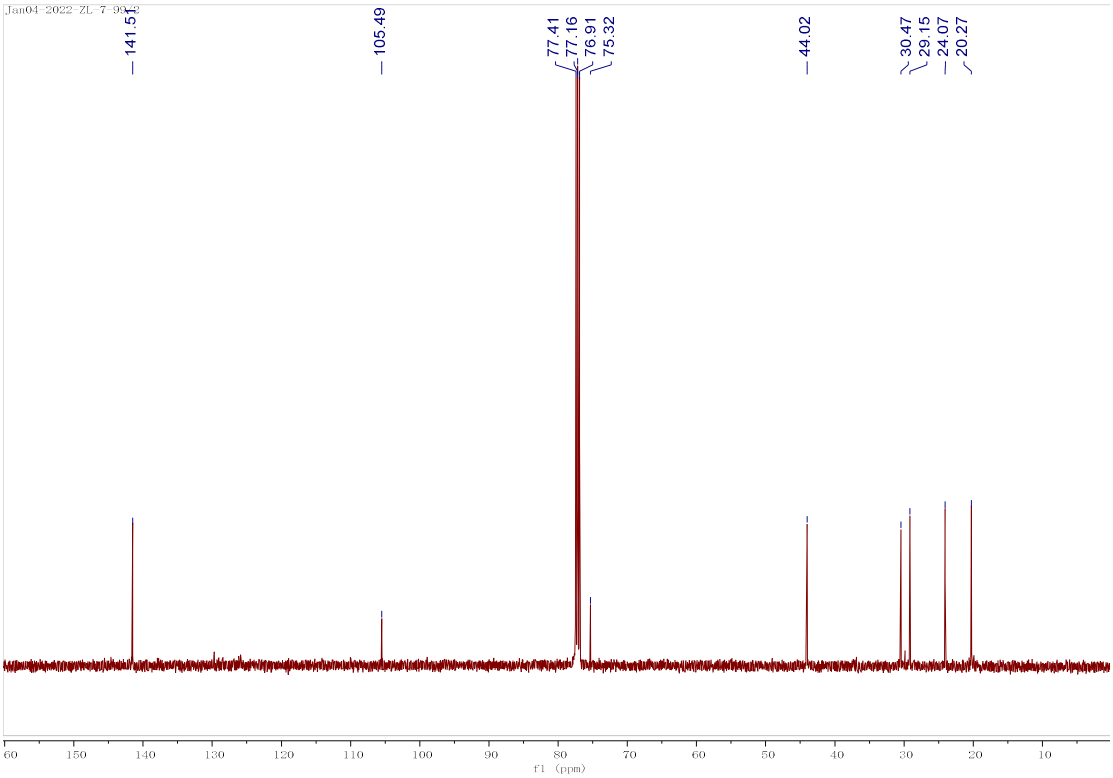

Supplementary Fig. 65. ^{13}C NMR spectrum of **29** ($CDCl_3$, 126 MHz)

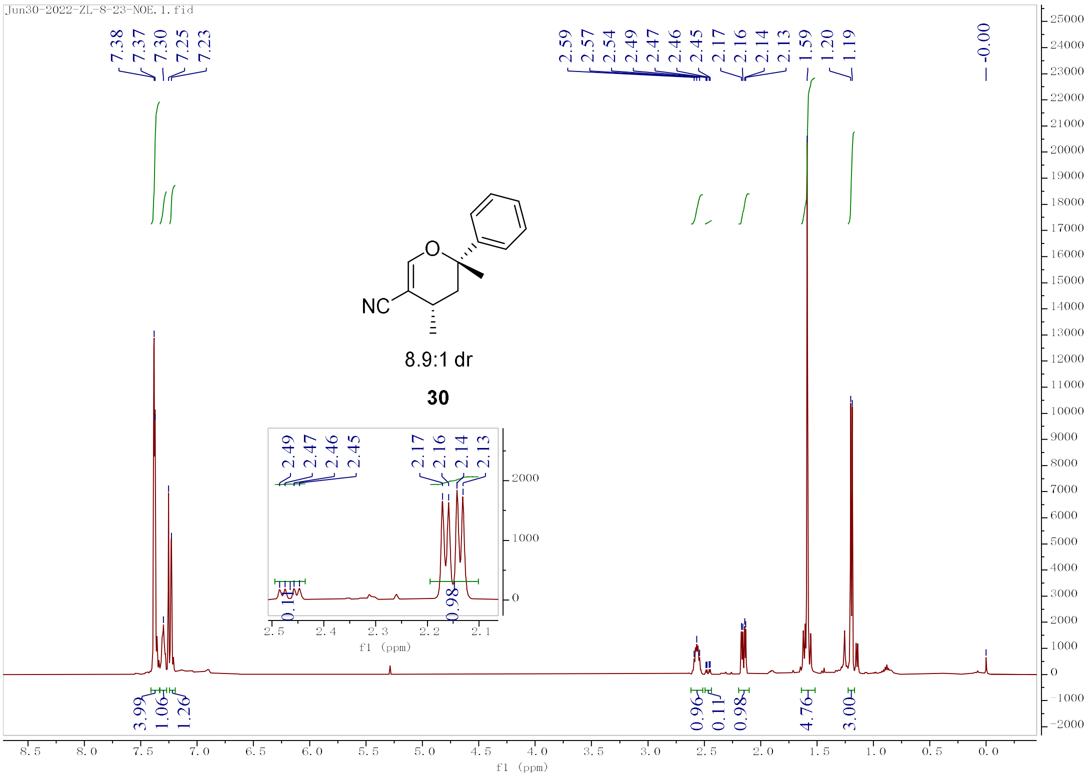

Supplementary Fig. 66. ^{1}H NMR spectrum of **30** ($CDCl_3$, 500 MHz)

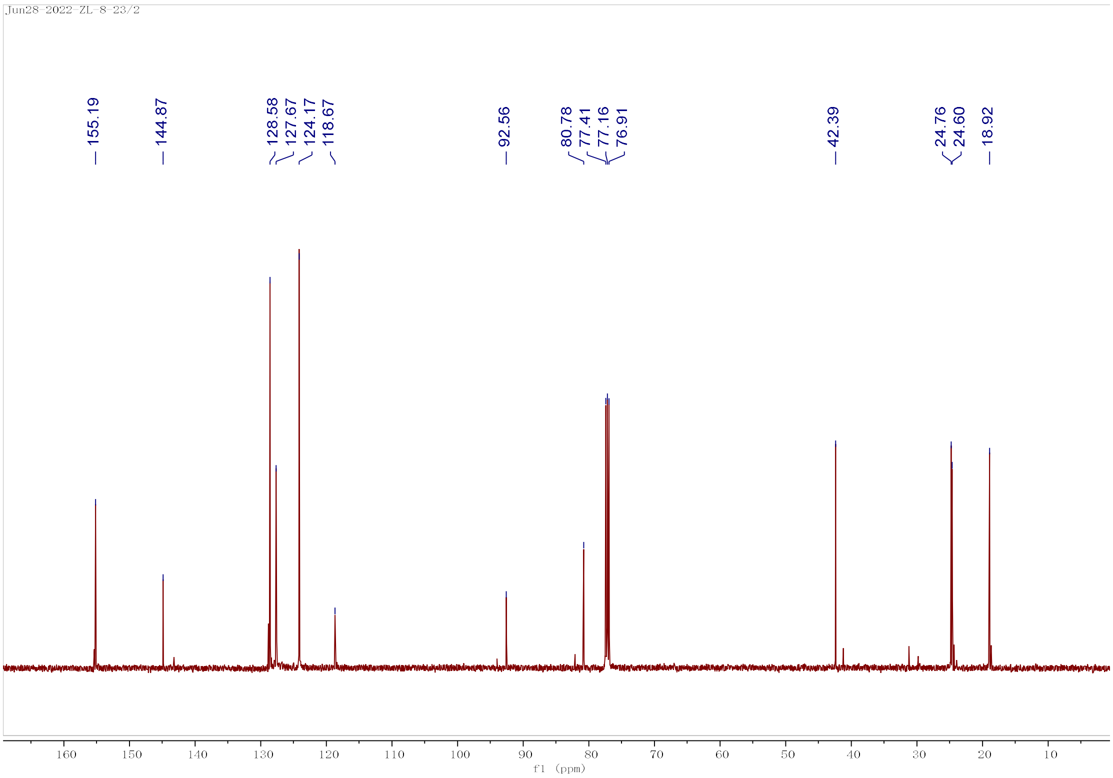

Supplementary Fig. 67. ^{13}C NMR spectrum of **30** ($CDCl_3$, 126 MHz)

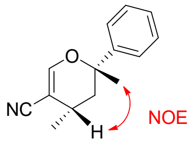

30

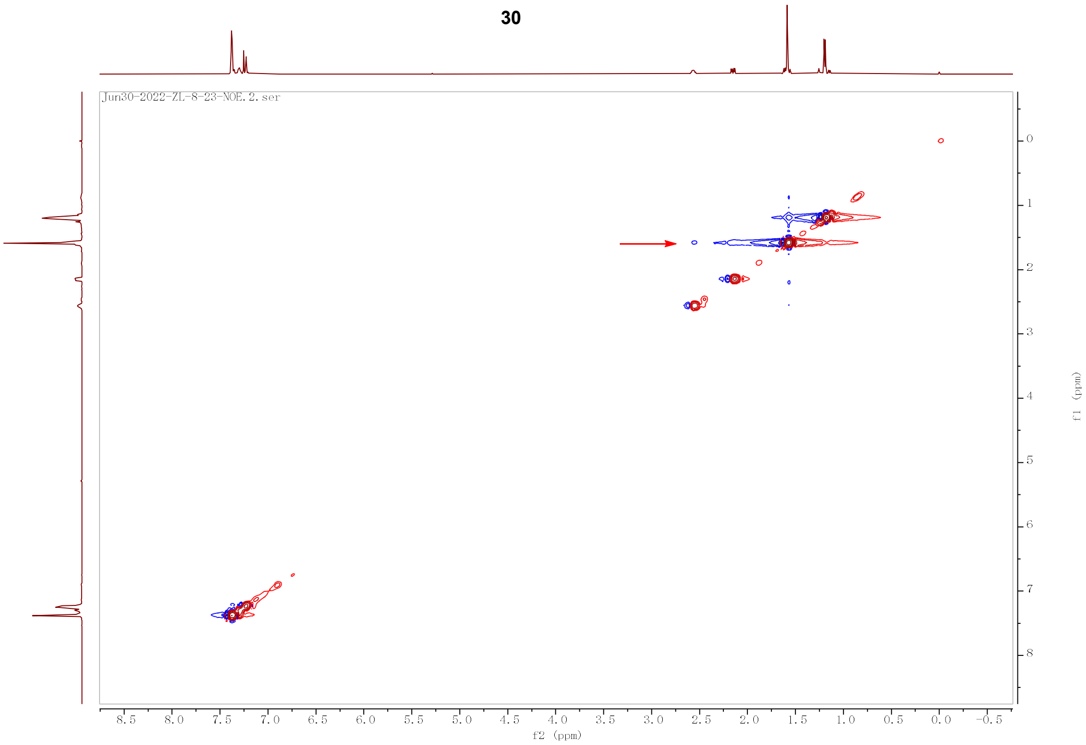

Supplementary Fig. 68. NOE spectrum of **30** ($CDCl_3$, 500 MHz)

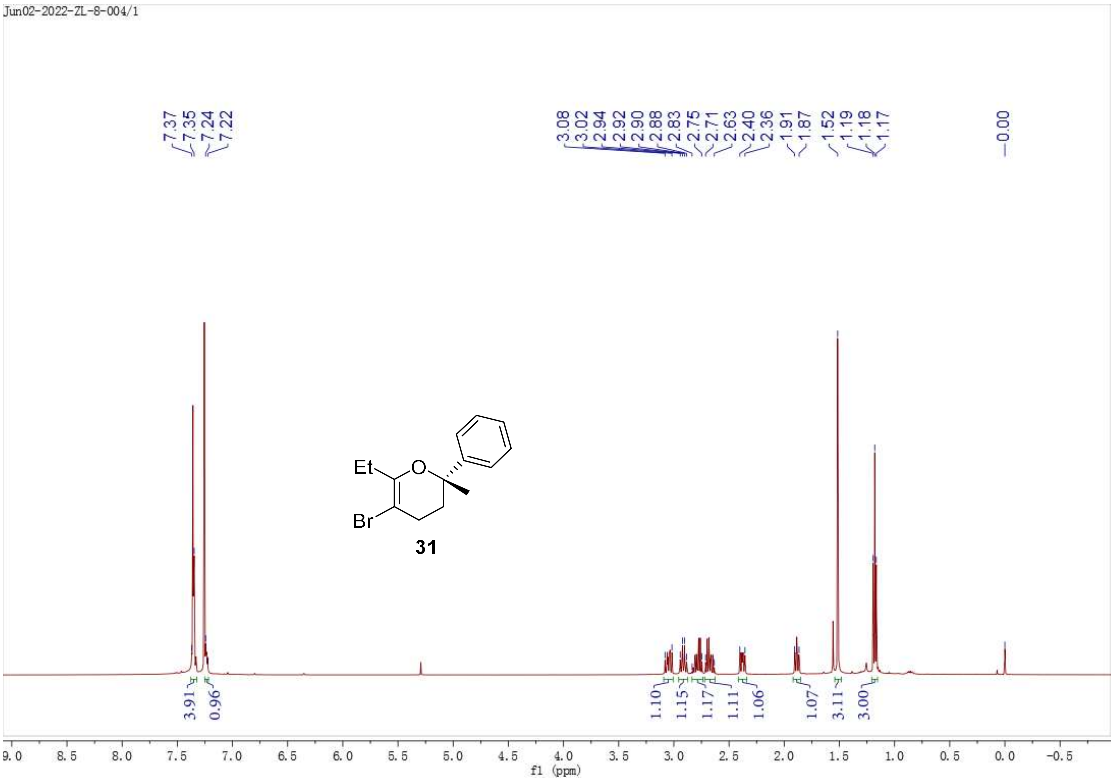

Supplementary Fig. 69. ^{1}H NMR spectrum of **31** ($CDCl_3$, 500 MHz)

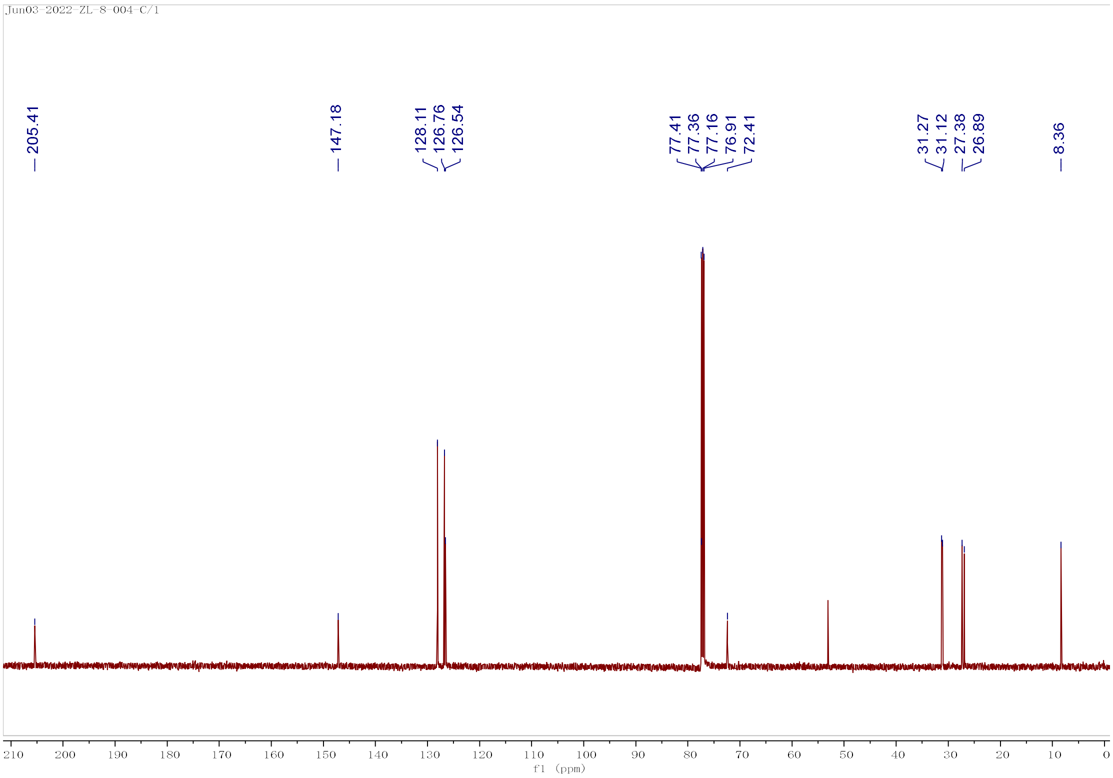

Supplementary Fig. 70. ^{13}C NMR spectrum of **31** ($CDCl_3$, 126 MHz)

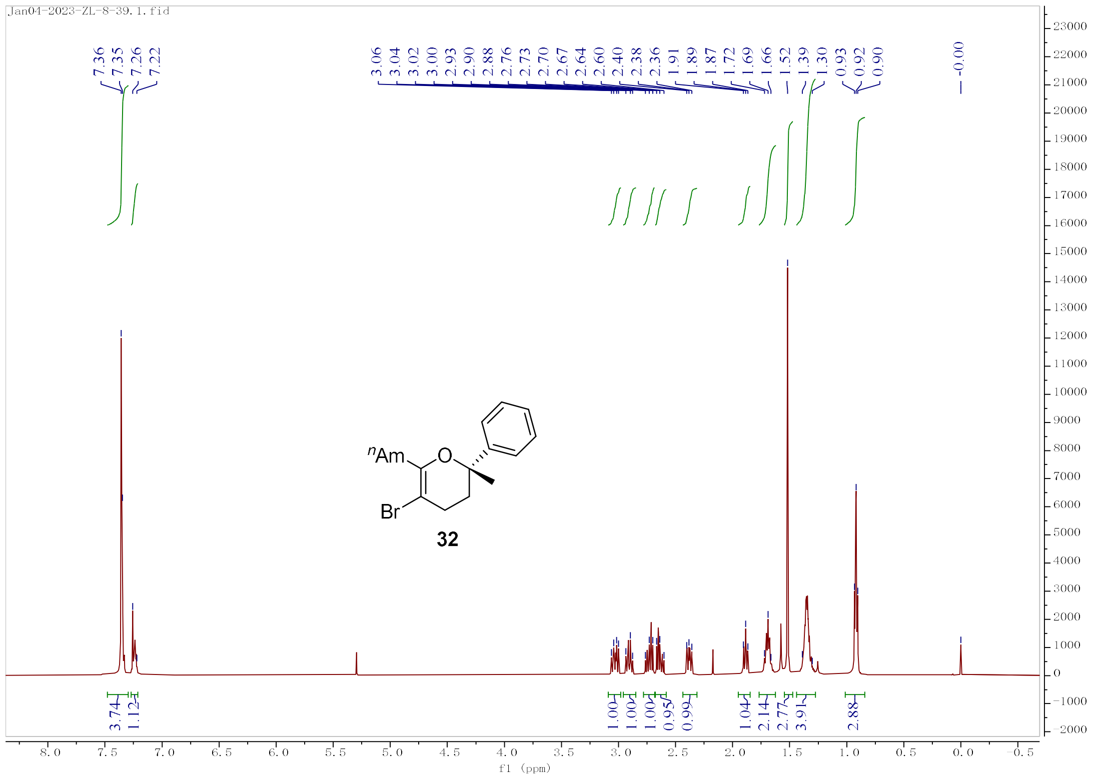

Supplementary Fig. 71. ^{1}H NMR spectrum of **32** ($CDCl_3$, 500 MHz)

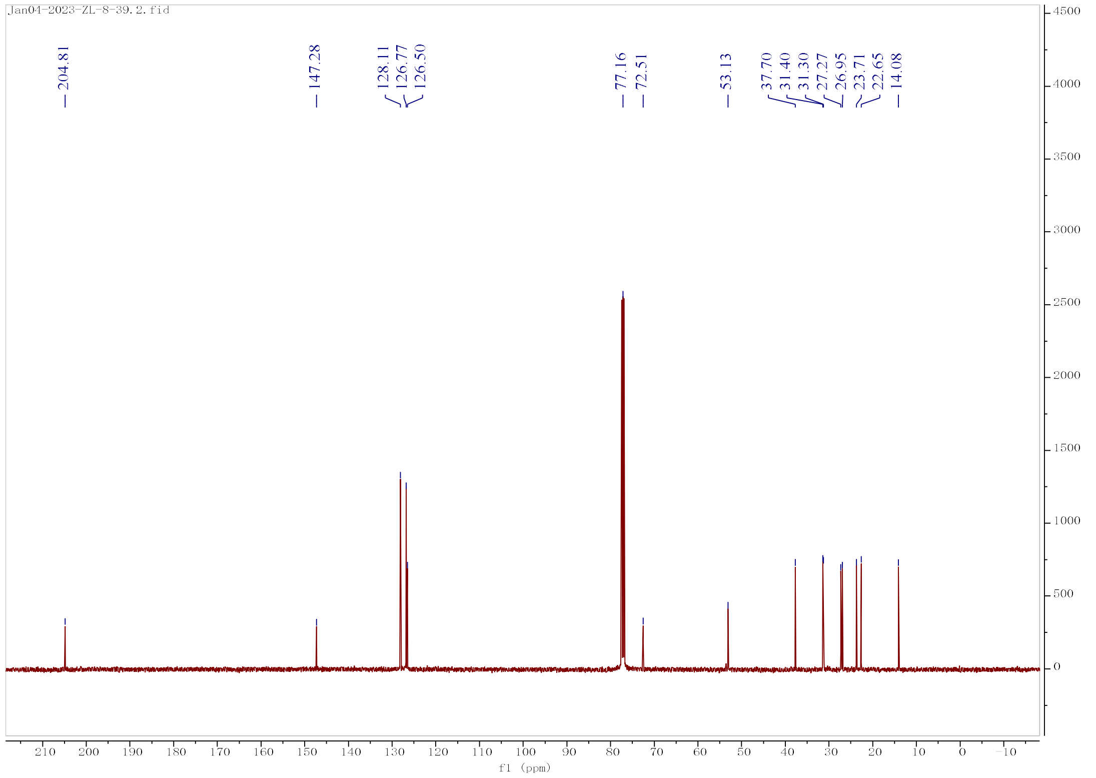

Supplementary Fig. 72. ^{13}C NMR spectrum of **32** ($CDCl_3$, 126 MHz)

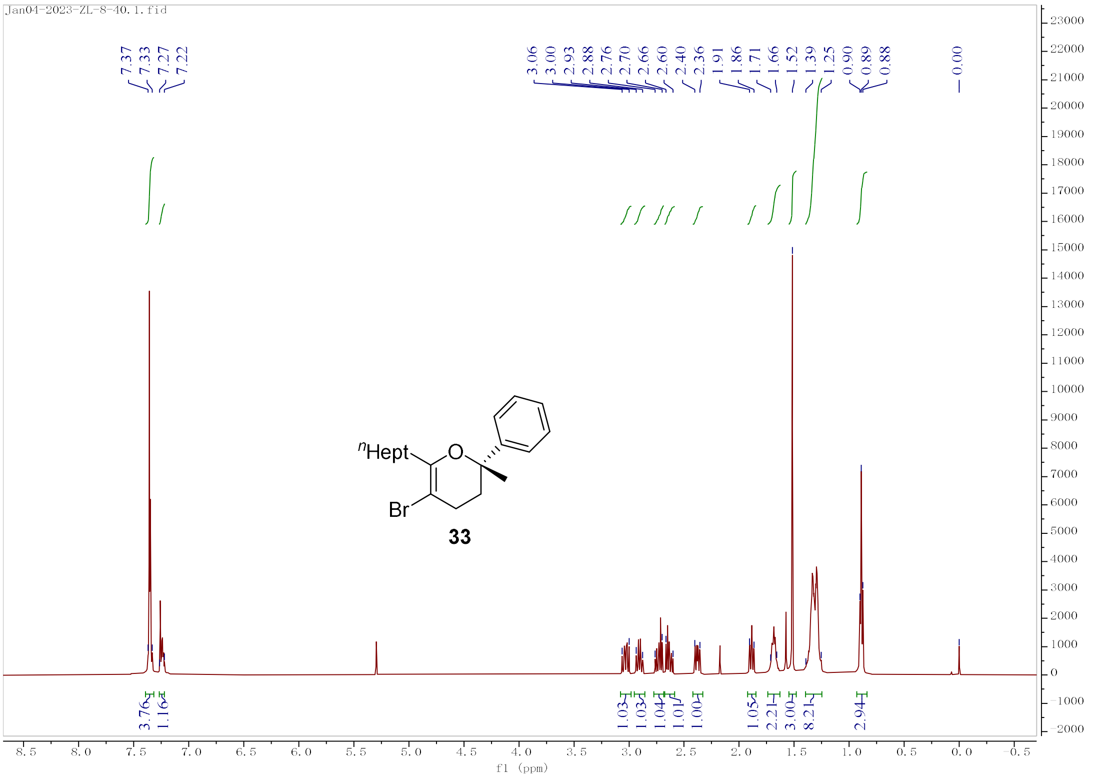

Supplementary Fig. 73. ^{1}H NMR spectrum of **33** ($CDCl_3$, 500 MHz)

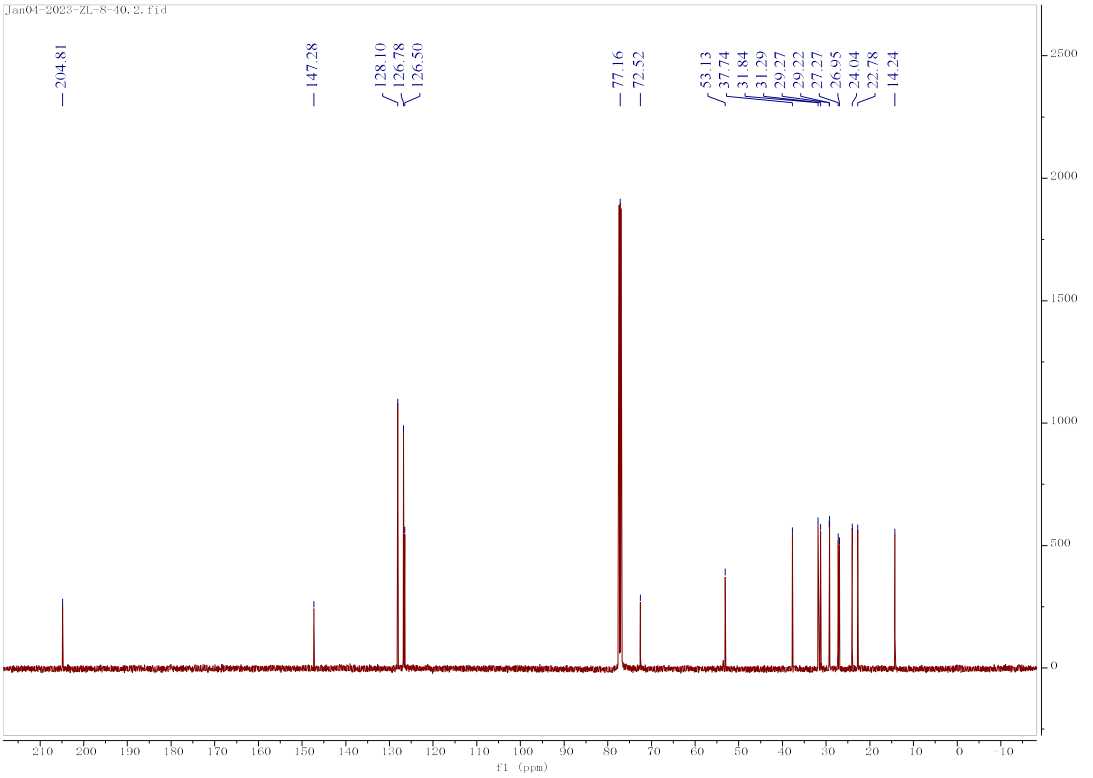

Supplementary Fig. 74. ^{13}C NMR spectrum of **33** ($CDCl_3$, 126 MHz)

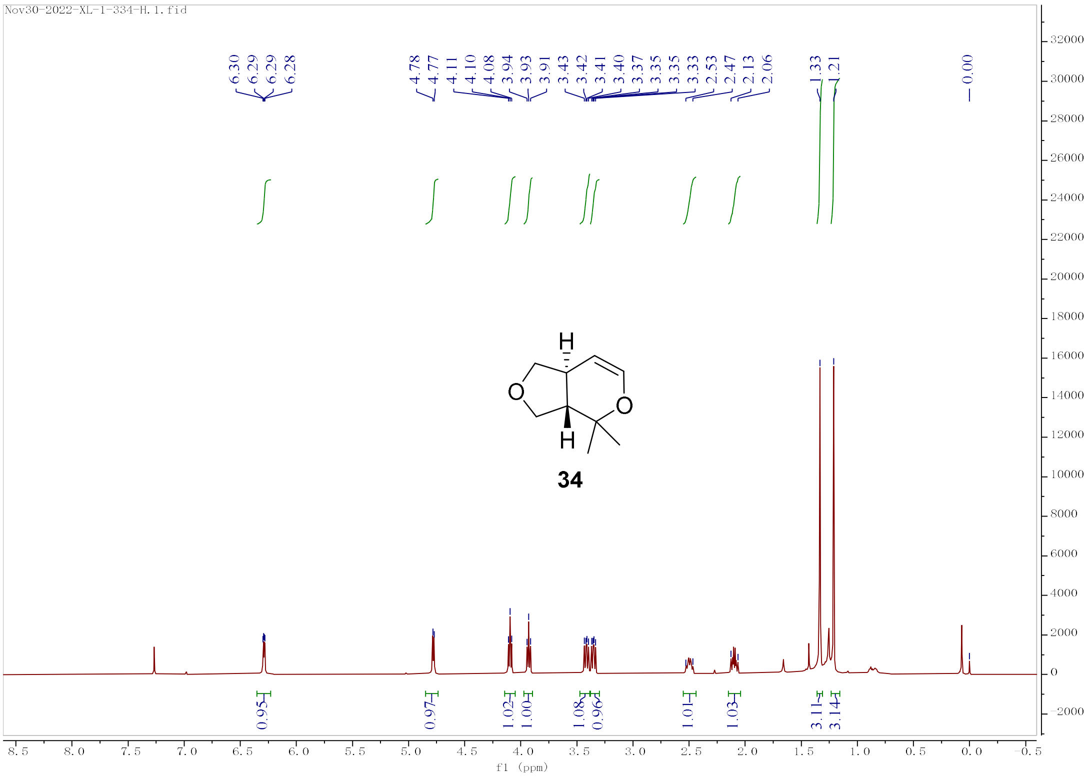

Supplementary Fig. 75. ^{1}H NMR spectrum of **34** ($CDCl_3$, 500 MHz)

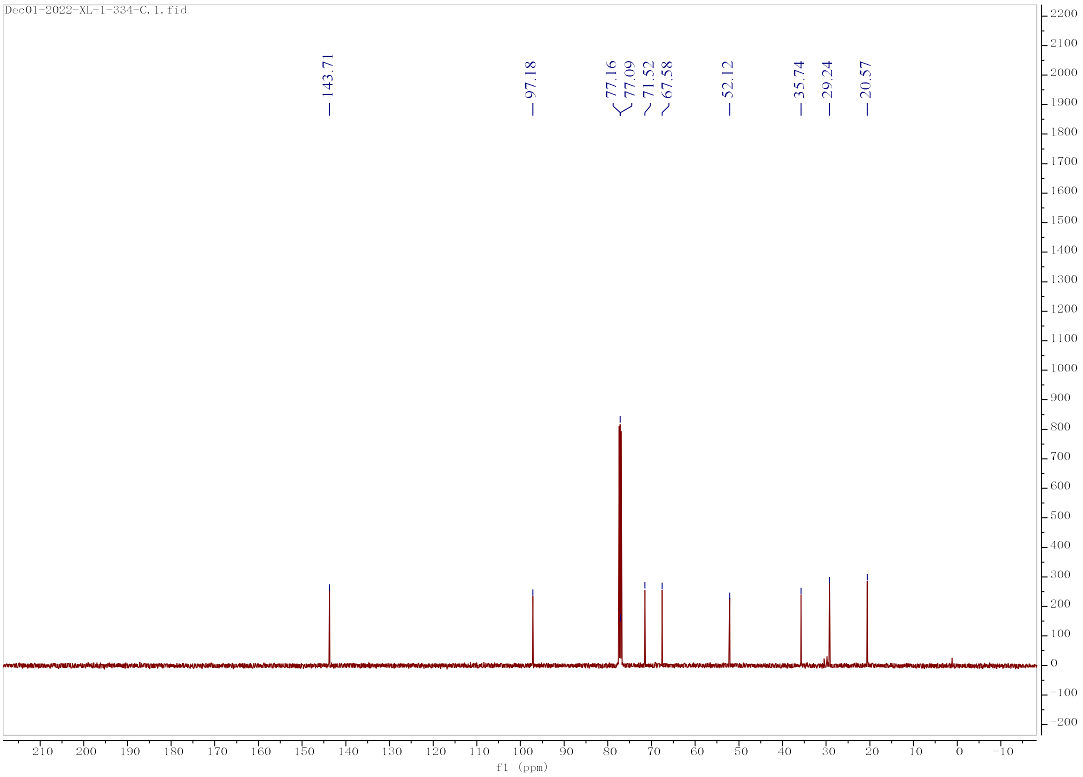

Supplementary Fig. 76. ^{13}C NMR spectrum of **34** ($CDCl_3$, 126 MHz)

Configurational assignment of the ring junction:

For the compound **34**, the 1H signals at 2.1 and 2.5 ppm should be these two protons at the ring junctions. Both protons have two large *J* values more than 10 Hz, which are typical axil/axil coupling constants. So compound **34** is a *trans* compound.

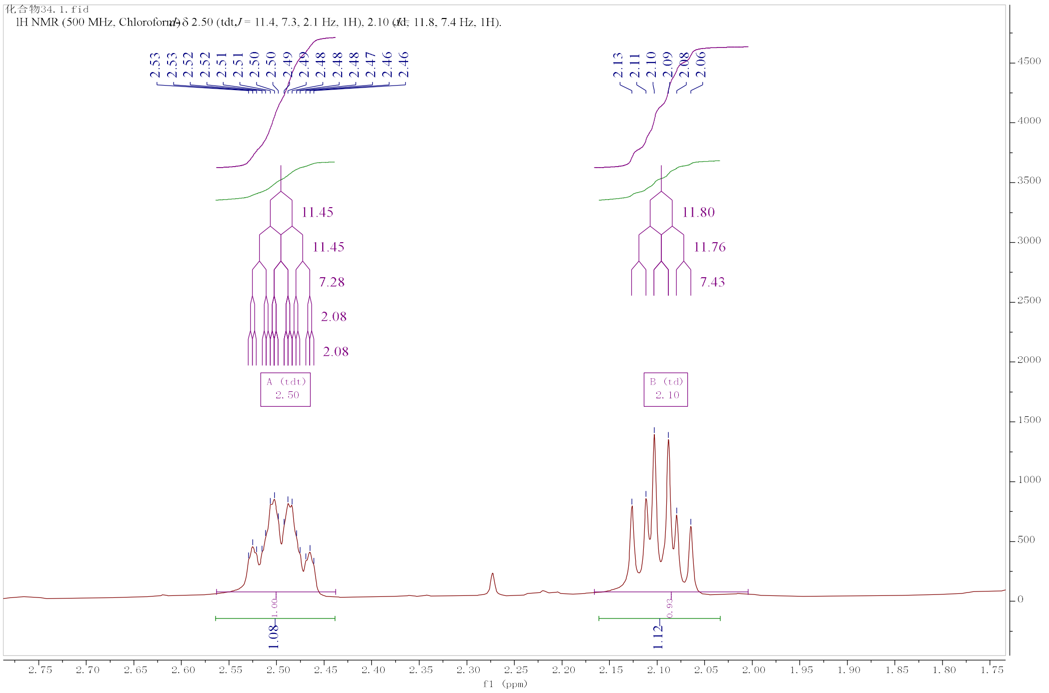

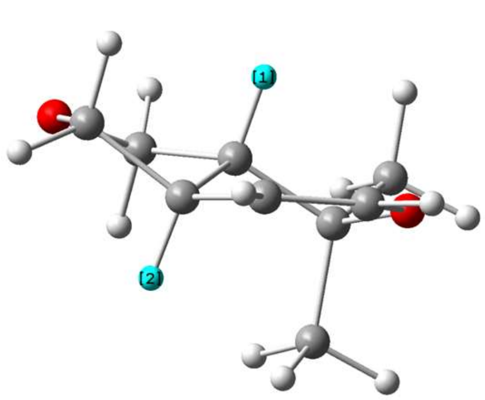

trans-**34**

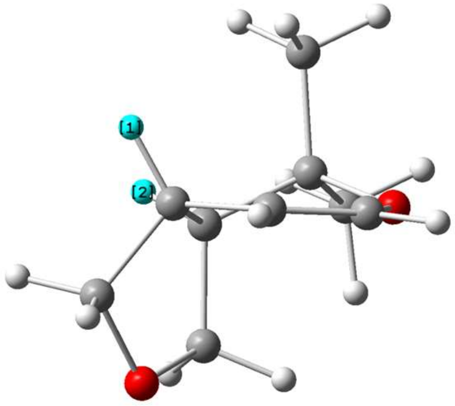

cis-**34**

Supplementary Fig. 77. Partial ^{1}H NMR spectrum and the molecular model of *trans*/*cis* **34**

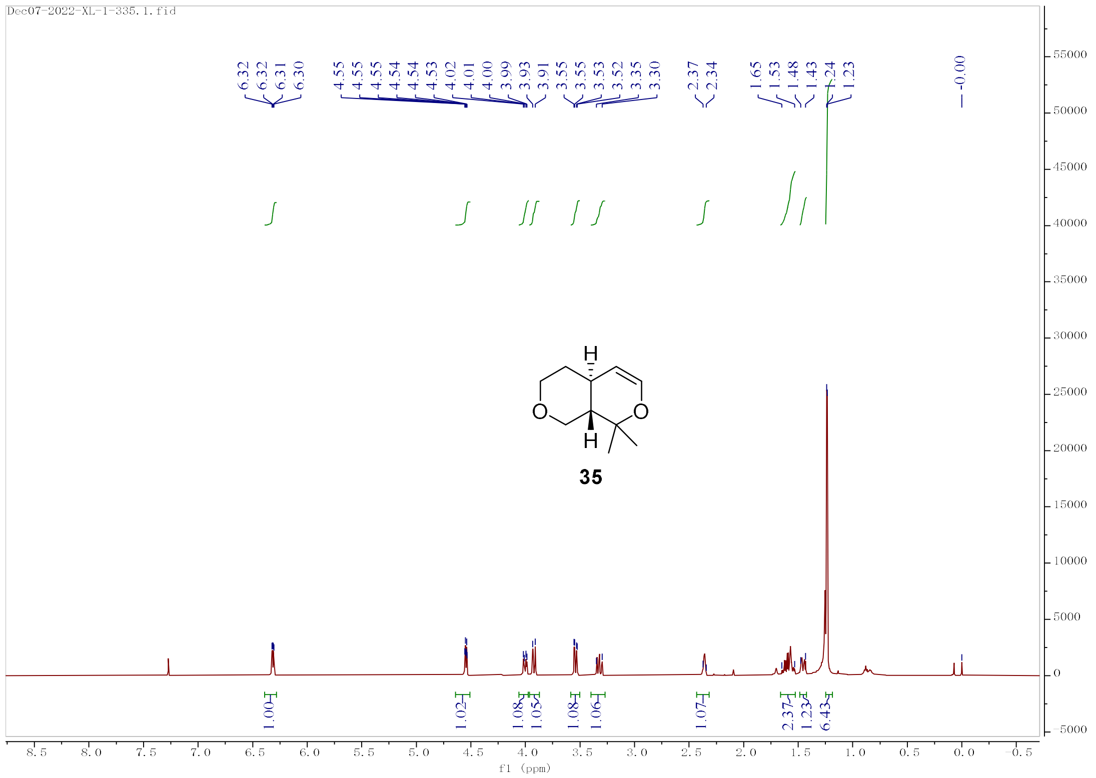

Supplementary Fig. 78. ^{1}H NMR spectrum of **35** ($CDCl_3$, 500 MHz)

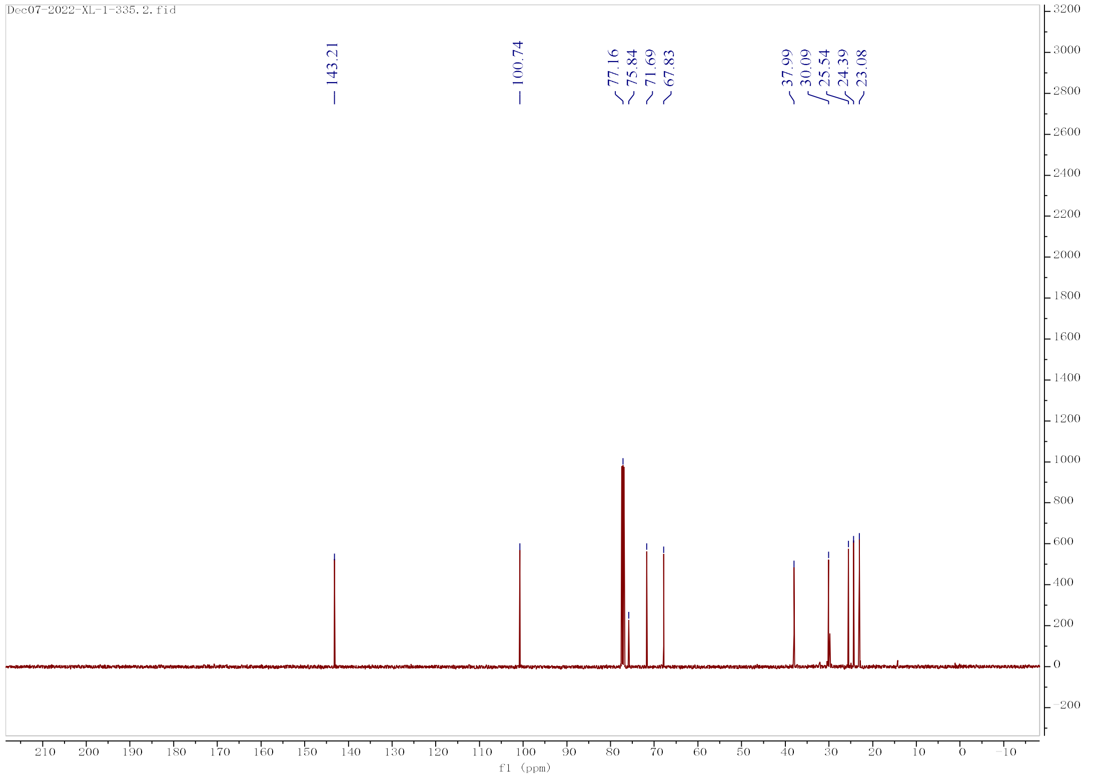

Supplementary Fig. 79. ^{13}C NMR spectrum of **35** ($CDCl_3$, 126 MHz)

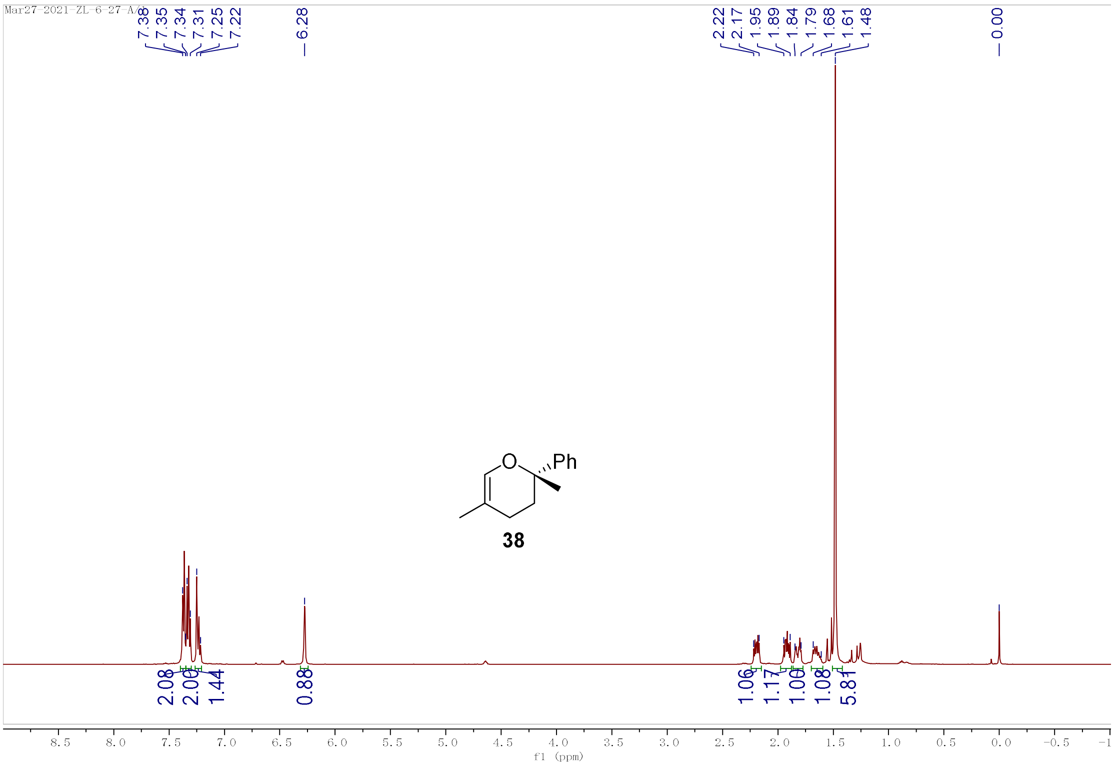

Supplementary Fig. 80. ^{1}H NMR spectrum of **38** ($CDCl_3$, 500 MHz)

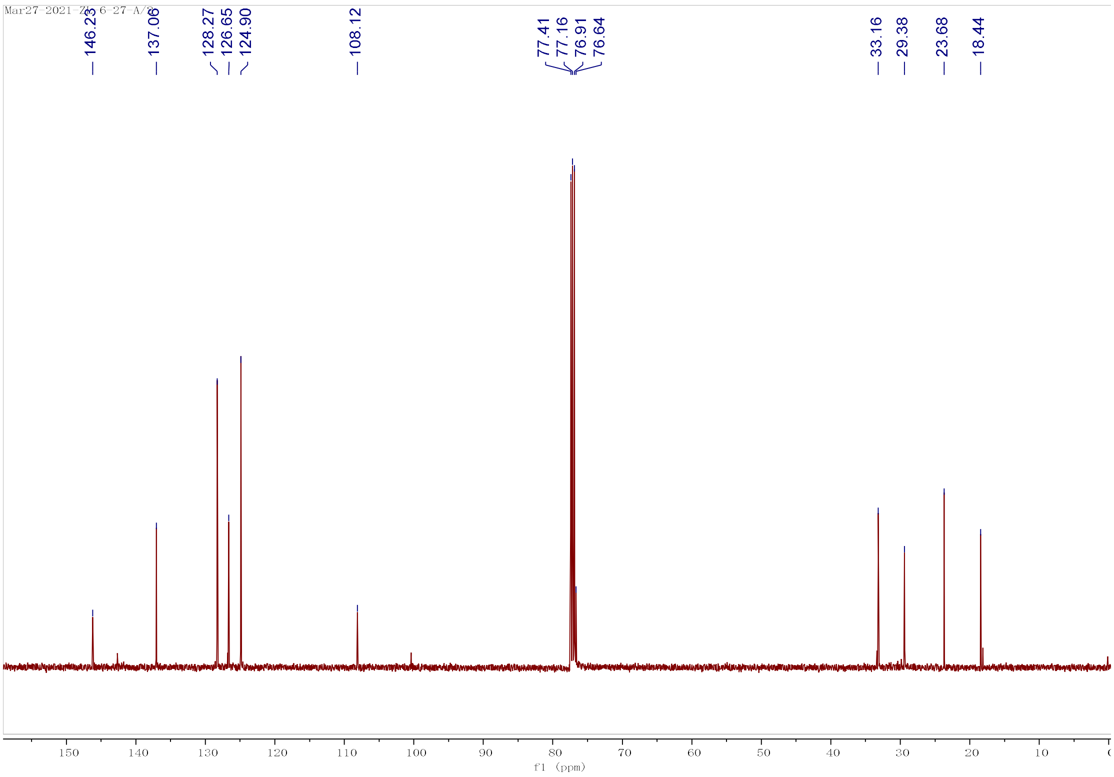

Supplementary Fig. 81. ^{13}C NMR spectrum of **38** ($CDCl_3$, 126 MHz)

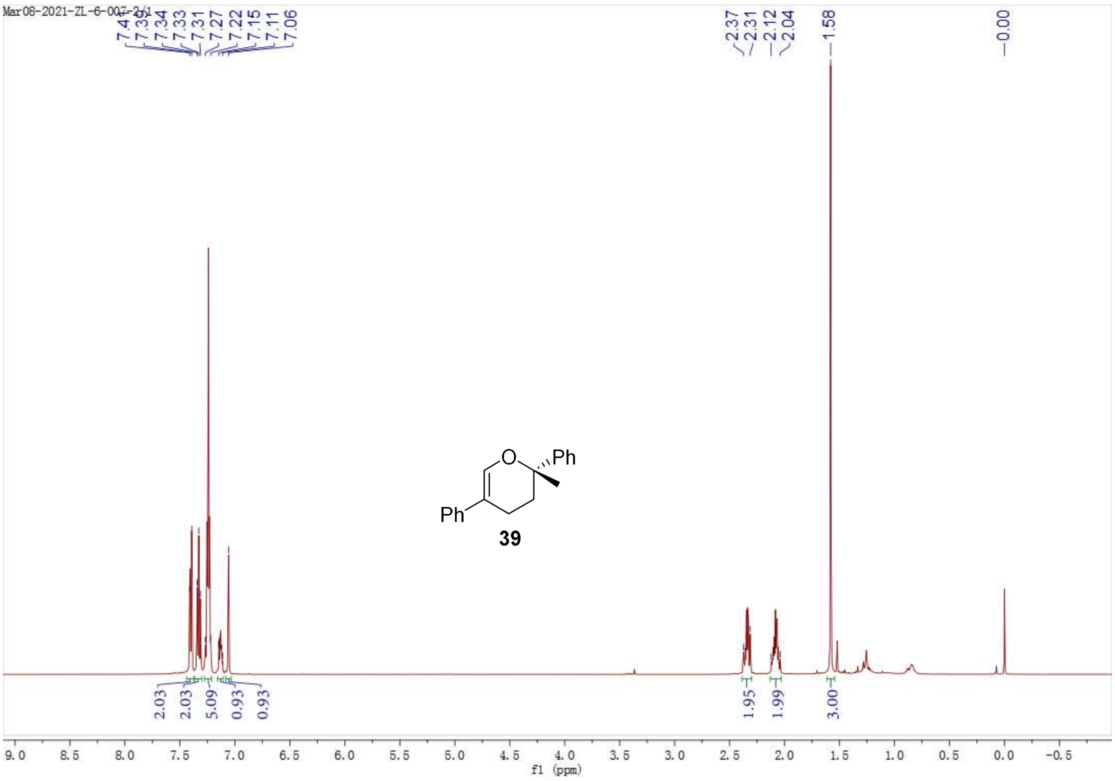

Supplementary Fig. 82. ^{1}H NMR spectrum of **39** ($CDCl_3$, 500 MHz)

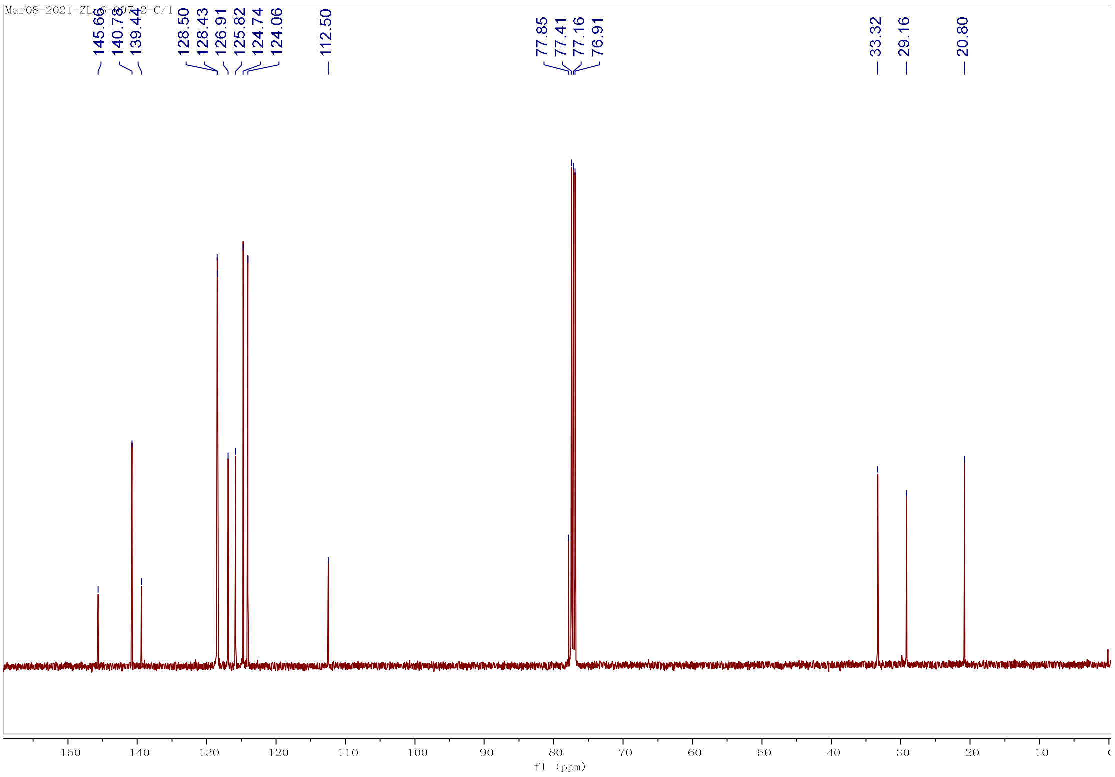

Supplementary Fig. 83. ^{13}C NMR spectrum of **39** ($CDCl_3$, 126 MHz)

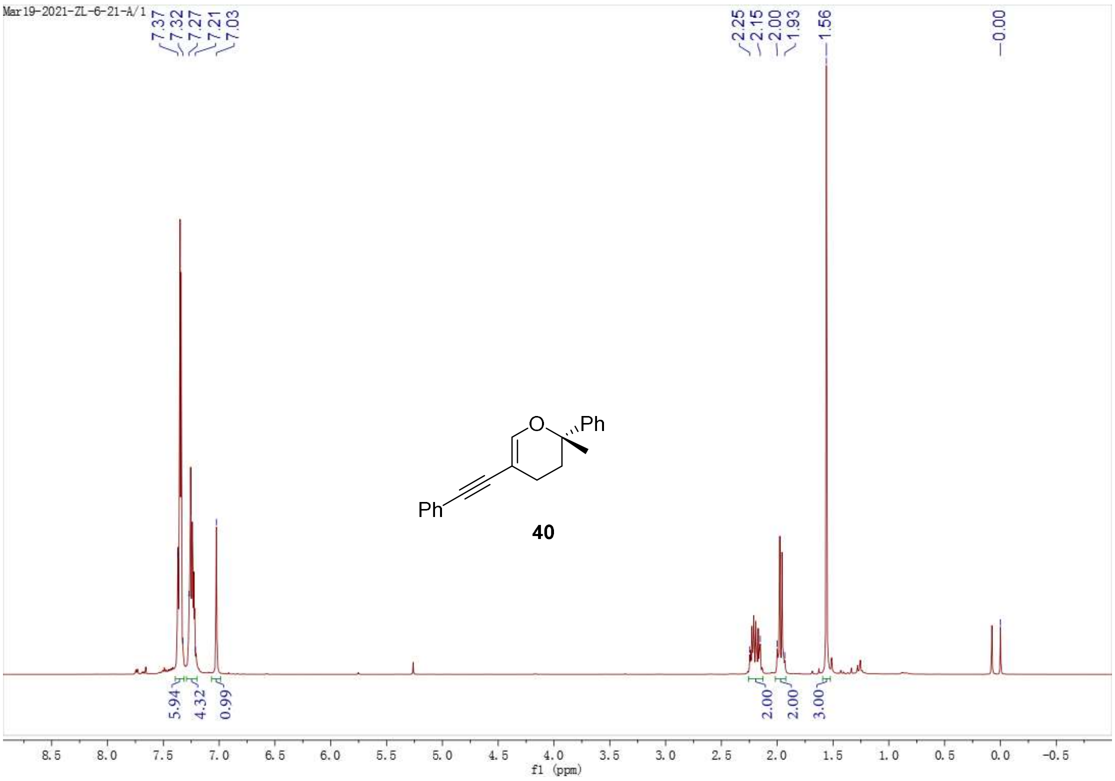

Supplementary Fig. 84. ^{1}H NMR spectrum of **40** ($CDCl_3$, 500 MHz)

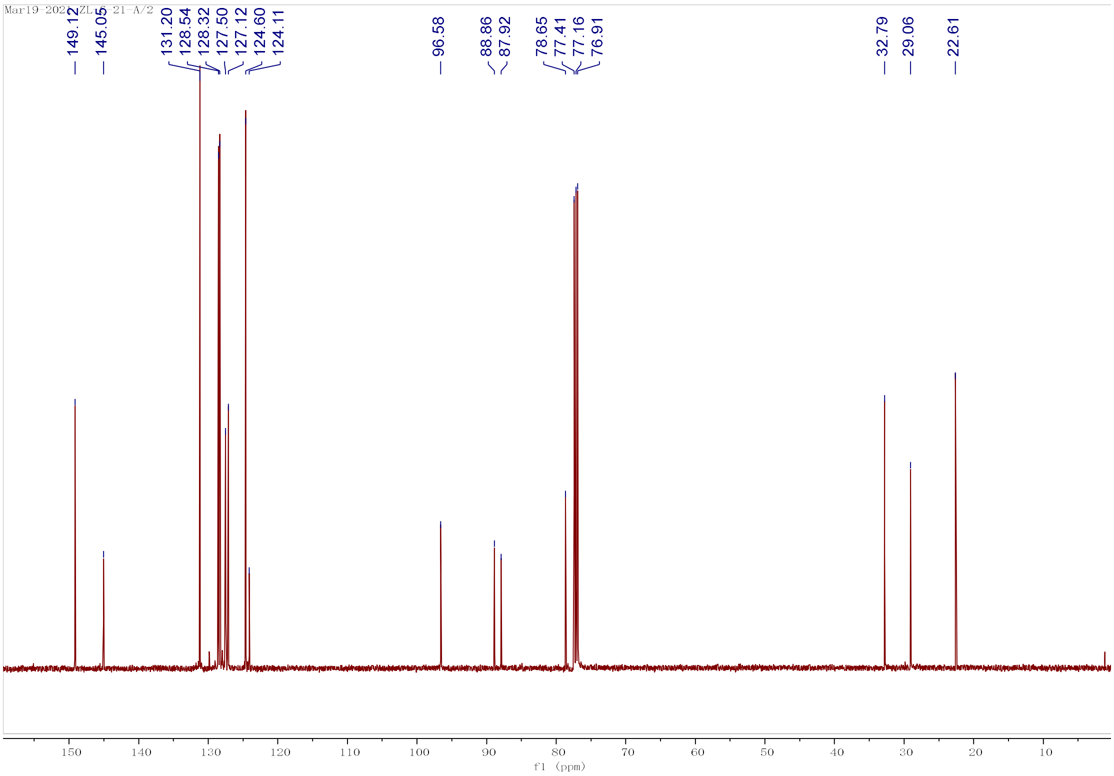

Supplementary Fig. 85. ^{13}C NMR spectrum of **40** ($CDCl_3$, 126 MHz)

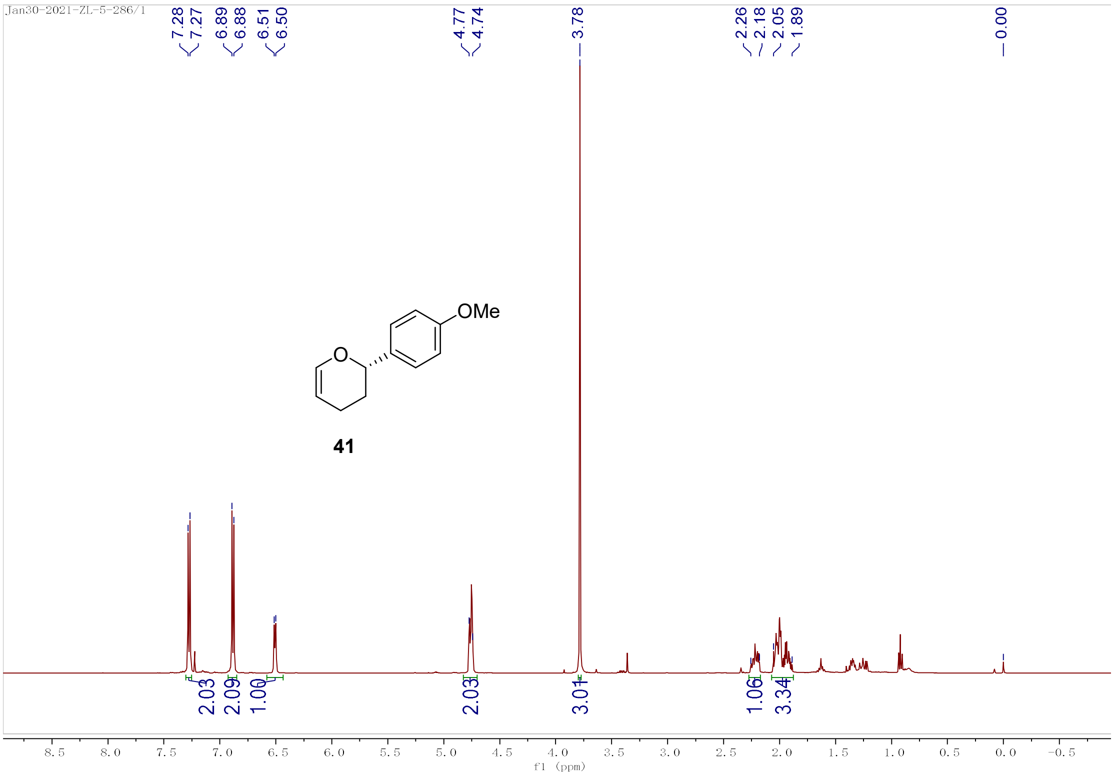

Supplementary Fig. 86. ^{1}H NMR spectrum of **41** ($CDCl_3$, 500 MHz)

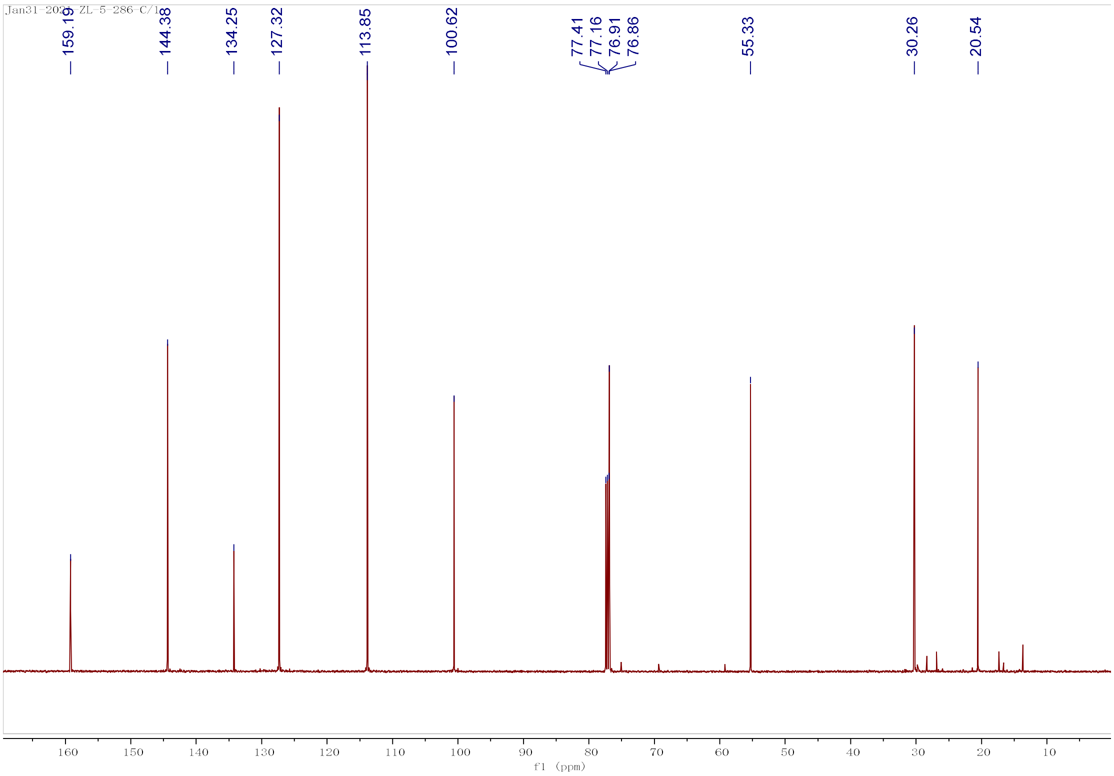

Supplementary Fig. 87. ^{13}C NMR spectrum of **41** ($CDCl_3$, 126 MHz)

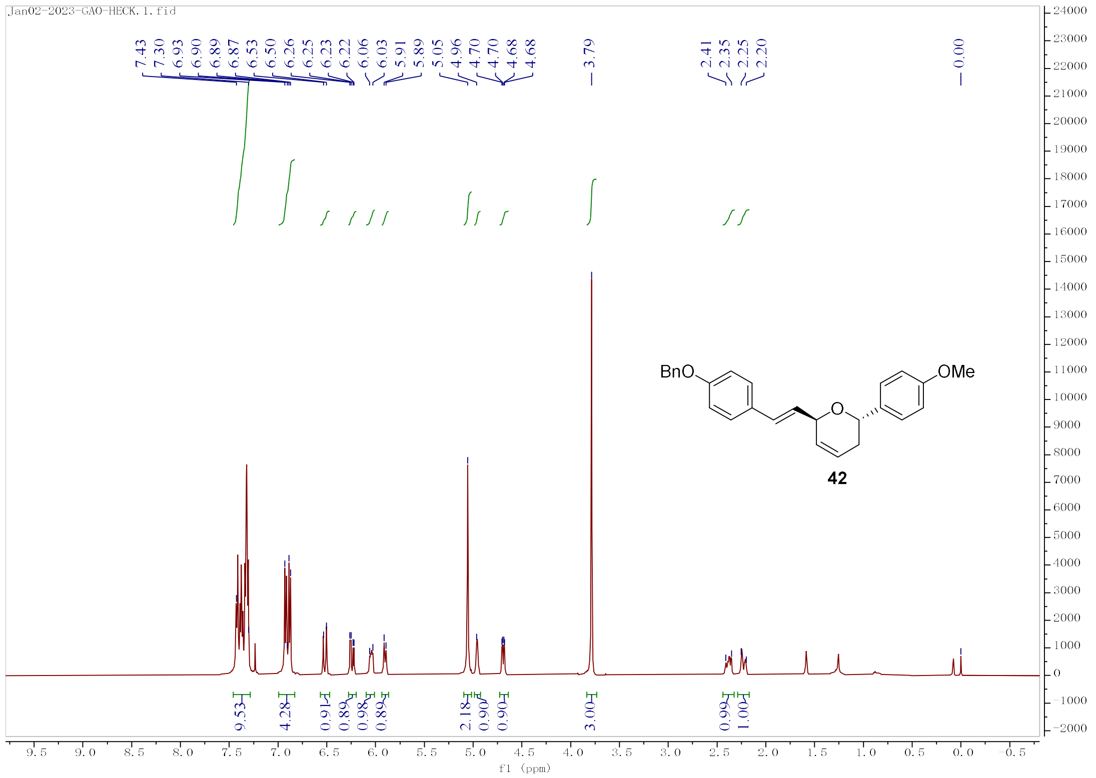

Supplementary Fig. 88. ^{1}H NMR spectrum of **42** ($CDCl_3$, 500 MHz)

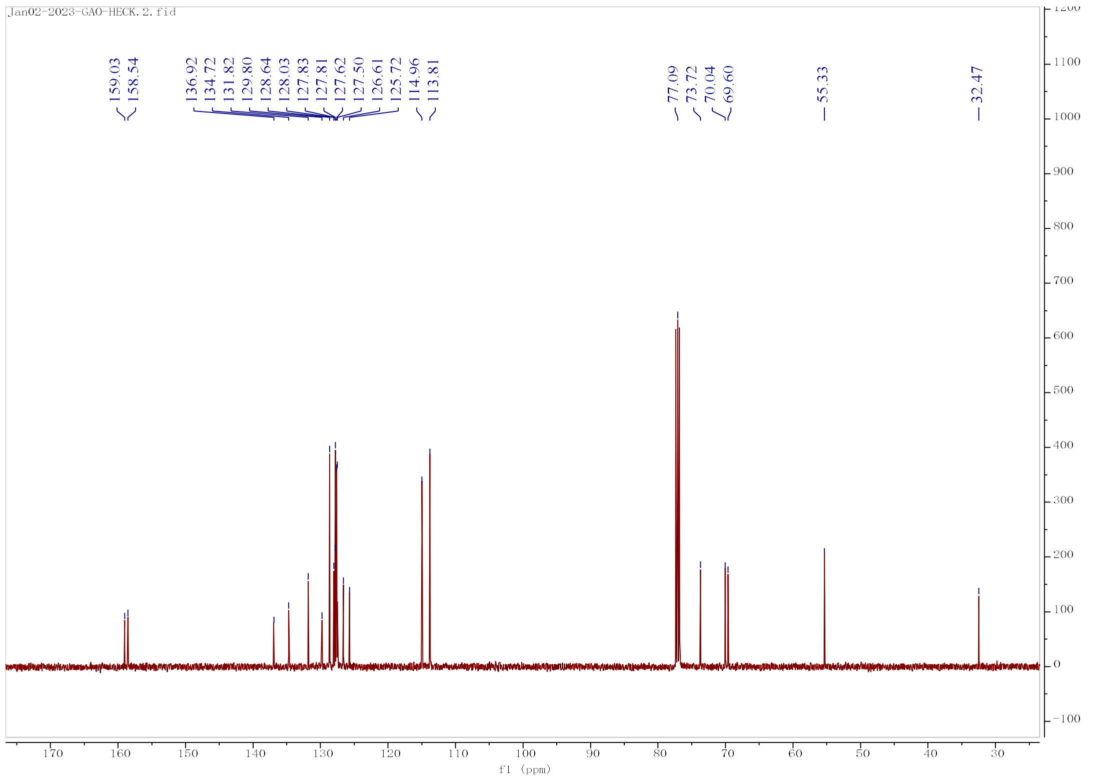

Supplementary Fig. 89. ^{13}C NMR spectrum of **42** ($CDCl_3$, 126 MHz)

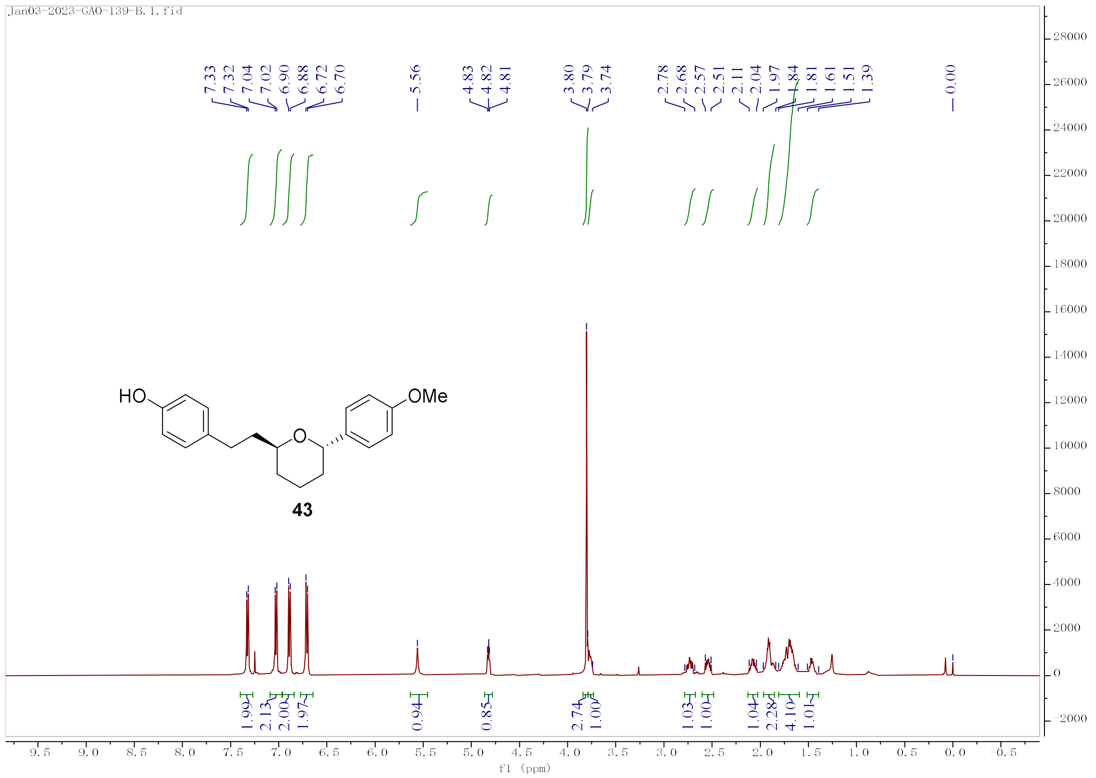

Supplementary Fig. 90. ^{1}H NMR spectrum of **43** ($CDCl_3$, 500 MHz)

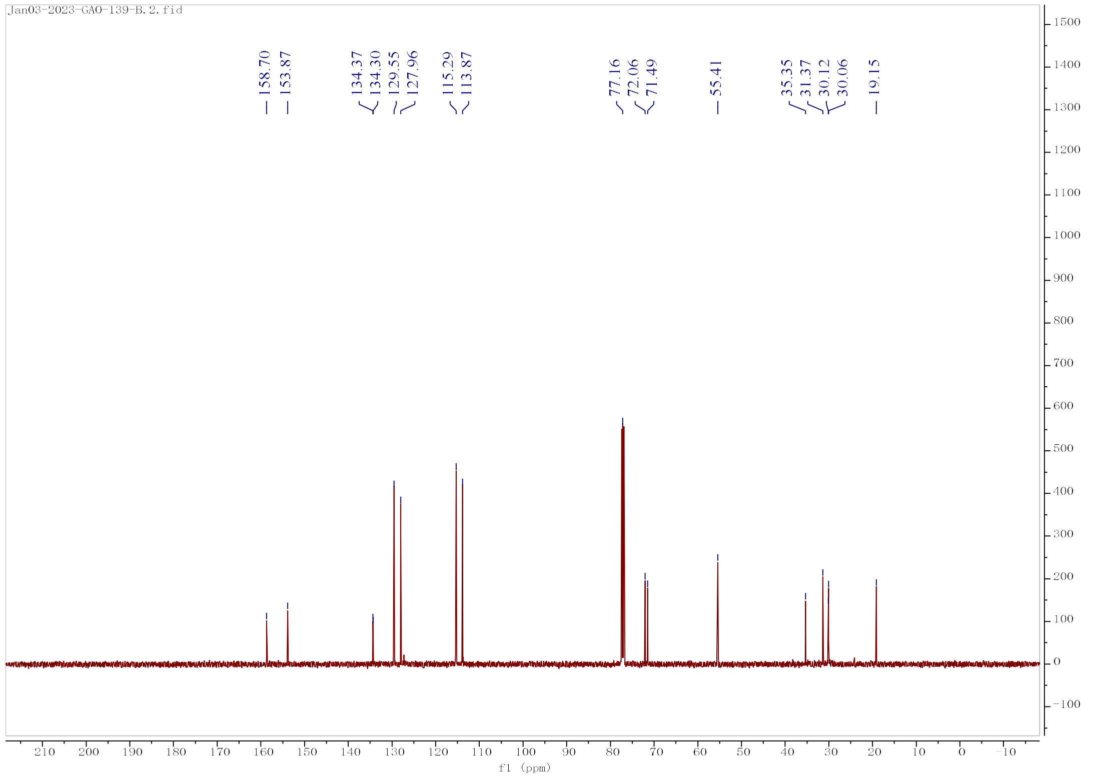

Supplementary Fig. 91. ^{13}C NMR spectrum of **43** ($CDCl_3$, 126 MHz)

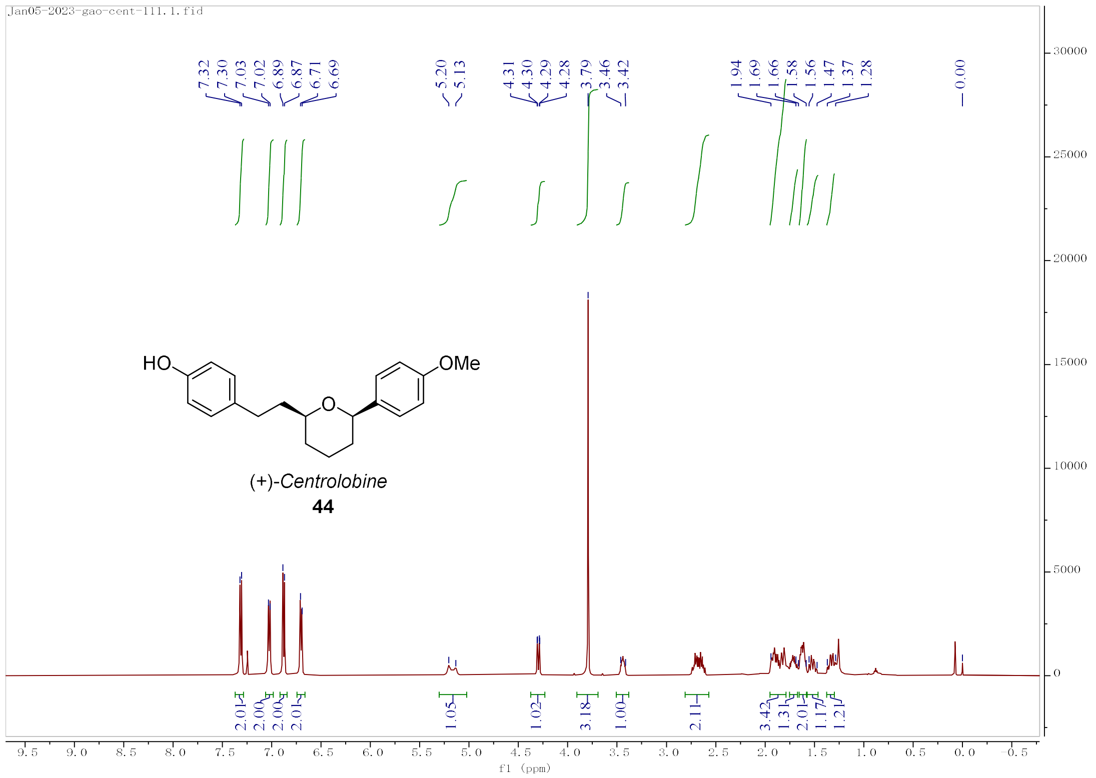

Supplementary Fig. 92. ^{1}H NMR spectrum of **44** ($CDCl_3$, 500 MHz)

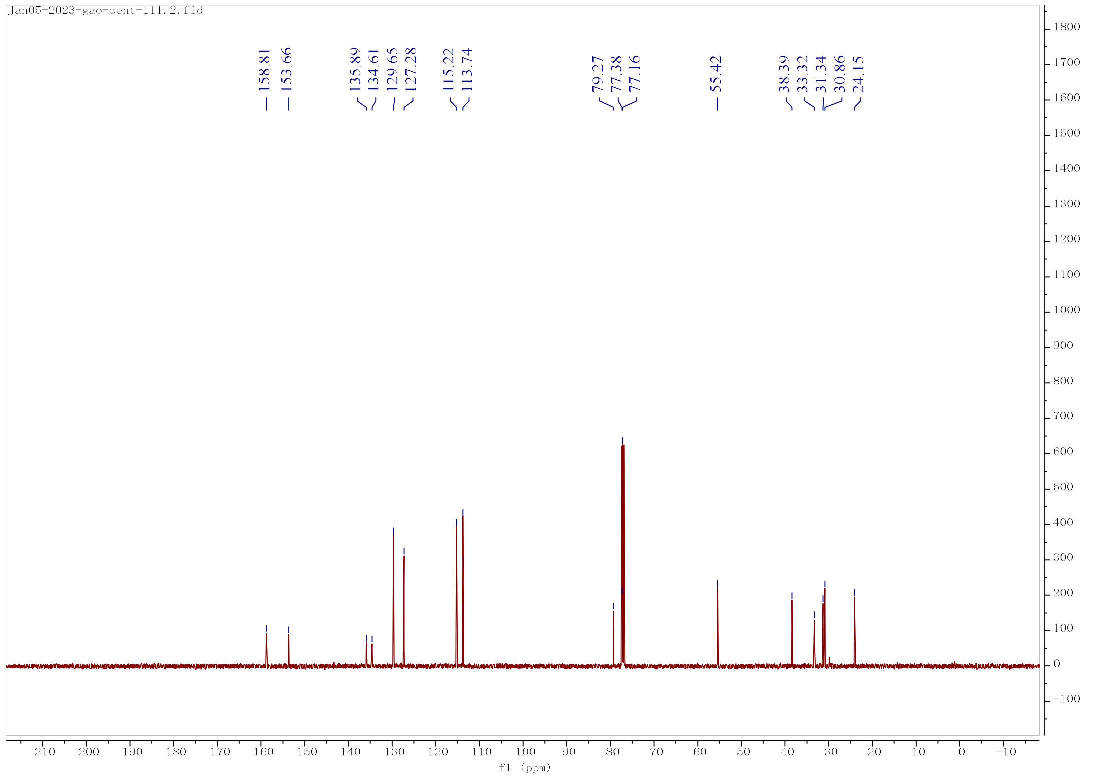

Supplementary Fig. 93. ^{13}C NMR spectrum of **44** ($CDCl_3$, 126 MHz)

HPLC Spectra

Supplementary Fig. 94. HPLC Spectra of **2**

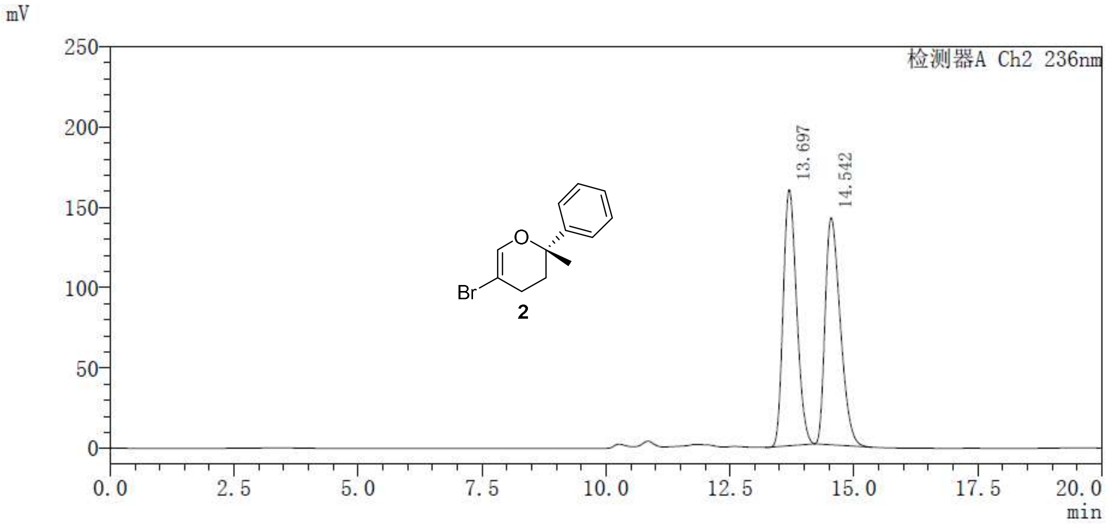

Peak	Retention Time (min)	Relative Area (%)
1	13.697	49.944
2	14.542	50.056

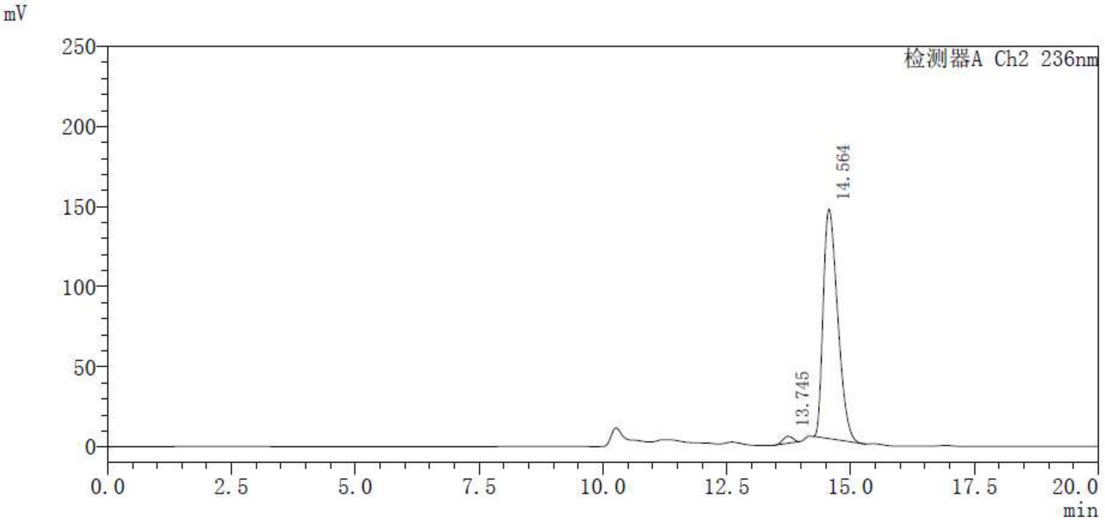

Peak	Retention Time (min)	Relative Area (%)	ee (%)
1	13.745	2.147	96
2	14.564	97.853	

Supplementary Fig. 95. HPLC Spectra of **4**

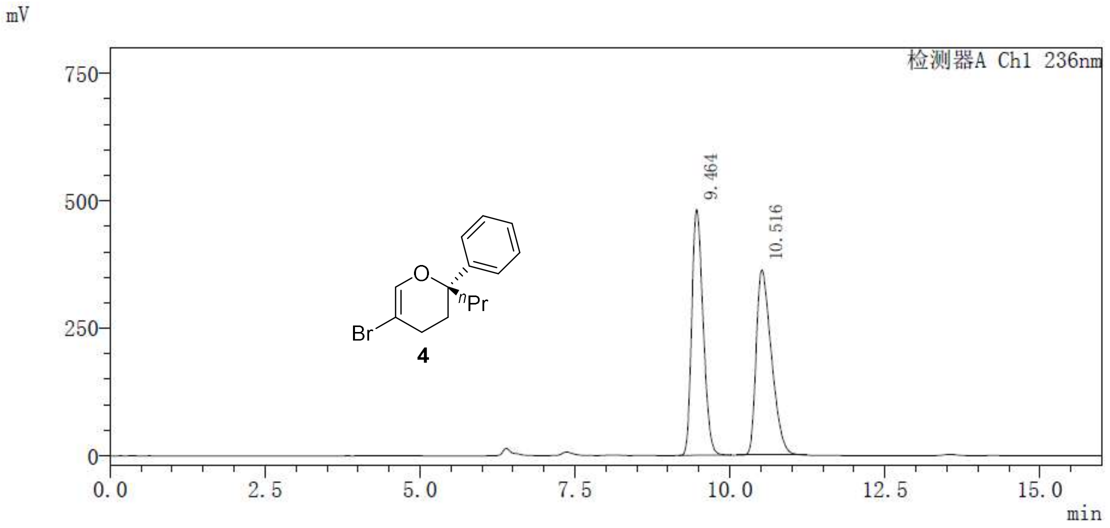

Peak	Retention Time (min)	Relative Area (%)
1	9.464	49.608
2	10.516	50.392

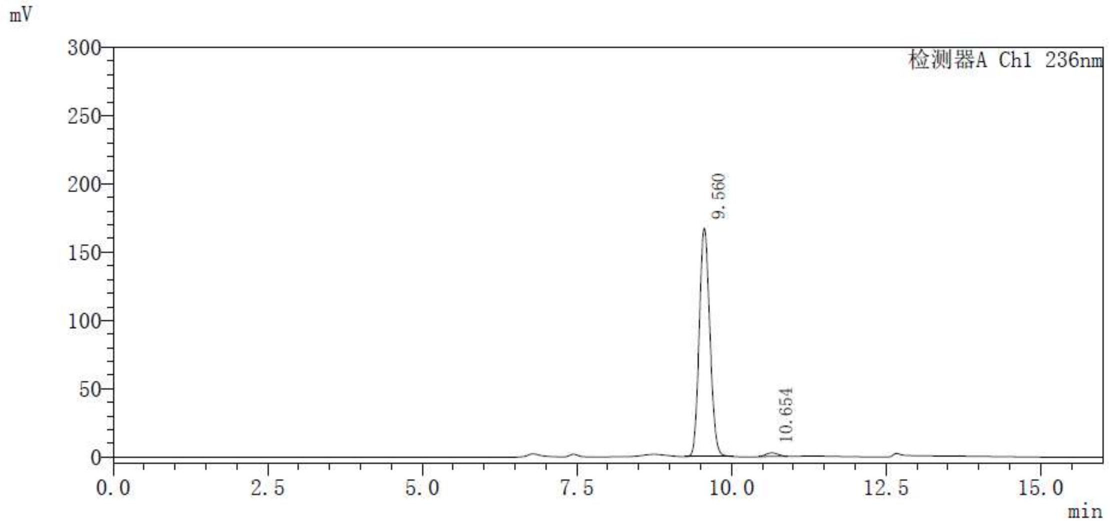

Peak	Retention Time (min)	Relative Area (%)	ee (%)
1	9.560	98.300	97
2	10.654	1.700	

Supplementary Fig. 96. HPLC Spectra of **5**

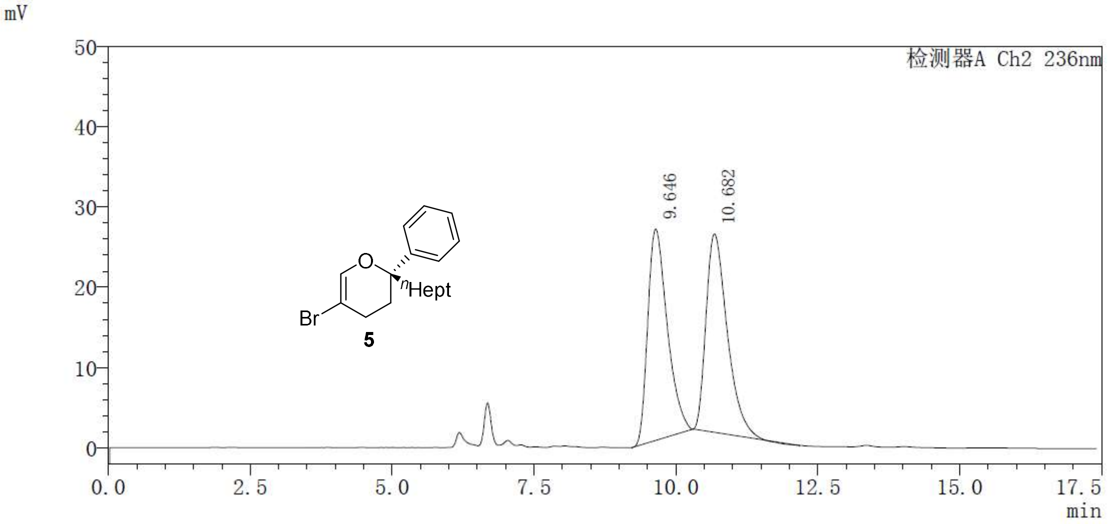

Peak	Retention Time (min)	Relative Area (%)
1	9.646	49.562
2	10.682	50.438

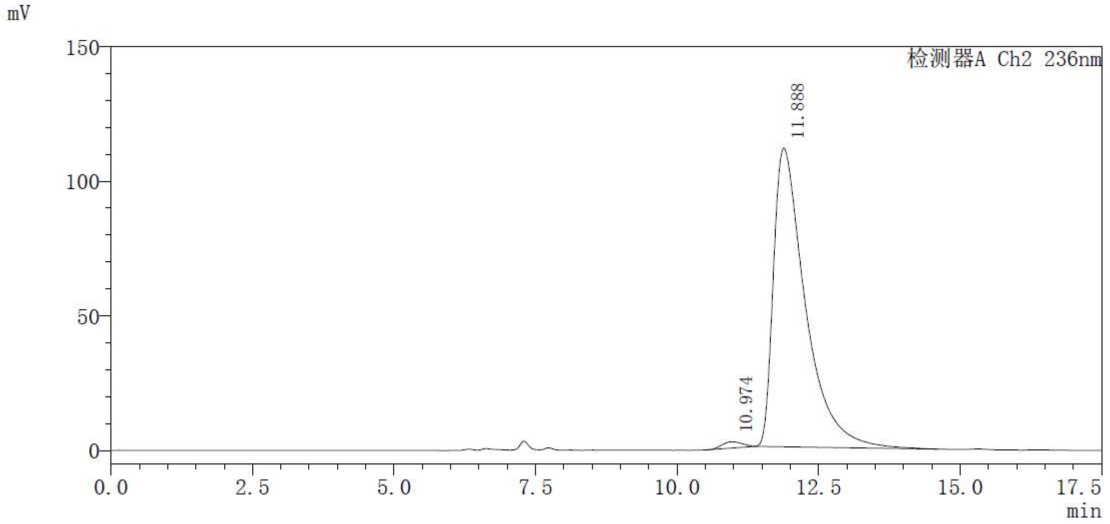

Peak	Retention Time (min)	Relative Area (%)	ee (%)
1	10.974	1.331	97
2	11.888	98.669	

Supplementary Fig. 97. HPLC Spectra of **6**

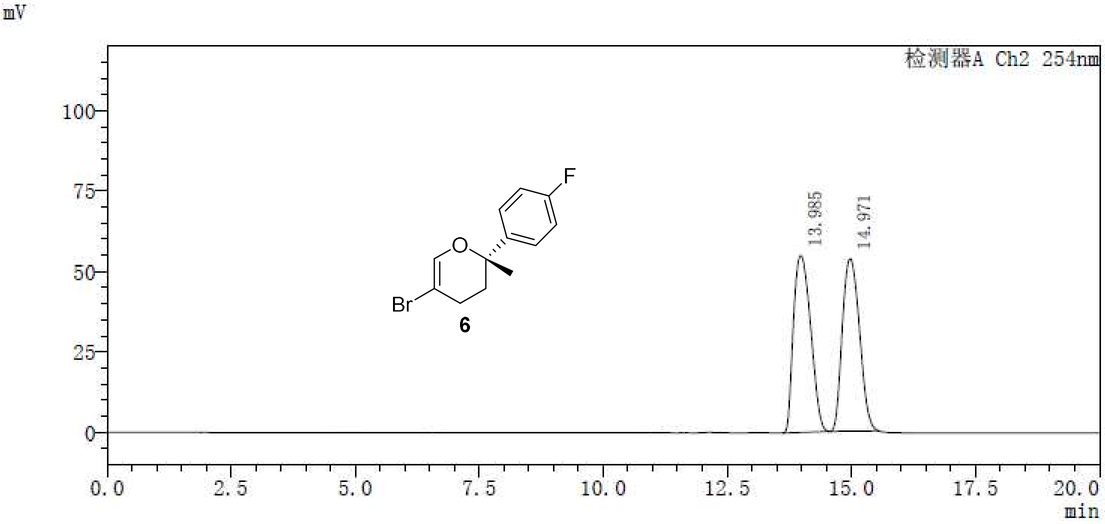

Peak	Retention Time (min)	Relative Area (%)
1	13.985	50.398
2	14.971	49.602

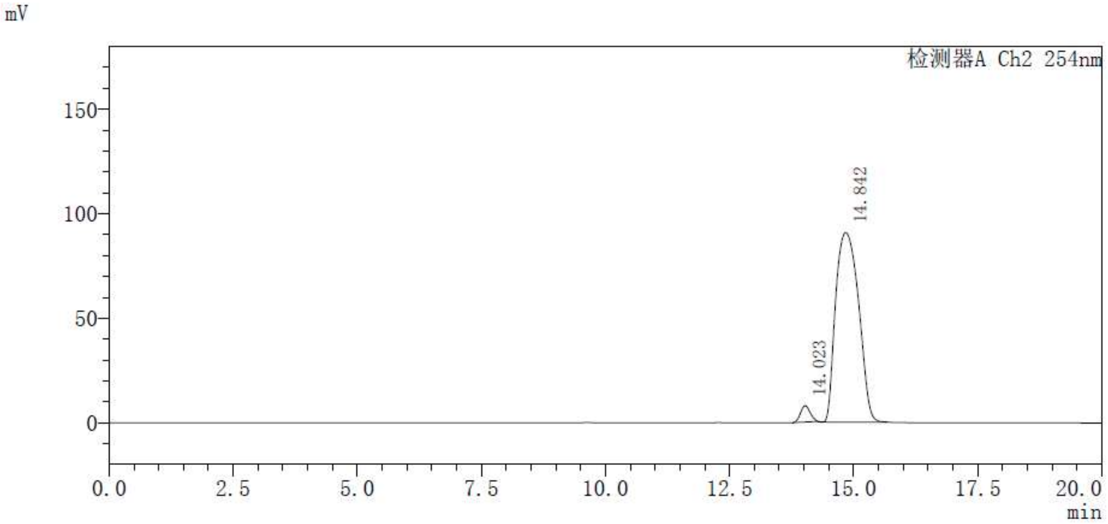

Peak	Retention Time (min)	Relative Area (%)	ee (%)
1	14.023	3.423	93
2	14.842	96.577	

Supplementary Fig. 98. HPLC Spectra of **7**

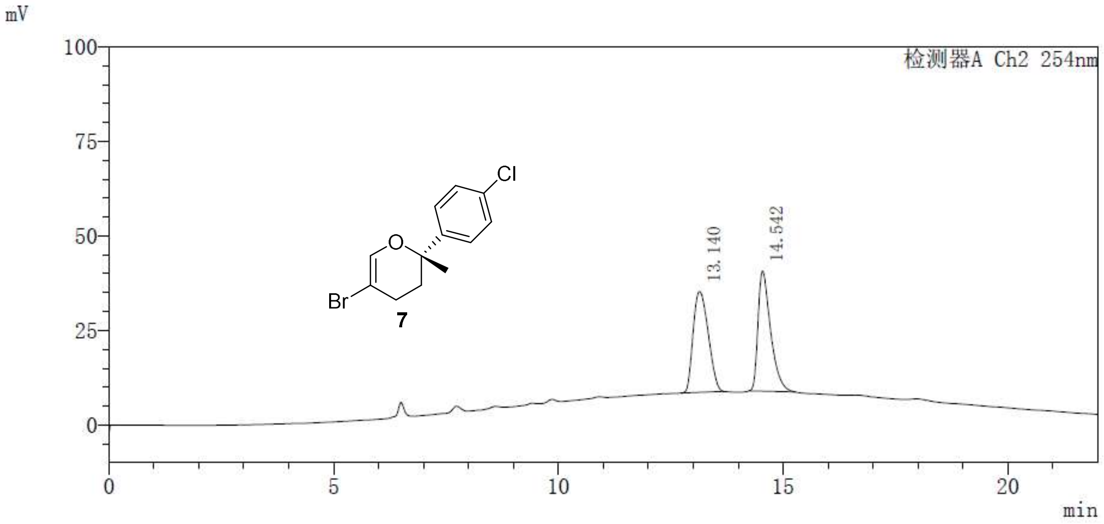

Peak	Retention Time (min)	Relative Area (%)
1	13.140	50.198
2	14.542	49.802

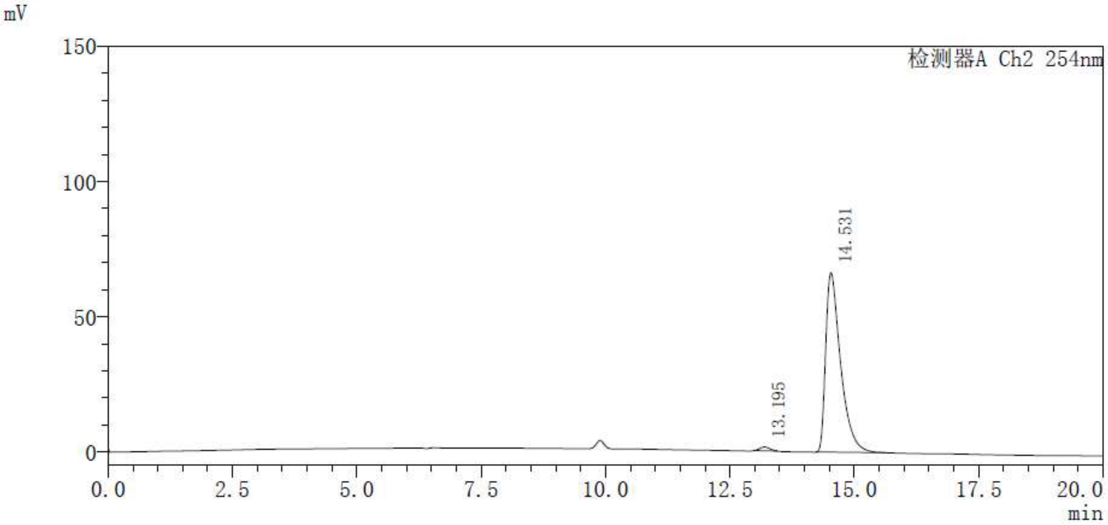

Peak	Retention Time (min)	Relative Area (%)	ee (%)
1	13.195	1.424	97
2	14.531	98.576	

Supplementary Fig. 99. HPLC Spectra of **8**

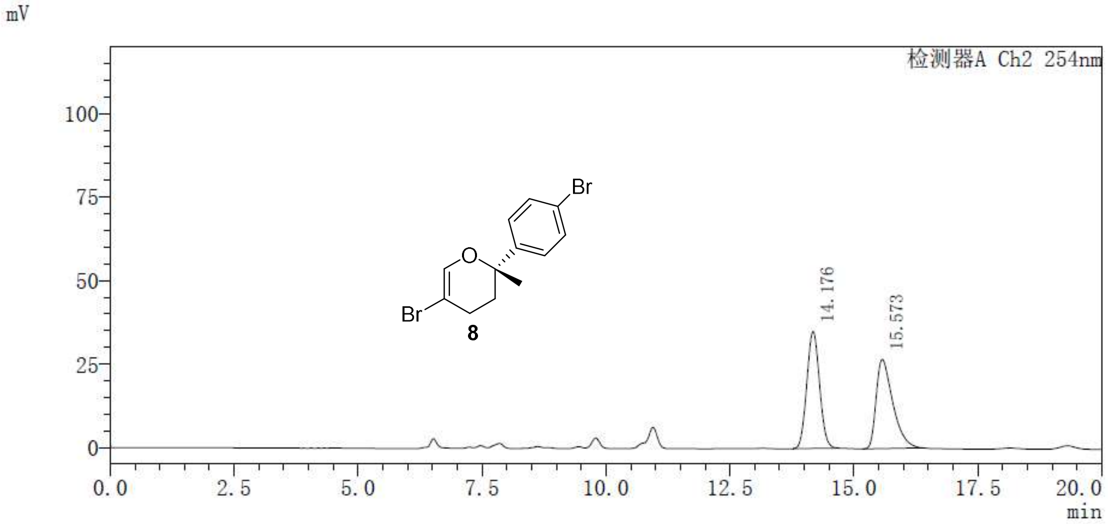

Peak	Retention Time (min)	Relative Area (%)
1	14.176	50.158
2	15.573	49.842

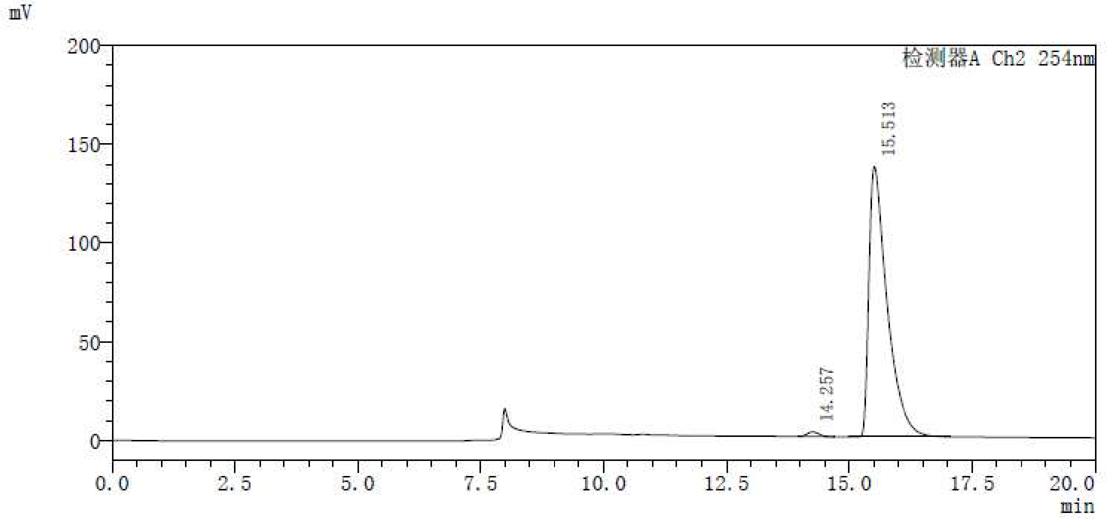

Peak	Retention Time (min)	Relative Area (%)	ee (%)
1	14.257	1.052	98
2	15.513	98.948	

Supplementary Fig. 100. HPLC Spectra of **9**

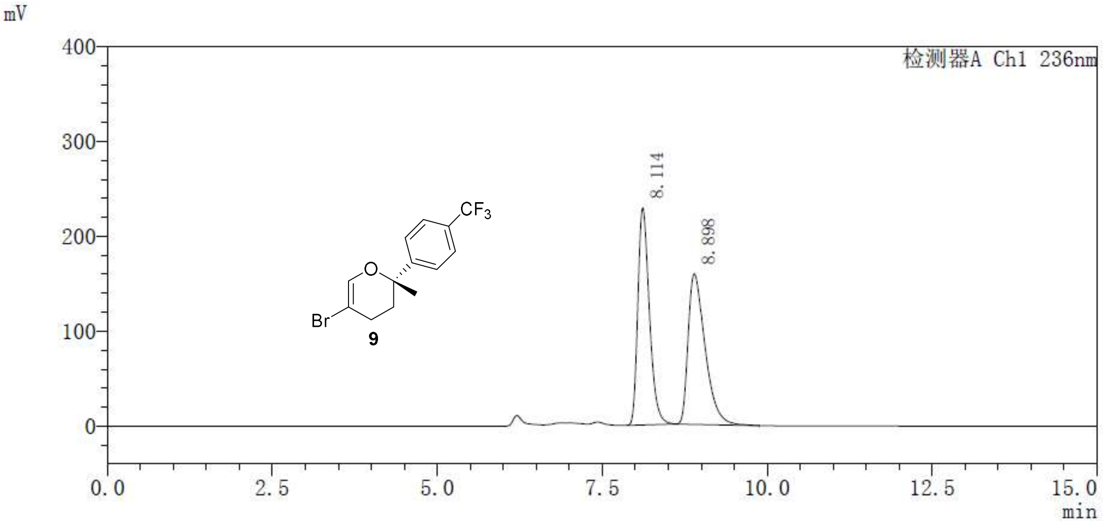

Peak	Retention Time (min)	Relative Area (%)
1	8.114	49.716
2	8.898	50.284

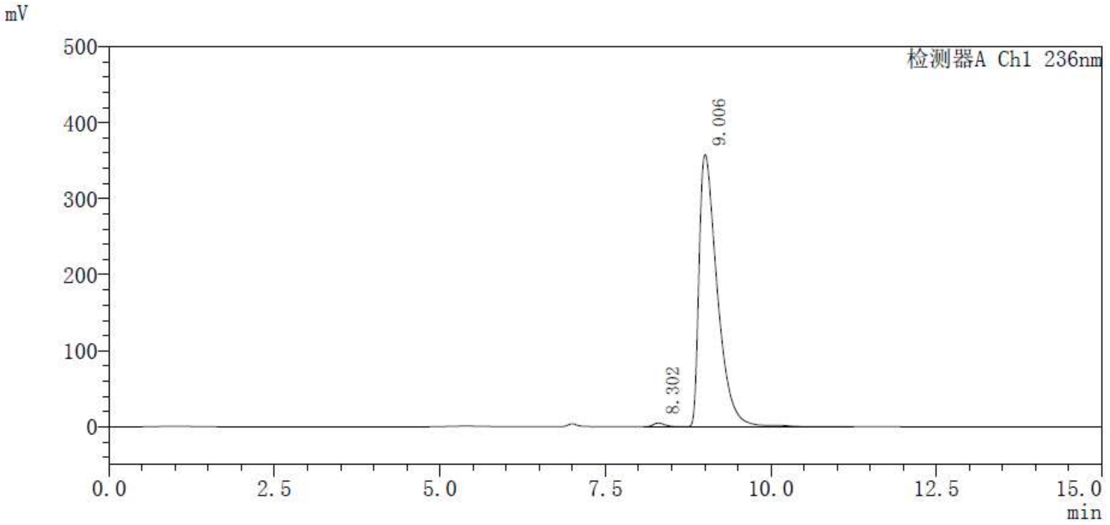

Peak	Retention Time (min)	Relative Area (%)	ee (%)
1	8.302	0.874	98
2	9.006	99.126	

Supplementary Fig. 101. HPLC Spectra of **10**

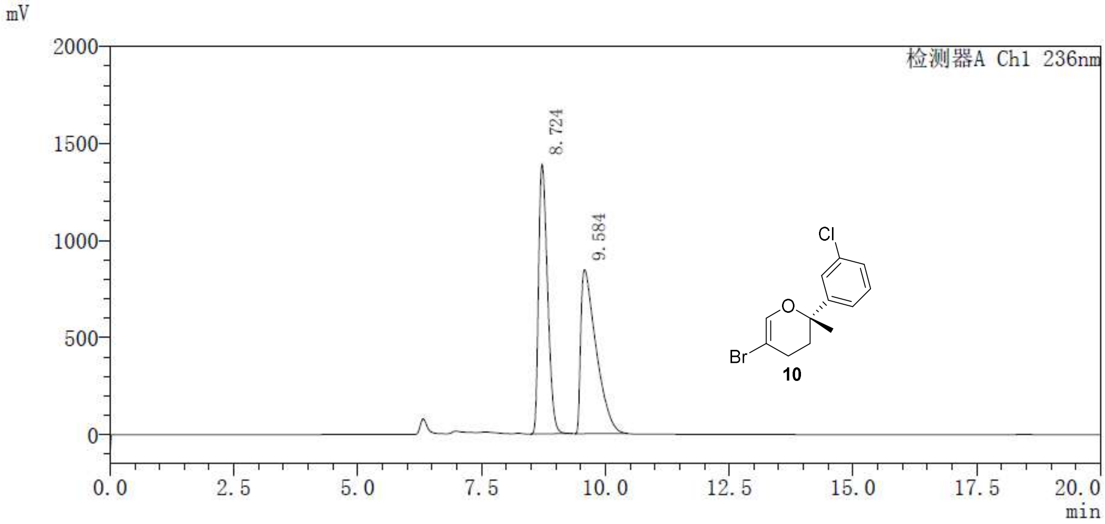

Peak	Retention Time (min)	Relative Area (%)
1	8.724	49.001
2	9.584	50.999

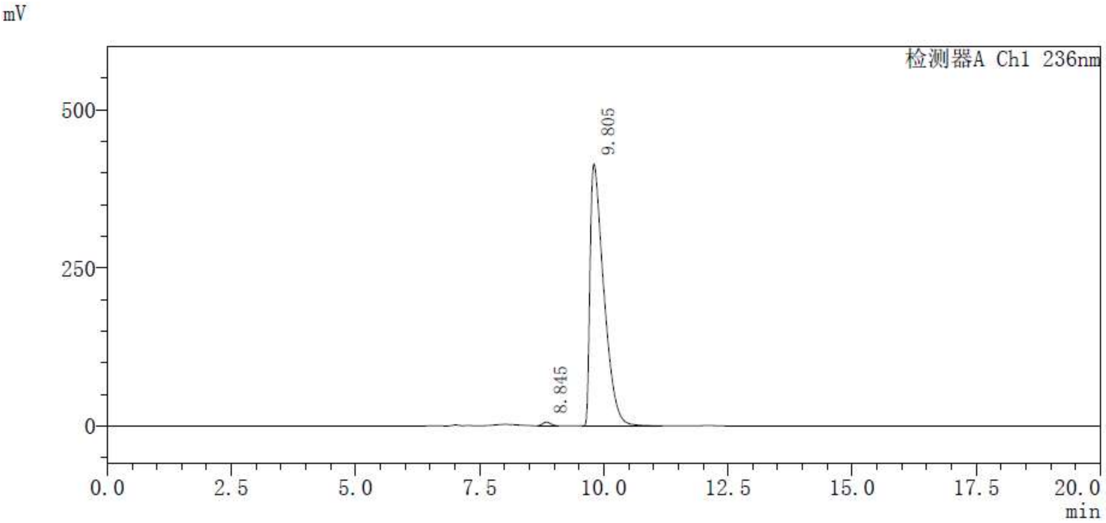

Peak	Retention Time (min)	Relative Area (%)	ee (%)
1	8.845	0.761	98
2	9.805	99.239	

Supplementary Fig. 102. HPLC Spectra of **11**

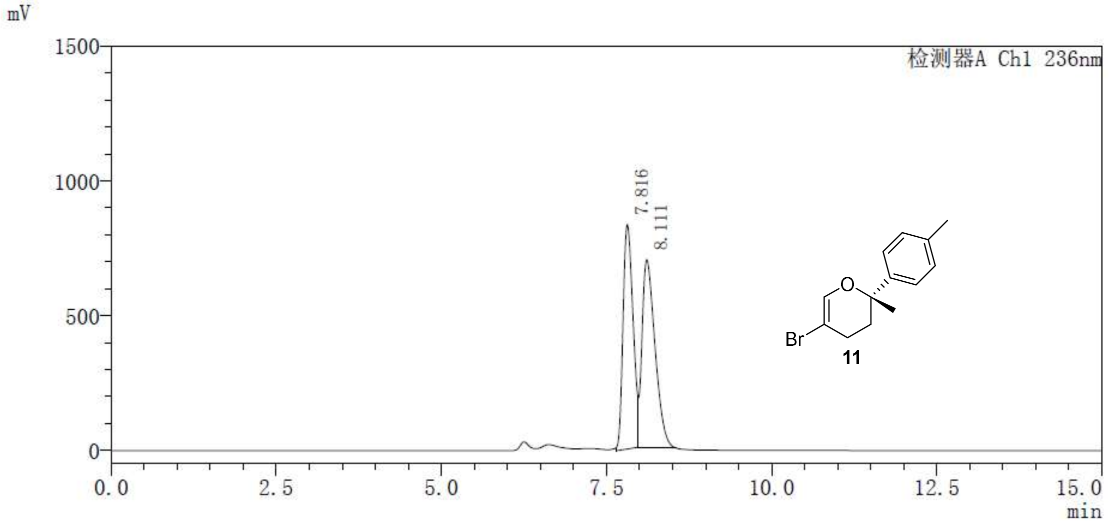

Peak	Retention Time (min)	Relative Area (%)
1	7.816	48.118
2	8.111	51.882

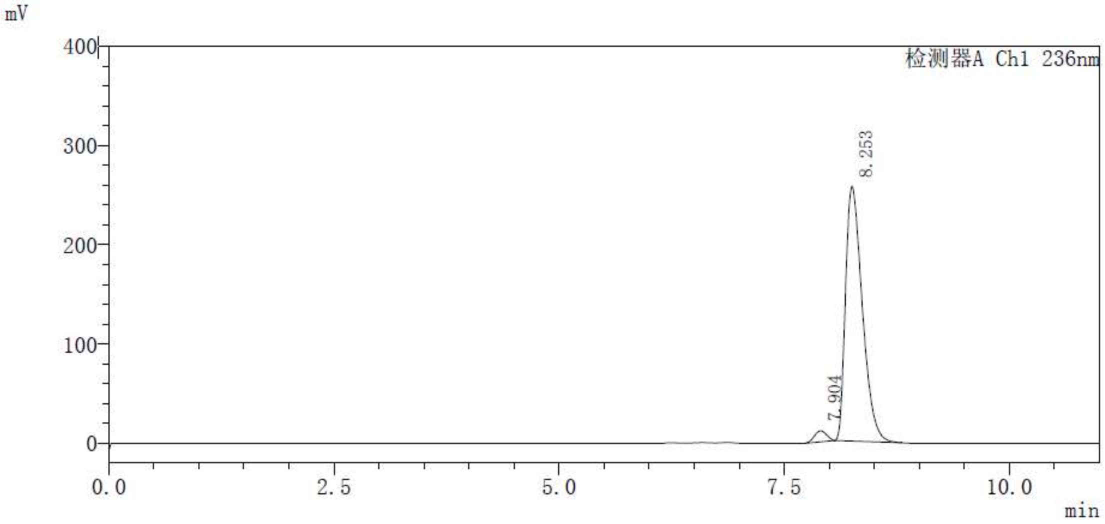

Peak	Retention Time (min)	Relative Area (%)	ee (%)
1	7.904	3.073	94
2	8.253	96.927	

Supplementary Fig. 103. HPLC Spectra of 12

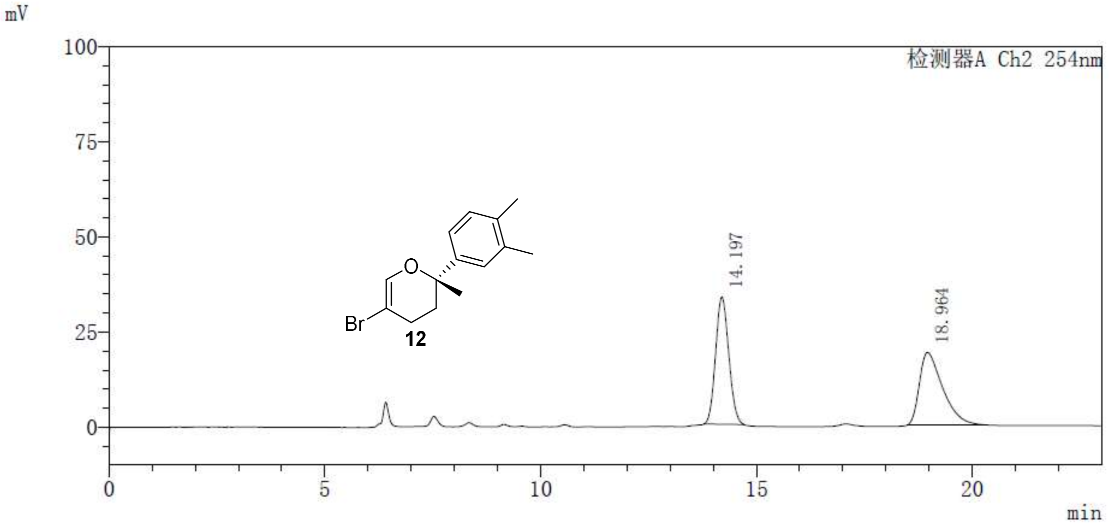

Peak	Retention Time (min)	Relative Area (%)
1	14.197	49.998
2	18.964	50.002

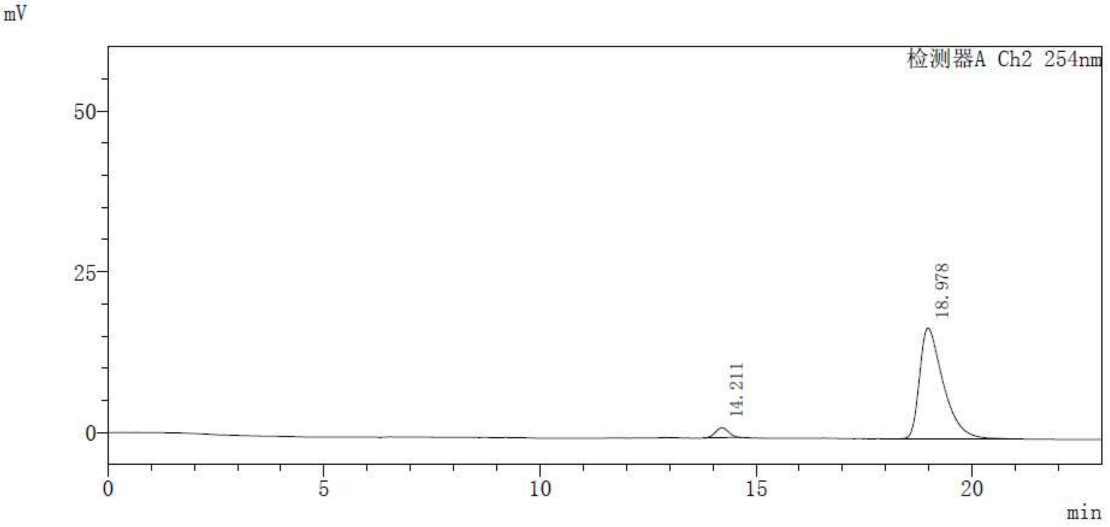

Peak	Retention Time (min)	Relative Area (%)	ee (%)
1	14.211	4.673	91
2	18.978	95.327	

Supplementary Fig. 104. HPLC Spectra of **13**

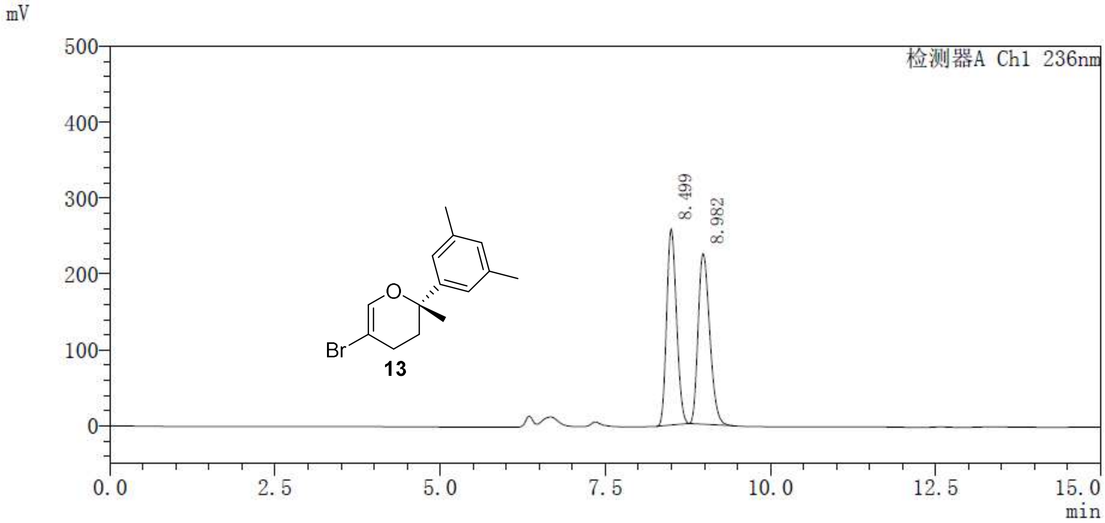

Peak	Retention Time (min)	Relative Area (%)
1	8.499	49.845
2	8.982	50.155

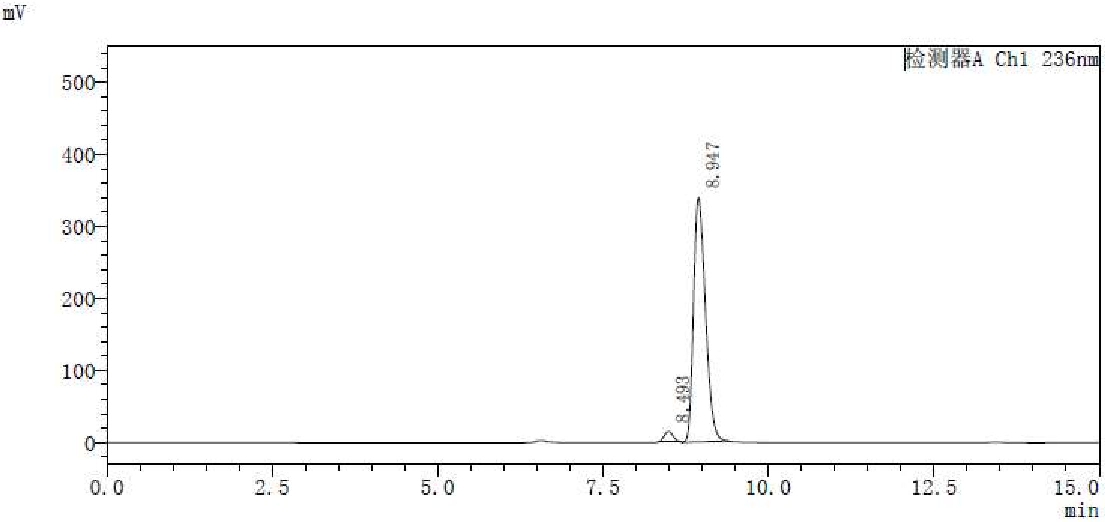

Peak	Retention Time (min)	Relative Area (%)	ee (%)
1	8.493	3.182	94
2	8.947	96.818	

Supplementary Fig. 105. HPLC Spectra of **14**

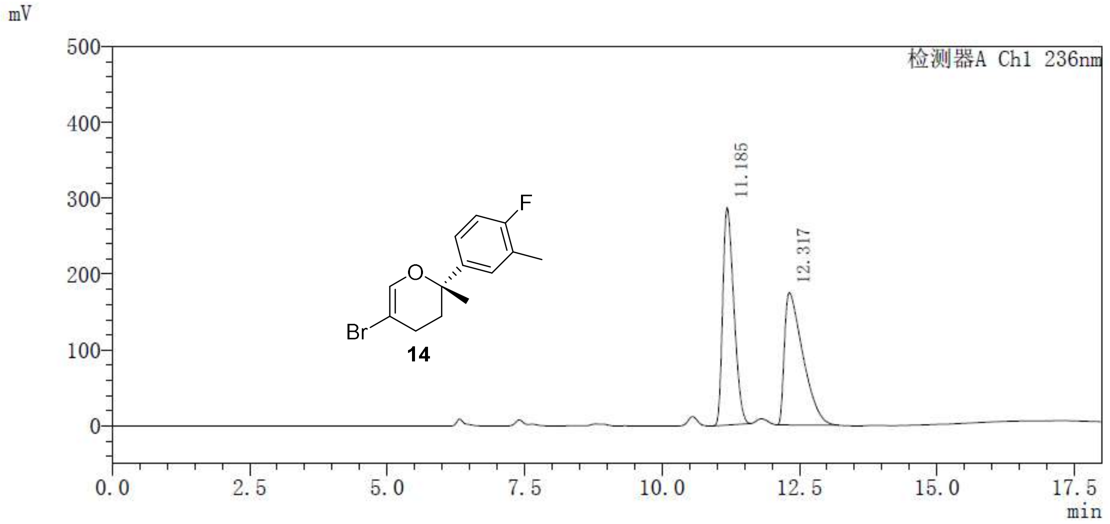

Peak	Retention Time (min)	Relative Area (%)
1	11.185	49.757
2	12.317	50.243

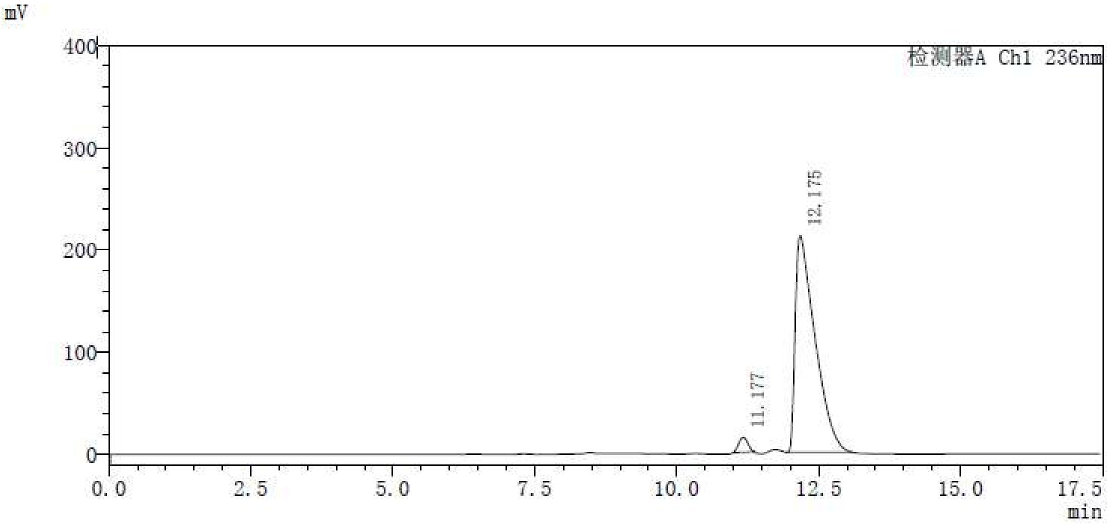

Peak	Retention Time (min)	Relative Area (%)	ee (%)
1	11.177	3.113	94
2	12.175	96.887	

Supplementary Fig. 106. HPLC Spectra of **15**

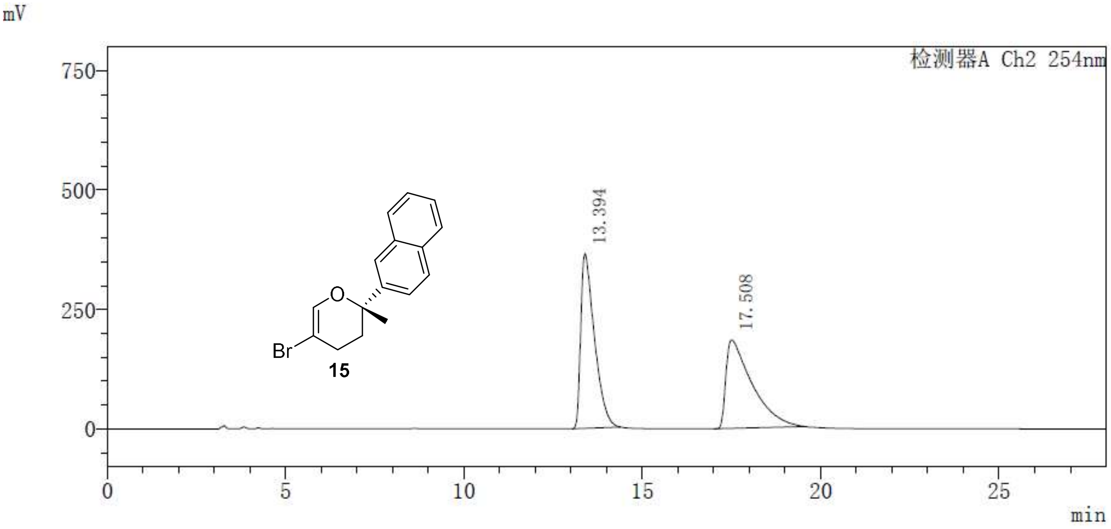

Peak	Retention Time (min)	Relative Area (%)
1	13.394	50.736
2	17.508	49.264

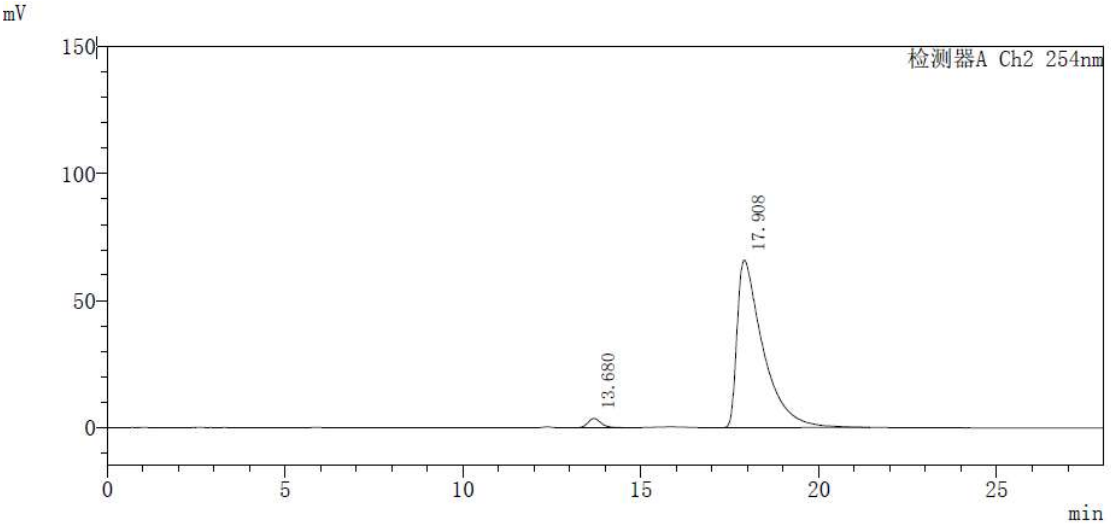

Peak	Retention Time (min)	Relative Area (%)	ee (%)
1	13.680	2.687	95
2	17.908	97.313	

Supplementary Fig. 107. HPLC Spectra of **16**

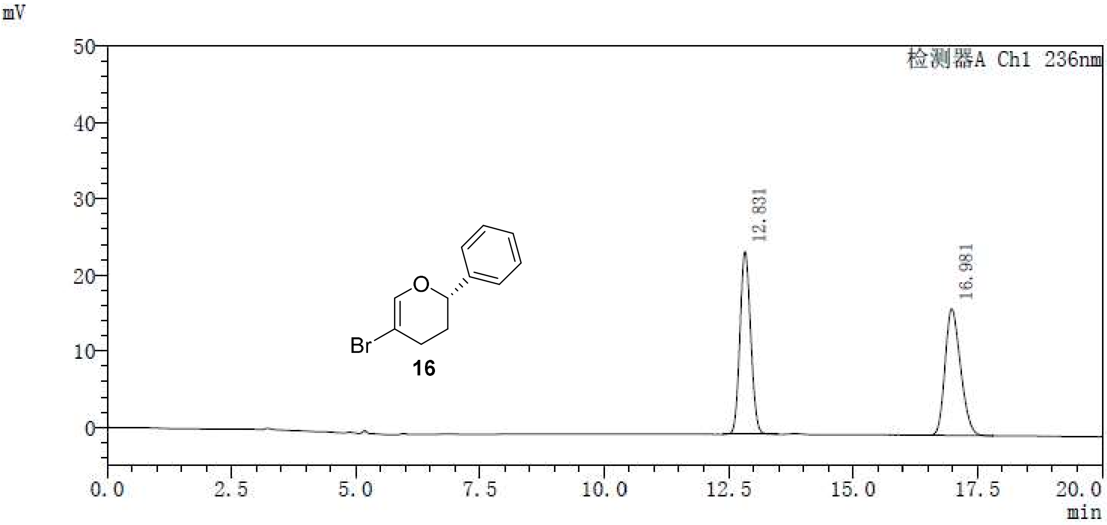

Peak	Retention Time (min)	Relative Area (%)
1	12.831	49.894
2	16.981	50.106

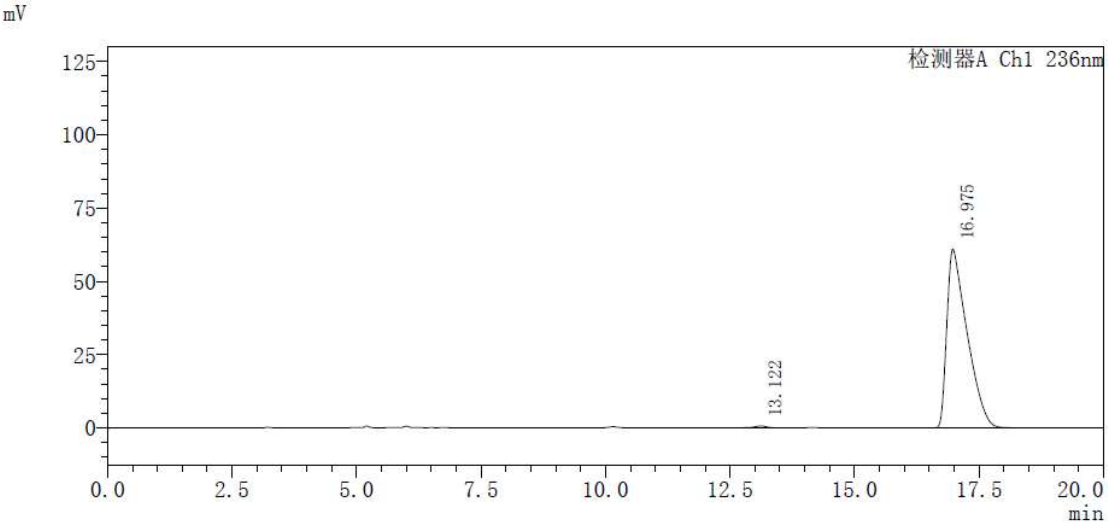

Peak	Retention Time (min)	Relative Area (%)	ee (%)
1	13.122	0.537	99
2	16.975	99.463	

Supplementary Fig. 108. HPLC Spectra of **17**

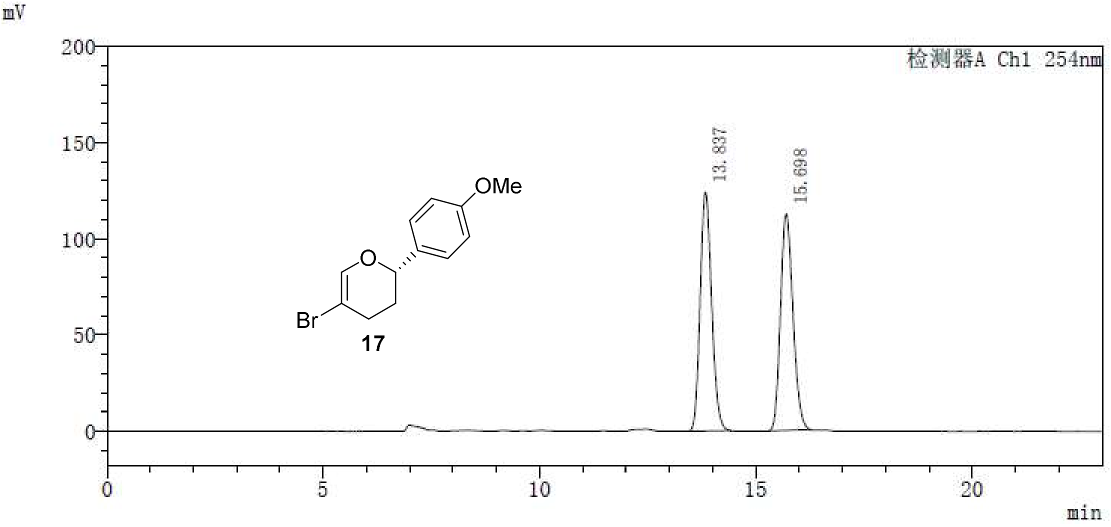

Peak	Retention Time (min)	Relative Area (%)
1	13.837	50.371
2	15.698	49.629

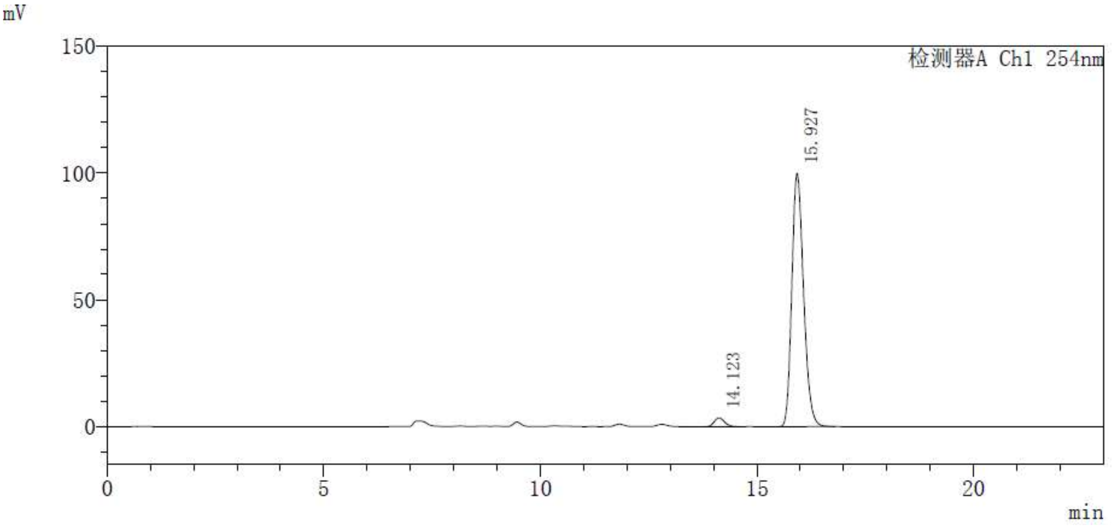

Peak	Retention Time (min)	Relative Area (%)	ee (%)
1	14.123	2.987	94
2	15.927	97.013	

Supplementary Fig. 109. HPLC Spectra of **18**

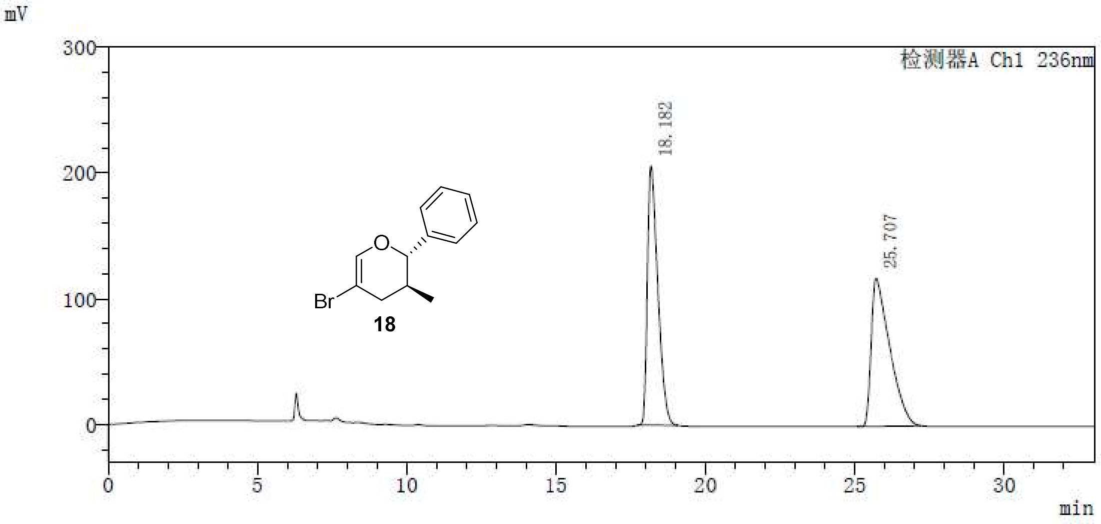

Peak	Retention Time (min)	Relative Area (%)
1	18.182	49.455
2	25.707	50.545

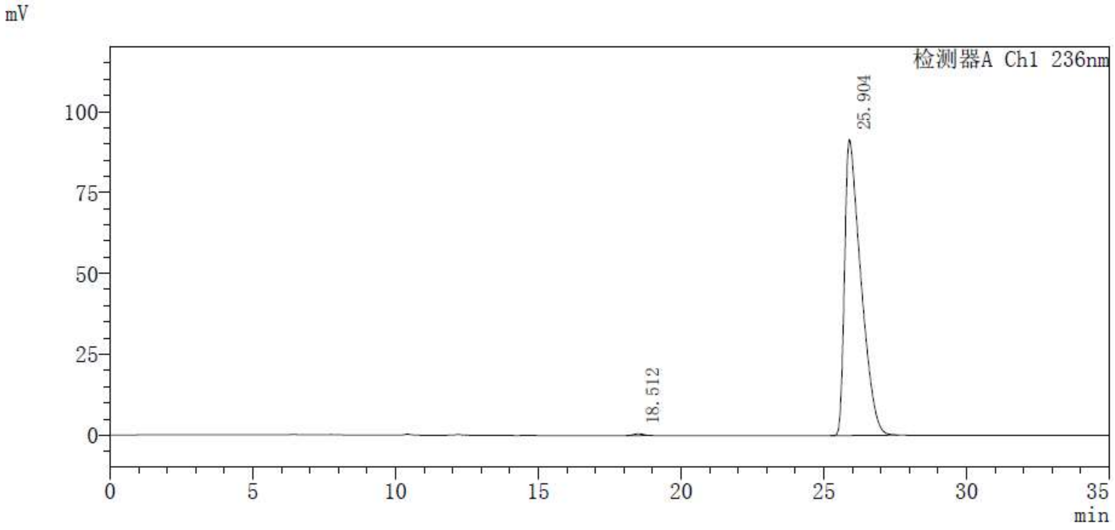

Peak	Retention Time (min)	Relative Area (%)	ee (%)
1	18.512	0.337	99
2	25.904	99.663	

Supplementary Fig. 110. HPLC Spectra of **19**

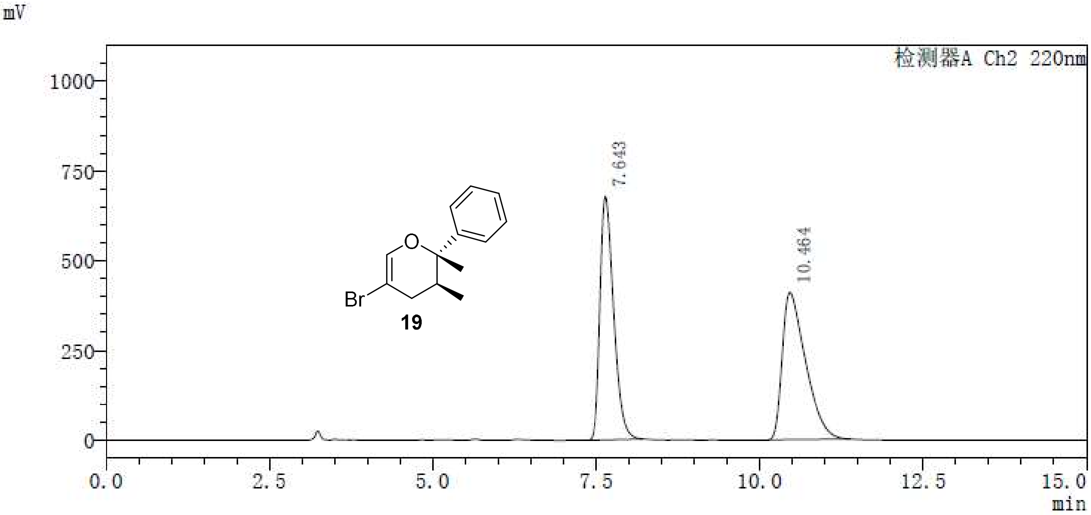

Peak	Retention Time (min)	Relative Area (%)
1	7.643	49.216
2	10.464	50.784

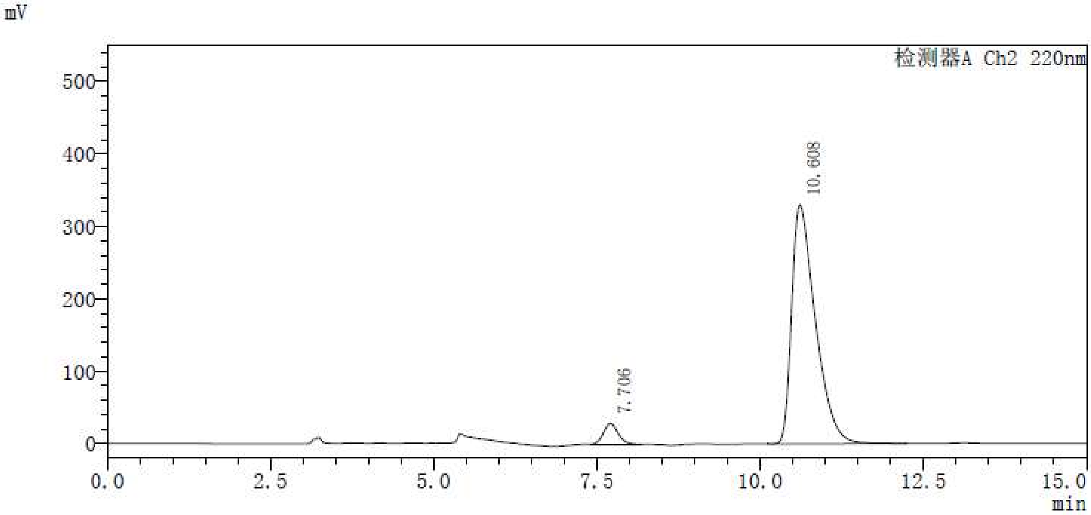

Peak	Retention Time (min)	Relative Area (%)	ee (%)
1	7.706	5.059	90
2	10.608	94.941	

Supplementary Fig. 111. HPLC Spectra of **20**

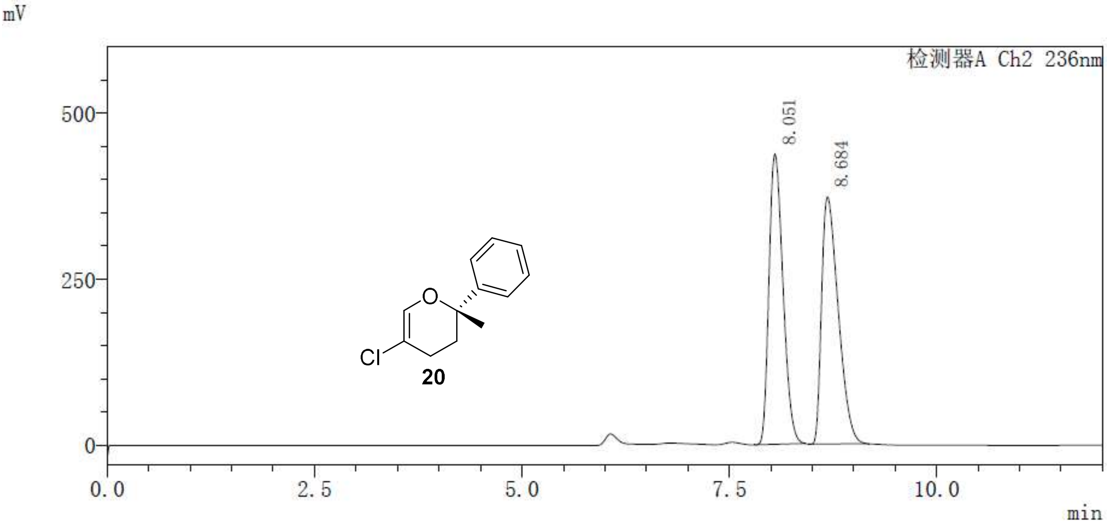

Peak	Retention Time (min)	Relative Area (%)
1	8.051	49.742
2	8.684	50.258

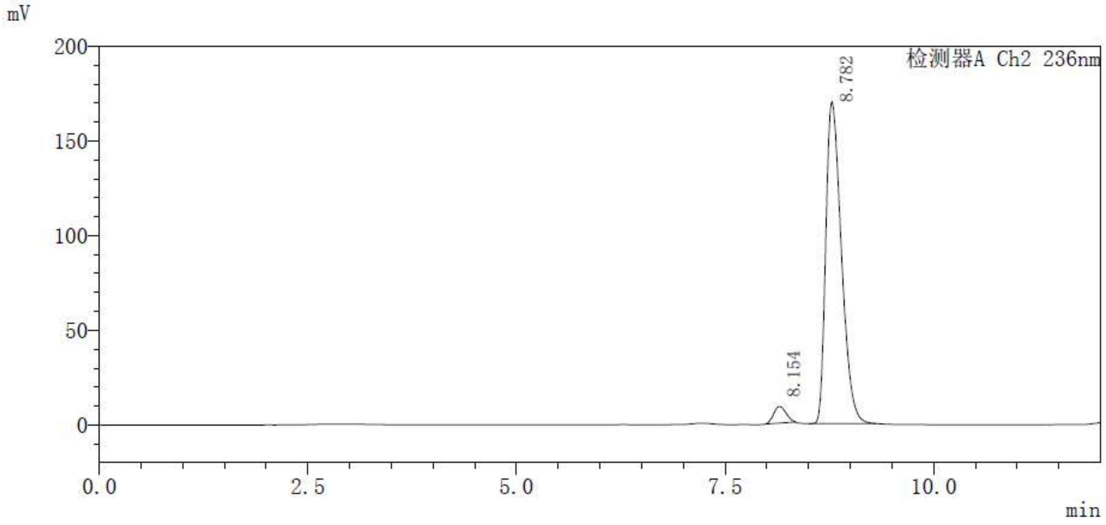

Peak	Retention Time (min)	Relative Area (%)	ee (%)
1	8.154	3.914	92
2	8.782	96.086	

Supplementary Fig. 112. HPLC Spectra of **21**

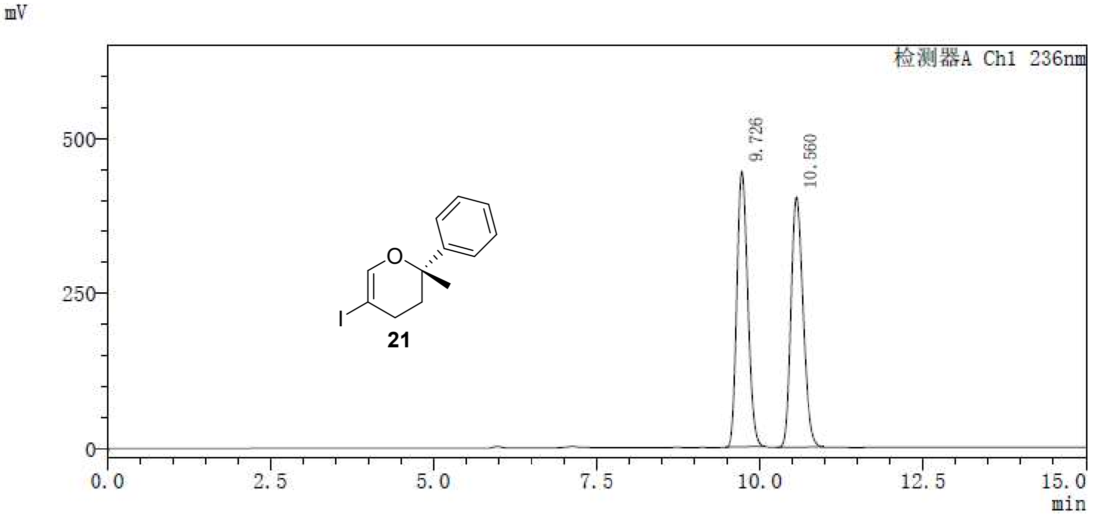

Peak	Retention Time (min)	Relative Area (%)
1	9.726	49.620
2	10.560	50.380

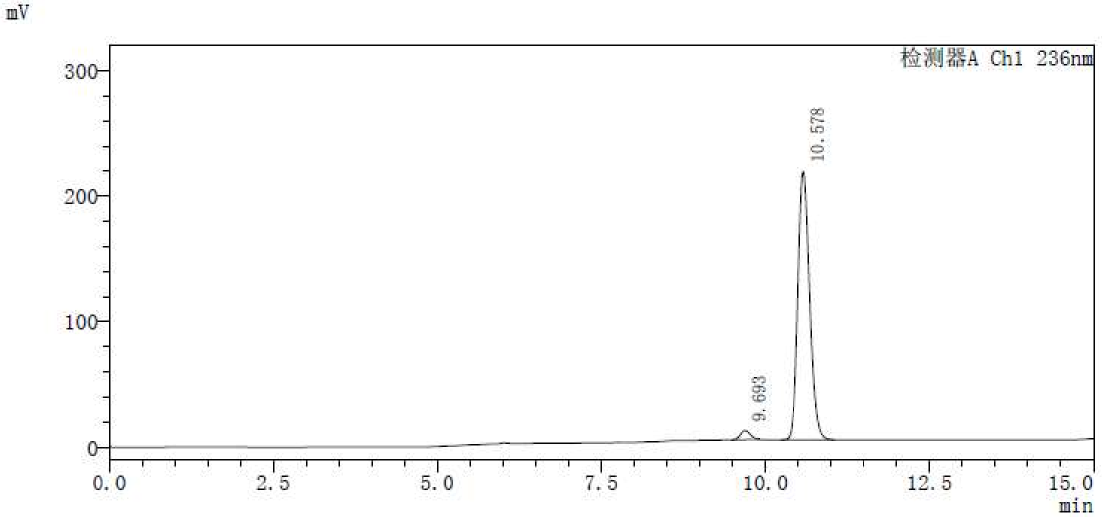

Peak	Retention Time (min)	Relative Area (%)	ee (%)
1	9.693	2.686	95
2	10.578	97.314	

Supplementary Fig. 113. HPLC Spectra of **22**

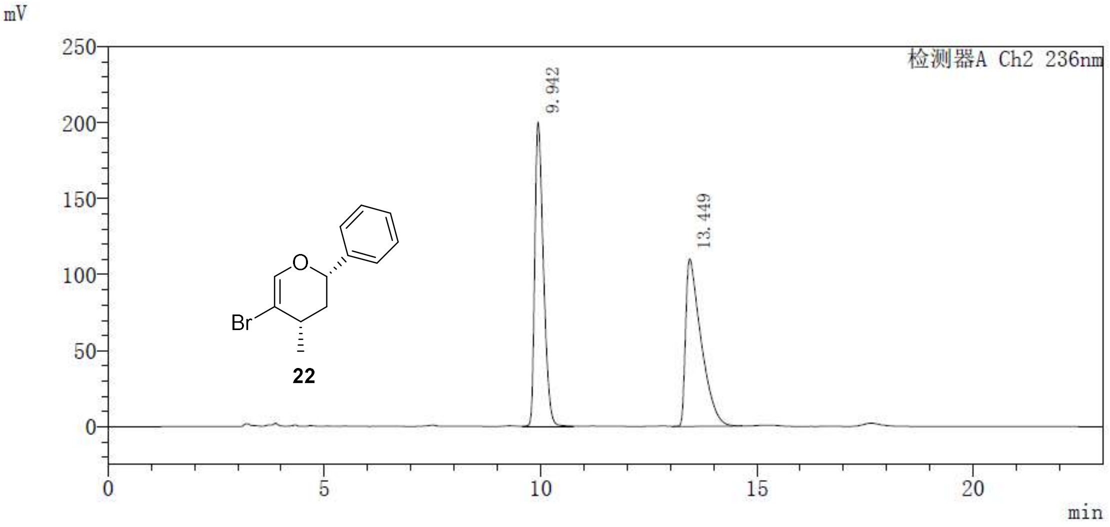

Peak	Retention Time (min)	Relative Area (%)
1	9.942	50.205
2	13.449	49.795

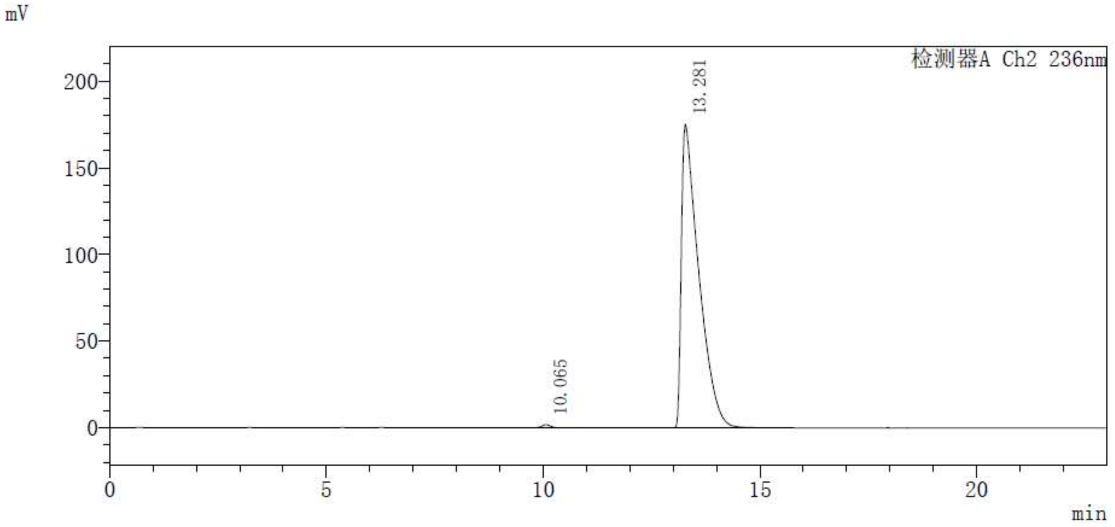

Peak	Retention Time (min)	Relative Area (%)	ee (%)
1	10.065	0.473	99
2	13.281	99.527	

Supplementary Fig. 114. HPLC Spectra of **23**

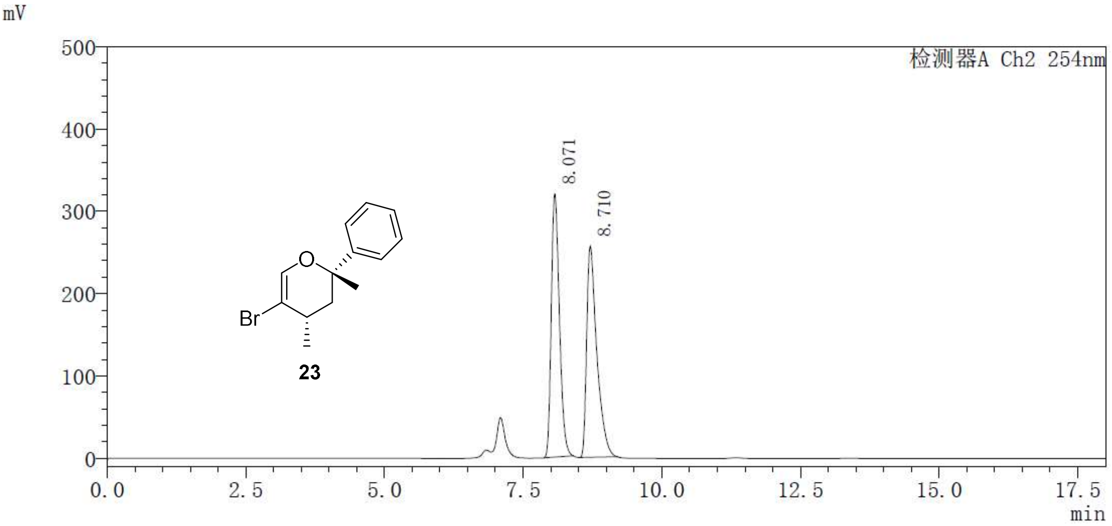

Peak	Retention Time (min)	Relative Area (%)
1	8.071	49.942
2	8.710	50.058

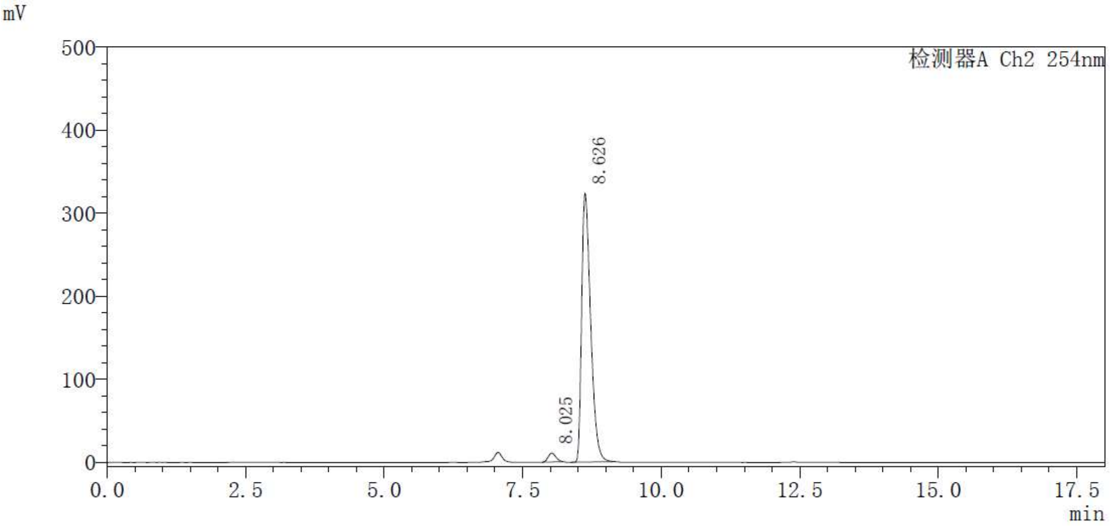

Peak	Retention Time (min)	Relative Area (%)	ee (%)
1	8.025	2.775	95
2	8.626	97.225	

Supplementary Fig. 115. HPLC Spectra of **24**

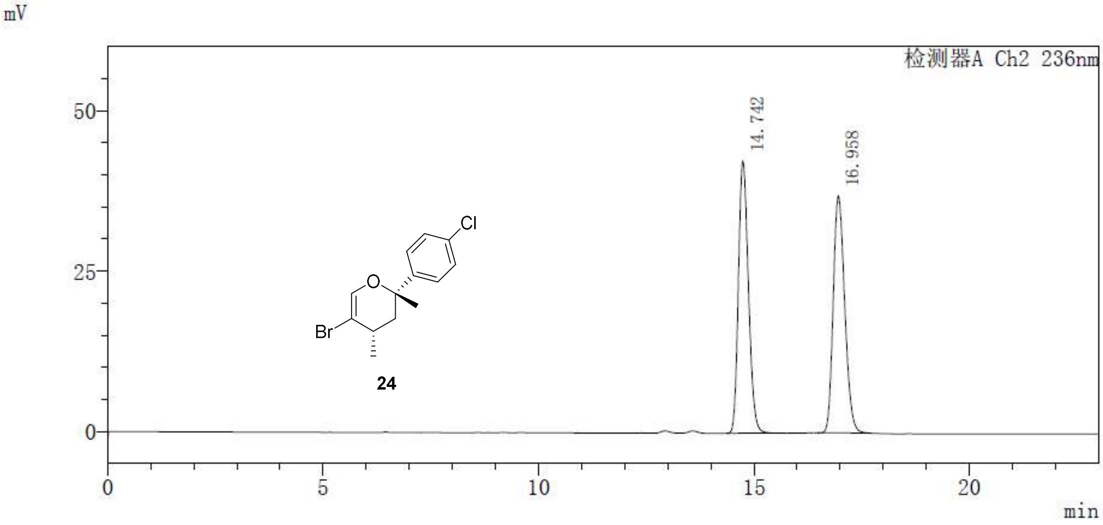

Peak	Retention Time (min)	Relative Area (%)
1	14.742	49.899
2	16.958	50.101

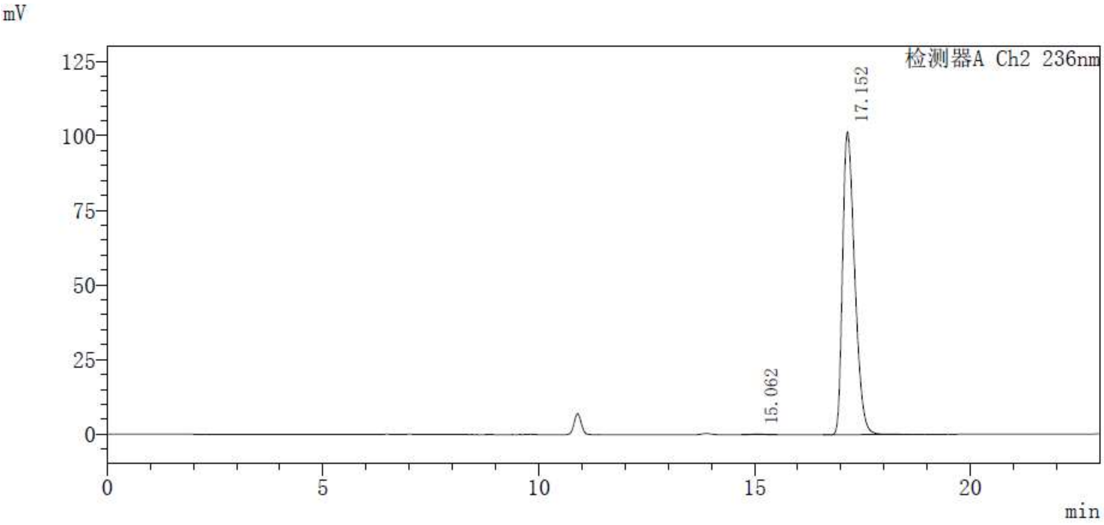

Peak	Retention Time (min)	Relative Area (%)	ee (%)
1	15.062	0.251	99.5
2	17.152	99.749	

Supplementary Fig. 116. HPLC Spectra of **25**

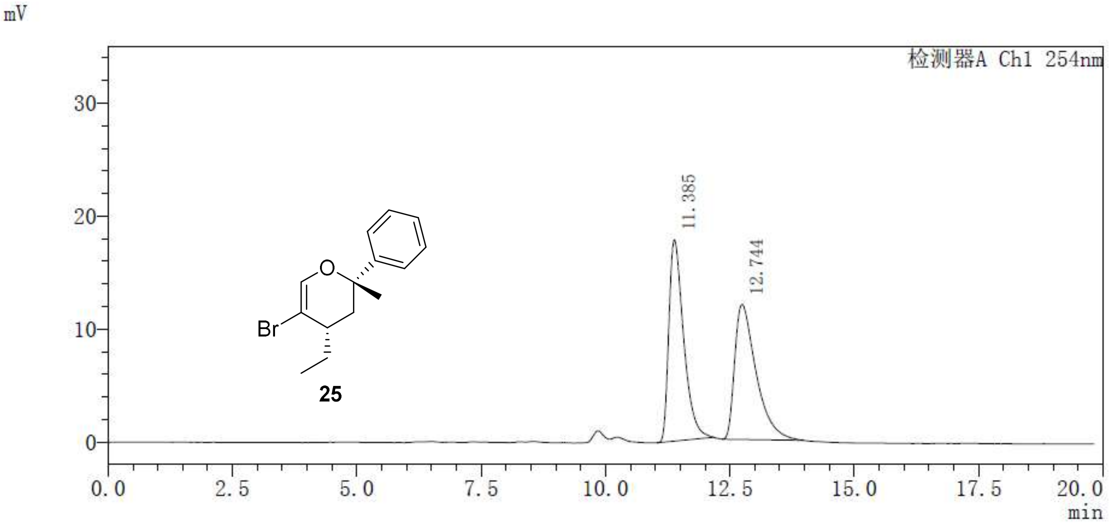

Peak	Retention Time (min)	Relative Area (%)
1	11.385	50.743
2	12.744	49.257

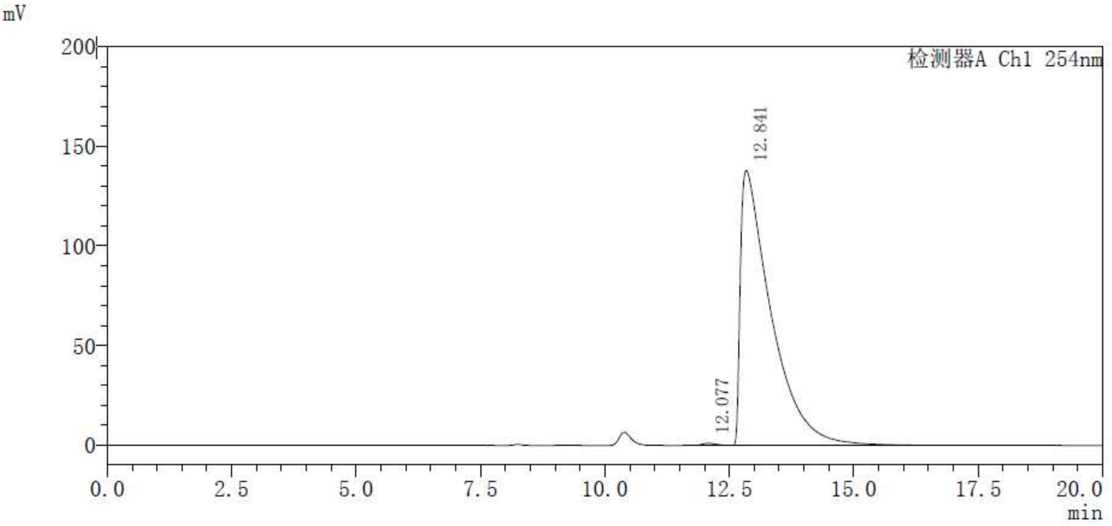

Peak	Retention Time (min)	Relative Area (%)	ee (%)
1	12.077	0.346	99
2	12.841	99.654	

Supplementary Fig. 117. HPLC Spectra of **26**

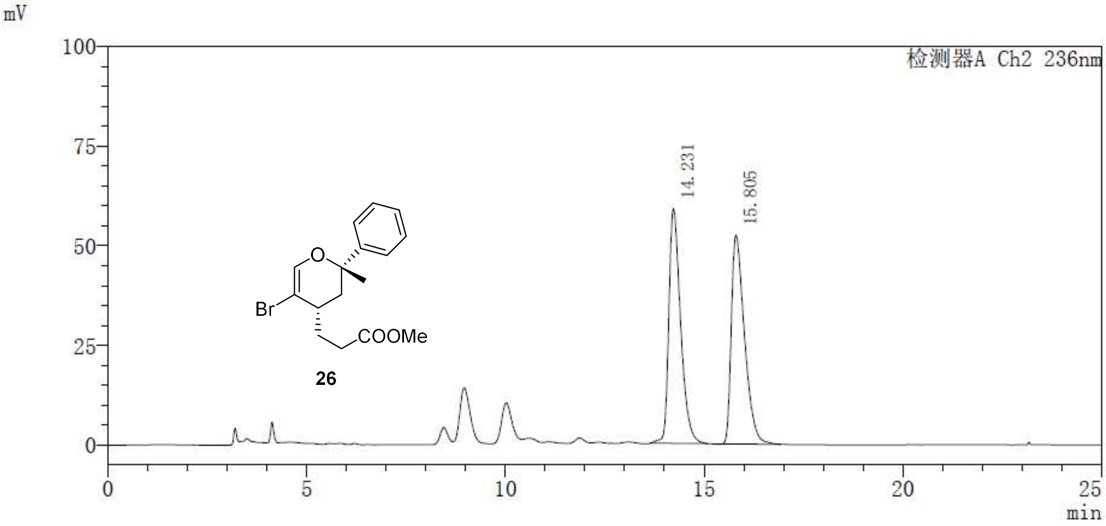

Peak	Retention Time (min)	Relative Area (%)
1	14.231	50.188
2	15.805	49.812

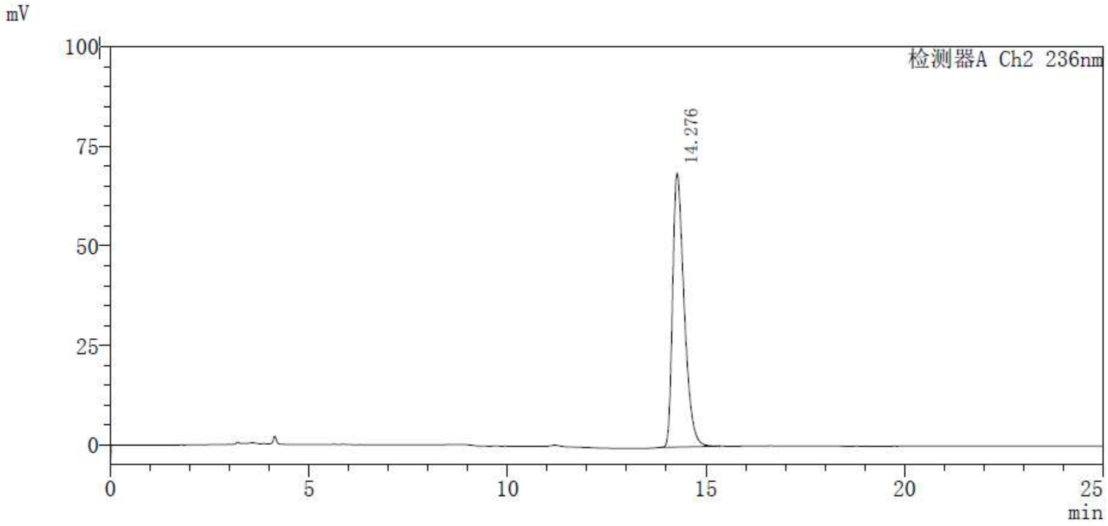

Peak	Retention Time (min)	Relative Area (%)	ee (%)
1	14.276	100	100
2	/	/	

Supplementary Fig. 118. HPLC Spectra of **27**

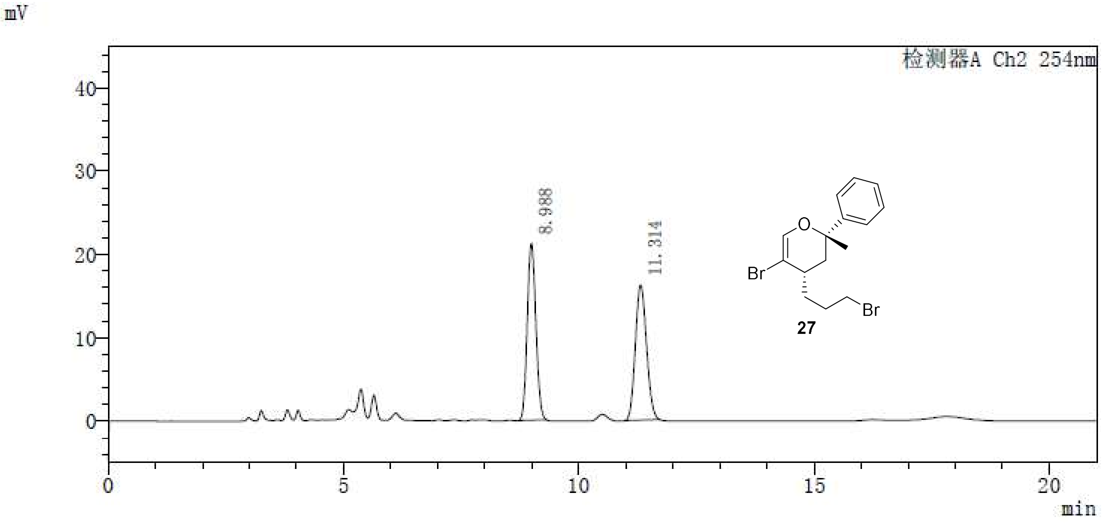

Peak	Retention Time (min)	Relative Area (%)
1	8.988	50.182
2	11.314	49.818

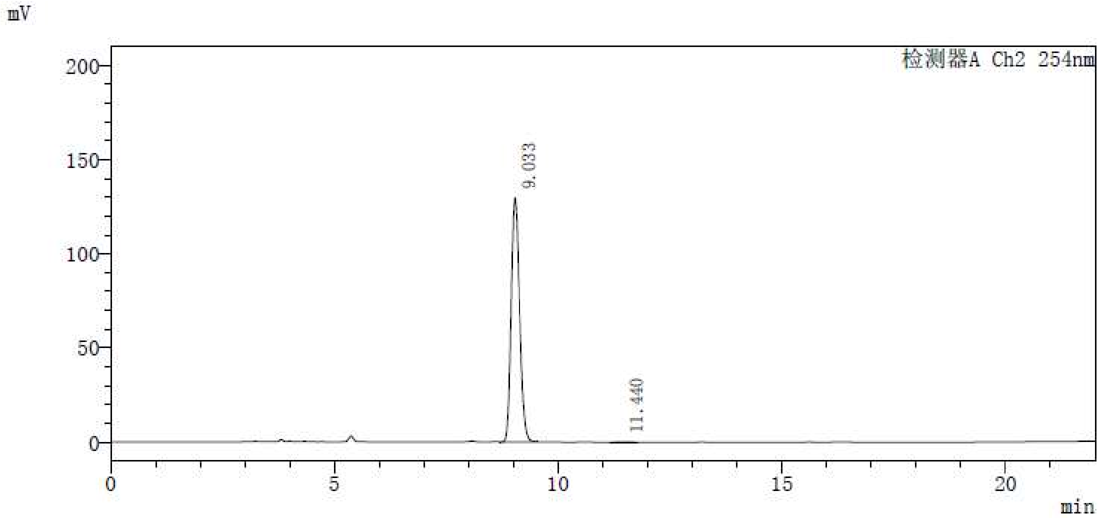

Peak	Retention Time (min)	Relative Area (%)	ee (%)
1	9.033	99.879	99.8
2	11.440	0.121	

Supplementary Fig. 119. HPLC Spectra of **28**

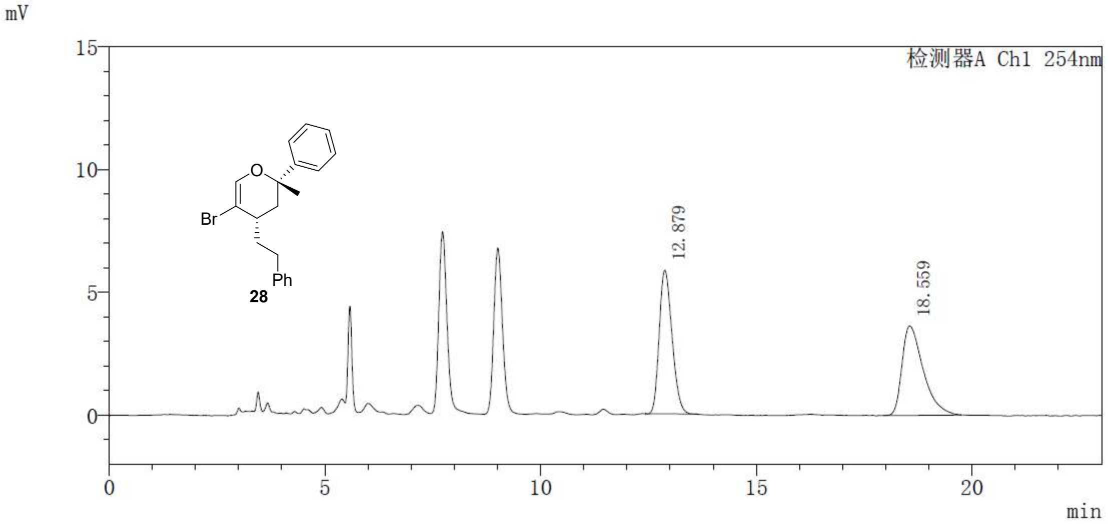

Peak	Retention Time (min)	Relative Area (%)
1	12.879	49.196
2	18.559	50.804

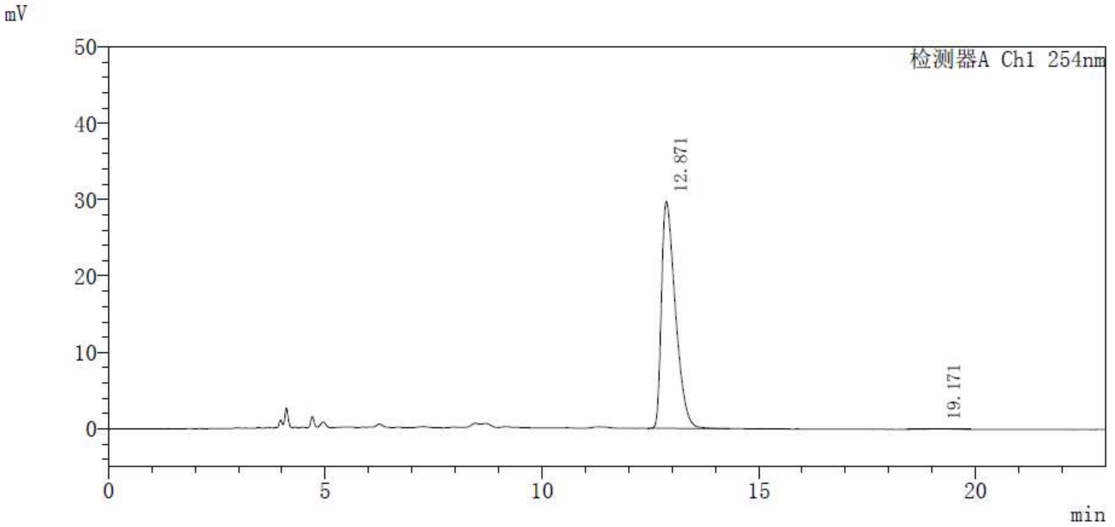

Peak	Retention Time (min)	Relative Area (%)	ee (%)
1	12.871	99.574	99
2	19.171	0.426	

Supplementary Fig. 120. HPLC Spectra of **29**

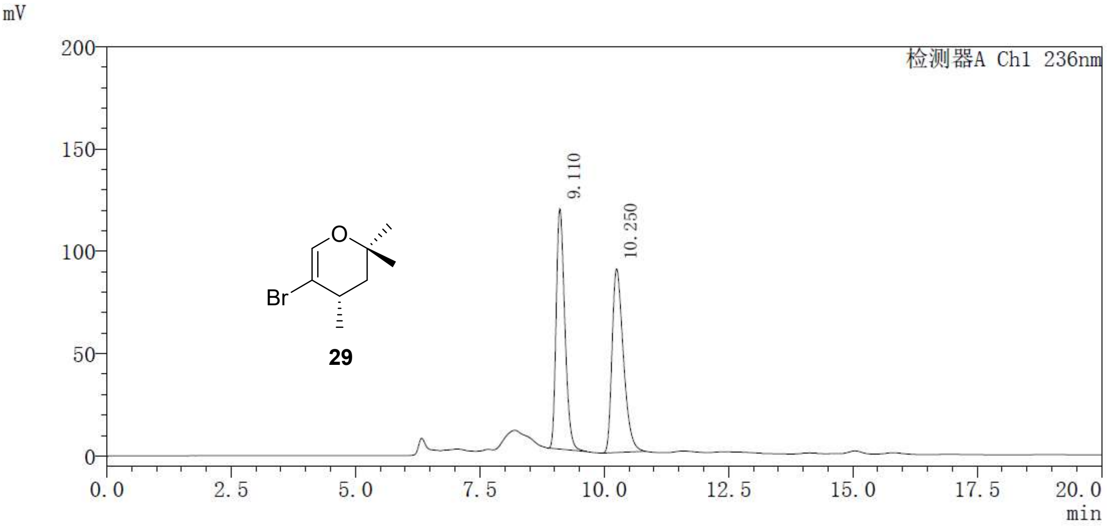

Peak	Retention Time (min)	Relative Area (%)
1	9.110	49.908
2	10.250	50.092

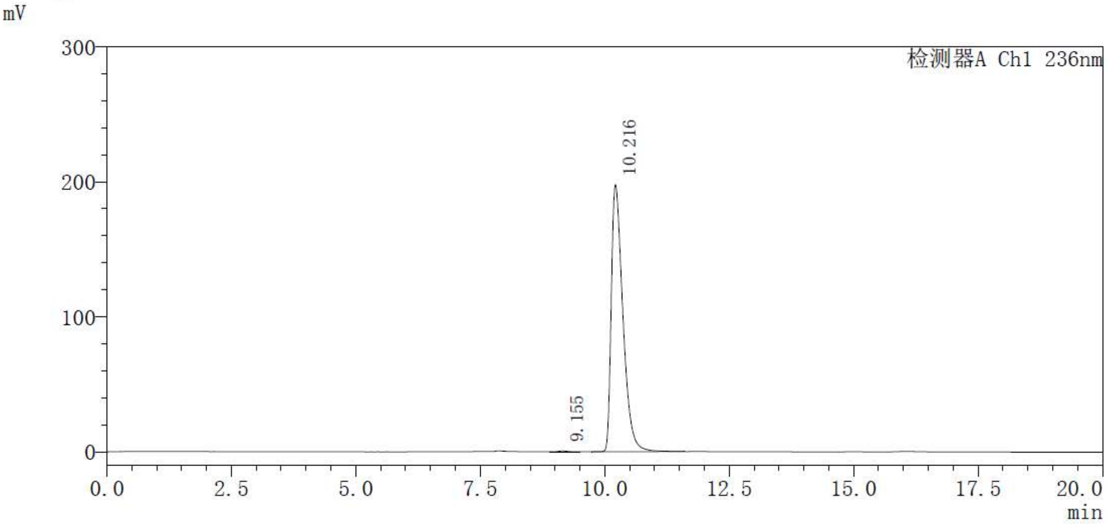

Peak	Retention Time (min)	Relative Area (%)	ee (%)
1	9.155	0.199	99.6
2	10.216	99.801	

Supplementary Fig. 121. HPLC Spectra of **30**

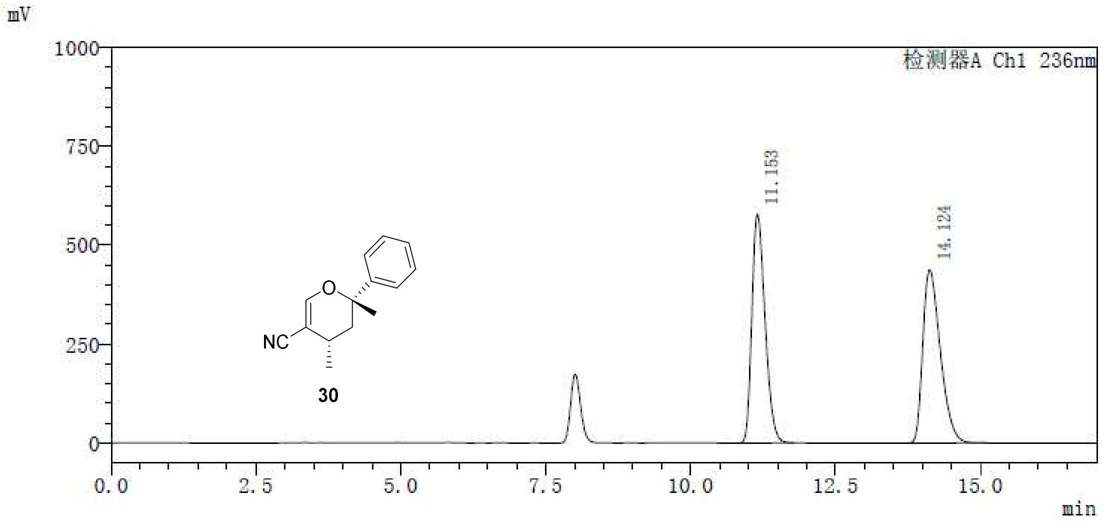

Peak	Retention Time (min)	Relative Area (%)
1	11.153	49.793
2	14.124	50.207

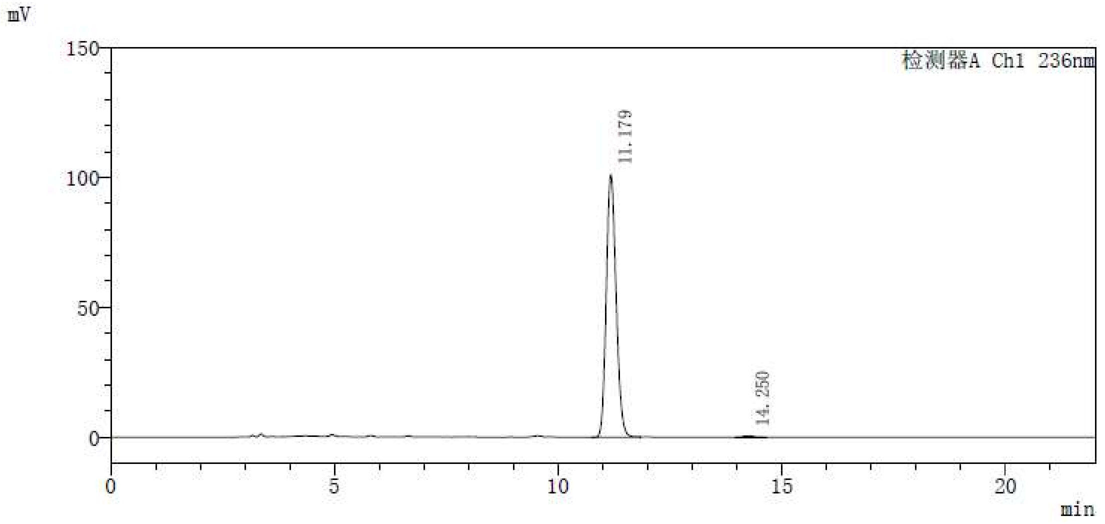

Peak	Retention Time (min)	Relative Area (%)	ee (%)
1	11.179	99.469	99
2	14.250	0.531	

Supplementary Fig. 122. HPLC Spectra of **31**

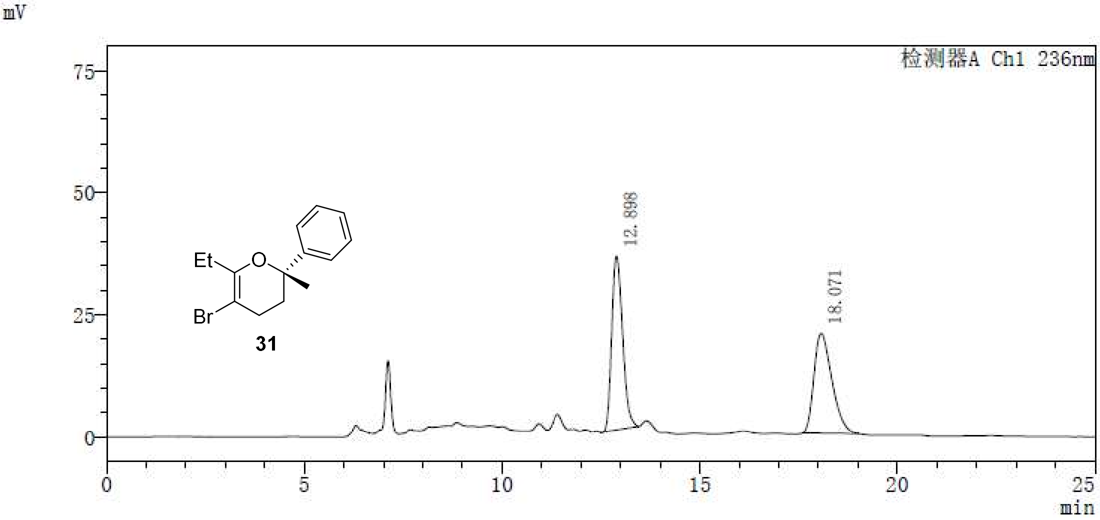

Peak	Retention Time (min)	Relative Area (%)
1	12.898	51.262
2	18.071	48.738

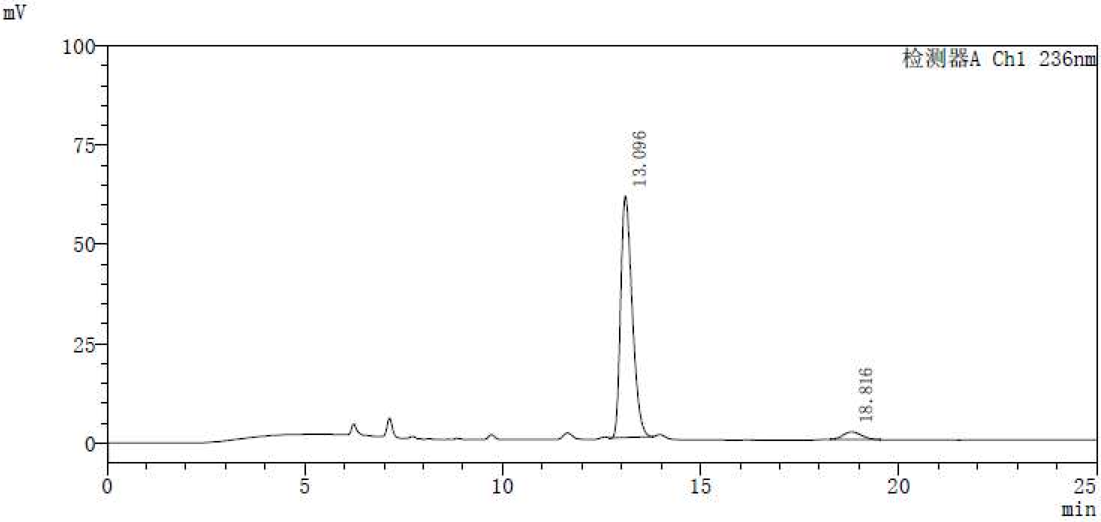

Peak	Retention Time (min)	Relative Area (%)	ee (%)
1	13.096	95.123	90
2	18.816	4.877	

Supplementary Fig. 123. HPLC Spectra of **32**

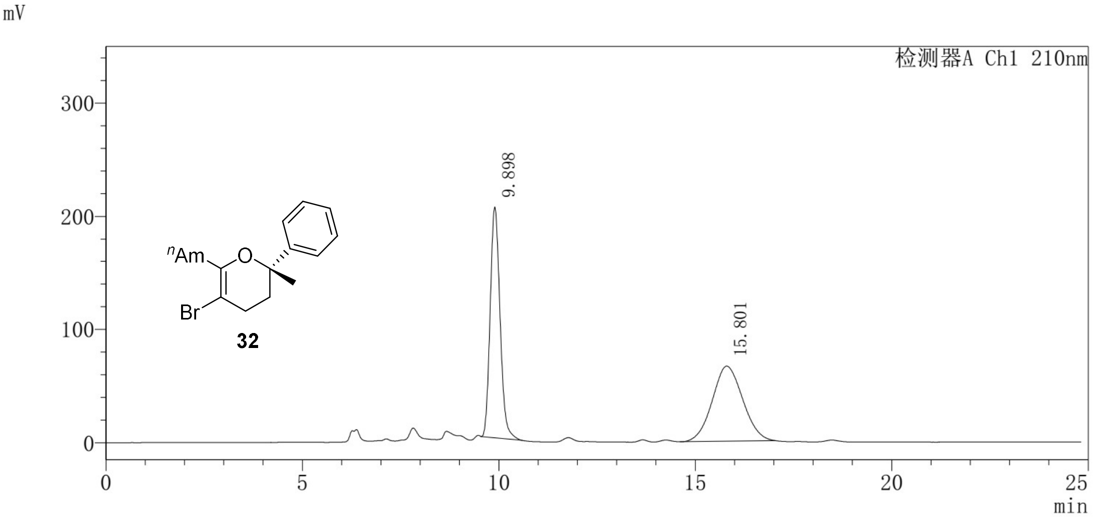

Peak	Retention Time (min)	Relative Area (%)
1	9.898	49.774
2	15.801	50.226

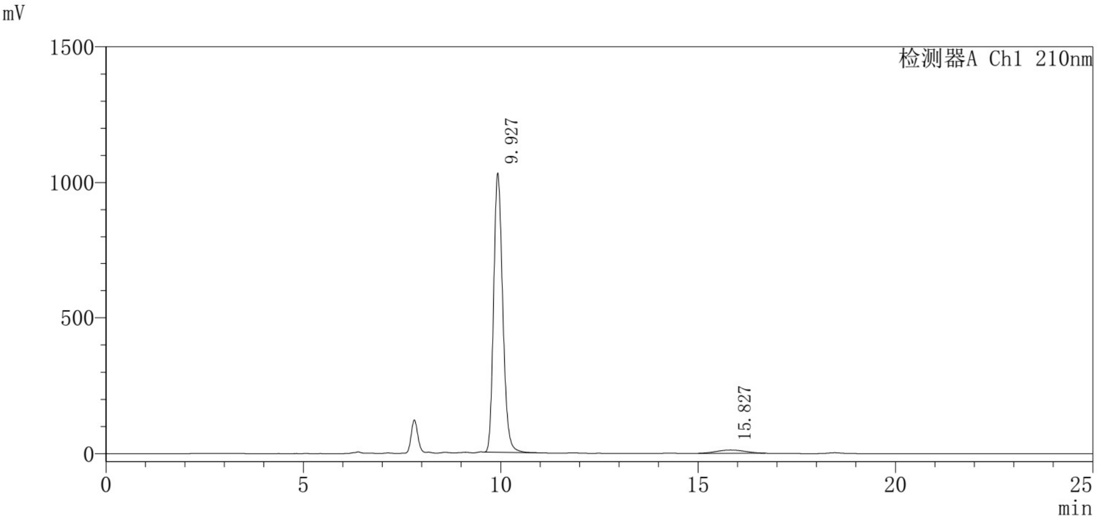

Peak	Retention Time (min)	Relative Area (%)	ee (%)
1	9.927	96.452	93
2	15.827	3.548	

Supplementary Fig. 124. HPLC Spectra of **33**

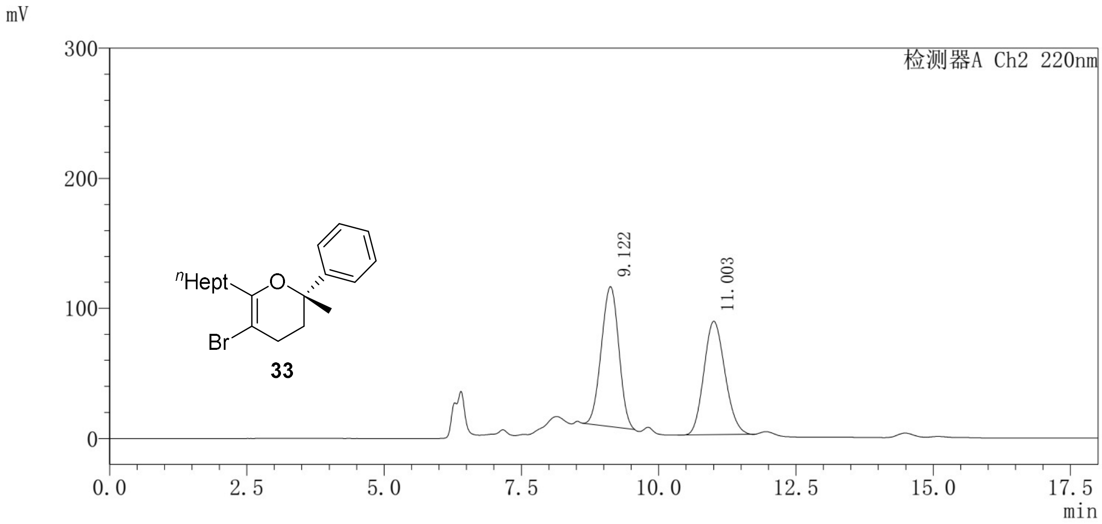

Peak	Retention Time (min)	Relative Area (%)
1	9.122	50.676
2	11.003	49.324

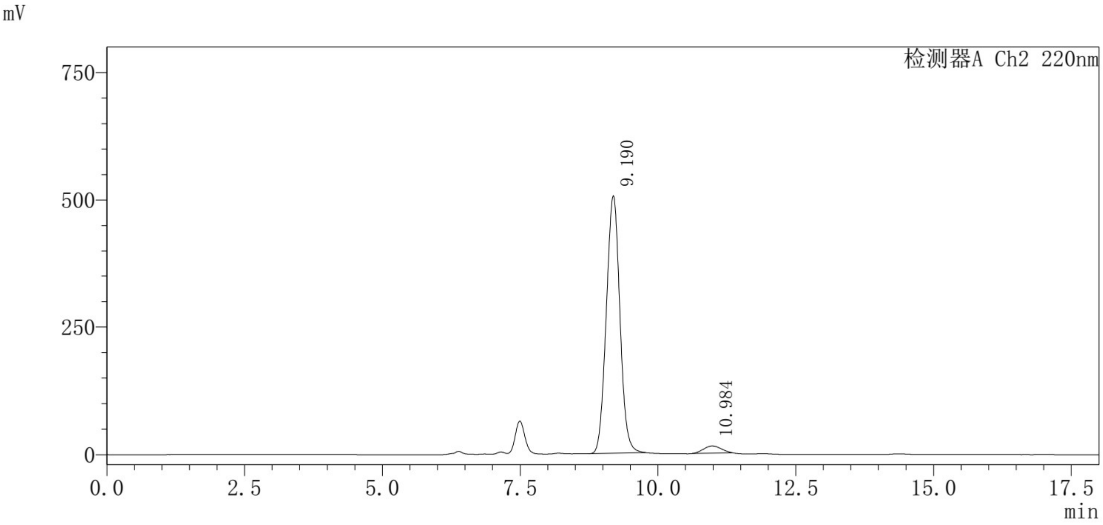

Peak	Retention Time (min)	Relative Area (%)	ee (%)
1	9.190	96.531	93
2	10.984	3.469	

Supplementary Fig. 125. HPLC Spectra of **34**

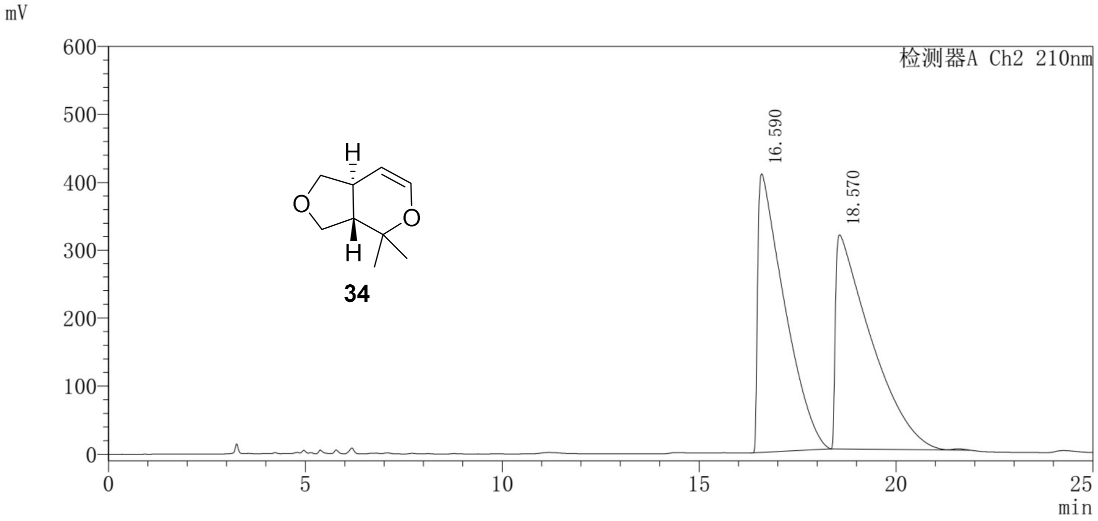

Peak	Retention Time (min)	Relative Area (%)
1	16.590	49.429
2	18.570	50.571

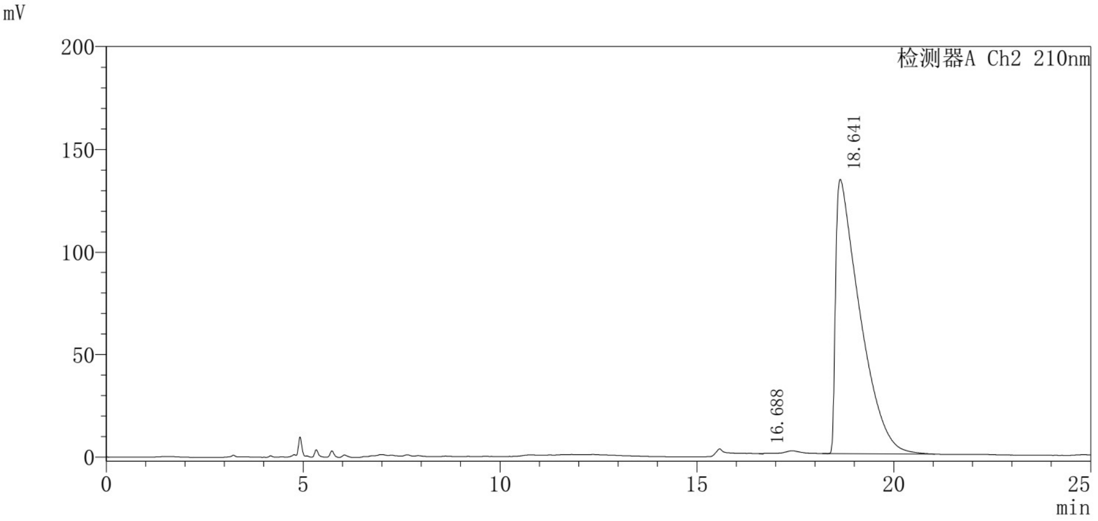

Peak	Retention Time (min)	Relative Area (%)	ee (%)
1	16.688	0.028	99.9
2	18.641	99.972	

Supplementary Fig. 126. HPLC Spectra of **33**

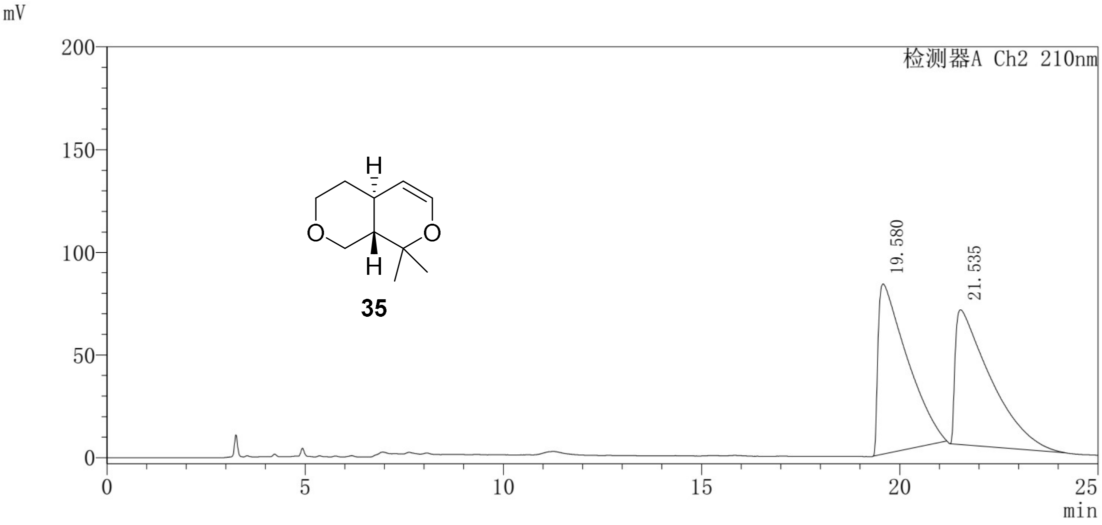

Peak	Retention Time (min)	Relative Area (%)
1	19.580	50.792
2	21.535	49.208

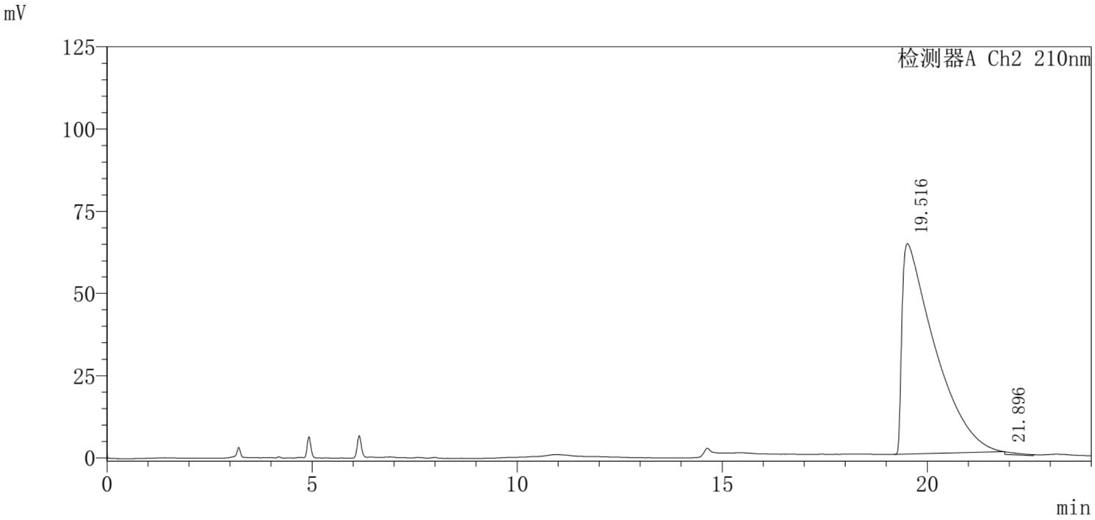

Peak	Retention Time (min)	Relative Area (%)	ee (%)
1	19.516	99.432	99
2	21.896	0.568	

Supplementary Fig. 127. HPLC Spectra of **38**

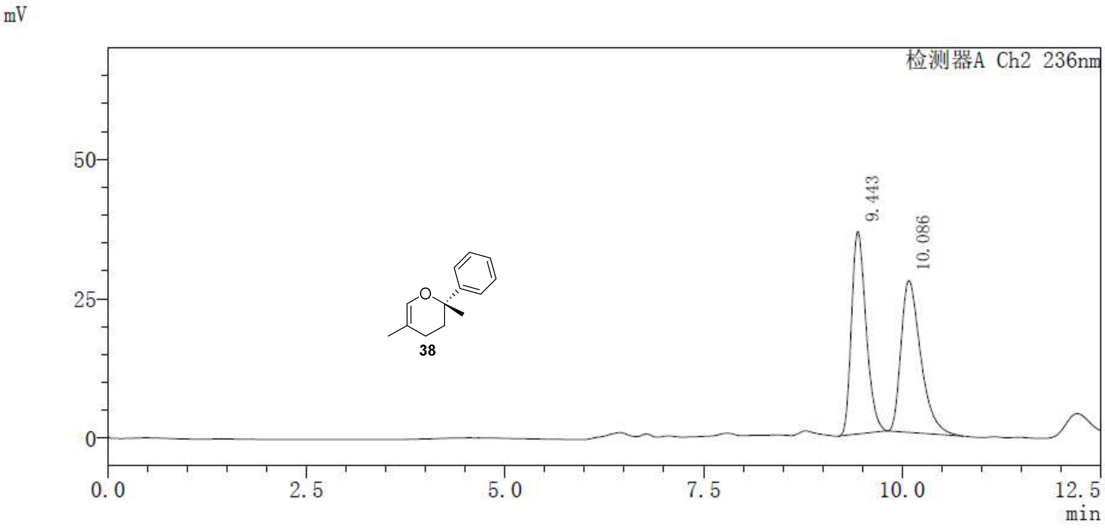

Peak	Retention Time (min)	Relative Area (%)
1	9.443	49.600
2	10.086	50.400

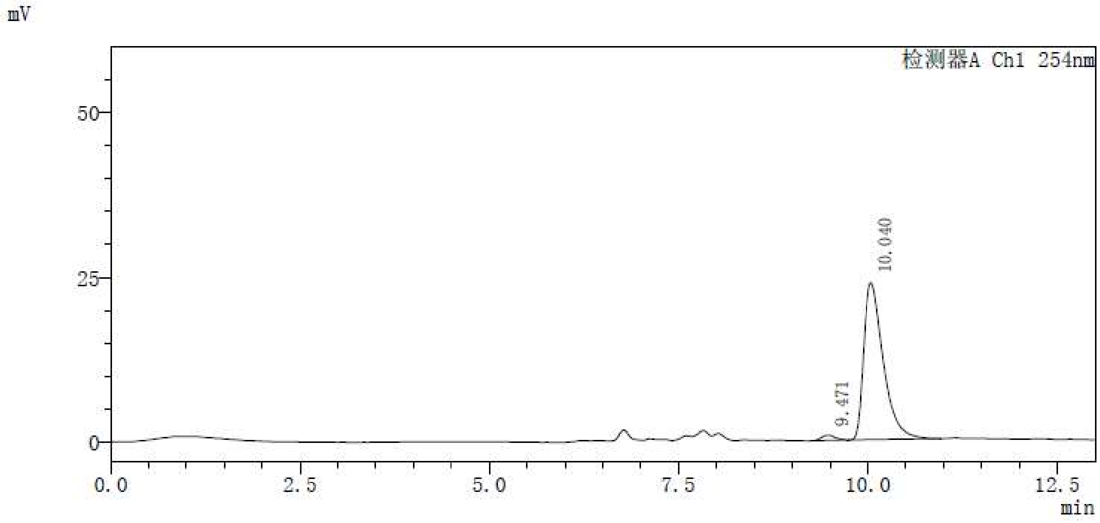

Peak	Retention Time (min)	Relative Area (%)	ee (%)
1	9.471	2.252	95
2	10.040	97.748	

Supplementary Fig. 128. HPLC Spectra of **39**

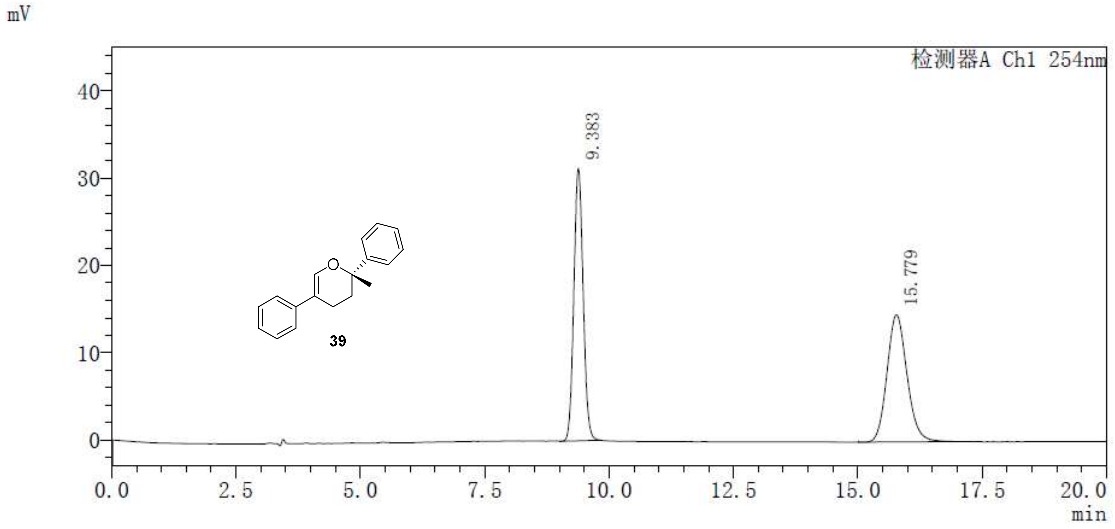

Peak	Retention Time (min)	Relative Area (%)
1	9.383	49.994
2	15.779	50.006

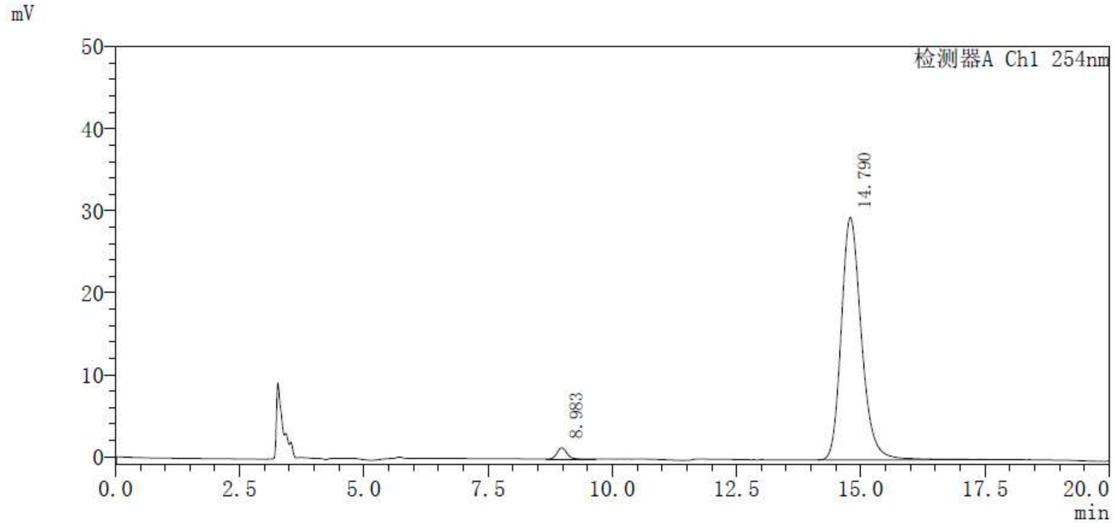

Peak	Retention Time (min)	Relative Area (%)	ee (%)
1	8.983	2.207	95
2	14.790	97.793	

Supplementary Fig. 129. HPLC Spectra of **40**

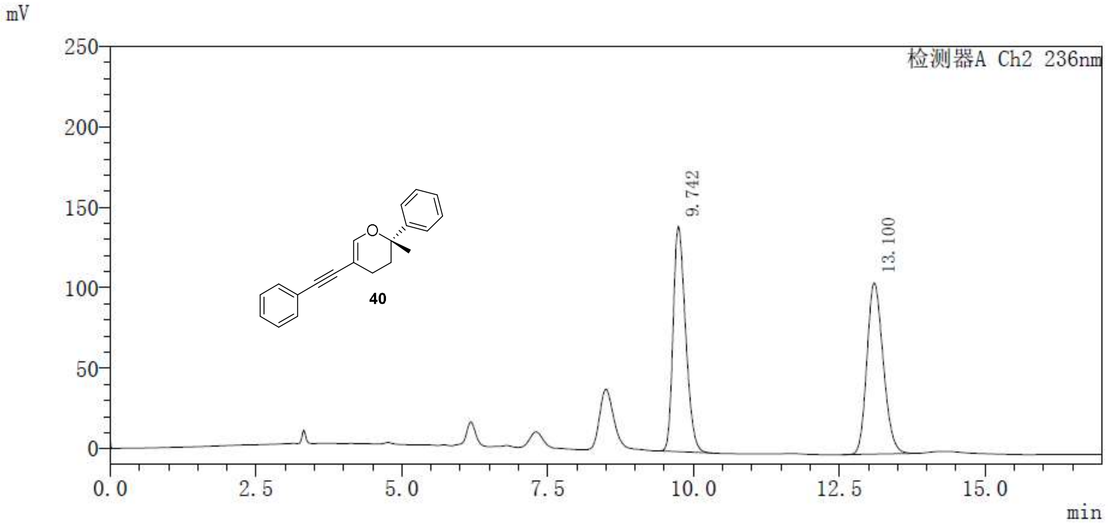

Peak	Retention Time (min)	Relative Area (%)
1	9.742	50.132
2	13.100	49.868

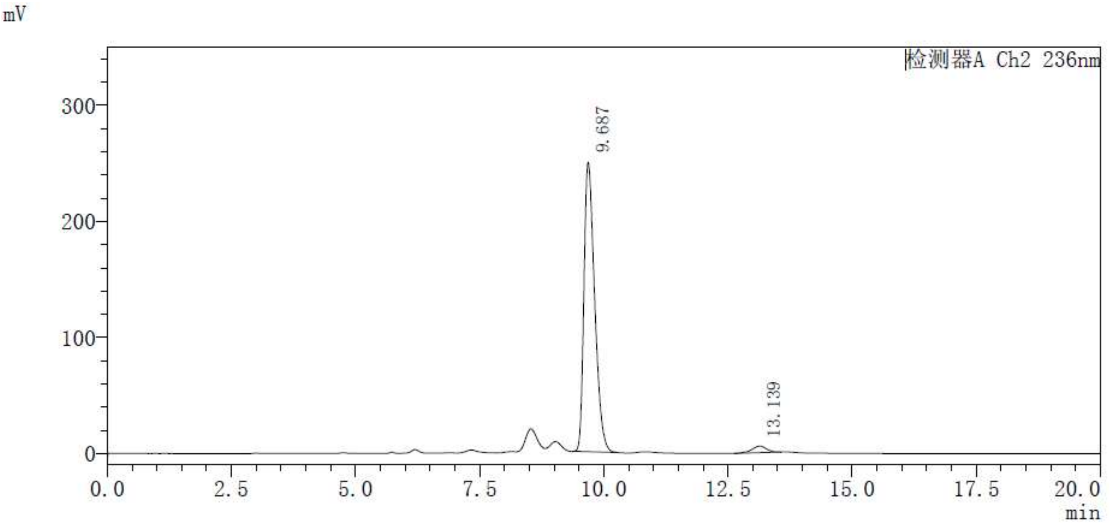

Peak	Retention Time (min)	Relative Area (%)	ee (%)
1	9.687	97.083	94
2	13.139	2.917	

Supplementary Fig. 130. HPLC Spectra of **41**

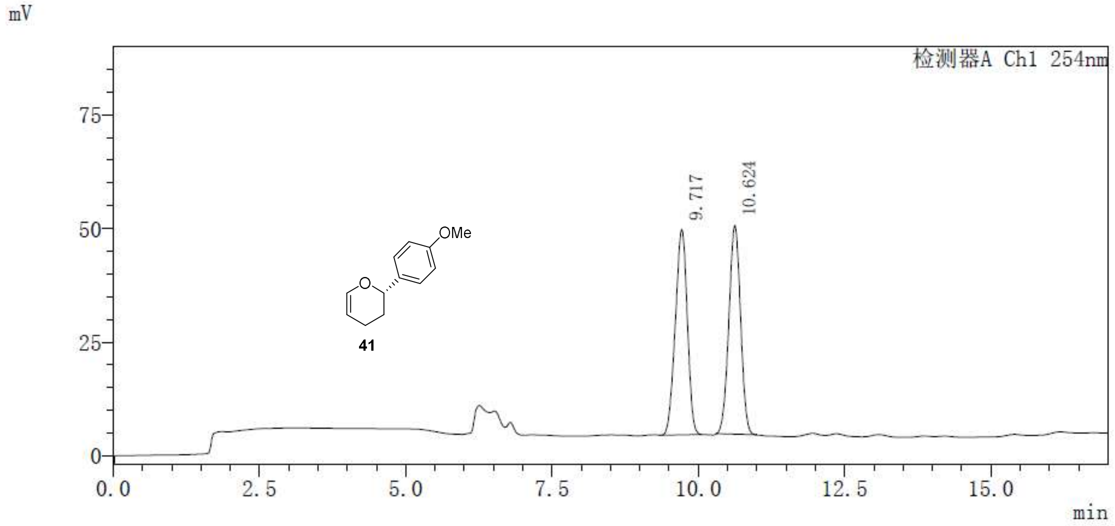

Peak	Retention Time (min)	Relative Area (%)
1	9.717	50.112
2	10.624	49.888

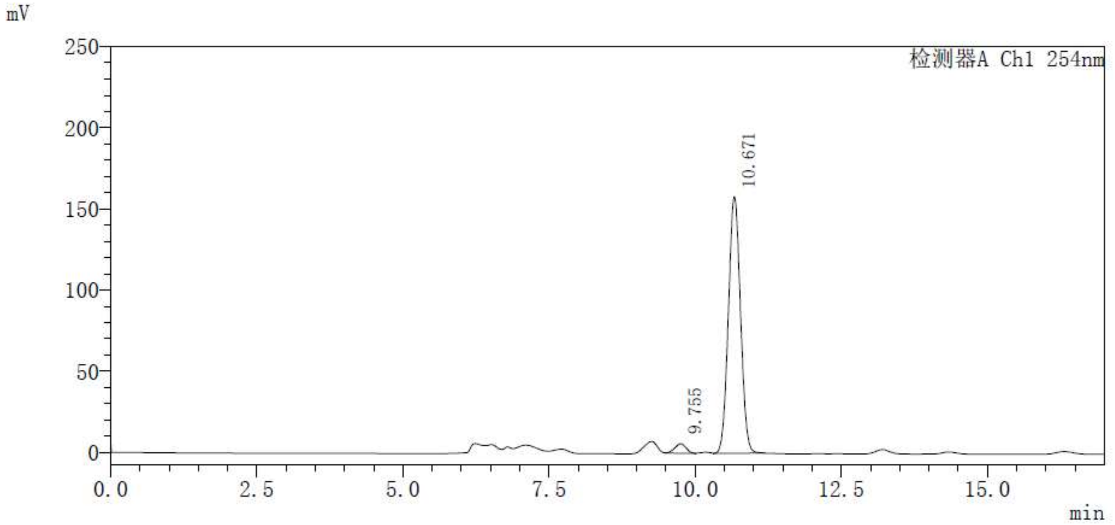

Peak	Retention Time (min)	Relative Area (%)	ee (%)
1	9.755	3.246	94
2	10.671	96.754	

Supplementary Fig. 131. HPLC Spectra of **42**

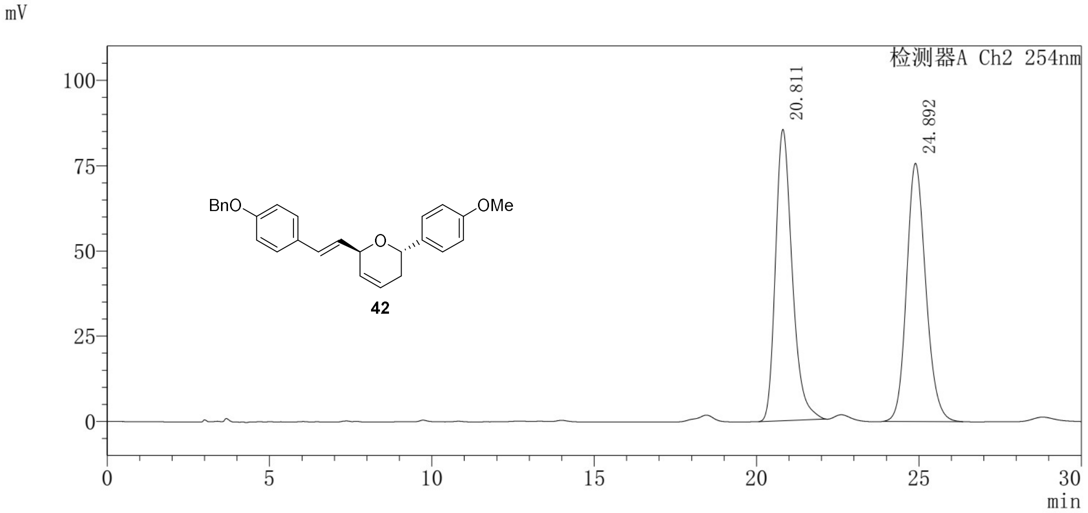

42

Peak	Retention Time (min)	Relative Area (%)
1	20.811	49.566
2	24.892	50.434

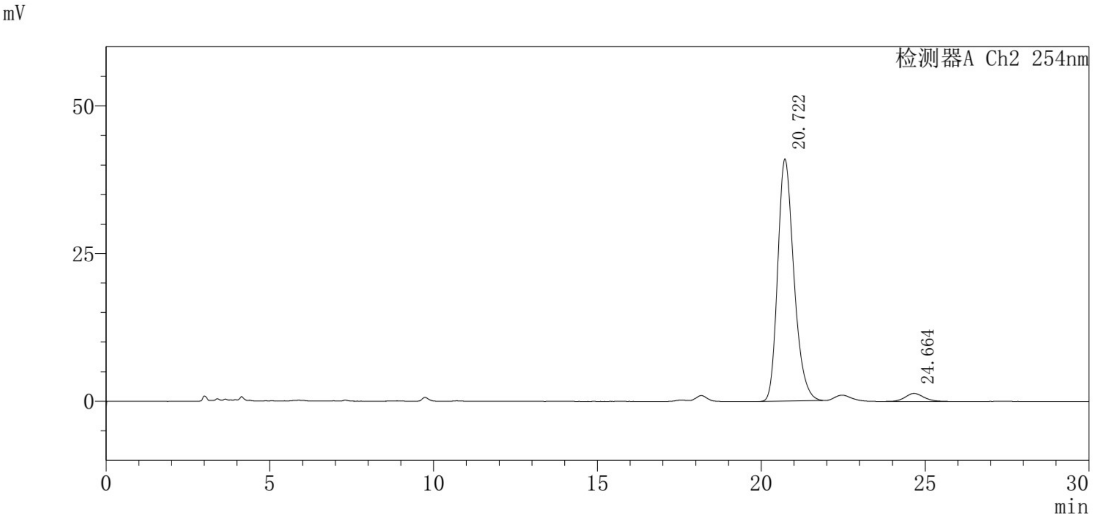

Peak	Retention Time (min)	Relative Area (%)	ee (%)
1	20.722	96.346	93
2	24.664	3.654	

Supplementary Fig. 132. HPLC Spectra of **43**

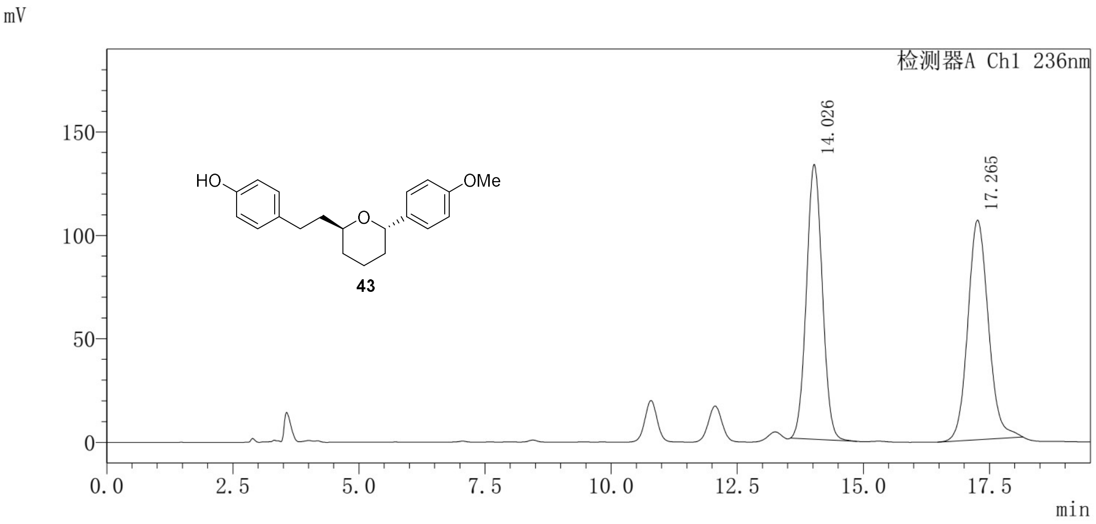

Peak	Retention Time (min)	Relative Area (%)
1	14.026	49.202
2	17.265	50.798

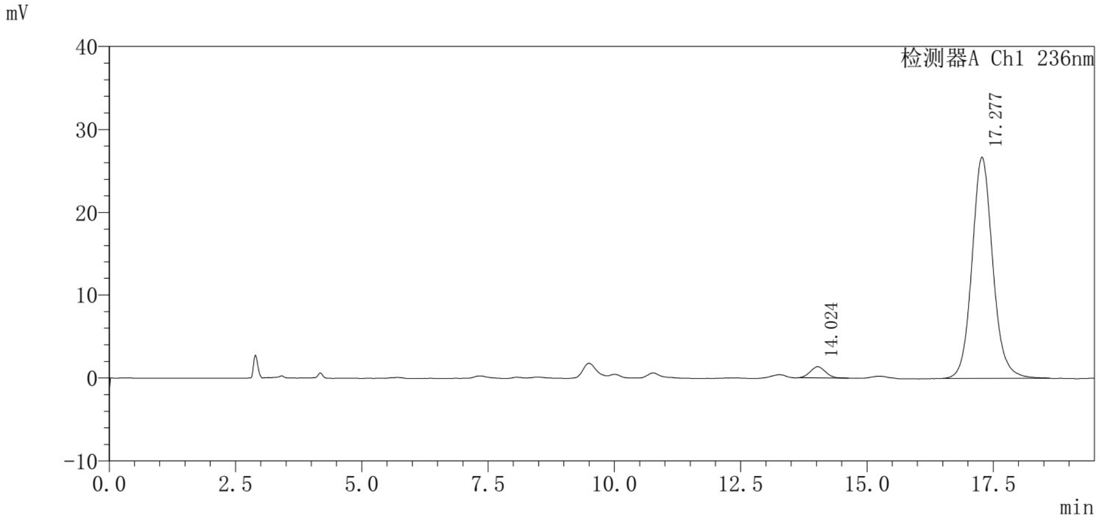

Peak	Retention Time (min)	Relative Area (%)	ee (%)
1	14.024	3.519	93
2	17.277	96.481	

Supplementary Fig. 133. HPLC Spectra of **44**

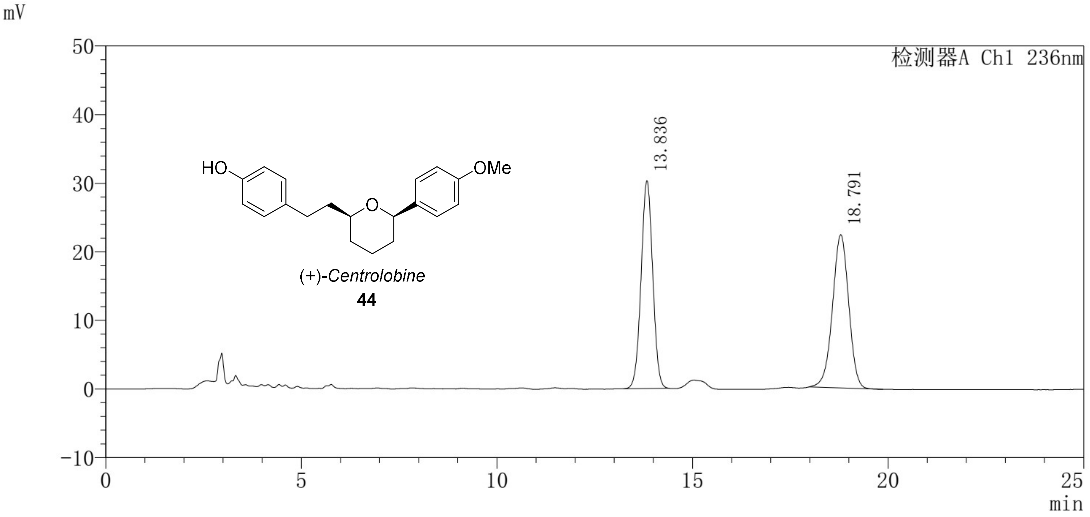

Peak	Retention Time (min)	Relative Area (%)
1	13.836	49.336
2	18.791	50.664

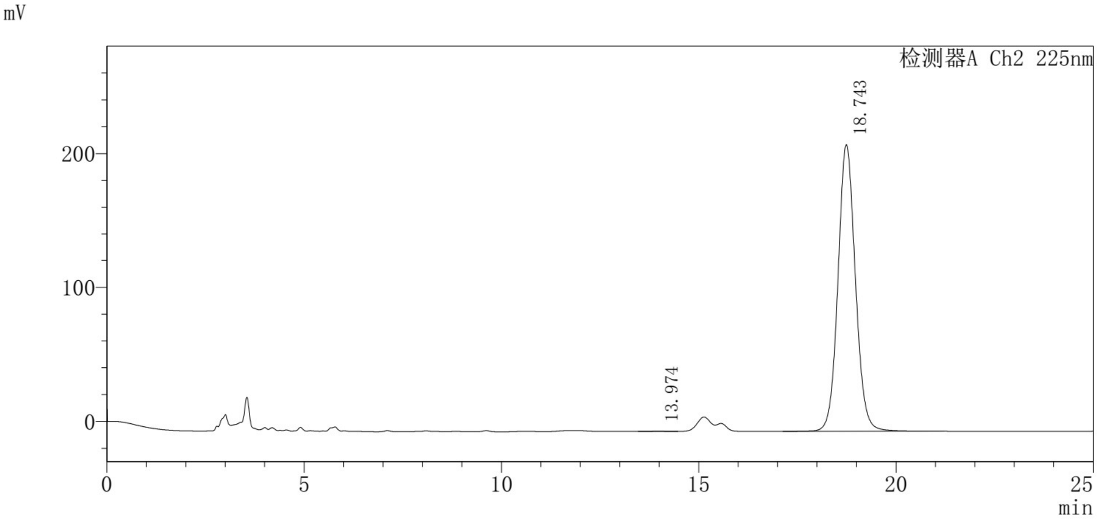

Peak	Retention Time (min)	Relative Area (%)	ee (%)
1	13.974	0.077	99.8
2	18.743	99.923	

4. Supplementary References

[1] Nicolaou, K. C., Brenzovich, W. E., Bulger, P. G. & Francis, T. M. Synthesis of *iso*-epoxy-amphidinolide N and *des*-epoxy-caribenolide I structures. Initial forays. *Org. Biomol. Chem.* **4**, 2119–2157 (2006).

[2] Beltaïef, I., Hbaïeb, S., Besbes, R., Amri, H., Villiéras, M. & Villiéras, J. A New and Efficient Method for the Isomerization of Secondary Functional Allylic Alcohols into their Primary Isomers. *Synthesis* 1765–1768 (1998).

[3] Bikshapathi, R., Prathima, P. S. & Rao, V. J. Hypervalent iodine catalysis for selective oxidation of Baylis–Hillman adducts via in situ generation of o-iodoxybenzoic acid (IBX) from 2-iodosobenzoic acid (IBA) in the presence of oxone. *New J. Chem.* **40**, 10300–10304 (2016).

[4] Mathre, D. J., Jones, T. K., Xavier, L. C., Blacklock, T. J., Reamer, R. A., Mohan, J. J., Jones, E. T. T., Hoogsteen, K., Baum, M. W. & Grabowski, E. J. J. A Practical Enantioselective Synthesis of α,α-Diaryl-2-pyrrolidinemethanol. Preparation and Chemistry of the Corresponding Oxazaborolidines. *J. Org. Chem.* **56**, 751–762 (1991).

[5] Corey, E. J., Shibata, T. & Lee, T. W. Asymmetric Diels-Alder Reactions Catalyzed by a Triflic Acid Activated Chiral Oxazaborolidine. *J. Am. Chem. Soc.* **124**, 3808−3809 (2002).

[6] Ko, C., Feltenberger, J. B., Ghosh, S. K. & Hsung, R. P. Gassman's intramolecular [2 + 2] cationic cycloaddition. Formal total syntheses of raikovenal and epi-Raikovenal. *Org. Lett.* **10**, 1971–1974 (2008).

[7] Zhang, Z., Qiao, J., Wang, D., Han, L. & Ding, R. Synthesis of isoflavones by room-temperature nickel-catalyzed cross-couplings of 3-iodo(bromo)chromones with arylzincs. *Mol. Divers.* **18**, 245−251 (2014).

[8] Nakatsuji, H., Kamada, R., Kitaguchi, H. & Tanabe, Y. Dehydration-type Ti-Claisen condensation (carbonhomologation) of α-heteroatom-substituted acetates with alkyl aormates: utilization as (Z)-stereodefined cross-coupling partners and application to concise synthesis of Strobilurin A. *Adv. Synth. Catal.* **359**, 3865−3879 (2017).

[9] Wu, Y.-T., Noltemeyer, M. & de Meijere, A. Cascade reactions of β-Amino-Substituted α,β-unsaturated Fischer carbene complexes with 1,5-dien-3-ynes as a convenient access to ring-annelated benzene derivatives. Eur. *J. Org. Chem.* **2005**, 2802−2810 (2005).

[10] Srikanth, G. S. C. & Castle, S. L. Advances in radical conjugate additions. *Tetrahedron* **61**, 10377−10441 (2005).

[11] Pandey, A. K., Sharma, R., Shivahare, R., Arora, A., Rastogi, N., Gupta, S. & Chauhan, P. M. S. Synthesis of perspicamide A and related diverse analogues: their bioevaluation as potent antileishmanial agents *J. Org. Chem.* **78**, 1534–1546 (2013).

[12] Larock, R. C., Gong, W. H. & Baker, B. E. Improved procedures for the palladium-catalyzed intermolecular arylation of cyclic alkenes. *Tetrahedron Lett.* **30**, 2603−2606 (1989).

[13] Reddy, C. R., Madhavi, P. P. Chandrasekhar, S. *Synthesis* 2939−2942 (2008).

[14] Zhou, P., Cai, Y., Zhong, X., Luo, W., Kang, T., Li, J., Liu, X., Lin, L. & Feng, X. *ACS Catal.* **6**, 7778−7783 (2016).

[15] Frisch, M. J., Trucks, G. W., Schlegel, H. B., Scuseria, G. E., Robb, M. A., Cheeseman, J. R., Scalmani, G., Barone, V., Men-nucci, B., Petersson, G. A., Nakatsuji, H., Caricato, M., Li, X., Hratchian, H. P., Izmaylov, A. F., Bloino, J., Zheng, G., Sonnenberg, J. L., Hada, M., Ehara, M., Toyota, K., Fukuda, R., Hasegawa, J., Ishida, M., Nakajima, T., Honda, Y., Kitao, O., Nakai, H., Vreven, T., Montgomery Jr., J. A., Peralta, J. E., Ogliaro, F., Bearpark, M., Heyd, J. J., Brothers, E., Kudin, K. N., Staroverov, V. N., Ko-bayashi, R., Normand, J., Raghavachari, K., Rendell, A., Burant, J. C., Iyen-gar, S. S., Tomasi, J., Cossi, M., Rega, N., Millam, J. M., Klene, M., Knox, J. E., Cross, J. B., Bakken, V., Adamo, C., Jaramillo, J., Gomperts, R., Stratmann, R. E., Yazyev, O., Austin, A. J., Cammi, R., Pomelli, C., Ochterski, J. W., Martin, R. L., Morokuma, K., Zakrzewski, V. G., Voth, G. A., Salvador, P., Dannenberg, J. J., Dapprich, S., Daniels, A. D., Farkas, Ö., Foresman, J. B., Ortiz, J. V., Cio-slowski, J. & Fox, D. J. Gaussian 09, revision D.01; Gaussian, Inc.: Wallingford, CT, 2013.

[16] Becke, A. D. Density-functional thermochemistry. III. The role of exact exchange. *J. Chem. Phys.* **98**, 5648–5652 (1993).

[17] Lee, C., Yang, W. & Parr, R. G. Development of the Colle-Salvetti correlation-energy formula into a functional of the electron density. *Phys. Rev. B* **37**, 785–789 (1988).

[18] Marenich, A. V., Cramer, C. J. & Truhlar, D. G. Universal Solvation Model Based on Solute Electron Density and on a Continuum Model of the Solvent Defined by the Bulk Dielectric Constant and Atomic Surface Tensions. *J. Phys. Chem. B* **113**, 6378–6396 (2009).

[19] Zhao, Y. & Truhlar, D. G. The M06 suite of density functionals for main group thermochemistry, thermochemical kinetics, noncovalent interactions, excited states, and transition elements: two new functionals and systematic testing of four M06-class functionals and 12 other functionals. *Theor. Chem. Acc.* **120**, 215–241 (2008).
