# Supplementary material for: Catalytic asymmetric oxa-Diels–Alder reaction of acroleins with simple alkenes
Source: Nat Commun. 2023 Jun 14;14:3511. doi: 10.1038/s41467-023-39184-z (PMC10267187; doi:10.1038/s41467-023-39184-z)
Supplement: Supplementary file 2 — Description of Additional Supplementary Files [file 41467_2023_39184_MOESM2_ESM.docx]

**Description of Additional Supplementary Files**

File Name: Supplementary Data 1
Description: Crystallographic data for compound **9** (CCDC 2131534), **17**(CCDC 2143819) and **24** (CCDC 2131535).
